# Functional Diversification of SRSF Protein Kinase to Control Ubiquitin-Dependent Neurodevelopmental Signaling

**DOI:** 10.1016/j.devcel.2020.09.025

**Published:** 2020-12-07

**Authors:** Francisco Bustos, Anna Segarra-Fas, Gino Nardocci, Andrew Cassidy, Odetta Antico, Lindsay Davidson, Lennart Brandenburg, Thomas J. Macartney, Rachel Toth, C. James Hastie, Jennifer Moran, Robert Gourlay, Joby Varghese, Renata F. Soares, Martin Montecino, Greg M. Findlay

**Affiliations:** 1 The MRC Protein Phosphorylation and Ubiquitylation Unit, School of Life Sciences, the University of Dundee, Dundee DD1 5EH, UK; 2 Institute of Biomedical Sciences and FONDAP Center for Genome Regulation, Universidad Andrés Bello, Santiago, Chile; 3 Tayside Centre for Genomic Analysis, School of Medicine, University of Dundee, Dundee DD1 9SY, UK; 4 School of Life Sciences, The University of Dundee, Dundee DD1 5EH, UK

**Keywords:** metazoan evolution, development, signal transduction, protein kinase, protein phosphorylation, ubiquitin signaling, stem cells, transcriptomics, neural development, neurodevelopmental disorders

## Abstract

Conserved protein kinases with core cellular functions have been frequently redeployed during metazoan evolution to regulate specialized developmental processes. The Ser/Arg (SR)-rich splicing factor (SRSF) protein kinase (SRPK), which is implicated in splicing regulation, is one such conserved eukaryotic kinase. Surprisingly, we show that SRPK has acquired the capacity to control a neurodevelopmental ubiquitin signaling pathway. In mammalian embryonic stem cells and cultured neurons, SRPK phosphorylates Ser-Arg motifs in RNF12/RLIM, a key developmental E3 ubiquitin ligase that is mutated in an intellectual disability syndrome. Processive phosphorylation by SRPK stimulates RNF12-dependent ubiquitylation of nuclear transcription factor substrates, thereby acting to restrain a neural gene expression program that is aberrantly expressed in intellectual disability. SRPK family genes are also mutated in intellectual disability disorders, and patient-derived SRPK point mutations impair RNF12 phosphorylation. Our data reveal unappreciated functional diversification of SRPK to regulate ubiquitin signaling that ensures correct regulation of neurodevelopmental gene expression.

## Highlights

• SRPK has acquired a developmental function regulating RNF12
• RNF12 phosphorylation by SRPK promotes E3 ligase activity and nuclear anchoring
• SRPK-RNF12 signaling to the REX1 transcription factor controls neural genes
• This signaling network is disrupted in neurodevelopmental disorders

## Graphical Abstract

**Figure:**
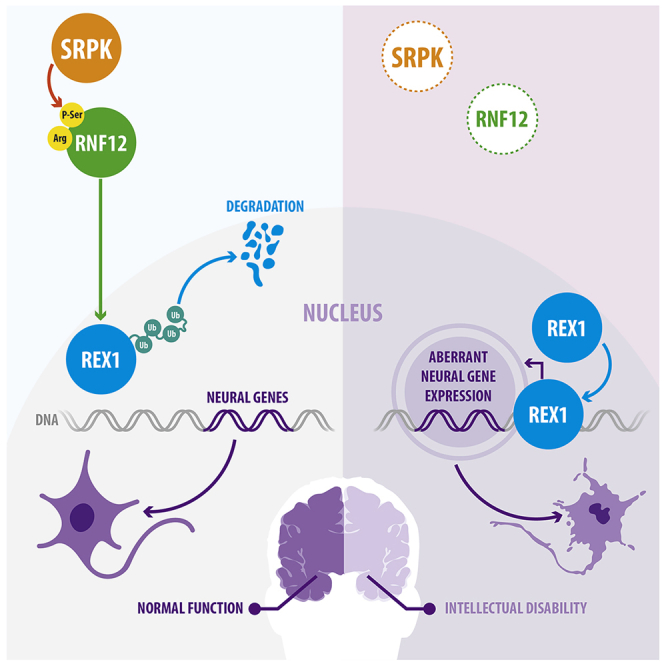

## Teaser

Bustos et al. show that SRPK splicing factor kinase has acquired a developmental function— phosphorylating the RNF12 E3 ubiquitin ligase to promote degradation of the transcription factor, REX1. This signaling pathway regulates a neurodevelopmental gene expression program and is mutated in patients with neurodevelopmental disorders.

## Introduction

Signal transduction by protein kinases controls all aspects of eukaryotic biology (Cohen, 2002), from metabolism to complex developmental programs. As such, protein kinases involved in core eukaryotic processes have been redeployed during metazoan evolution to regulate specialized processes required for multicellular life. This is illustrated by acquisition of increasingly complex roles of the mitogen activated protein kinase (MAPK) signaling pathway from yeast to metazoans. In yeast, MAPK signaling controls simple unicellular functions, such as sensing mating pheromones and environmental stress (Chen and Thorner, 2007), while metazoan MAPK signaling has acquired the ability to regulate complex multicellular processes, including lineage-specific differentiation (Cowley et al., 1994; Traverse et al., 1992). Other highly conserved protein kinases may have undergone similar “functional diversification” to acquire new functions, thereby facilitating metazoan evolution.

In principle, functional diversification of protein kinases can be achieved via several non-mutually exclusive mechanisms: (1) evolutionary wiring of protein kinase pathways to newly evolved cell-cell communication systems that control metazoan biology, such as receptor tyrosine kinases (Lim and Pawson, 2010), (2) evolution of new kinase-substrate relationships, and (3) evolution of specific kinase activity or expression profiles that differ according to developmental time and tissue context. These mechanisms individually or in combination have the capacity to drive functional diversification, enabling highly conserved eukaryotic protein kinases to evolve novel functions in the control of key metazoan processes.

The Ser-Arg rich splicing factor (SRSF) protein kinase (SRPK) family represent a prominent case study for functional diversification, as they perform core functions in mRNA splicing regulation that are thought to be conserved throughout eukaryotes (Dagher and Fu, 2001; Gui et al., 1994b; Siebel et al., 1999; Yeakley et al., 1999). SRPKs phosphorylate SRSFs, modulating their subcellular localization and regulating spliceosome assembly (Cao et al., 1997; Koizumi et al., 1999; Mathew et al., 2008; Xiao and Manley, 1997). Few non-splicing functions of SRPKs have been reported (Gou et al., 2020; Hong et al., 2012; Wang et al., 2017), and it remains unclear whether SRPKs have evolved further regulatory roles in metazoans. However, SRPK family members exhibit highly tissue-specific expression profiles (Nakagawa et al., 2005; Wang et al., 1998), suggesting that these protein kinases may indeed perform specialized functions required for multicellular development.

Here, we show that SRPKs have undergone functional diversification to acquire a critical role in mammalian development. Surprisingly, SRPK activity does not make a major contribution to SRSF phosphorylation or to a key splicing switch in mammalian embryonic stem cells. Instead, SRPK controls a ubiquitin signaling pathway to regulate expression of neurodevelopmental genes. In this pathway, SRPK phosphorylates a Ser-Arg-rich regulatory motif on the E3 ubiquitin ligase RNF12/RLIM (Barakat et al., 2011; Shin et al., 2010, 2014; Zhang et al., 2012), which is mutated in the X-linked intellectual disability disorder Tonne-Kalscheuer syndrome (TOKAS) (Frints et al., 2019; Hu et al., 2016; Tønne et al., 2015). Processive RNF12 phosphorylation by SRPK stimulates ubiquitylation of transcription factor substrates to modulate expression of neural genes. Data mining indicates that SRPK family genes are also mutated in intellectual disability disorders, and SRPK3 point mutations, identified in patients, impair RNF12 phosphorylation. Thus, we uncover a previously unappreciated function for SRPK in neurodevelopmental signaling, indicating that functional diversification during eukaryotic evolution has enabled this highly conserved kinase family to govern complex metazoan processes beyond splicing regulation.

## Results

### SRPK Activity Plays a Minor Role in Ser-Arg Rich Splicing Factor (SRSF) Phosphorylation in Embryonic Cells

**Figure 1:**
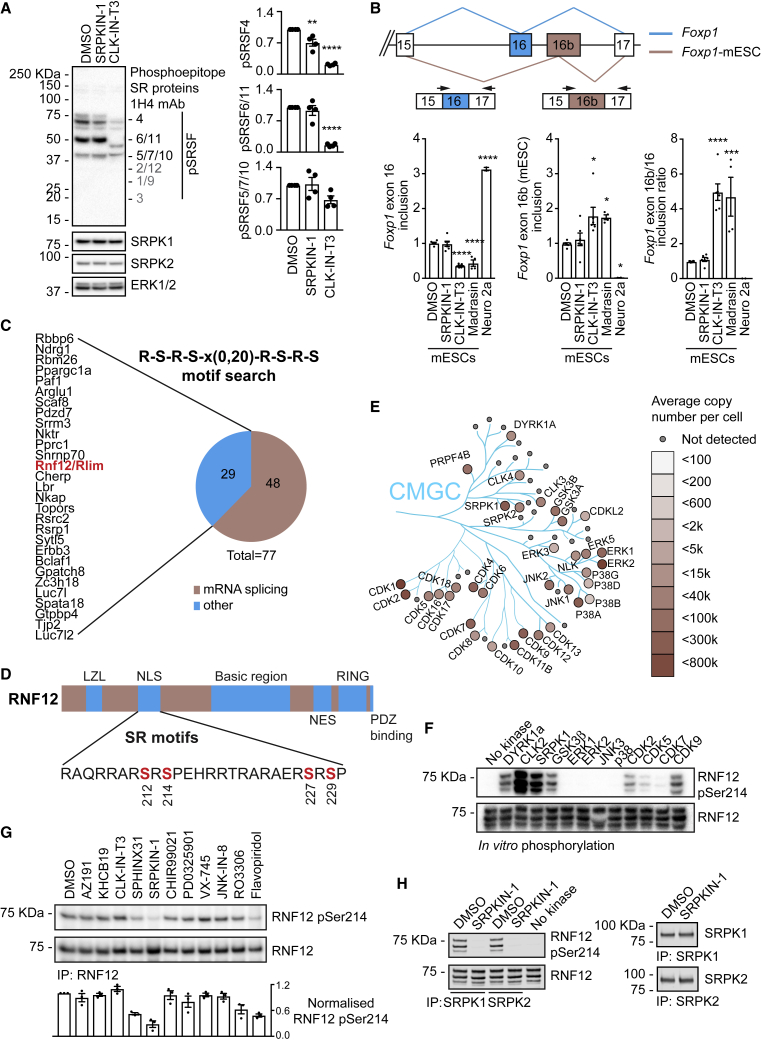
Functional Diversification of SRPK to Control Developmental Ubiquitin Signaling (A) Wild-type (WT) mESCs were treated with 10 μM SRPKIN-1 or CLK-IN-T3 for 4 h, and phosphorylation of Ser-Arg rich splicing factors (SRSF) was assessed (Left). SRSF phosphorylation, SRPK1, SRPK2, and ERK1/2 levels were determined by immunoblotting. Expected positions of SRSFs that are not detected are shown in gray. Quantification of SRSF phosphorylation (Right). Data represented as mean ± SEM (n = 4). One-way ANOVA followed by Tukey’s multiple comparisons test; confidence level 95%. pSRSF4: (^∗∗^) p = 0.0032, (^∗∗∗∗^) p < 0.0001, pSRSF6/11: (^∗∗∗∗^) p < 0.0001. (B) Splice variants of *Foxp1* mRNA including mutually exclusive exons 16 (*Foxp1*, GenBank: NM_053202.2, cyan) or 16b (*Foxp1*-ESC, GenBank: XM_030255074.1, tan) (Top). mESCs were treated with 1 μM SRPKIN-1 or CLK-IN-T3, or 10 μM Madrasin for 8 h, and *Foxp1* exon 16-16b incorporation determined using specific quantitative RT-PCR primers. Neuro 2a is a control for exon 16b exclusion in differentiated cells (Bottom). Data represented as mean ± SEM (n = 3). One-way ANOVA followed by Tukey’s multiple comparisons test; confidence level 95%. Exon 16 inclusion: (^∗∗∗∗^) p < 0.0001, Exon 16b inclusion: (^∗^) p = 0.0164, p = 0.0485, and p = 0.0489 (left to right). Ratio exon 16b/16: (^∗∗∗∗^) p < 0.0001, (^∗∗∗^) p = 0.0003. (C) SRPK substrates predicted using ScanProsite and grouped according to UniProt functions. (D) RNF12 phosphorylation sites detected by mass-spectrometry. LZL, leucine-zipper like; NLS, nuclear localization signal; NES, nuclear export signal; RING, RING E3 ubiquitin ligase catalytic domain. (E) CMGC family kinase copy numbers in mESCs determined by quantitative proteomics and represented using Kinoviewer. (F) CMGC kinase (200 mU) phosphorylation of the RNF12 SR-motif *in vitro* was determined by immunoblotting for RNF12 phospho-Ser214 and total RNF12. (G) mESCs were treated with 10 μM of the following kinase inhibitors: AZ-191 (DYRK1B), KH-CB19 (CLK-DYRK), CLK-IN-T3 (CLK), SPHINX31 (SRPK1), SRPKIN-1 (pan-SRPK), CHIR-99021 (GSK-3), PD-0325901 (MEK1/2), VX-745 (p38), JNK-IN-8 (JNK), RO-3306 (CDK1), and flavopiridol (CDK7/9) for 4 h and RNF12 SR-motif phosphorylation determined by immunoblotting for RNF12 phospho-Ser214 and total RNF12. Normalized RNF12 Ser214 phosphorylation is shown below. Data represented as mean ± SEM (n = 3). (H) SRPKIN-1 inhibition of SRPKs *in vivo* was determined by pre-treatment of mESCs with 10 μM SRPKIN-1 for 4 h followed by SRPK1 or SRPK2 immunoprecipitation kinase assay using RNF12 as a substrate. RNF12 SR-motif phosphorylation was analyzed by immunoblotting for RNF12 phospho-Ser214 and RNF12. SRPK1 and SRPK2 levels are shown as a loading control, Related to Figure S1; Tables S1 and S2.

SRPKs are thought to be key players in splicing regulation, controlling spliceosome assembly and activity (Dagher and Fu, 2001; Yeakley et al., 1999) via phosphorylation of SRSFs (Long and Caceres, 2009; Roscigno and Garcia-Blanco, 1995; Wu and Maniatis, 1993). Although splicing plays a critical role in stem cell regulation (Gabut et al., 2011; Salomonis et al., 2010), the first function of SRPK during early development in mammals has only recently been reported (Gou et al., 2020). This prompted us to examine the role of SRPK in mouse embryonic stem cells (mESCs). We first sought to confirm that SRPK activity is required for SRSF phosphorylation using an antibody that detects phosphorylated Ser-Arg-rich motifs. Surprisingly, in contrast with reports from somatic cells (Hatcher et al., 2018), phosphorylation of the major phosphorylated SRSF proteins in mESCs is either not significantly altered (SRSF6/11, SRSF5/7/10) or only slightly inhibited (SRSF4) by the selective pan-SRPK inhibitor, SRPKIN-1 (Hatcher et al., 2018) (Figure 1A). In contrast, treatment of mESCs with CLK-IN-T3, a selective inhibitor of the closely related CLK kinases (Funnell et al., 2017), which also phosphorylate SRSF splicing factors (Colwill et al., 1996), leads to widespread, robust inhibition of SRSF phosphorylation (Figure 1A). Our results therefore suggest that SRPKs are not the major SRSF kinases in mESCs.

This unexpected observation prompted us to examine whether SRPK activity is required for a key mESC alternative splicing switch, namely, inclusion of a specific exon within the developmental transcription factor FOXP1. mESCs express *Foxp1* mRNA that includes either exon 16b or exon 16, while differentiated somatic cells include only exon 16 (Figure 1B) (Gabut et al., 2011). As expected, the exon 16b-exon 16 switch requires mRNA splicing activity, as treatment of mESCs with the splicing inhibitor Madrasin (Pawellek et al., 2014) promotes inclusion of exon 16b over exon 16 (Figure 1B). However, selective inhibition of SRPK with SRPKIN-1 in mESCs has little effect on exon 16b-exon 16 inclusion (Figure 1B), consistent with the minor impact of SRPK inhibition on SRSF splicing factor phosphorylation. In contrast, selective inhibition of CLK by CLK-IN-T3 phenocopies splicing inhibition and promotes exon 16b inclusion while suppressing inclusion of exon 16 (Figure 1B). These data indicate that SRPK activity is not required for a FOXP1 alternative splicing switch in mESCs, implying that SRPK may have acquired other developmental function(s) during metazoan evolution.

### Identification of SRPK Substrates and Functions in Embryonic Stem Cells

In order to shed light on further developmental functions of SRPKs, we sought to identify SRPK substrates. Previous studies have demonstrated that SRPKs directly phosphorylate Ser-Arg repeat (SR) motifs (Gui et al., 1994a, 1994b; Wang et al., 1998). Therefore, we interrogated the mouse proteome for characteristic SRPK consensus motifs of RSRS repeats separated by a linker of 0–20 residues using ScanProsite (https://prosite.expasy.org/scanprosite). A similar approach has been employed previously to identify a neural-specific splicing factor (Calarco et al., 2009). This analysis uncovered 77 predicted SRPK substrates, of which 48 have annotated splicing functions, while a smaller cohort of 29 is not known to participate in splicing regulation (Figure 1C; Tables S1 and S2). Interestingly, several have annotated developmental roles, including PAF1, which controls RNA PolII and stem cell pluripotency (Ding et al., 2009; Ponnusamy et al., 2009), and TJP2/ZO-2, a component of tight junctions. Also in this dataset is RNF12/RLIM, a RING-type E3 ubiquitin ligase (Figure 1C), which controls key developmental processes, including imprinted X-chromosome inactivation (Shin et al., 2014), and stem cell maintenance and differentiation (Bustos et al., 2018; Zhang et al., 2012). RNF12 variants cause an X-linked neurodevelopmental disorder termed as TOKAS (Frints et al., 2019; Hu et al., 2016; Tønne et al., 2015), which is underpinned by impaired RNF12 E3 ubiquitin ligase activity resulting in deregulated neuronal differentiation (Bustos et al., 2018). Thus, we hypothesized that SRPK phosphorylates and regulates RNF12, representing unappreciated functional diversification of SRPKs into developmental signaling.

### The RNF12 SR-Motifs Are Phosphorylated by SRPK and Other CMGC Family Kinases

Previous work has shown that RNF12 is phosphorylated at the SR-motifs (Jiao et al., 2013), although the kinase(s) have not been identified. In order to confirm that RNF12 SR-motifs are phosphorylated *in vivo*, we performed immunoprecipitation mass spectrometry. RNF12 phosphorylation was robustly detected at two conserved sites in mESCs—the SR-motifs encompassing Ser212/214/227/229 and an unstudied Ser163 site (Figure 1D; Table S3)—confirming that the SR-motifs are major sites of RNF12 phosphorylation.

The RNF12 SR-motifs consist of tandem RpSRpSP sequences (Figure 1D) flanking a nuclear localization signal (NLS), which resemble sequences phosphorylated by SRPKs and several other CMGC kinase sub-families. Absolute quantitative proteomics shows that many CMGC family kinases, including SRPKs, are expressed in mESCs (Figures 1E and S1A). Thus, we employed a representative CMGC kinase panel to identify kinases that directly phosphorylate RNF12 *in vitro*. GSK-3β, CDK2, CDK9, and DYRK1A readily phosphorylate RNF12 at Ser214 within the SR-motifs (Figure 1F), while SRPK1 or the closely related kinase CLK2 give a higher level of RNF12 Ser214 phosphorylation (Figure 1F). The ERK subfamily of CMGC kinases, including ERK2, JNK, and p38, do not appreciably phosphorylate RNF12 at Ser214 (Figure 1F). These data identify SRPK and closely related kinases as strong candidates for catalyzing RNF12 SR-motif phosphorylation.

### A Covalent SRPK Inhibitor Ablates RNF12 SR-Motif Phosphorylation

In order to identify the kinase that phosphorylates the RNF12 SR-motifs *in vivo*, we assembled a panel of kinase inhibitors that selectively inhibit CMGC family members. Of 12 CMGC family kinase inhibitors, a selective covalent inhibitor of SRPKs, SRPKIN-1 (Hatcher et al., 2018), had the greatest impact on the RNF12 phospho-Ser214/total ratio in mESCs (Figure 1G). The CDK7/9 inhibitor flavopiridol and the CDK1 inhibitor RO-3306 also have some effect, while the pan-CLK inhibitor CLK-IN-T3 has little impact on RNF12 phospho-Ser214/total ratio (Figure 1G). Interestingly, the structurally unrelated SRPK1 inhibitor SPHINX31 (Batson et al., 2017) has a minor effect on RNF12 Ser214 phosphorylation (Figure 1G), which is explained by the observation that SRPKIN-1 is ∼10-fold and ∼300-fold more potent toward SRPK1 than SPHINX31 and another commonly used SRPK inhibitor, SRPIN-340 (Fukuhara et al., 2006), respectively (Figure S1B). Furthermore, only SRPKIN-1 potently inhibited SRPK2 (Figure S1B), which is the other major SRPK isoform expressed in mESCs (Figures 1E, S1A, and S1C). Indeed, SRPK1 and SRPK2 are potently inhibited by SRPKIN-1 *in vivo*, as measured by the ability of SRPK1 or SRPK2 immunoprecipitates to phosphorylate RNF12 (Figure 1H). Our data therefore propose SRPK1/2 as candidate RNF12 SR-motif kinases.

### Widespread, Selective RNF12 SR-Motif Phosphorylation by SRPK

**Figure 2:**
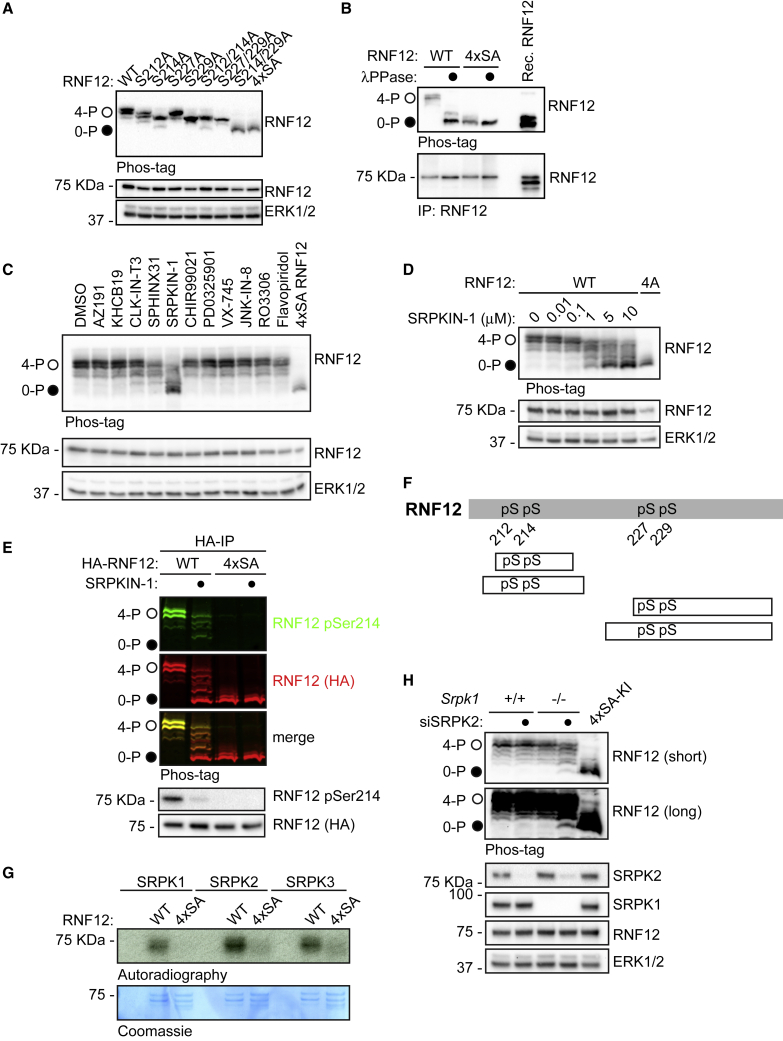
RNF12/RLIM E3 Ubiquitin Ligase Is Selectively Phosphorylated by SRPKs at a SR-Rich Motif (A) RNF12-deficient (*Rlim*^−^^/y^) mESCs were transfected with WT RNF12 or the indicated point mutants and RNF12 SR-motif phosphorylation analyzed by phos-tag immunoblotting for RNF12. Fully phosphorylated (4-P) and unphosphorylated (0-P) RNF12 SR-motifs are indicated by open (○) and closed (●) circles, respectively. RNF12 4xSA = S212A/S214A/S227A/S229A. (B) *Rlim*^−^^/y^ mESCs were transfected with the indicated RNF12 constructs and lysates treated with λ-phosphatase and analyzed by phos-tag immunoblotting for RNF12. Unphosphorylated recombinant RNF12 is included as a control. (C) mESCs were treated with 10 μM of the following kinase inhibitors: AZ-191 (DYRK1B), KH-CB19 (CLK-DYRK), CLK-IN-T3 (CLK), SPHINX31 (SRPK1), SRPKIN-1 (pan-SRPK), CHIR-99021 (GSK-3), PD-0325901 (MEK1/2), VX-745 (p38), JNK-IN-8 (JNK), RO-3306 (CDK1), and flavopiridol (CDK7/9) for 4 h and RNF12 SR-motif phosphorylation analyzed by phos-tag immunoblotting for RNF12. RNF12 4xSA is included as an unphosphorylated control. (D) mESCs were treated with the indicated concentrations of SRPKIN-1 for 4 h and RNF12 SR-motif phosphorylation analyzed by phos-tag immunoblotting for RNF12. (E) mESCs were treated with 10 μM SRPKIN-1 for 4 h and RNF12 phosphorylation analyzed from HA-RNF12 immunoprecipitates via RNF12 phos-tag and phospho-Ser214 immunoblotting using multiplex infrared immunoblot. (F) Phosphorylated peptides detected by mass spectrometry following *in vitro* phosphorylation of RNF12 by SRPK1. pS, phospho-serine. (G) Autoradiography of RNF12 WT or S212A/S214A/S227A/S229A (4xSA) following a radioactive kinase reaction with SRPK1, SRPK2, or SRPK3. RNF12 protein is detected by Coomassie staining. (H) Srpk1^+/+^ and Srpk1^−/−^ mESCs were transfected with control or SRPK2 siRNA and RNF12 SR-motif phosphorylation analyzed by phos-tag immunoblotting for RNF12. SRPK2, SRPK1, RNF12, and ERK1/2 levels were determined by immunoblotting. Related to Figure S2; Tables S3 and S4.

Phosphoproteomic analysis suggests that RNF12 is phosphorylated at Ser212, Ser214, Ser227, and Ser229 within the SR-motifs (Jiao et al., 2013) (Figure 1D; Table S3). In order to globally assess phosphorylation of these sites, we devised a phos-tag approach, which retards the mobility of phosphorylated proteins on SDS-PAGE (Kinoshita et al., 2006). RNF12 is phosphorylated to high stoichiometry at all Ser residues within the SR-motif, as mutation of each increases RNF12 mobility (Figure 2A). Interestingly, mutation of Ser214 and Ser229 disrupts RNF12 phosphorylation to a similar extent as mutation of all four sites (4xSA; Figure 2A), suggesting that RNF12 SR-motifs undergo hierarchical phosphorylation with C- to N-terminal processivity characteristic of SRPK substrates (Ma et al., 2008; Ngo et al., 2008). Importantly, an RNF12 4xSA mutant displays phos-tag mobility similar to that of dephosphorylated RNF12 (Figure 2B).

In order to determine whether SRPKs and/or other kinases phosphorylate further sites within the RNF12 SR-motifs, we again screened our CMGC kinase inhibitor panel in combination with RNF12 phos-tag analysis. Of these, only SRPKIN-1 drove a major dephosphorylation of the RNF12 SR-motif (Figure 2C). In contrast, the SRPK1 selective inhibitor SPHINX31 and pan-CLK inhibitor CLK-IN-T3 showed a minor effect on RNF12 phosphorylation (Figure 2C), while the CDK7/9 inhibitor flavopiridol and the CDK1 inhibitor RO-3306, which also suppress the RNF12 phospho-Ser214/total ratio (Figure 1G), had little impact. SRPKIN-1 treatment led to RNF12 SR-motif de-phosphorylation at concentrations as low as 1 μM (Figure 2D) and within 1–2 h (Figure S2A). Furthermore, the high-mobility form of RNF12 was completely dephosphorylated at Ser214 upon SRPKIN-1 treatment (Figure 2E), indicating that SRPKs mediate widespread RNF12 SR-motif phosphorylation. In support of this notion, mass spectrometry indicated that SRPKs directly phosphorylate all four Ser residues within the RNF12 SR-motif *in vitro* (Figure 2F; Table S4). Furthermore, SRPK is highly selective for the RNF12 SR-motif, phosphorylating wild-type RNF12 but not a mutant in which the SR-motif is mutated (4xSA; Figure 2G). In summary, our data uncovered a major role for SRPKs in phosphorylating the RNF12 SR-motif.

### Further Evidence that SRPK1/2 Are RNF12 SR-Motif Kinases

In order to confirm that SRPK1/2 activity is responsible for RNF12 SR-motif phosphorylation, we first determined SRPKIN-1 kinase inhibition specificity. Consistent with previous kinase interaction data (Hatcher et al., 2018), SRPKIN-1 is highly specific for SRPK1 inhibition compared with 49 other kinases (Figure S2B). Furthermore, inhibitors of major SRPKIN-1 off-target kinases, including CHK2, PLK1, and DYRK1A, did not impact RNF12 SR-motif phosphorylation *in vivo* (Figure S2C). In addition, RNF12 SR-motif phosphorylation was inhibited by SRPKIN-1 in washout assays, where SRPKIN-1 remained covalently bound to SRPKs but off-target kinases were removed (Hatcher et al., 2018) (Figure S2D).

To further substantiate the role of SRPK1/2 in RNF12 SR-motif phosphorylation, we sought to generate Srpk1^−/−^:Srpk2^−/−^ mESC lines using CRISPR-Cas9. Although we were able to obtain Srpk1^−/−^ and Srpk2^−/−^ mESC lines (Figure S2E), no Srpk1^−/−^:Srpk2^−/−^ mESC lines were recovered, suggesting that SRPK1/2 perform redundant functions in mESCs. Accordingly, RNF12 SR-motif phosphorylation was unaffected in Srpk1^−/−^ and Srpk2^−/−^ mESCs (Figure S2E). We therefore sought to deplete SRPK2 in Srpk1^−/−^ mESCs using siRNA. Partial depletion of SRPK2 expression in the absence of SRPK1 led to the appearance of a fraction of completely dephosphorylated RNF12 (Figure 2H), providing further evidence that SRPK1/2 phosphorylates the RNF12 SR-motif in mESCs. However, as several closely related CMGC family kinases, including CLK and DYRK, are expressed (Figure 1E) and able to phosphorylate RNF12 at Ser214 *in vitro* (Figure 1F), these kinases may also contribute to RNF12 SR-motif phosphorylation *in vivo*.

### RNF12 SR-Motif Phosphorylation Drives Nuclear Anchoring

We then explored functions of the SRPK1/2-RNF12 pathway using RNF12 SR-motif knockin (KI) mutant mESCs. Employing CRISPR-Cas9, we engineered RNF12 4xSA-KI mESCs, which cannot be phosphorylated at the SR-motifs, and RNF12 ΔSR-KI mESCs, in which residues 206–229 of the SR-motif are deleted. We also engineered control RNF12 wild-type (WT)-KI mESCs and catalytically inactive RNF12 W576Y-KI mESCs. All mutants are expressed at similar levels and have a similar half-life (Figure S3A), but RNF12 4xSA is not phosphorylated at the SR-motifs (Figure S3B).

**Figure 3:**
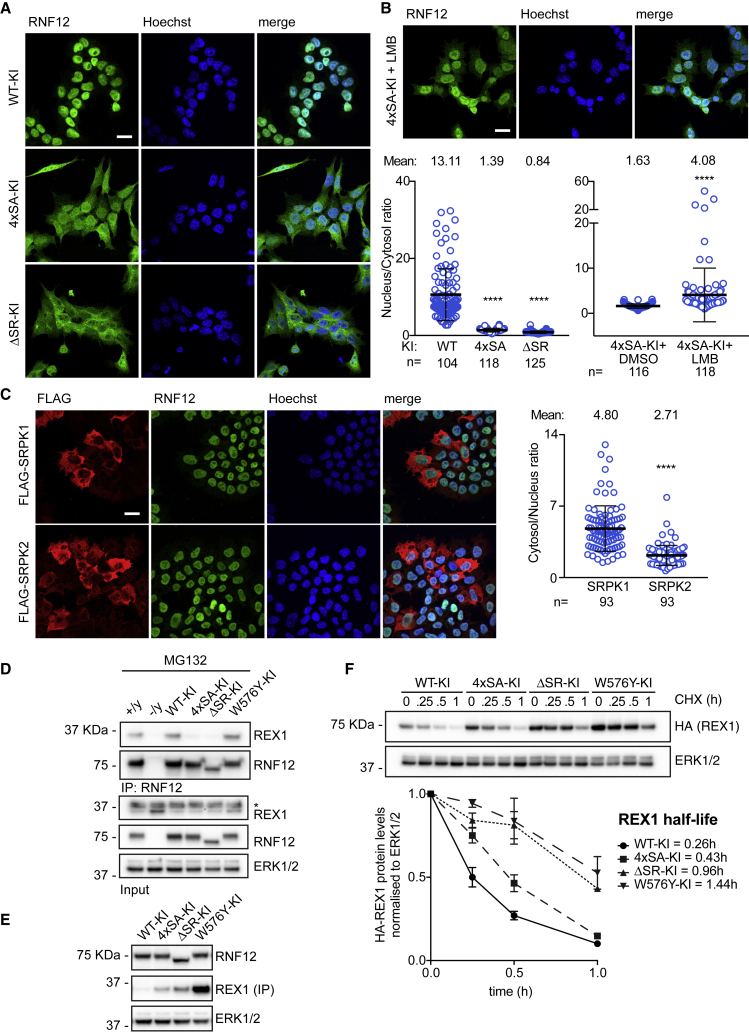
SRPK Phosphorylation of RNF12 Regulates Nuclear Anchoring and E3 Ubiquitin Ligase Activity (A) RNF12 localization in wild-type knockin (WT-KI), SR-motif phosphorylation site knockin (4xSA-KI), or SR-motif deletion (ΔSR-KI) mESCs was determined by immunofluorescence. Scalebar: 20 μm (Left). Quantification of the Nucleus/cytosol fluorescence intensity ratio (Right). Data represented as mean ± SEM. One-way ANOVA followed by Tukey’s multiple comparisons test; confidence level 95%. (^∗∗∗∗^) p < 0.0001. (B) RNF12 4xSA-KI mESCs were treated with 30 nM leptomycin B for 6 h and RNF12 localization analyzed by immunofluorescence. Scale bar: 20 μm (Top). Quantification of the nucleus/cytosol fluorescence intensity ratio (Bottom). Data represented as mean ± SEM Unpaired Student’s t test, two-sided, confidence level 95%. (^∗∗∗∗^) p < 0.0001. (C) FLAG-tagged SRPK1 and SRPK2 were expressed in mESCs and localization of SRPKs and RNF12 analyzed by immunofluorescence. Scale bar: 20 μm (Left). Quantification of the cytosol/nucleus fluorescence intensity ratio (Right). Data represented as mean ± SEM Unpaired Student’s t test, two-sided, confidence level 95%. (^∗∗∗∗^) p < 0.0001. (D) WT, *Rlim*^−^^/y^, RNF12 WT-KI, 4xSA-KI, ΔSR-KI, and W576Y-KI mESCs were treated with 10 μM MG132 for 6 h and RNF12-REX1 co-immunoprecipitation analyzed. RNF12, REX1 and ERK1/2 were detected by immunoblotting. (^∗^) indicates non-specific signal. (E) REX1 levels were analyzed in RNF12 WT-KI, 4xSA-KI, ΔSR-KI, and W576Y-KI mESCs by immunoprecipitation followed by immunoblotting. ERK1/2 levels were detected by immunoblotting. (F) REX1 half-life was determined in RNF12 WT-KI, 4xSA-KI, ΔSR-KI, and W576Y-KI mESCs by immunoblotting. (Top) quantification of HA-REX1 protein levels normalized to ERK1/2 and calculated protein half-life (Bottom). Data represented as mean ± SEM (n = 3). Related to Figure S3.

As RNF12 SR-motifs flank a NLS (Jiao et al., 2013), we used KI mutant mESC lines to investigate the role of SR-motif phosphorylation in subcellular localization. Wild-type RNF12 (RNF12 WT-KI) was localized entirely in the nucleus (Figure 3A), while RNF12 4xSA-KI and RNF12 ΔSR-KI showed significant staining in both the nucleus and cytosol (Figure 3A, nucleus/cytosol ratio: WT-KI = 13.11, 4xSA−KI = 1.39, ΔSR-KI = 0.84), indicating that RNF12-SR-motif phosphorylation promotes, but is not essential for, nuclear localization. In support of this, RNF12 4xSA was primarily nuclear in mESCs treated with the CRM nuclear export inhibitor leptomycin B (LMB) (Figure 3B, 4xSA-KI nucleus/cytosol ratio: Control = 1.63, LMB = 4.08). SRPK1 and SRPK2 were largely cytosolic, with some nuclear staining, particularly for SRPK2 (Figure 3C, cytosol/nucleus ratio: SRPK1 = 4.80, SRPK2 = 2.71), consistent with the notion that these kinases function outside the nucleus (Ding et al., 2006; Jang et al., 2009). Taken together, our data indicate that SRPK phosphorylation of the RNF12 SR-motif drives RNF12 nuclear anchoring but is not critical for nuclear translocation.

In light of these results, we tested whether RNF12 SR-motif phosphorylation is required for efficient degradation of nuclear substrates. A major RNF12 substrate is the REX1/ZFP42 transcription factor, which mediates RNF12 function in X-chromosome inactivation (Gontan et al., 2012, 2018). We first investigated the importance of RNF12 SR-motif phosphorylation for REX1 substrate engagement. RNF12-REX1 interaction was reduced in RNF12 4xSA-KI and RNF12 ΔSR-KI mESCs (Figure 3D), suggesting that SR-motif phosphorylation promotes RNF12 delivery to key nuclear substrates. Consistent with this notion, increased REX1 protein levels were observed in RNF12 4xSA-KI and RNF12 ΔSR-KI mESCs, to levels approaching that of catalytically inactive RNF12 W576Y-KI mESCs (Figure 3E). Furthermore, REX1 stability was increased in RNF12 4xSA KI, RNF12 ΔSR-KI, and RNF12 W576Y-KI mESCs, compared with RNF12 WT-KI control mESCs (Figure 3F). These data demonstrate that SRPK phosphorylation of RNF12 promotes REX1 targeting and degradation, and potentially that of other nuclear substrates.

### RNF12 SR-Motif Phosphorylation by SRPK Stimulates E3 Ubiquitin Ligase Activity

**Figure 4:**
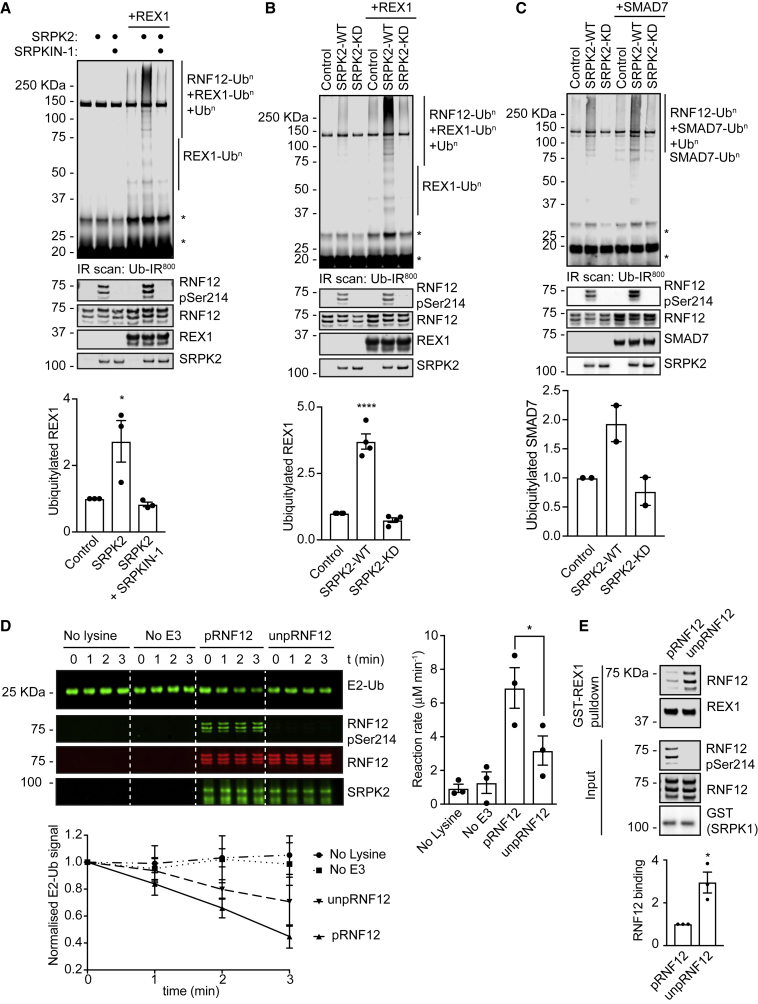
SRPK Phosphorylation Directly Stimulates RNF12 E3 Ubiquitin Ligase Activity (A) Recombinant RNF12 was incubated with SRPK2 ± 10 μM SRPKIN-1 and REX1 ubiquitylation assessed. Infrared scans of ubiquitylated substrate signal (Top) and quantification (Bottom). Data represented as mean ± SEM (n = 3). One-way ANOVA followed by Tukey’s multiple comparisons test; confidence level 95%. (^∗^) p = 0.0350. Phospho-Ser214 and total RNF12, REX1, and SRPK2 infrared immunoblots are shown. ^∗^ = non-specific fluorescent signal. (B) Recombinant RNF12 was incubated with WT or kinase dead (KD) SRPK2 and subjected to REX1 fluorescent ubiquitylation assays. Infrared scans of ubiquitylated substrate signal (Top) and quantification (Bottom). Data represented as mean ± SEM (n = 3). One-way ANOVA followed by Tukey’s multiple comparisons test; confidence level 95%. (^∗∗∗∗^) p < 0.0001. Phospho-Ser214 and total RNF12, REX1, and SRPK2 infrared immunoblots are shown. ^∗^ = non-specific fluorescent signal. (C) Recombinant RNF12 was incubated with WT or KD SRPK2 and SMAD7 ubiquitylation assessed. Infrared scans of ubiquitylated substrate signal (Top) and quantification (Bottom). Data represented as mean ± SD (n = 2). Phospho-Ser214 and total RNF12, REX1, and SRPK2 infrared immunoblots are shown. ^∗^ = non-specific fluorescent signal. (D) Recombinant RNF12 was incubated with WT (pRNF12) or KD (unpRNF12) SRPK2 and subjected to E2 ubiquitin discharge assay. Infrared immunoblot scans (Top Left), reaction rate determinations (Top Right) and normalized quantification of E2-ubiquitin conjugate signal (Bottom) are shown. Data represented as mean ± SEM (n = 3). One-way ANOVA followed by Tukey’s multiple comparisons test; confidence level 95%. (^∗^) p = 0.0490. (E) Recombinant RNF12 was incubated with WT or KD SRPK1 and subjected to GST-REX1 pull-down assay. RNF12, REX1, phospho-Ser214 RNF12 and SRPK1 infrared immunoblots (Top) and RNF12-REX1 binding quantification (bottom) are shown. Data represented as mean ± SEM (n = 3). Unpaired Student’s t test, two-sided, confidence level 95%. (^∗^) p = 0.0162. Related to Figure S4.

As RNF12 SR-motif phosphorylation impacts substrate degradation, we investigated whether SRPK-mediated SR-motif phosphorylation also regulates RNF12 catalytic activity. We used SRPK to phosphorylate the RNF12 SR-motifs to high stoichiometry *in vitro* (Figures S4A and S4B) and compared the E3 ubiquitin ligase activity of phosphorylated and non-phosphorylated RNF12. Strikingly, REX1 ubiquitylation detected by fluorescently labeled ubiquitin was enhanced following RNF12 phosphorylation by SRPK2 (Figures 4A and 4B), which is not observed upon pre-incubation with SRPKIN-1 (Figure 4A), or with catalytically inactive SRPK2 (Figure 4B). We also used a REX1 antibody to directly visualize mono-ubiquitylated REX1 (Figure S4C). Similar results were obtained with SRPK1 (Figures S4D and S4E) and ubiquitylation of SMAD7 (Figure 4C), another reported RNF12 substrate (Zhang et al., 2012). Taken together, these results suggest that SRPK phosphorylation stimulates RNF12 substrate ubiquitylation. However, the impact on RNF12 substrate poly-ubiquitylation has not yet been directly demonstrated.

We then sought to determine the mechanism by which RNF12 SR-motif phosphorylation stimulates catalytic activity. The SR-motif resides proximal to a basic region implicated in RNF12 substrate ubiquitylation (Bustos et al., 2018), and as such could potentially regulate RNF12 engagement with E2 ubiquitin conjugating enzyme or substrate. First, we investigated the impact of SR-motif phosphorylation on RNF12-dependent discharge of ubiquitin from a loaded E2 conjugating enzyme onto free lysine. At a concentration where unphosphorylated RNF12 poorly discharges ubiquitin from UBE2D1 E2 (Figure S4F), phosphorylation by SRPK2 augments E2 discharge activity (Figure 4D). Therefore, RNF12 SR-motif phosphorylation enhances substrate-independent ubiquitin discharge from E2 ubiquitin conjugating enzyme.

We also explored the direct impact of RNF12 SR-motif phosphorylation on substrate interaction. *In vivo*, RNF12 SR-motif phosphorylation promotes ubiquitylation activity and delivery to nuclear substrates, such as REX1 (Figure 3). In contrast, the interaction between RNF12 and REX1 *in vitro* is destabilized by RNF12 SR-motif phosphorylation by SRPK1 (Figure 4E) or SRPK2 (Figure S4G), confirming that phosphorylation does not stimulate catalytic activity via increased substrate affinity. Taken together, our data indicate that RNF12 SR-motif phosphorylation by SRPK promotes delivery to nuclear substrates and stimulates intrinsic E3 ubiquitin ligase activity.

### RNF12 E3 Ubiquitin Ligase Activity Controls a Neurodevelopmental Gene Expression Program

**Figure 5:**
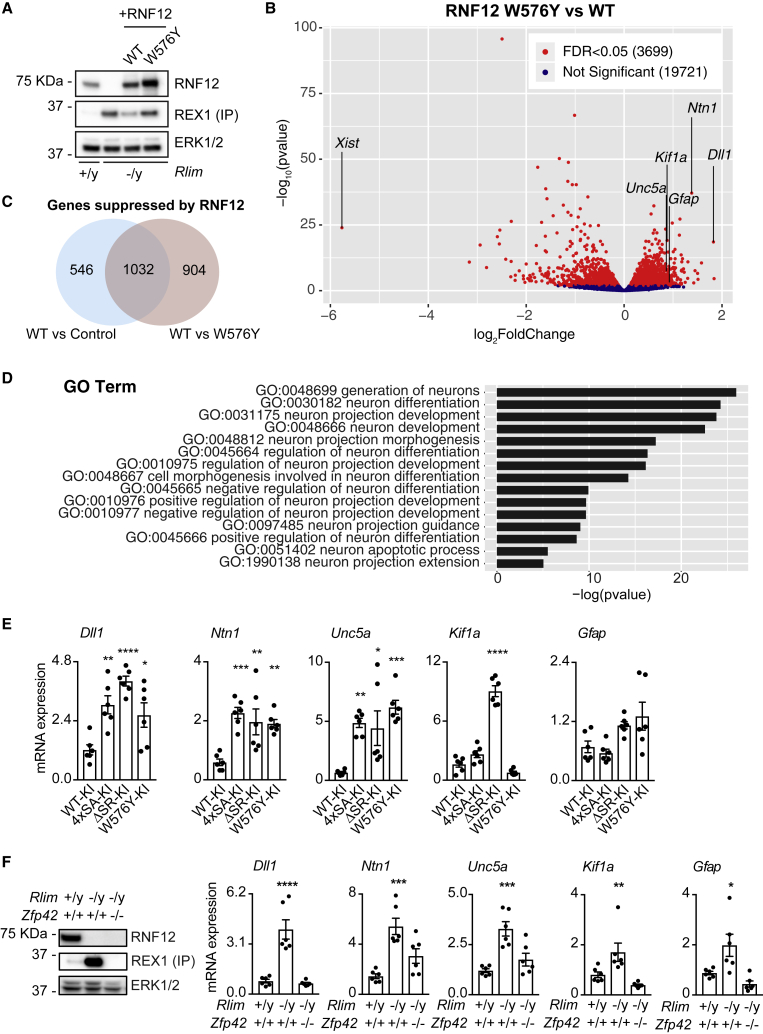
RNF12-REX1 Signaling Controls a Neurodevelopmental Gene Expression Program (A) *Rlim*^−^^/y^ mESCs were transfected with WT or catalytically inactive (W576Y) RNF12. REX1 levels were analyzed by immunoprecipitation and immunoblotting, RNF12 and ERK1/2 levels were determined by immunoblotting. (B) Volcano plot of RNA-seq analysis comparing *Rlim*^−^^/y^ mESCs transfected with WT or W576Y RNF12. RNAs that are significantly altered by RNF12 E3 ubiquitin ligase activity are displayed in red. Selected neurodevelopmental mRNAs are labeled (*Dll1*, *Ntn1*, *Unc5a*, *Kif1a*, *Gfap*). *Xist* is a positive control for RNF12 E3 ubiquitin ligase activity. FDR, false discovery rate. (C) Venn diagram displaying total number of RNAs negatively regulated by RNF12 catalytic activity. Intersection (1,032 genes) represents RNAs whose expression is significantly altered when comparing control versus WT RNF12, and WT RNF12 versus W576Y catalytic mutant. (D) GO category enrichment analysis of genes/RNAs related to the GO term “neuron” whose expression is inhibited by RNF12 (232 genes). (E) RNF12 WT-KI, 4xSA-KI, ΔSR-KI, and W576Y-KI mESCs were subjected to quantitative RT-PCR analysis of relative mRNA expression. Data represented as mean ± SEM (n = 3). One-way ANOVA followed by Tukey’s multiple comparisons test; confidence level 95%. *Dll1* (^∗∗^) p = 0.0058, (^∗∗∗∗^) p < 0.0001, (^∗^) p = 0.0377; *Ntn1* (^∗∗∗^) p = 0.0008, (^∗∗^) p = 0.0057, (^∗∗^) p = 0.0082; *Unc5a* (^∗∗^) p = 0.0079, (^∗^) p = 0.0188, (^∗∗∗^) p = 0.0006. *Kif1a* (^∗∗∗∗^) p < 0.0001. (F) RNF12, REX1, and ERK1/2 protein levels in WT, *Rlim*^−^^/y^ and *Rlim*^−^^/y^:Zfp42^−/−^ mESCs were determined by immunoblotting (RNF12 and ERK1/2) and immunoprecipitation followed by immunoblotting (REX1) (Left). WT, *Rlim*^-/y^ and *Rlim*^−^^/y^:Zfp42^−/−^ mESCs were analyzed for relative mRNA expression by quantitative RT-PCR (Right). Data represented as mean ± SEM (n = 3). One-way ANOVA followed by Tukey’s multiple comparisons test; confidence level 95%. *Dll1* (^∗∗∗∗^) p < 0.0001; *Ntn1* (^∗∗∗^) p = 0.0002; *Unc5a* (^∗∗∗^) p = 0.0003; *Kif1a* (^∗^) p = 0.0316; *Gfap* (^∗^) p = 0.0261, Related to Figure S5; Tables S5–S7.

As SRPK-dependent phosphorylation of the SR-motif activates RNF12 and anchors it in the nucleus to promote degradation of transcription factor substrates, such as REX1, we sought to identify the gene expression program that is regulated by this emergent signaling pathway. To this end, we employed RNF12-deficient (*Rlim*^−^^/y^) mESCs (Bustos et al., 2018) reconstituted with either wild-type RNF12 or an E3 ubiquitin ligase catalytic mutant (W576Y) and performed RNA sequencing (RNA-seq) to identify genes that are specifically regulated by RNF12. As validation of this experimental system, we show that REX1 degradation is restored by wild-type RNF12, but not RNF12 W576Y (Figure 5A). RNA-seq analysis reveals that RNF12 E3 ubiquitin ligase activity modulates expression of a significant cohort of RNAs (Figure 5B; 3,699 RNAs significantly altered, 19,721 RNAs not significantly altered). As proof of principle, the *Xist* long non-coding RNA, which has a key function in X-chromosome inactivation (Barakat et al., 2011), is regulated by RNF12 E3 ubiquitin ligase activity in the expected fashion (Figure 5B). Interestingly, additional comparison to control RNF12-deficient mESCs (Figure S5A) confirms that 1,032 RNAs are specifically suppressed by RNF12 in a manner dependent upon catalytic activity (Figure 5C).

In order to pinpoint functional groups of genes that are regulated by RNF12 E3 ubiquitin ligase activity, we employed Gene Ontology (GO) term analysis. Enriched within the cohort of RNF12-suppressed RNAs are those with GO terms associated with neuronal (Figure 5D; Table S5) and neural (Figure S5B; Table S6) development, differentiation, and function. This is consistent with a role for RNF12 in restricting mESC differentiation to neurons (Bustos et al., 2018). Genes assigned to neuron and/or neural GO terms are highlighted on a further plot of RNAs that are specifically regulated by RNF12 re-expression (Figure S5C). Interestingly, RNF12 suppresses expression of genes assigned to the “neural crest cell differentiation” GO term (GO: 0014033), which are linked to craniofacial abnormalities associated with neurodevelopmental syndromes (Table S7). In summary, we uncovered a neural and/or neuronal gene expression program that is suppressed by RNF12, providing a molecular framework for RNF12-dependent regulation of neurodevelopmental processes (Bustos et al., 2018).

### SRPK Signaling to RNF12 Regulates Neurodevelopmental Genes

These results prompted us to investigate the function of SRPK-RNF12 signaling in regulating expression of RNF12-responsive genes that have key functions in neural development. These are Delta-like 1 (*Dll1*), a regulator of Notch signaling in neural stem cells (Grandbarbe et al., 2003), Netrin-1 (*Ntn1*) and *Unc5a*, an axon guidance system essential for coordination of neuronal connections (Ackerman et al., 1997; Leonardo et al., 1997; Serafini et al., 1996), *Kif1a*, a motor protein for axonal transport (Okada and Hirokawa, 1999) and *Gfap*, an marker of astrocytes and radial glial cells (Middeldorp and Hol, 2011). Expression of each of these mRNAs, with the exception of *Unc5a*, increases during *in vitro* neural differentiation (Figure S5D), when the RNF12 SR-motif is phosphorylated (Figure S5E). Consistent with our RNA-seq data (Figure 5B), *Dll1*, *Ntn1*, *Unc5a*, and *Gfap* are expressed at low levels in control RNF12 WT-KI mESCs, and this was augmented in catalytically inactive RNF12 W576Y-KI mESCs (Figure 5E). *Kif1a* is expressed as at least 7 different splice isoforms in mouse, which likely explains conflicting results between RNA-seq and quantitative RT-PCR analysis. Nevertheless, our data confirm that RNF12-regulated neural genes are controlled by endogenous RNF12 E3 ubiquitin ligase activity in mESCs.

We next employed RNF12 KI mESC lines to determine the importance of SRPK signaling to RNF12 in regulation of neural gene expression. Compared with RNF12 WT-KI mESCs, neural gene expression is generally augmented by mutation of the SR-motif phosphorylation sites (RNF12 4xSA KI), deletion of the entire motif (RNF12 ΔSR-KI), or disruption of E3 ubiquitin ligase activity (RNF12 W576Y-KI; Figure 5E). Therefore, SRPK phosphorylation of RNF12 regulates key neural genes, implicating the SRPK-RNF12 pathway in the control of neurodevelopmental processes. As further evidence of the importance of the SR-motif for RNF12-dependent transcriptional regulation, induction of the known RNF12 target gene *Xist* was similarly disrupted by SR-motif mutation or deletion (Figure S5F).

### The SRPK-RNF12 Pathway Regulates Gene Expression by Promoting REX1 Degradation

As RNF12 SR-motif phosphorylation is required for efficient substrate ubiquitylation and target gene regulation, we sought to further define the molecular pathway. The REX1 transcription factor substrate plays a critical role in RNF12-dependent regulation of *Xist* gene expression and X-chromosome activation (Gontan et al., 2012, 2018). Thus, we hypothesized that REX1 ubiquitylation and degradation is the mechanism by which RNF12 modulates neural gene expression. We generated RNF12/REX1 double knockout mESCs (*Rlim*^−^^/y^:Zfp42^−/−^; Figure 5F) to investigate whether REX1 disruption reverses the gene expression changes observed in RNF12-deficient mESCs (*Rlim*^−^^/y^). Neural gene expression was augmented in RNF12-deficient mESCs, while additional knockout of REX1 (*Rlim*^−^^/y^:Zfp42^−/−^) reverses this gene expression profile (Figure 5F). These data illuminate REX1 as a key substrate that controls neurodevelopmental gene expression downstream of SRPK-RNF12 signaling.

### Human Intellectual Disability Mutations in the SRPK-RNF12 Pathway Lead to a Deregulated Neurodevelopmental Gene Expression Program

**Figure 6:**
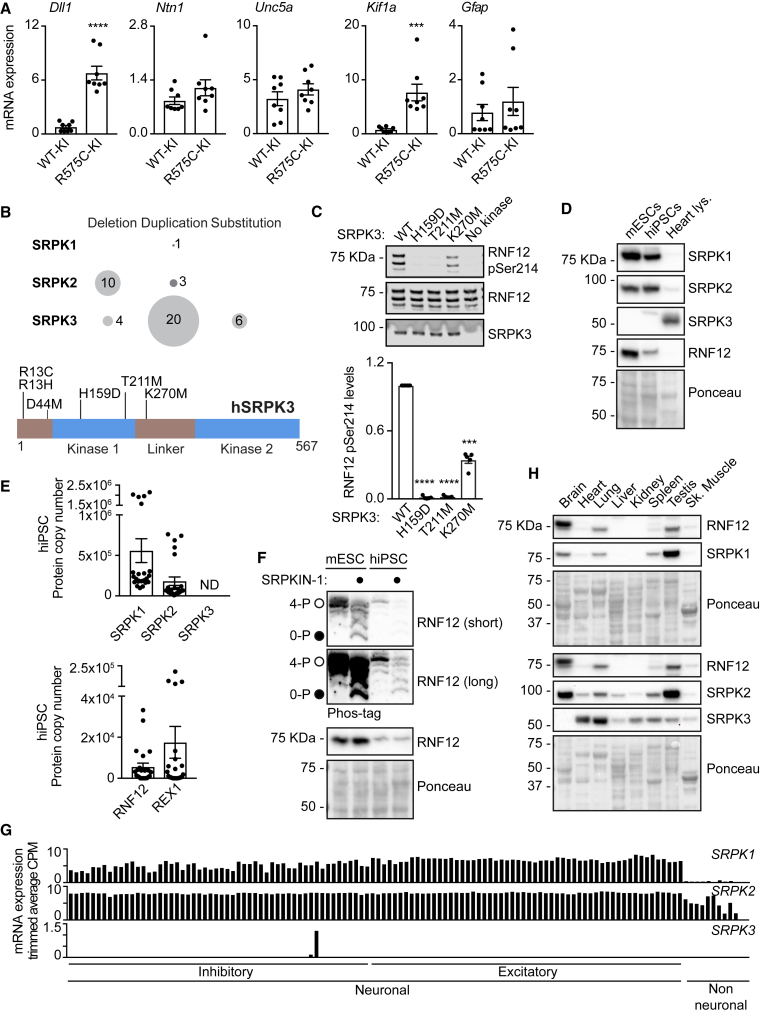
The SRPK-RNF12 Signaling Pathway Is Deregulated in Human Intellectual Disability (A) RNF12 WT-KI or R575C-KI mESCs were analyzed for relative mRNA expression by quantitative RT-PCR. Data represented as mean ± SEM (n = 3). Unpaired Student’s t test, two-sided, confidence level 95%. *Dll1* (^∗∗∗∗^) p < 0.0001; *Kif1a* (^∗∗∗^) p = 0.0005. (B) Graphical representation of SRPK intellectual disability variants reported in literature grouped by type of chromosomal mutation (Top) and position within the SRPK3 protein (Bottom). (C) RNF12 phosphorylation *in vitro* by WT SRPK3 or the indicated mutants was analyzed by immunoblotting for RNF12 phospho-Ser214 and total RNF12 (Top). Quantification of infrared RNF12 phospho-Ser214 immunoblotting blotting signal normalized to total RNF12 (Bottom). Data represented as mean ± SEM (n = 3). One-way ANOVA followed by Tukey’s multiple comparisons test; confidence level 95%. (^∗∗∗∗^) p > 0.0001, (^∗∗∗^) p = 0.0001. (D) SRPK1, SRPK2, SRPK3, and RNF12 levels in mESCs, hiPSCs (CHiPS4 cell line), and mouse heart lysate were analyzed by immunoblotting. (E) hiPSC (bubh_3 line) extracts were analyzed for average protein copy number via quantitative proteomics. Data were obtained from the human induced pluripotent stem cell initiative database (http://www.hipsci.org/) and represented as mean ± SEM (n = 24), ND, not detected. (F) hiPSCs (CHiPS4 cell line) and mESCs were treated with 10 μM SRPKIN-1 for 4 h and RNF12 SR-motif phosphorylation analyzed by phos-tag immunoblotting for RNF12. Fully phosphorylated (4-P) and unphosphorylated (0-P) RNF12 SR-motif is indicated with open (○) and closed (●) circles, respectively. (G) Single nuclei isolated from post-mortem human brain cortex neurosurgery were analyzed via SMART-seq v4 RNA-seq (data from Hodge et al., 2019). Each bar represents a distinct neuronal sub-type or non-neuronal cell. Trimmed average counts per million (CPM) for *SRPK1*, *SRPK2*, and *SRPK3* are shown. (H) Expression of RNF12, SRPK1, SRPK2, and SRPK3 in adult mouse tissues was analyzed by immunoblotting. Ponceau S staining is shown as a loading control.

Hereditable variants in RNF12 cause a neurodevelopmental disorder termed as TOKAS, which is a syndromic form of X-linked intellectual disability (Frints et al., 2019; Hu et al., 2016; Tønne et al., 2015). We showed previously that TOKAS mutations specifically impair RNF12 E3 ubiquitin ligase activity leading to deregulated neuronal differentiation (Bustos et al., 2018). In order to determine whether aberrant SRPK-RNF12 dependent neurodevelopmental gene expression might be relevant for TOKAS etiology, we examined expression of neural genes in mESCs harboring an RNF12 TOKAS patient mutation (mouse R575C—equivalent to human R599C) (Bustos et al., 2018). Expressions of *Dll1* and *Kif1a* were significantly increased in TOKAS mutant mESCs, with *Ntn1*, *Unc5a*, and *Gfap* also showing a tendency toward increased expression (Figure 6A). Thus, RNF12 TOKAS mutation partially phenocopies RNF12 SR-motif mutation with respect to the regulation of neurodevelopmental genes (Figure 5E).

As the SRPK-RNF12 signaling axis is disrupted in intellectual disability, we hypothesized that SRPK variants might cause related developmental syndromes. We mined molecular genetic databases of gene variants found in developmental disorders (Deciphering Developmental Disorders Study, 2015, 2017; Hu et al., 2016; Niranjan et al., 2015). A number of SRPK mutations have been identified in patients with intellectual disabilities or similar developmental abnormalities (Figure 6B, top). Of those, *SRPK2* is mainly deleted, suggesting that loss of SRPK2 expression may be a feature of these disorders. A number of duplications of the X-linked *SRPK3* gene were identified (Figure 6B), which is likely explained by frequent X-chromosome duplications in developmental disorders. Interestingly, several point mutations within the SRPK3 kinase domain (Figure 6B, bottom) have been reported in X-linked intellectual disability (Hu et al., 2016). We tested the effect of these mutations on the ability of SRPK3 to phosphorylate RNF12. SRPK3, H159D, and T211M mutations strongly impaired SRPK3 phosphorylation of RNF12, while K270M disrupted RNF12 phosphorylation to a lesser extent (Figure 6C). Thus, variants found in intellectual disability patients impair the ability of SRPK to phosphorylate RNF12, suggesting that SRPK function may be disrupted in intellectual disability disorders.

These findings prompted us to investigate the expression and function of SRPK family members in human pluripotent stem cells and the brain. SRPK1, SRPK2, and RNF12 are expressed in human induced pluripotent stem cells (hiPSCs; Figure 6D), and quantitative total proteomic analysis confirmed the expression of these components and REX1 (Figure 6E). Furthermore, the pathway is active in human pluripotent cells, as treatment of hiPSCs with the SRPK inhibitor SRPKIN-1 promotes RNF12 SR-motif dephosphorylation (Figure 6F). Mining single nuclei RNA-seq sequencing data (Hodge et al., 2019) revealed that *SRPK1* and *SRPK2* are broadly expressed in human cortical neurons, while *SRPK3* is specifically expressed in two GABAergic inhibitory neuron populations (Figure 6G), which have been implicated in intellectual disability (Sgadò et al., 2011; Smith-Hicks, 2013). RNF12, SRPK1, and SRPK2 are also robustly expressed in the adult mouse brain (Figure 6H). Therefore, the SRPK-RNF12 pathway is expressed and active in human pluripotent stem cells, and the components are expressed in adult human cortical neurons and mouse brain. Taken together, our data suggest that SRPK-RNF12 signaling is conserved during mouse and human neuronal development.

### SRPK Phosphorylates the RNF12 SR-Motif in Neurons

**Figure 7:**
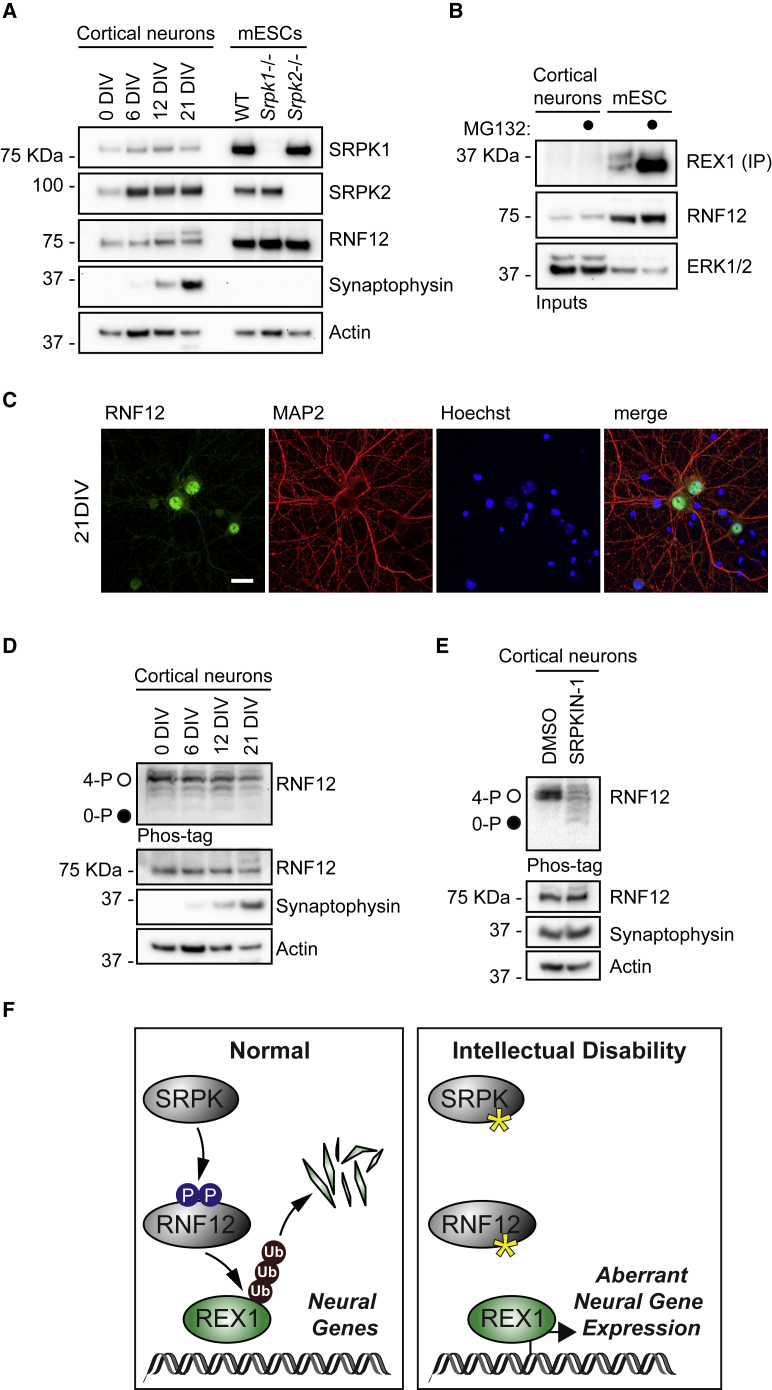
The SRPK-RNF12 Signaling Pathway Operates in Neurons (A) Primary cortical neurons isolated from E16.5 C57BL6 mice were cultured for the indicated number of days in vitro (DIV) and RNF12, SRPK1, and SRPK2, synaptophysin and actin levels analyzed via immunoblotting alongside the indicated mESC lines. (B) Cortical neurons were cultured for 21 days and treated with 10 μM MG132 and protein levels analyzed by immunoprecipitation and immunoblotting (REX1) and immunoblotting (RNF12 and ERK1/2). (C) Cortical neurons were cultured *in vitro* for the indicated number of days (DIV) and RNF12 and MAP2 neuron specific marker analyzed by immunofluorescence. Scale bar: 20 μm. (D) RNF12 SR-motif phosphorylation during *in vitro* mouse cortical neuron maturation was analyzed via phos-tag immunoblotting for RNF12. Fully phosphorylated (4-P) and unphosphorylated (0-P) RNF12 SR-motifs are indicated by open (○) and closed (●) circles, respectively. Synaptophysin and actin levels were determined by immunoblotting. (E) Cortical neurons were cultured for 21 days and treated with 10 μM SRPKIN-1 for 4 h RNF12 SR-motif phosphorylation was analyzed by phos-tag immunoblotting for RNF12. Fully phosphorylated (4-P) and unphosphorylated (0-P) RNF12 SR-motif is indicated with open (○) by closed (●) circles, respectively. Synaptophysin and actin levels were determined by immunoblotting. (F) The SRPK-RNF12-REX1 signaling pathway regulates neural gene expression and is disrupted in intellectual disability disorders. SRPK phosphorylates the RNF12 SR-motif to promote REX1 ubiquitylation and proteasomal degradation, which acts as a “brake” for neural gene expression in self-renewing pluripotent stem cells. In intellectual disability, inactivating mutations in SRPKs or RNF12 lead to REX1 accumulation and aberrant induction of neural genes.

Finally, we investigated the function of the SRPK-RNF12 pathway in neurons. Consistent with gene expression data from human cortical neurons (Figure 6G) and adult mouse brain (Figure 6H), RNF12, SRPK1, and SRPK2 were robustly expressed during maturation of isolated mouse fetal cortical neural progenitors *in vitro* (Figures 7A and S6). In contrast, SRPK3 (Figure S6) and REX1 (Figure 7B) were not detected in cultured mouse cortical neurons. RNF12 was predominantly localized to the nucleus in these neurons (Figure 7C), and phos-tag analysis indicated that the RNF12 SR-motif was heavily phosphorylated throughout a time course of neuronal maturation (Figure 7D). Furthermore, treatment of mature mouse cortical neurons with the selective SRPK inhibitor SRPKIN-1 suppressed RNF12 phosphorylation, as measured by phos-tag (Figure 7E). These data confirm that SRPKs phosphorylate the RNF12 SR-motif during neuronal maturation *in vitro*, suggesting that SRPK activity regulates RNF12 function in the nervous system.

## Discussion

Functional diversification of protein kinases is a key evolutionary tool, employing pre-existing signaling cassettes for regulation of complex cellular processes. However, the importance of functional diversification in the regulation of multi-cellularity remains unclear. Here, we show that SRSF protein kinase (SRPK), a highly conserved kinase family implicated in mRNA splicing, has undergone functional diversification to control developmental ubiquitin signaling. In mammalian embryonic stem cells, we found that SRPK activity is not required for splicing regulation. Instead, SRPK phosphorylates the E3 ubiquitin ligase RNF12/RLIM to control neurodevelopmental gene expression (Figure 7F). This function may have initially evolved to enable coordinated control of core cellular processes, such as RNA splicing, with key developmental events in multicellular organisms.

Our studies reveal that RNF12 SR-motif phosphorylation by SRPK drives delivery to nuclear substrates and increases substrate-independent ubiquitin discharge by a cognate E2-conjugating enzyme, indicating that phosphorylation of these motifs is required for maximal catalytic activity. Although RNF12 SR-motifs are distal to the catalytic RING domain, previous work confirms that distal non-RING regulatory elements play important roles in RNF12 catalysis (Bustos et al., 2018; Frints et al., 2019). Indeed, phosphorylation of distal non-RING elements in another RING E3 c-CBL mediates enzymatic activation (Dou et al., 2013). Structural investigations of full-length RNF12 in complex with cognate E2, ubiquitin, and substrate will be required to determine how phosphorylation drives enzymatic activation at the atomic level.

Our findings propose a critical role for SRPK in regulating developmental processes, although functional redundancy within the mammalian SRPK family has precluded genetic interrogation of SRPK functions during development. Nevertheless, a functional genomic screening indicated that SRPK2 is required for efficient X-chromosome inactivation (Chan et al., 2011), which is a key developmental function of RNF12. Furthermore, a recent study showed that SRPK1 initiates zygotic genome activation by phosphorylating protamine (Gou et al., 2020). Therefore, emerging evidence provides support for the notion that SRPKs perform key developmental functions.

SRPK signaling to RNF12 may ensure correct regulation of neural development. SRPK2 is highly expressed in the brain (Wang et al., 1998) and regulates processes relevant to neurodegeneration (Hong et al., 2012; Wang et al., 2017), suggesting a role for SRPK in development and maintenance of the nervous system. Additionally, we demonstrate that SRPK3 is expressed in sub-sets of human GABAergic neurons. Therefore, we propose that SRPK2 deletion or SRPK3 mutation may disrupt RNF12 function during development or maintenance of specific neuronal populations, leading to intellectual disability. A systematic analysis of SRPK and RNF12 expression during nervous system development is now required to identify specific cell populations in which SRPK-RNF12 signaling is relevant and potentially disrupted in intellectual disability.

Regulation of SRPK in a developmental context also remains unexplored. Previous work suggests that SRPKs are constitutively activated (Ngo et al., 2007), with additional regulatory inputs from the AKT-mTOR pathway (Jang et al., 2009; Lee et al., 2017). Diverse temporal and tissue-specific SRPK expression patterns also suggest that transcriptional regulation may be a key mechanism to ensure that SRPK phosphorylates substrates, such as RNF12, within the correct developmental time and space.

Finally, a key question relates to the function of RNF12 substrates in neuronal development. Our data indicate that RNF12 controls neural gene expression by ubiquitylating the REX1 transcription factor. The SRPK-RNF12 axis therefore appears to act as a safeguard to prevent aberrant REX1 accumulation and expression of neuronal genes in pluripotent stem cells. Although REX1 has not previously been implicated in the regulation of neuronal development and is undetectable in neurons, pathological REX1 accumulation upon RNF12 pathway mutation may unleash neomorphic transcriptional functions that are detrimental to neuronal development. This system could influence neuronal development by (1) transcriptional suppression of neural genes in non-neural cells, (2) modulating the timing and levels of neural gene expression in the developing neuroepithelium, or (3) acting to regulate a specific gene at the top of the neurogenesis signaling cascade. These findings suggest that approaches to activate SRPKs or normalize expression of REX1, for example using protein degradation technologies, such as proteolysis targeting chimeras (PROTACs), might provide therapeutic benefit in patients with neurodevelopmental disorders underpinned by deregulated SRPK-RNF12 signaling.

## STAR★Methods

### Key Resources Table

**Table:** 

REAGENT or RESOURCE	SOURCE	IDENTIFIER
**Antibodies**		

RNF12	Novus Biologicals	Cat#H00051132-M01; RRID: AB_547742
ERK1	BD Biosciences	Cat#610408; RRID: AB_397790
SRPK1	BD Biosciences	Cat#611072; RRID: AB_398385
SRPK2	BD Biosciences	Cat#611118; RRID: AB_398429
HA-tag	Abcam	Cat#ab9110; RRID: AB_307019
REX1	Abcam	Cat#ab28141; RRID: AB_882332
SRPK3	R&D Systems	Cat#MAB7230-SP
FLAG	Sigma Aldrich	Cat#F1804-50UG; RRID: AB_262044
HA-HRP	Roche	Cat#12013819001; RRID: AB_390917
Synaptophysin	Cell Signaling Technologies	Cat#5461 (D35E4); RRID: AB_10698743
beta-actin	Cell Signaling Technologies	Cat#4970 (13E5); RRID: AB_2223172
RNF12 (1-271)	MRC-PPU Reagents and Services	Cat#S691D third bleed
RNF12 pSer212/214 (QRRARpSRpSPEHRR)	MRC-PPU Reagents and Services	Cat#SA310 fourth bleed
GST	MRC-PPU Reagents and Services	Cat#S902A third bleed
Phosphoepitope SR proteins	Millipore	Cat#MABE50 clone 1H4; RRID: AB_10807429
KLF4	R&D Systems	Cat#AF3158; RRID: AB_2130245
MAP2	Sigma Aldrich	Cat#M2320; RRID: AB_609904
HA-tag	Sigma Aldrich	Cat#A2095 agarose beads; RRID: AB_257974

**Chemicals, Peptides, and Recombinant Proteins**		

AZ191	Tocris	Cat#5232
KH-CB19	Merck Millipore	Cat#219511
CLK-IN-T3	Aobious	Cat#AOB8827
SPHINX31	Axon Medchem	Cat#Axon 2714
CHIR99021	Axon Medchem	Cat#Axon 1386
PD0325901	Axon Medchem	Cat#Axon 1408
VX-745	Selleckchem	Cat#S1458
JNK-IN-8	Selleckchem	Cat#S4901
RO-3306	Sigma Aldrich	Cat#SML0569
Flavopiridol	Stratech	Cat#S2679
CCT241533	Cayman	Cat#CAY19178
Harmine	Sigma Aldrich	Cat#286044
WEHI-345	Cayman	Cat#CAY23023
IRAK-4 Ina	MRC-PPU Reagents and Services	N/A
GSK461364	Cayman	Cat#CAY18099
SRPIN340	Sigma Aldrich	Cat#SML1088
MG132	Sigma Aldrich	Cat#C2211
Cycloheximide	Sigma Aldrich	Cat#C7698
Leptomycin B	Sigma Aldrich	Cat#L2913
Madrasin (DDD00107587)	Kind gift of Dr. Andrea Pawellek (University of Dundee)	N/A
Phos-Tag	MRC-PPU Reagents and Services	N/A
RNF12	MRC-PPU Reagents and Services	DU61098
RNF12 S212A S214A S227A S229A	MRC-PPU Reagents and Services	DU53249
DYRK1a	MRC-PPU Reagents and Services	DU19040
CLK2	MRC-PPU Reagents and Services	DU16987
GSK3beta	MRC-PPU Reagents and Services	DU899
ERK1 (MAPK3)	MRC-PPU Reagents and Services	DU1509
ERK2 (MAPK1)	MRC-PPU Reagents and Services	DU650
JNK3 alpha 1 (SAPK1b)	MRC-PPU Reagents and Services	DU1511
p38 alpha (SAPK2a)	MRC-PPU Reagents and Services	DU979
CDK2 - CyclinA	MRC-PPU Reagents and Services	DU43557
CDK5 - p35	MRC-PPU Reagents and Services	DU39816
CDK7 - MAT1 - Cyclin H	MRC-PPU Reagents and Services	DU49574
CDK9 - Cyclin T1	MRC-PPU Reagents and Services	DU31050
REX1	MRC-PPU Reagents and Services	DU53244
SMAD7	MRC-PPU Reagents and Services	DU19219
SRPK1	MRC-PPU Reagents and Services	DU967
SRPK2	MRC-PPU Reagents and Services	DU36135
SRPK3	MRC-PPU Reagents and Services	DU967
SRPK1 D497A	MRC-PPU Reagents and Services	DU66208
SRPK2 D541A	MRC-PPU Reagents and Services	DU66209
SRPK3 H159D	MRC-PPU Reagents and Services	DU61121
SRPK3 T211M	MRC-PPU Reagents and Services	DU61140
SRPK3 K270M	MRC-PPU Reagents and Services	DU61135
Ube1	MRC-PPU Reagents and Services	DU32888
UBE2D1 (UbcH5a)	MRC-PPU Reagents and Services	DU4315
FLAG-Ubiquitin	MRC-PPU Reagents and Services	DU46789
Ubiquitin	MRC-PPU Reagents and Services	DU20027
Ubiquitin IR-800	Walden lab (Uni of Glasgow)	N/A

**Critical Commercial Assays**		

SRPKIN-1 kinase inhibitor profiling	This paper	http://www.kinase-screen.mrc.ac.uk/services/express-screen

**Deposited Data**		

Raw RNA-SEQ data	This paper	GEO: GSE149554
Raw Brain Cortex Single Nucleus RNA-SEQ data	Hodge et al., 2019	dbGAP: phs001790
Raw hiPSC mass-spectrometry data	Brenes et al., 2020	PRIDE: PXD010557

**Experimental Models: Cell Lines**		

Mouse: Embryonic Stem Cells	Laboratory of Janet Rossant, SickKids Research Institute, Toronto	CCE line
Human: induced Pluripotent Stem Cells	Cellartis AB Human Pluripotent Stem Cell Core Facility, School of Life Sciences, University of Dundee	CHiPS4 line
Human: Neuro2A	ATCC	Cat#CCL-131
Human: HEK 293	ATCC	Cat#CRL-1573
Human: U2OS	ATCC	Cat#HTB-96
Human: MCF7	ATCC	Cat#CRL-3435

**Experimental Models: Organisms/Strains**		

Mouse: C57B6/J	Animal Facility, School of Life Sciences, University of Dundee	N/A

**Oligonucleotides**		

Primers for qRT-PCR, see Table S8	This paper	N/A
Primers for genomic DNA sequencing, see Table S8	This paper	N/A
gRNA sequences for CRISPR/Cas9, see Table S8	This paper	N/A
ON-TARGETplus Srpk2 siRNA 06	Horizon Discovery	Cat#J-055142-06-0010
Non-targeting Pool siRNA	Horizon Discovery	Cat#D-001810-10-05

**Recombinant DNA**		

pCAGGS PURO RNF12	MRC-PPU Reagents and Services	DU50610
pCAGGS PURO RNF12 S212A	MRC-PPU Reagents and Services	DU53528
pCAGGS PURO RNF12 S214A	MRC-PPU Reagents and Services	DU50796
pCAGGS PURO RNF12 S227A	MRC-PPU Reagents and Services	DU53591
pCAGGS PURO RNF12 S229A	MRC-PPU Reagents and Services	DU53592
pCAGGS PURO RNF12 S212A S214A	MRC-PPU Reagents and Services	DU53518
pCAGGS PURO RNF12 S227A S229A	MRC-PPU Reagents and Services	DU53514
pCAGGS PURO RNF12 S214A S229A	MRC-PPU Reagents and Services	DU53593
pCAGGS PURO RNF12 S212A S214A S227A S229A	MRC-PPU Reagents and Services	DU50797
pCAGGS PURO RNF12 delta SR-motif	MRC-PPU Reagents and Services	DU53413
pCAGGS PURO HA-RNF12	MRC-PPU Reagents and Services	DU50854
pCAGGS PURO HA-RNF12 S212A S214A S227A S229A	MRC-PPU Reagents and Services	DU58741
pCAGGS PURO RNF12 W576Y	MRC-PPU Reagents and Services	DU50800
pCAGGS PURO FLAG SRPK1	MRC-PPU Reagents and Services	DU53820
pCAGGS PURO FLAG SRPK2	MRC-PPU Reagents and Services	DU53821
pKN7 RLIM ex5 KO Sense A	MRC-PPU Reagents and Services	DU52037
pX335 RLIM ex5 KO antisense A + Cas9n	MRC-PPU Reagents and Services	DU52046
pBabeD P U6 RLIM (mouse) Cter KI Sense A	MRC-PPU Reagents and Services	DU57881
pX335 RLIM (mouse) Cter KI AntiSense A	MRC-PPU Reagents and Services	DU57891
pMA RLIM Cter R575C IRES-GFP donor	MRC-PPU Reagents and Services	DU57963
pMA RLIM Cter del 206-229 IRES-GFP donor	MRC-PPU Reagents and Services	DU57964
pMA RLIM Cter S212A S214A S227A S229A IRES-GFP donor	MRC-PPU Reagents and Services	DU57966
pMA RLIM Cter wt control IRES-GFP donor	MRC-PPU Reagents and Services	DU57967
pMA RLIM Cter W576Y IRES-GFP donor	MRC-PPU Reagents and Services	DU60290
pBabeD P U6 Srpk1 (mouse) ex3 KO Sense B	MRC-PPU Reagents and Services	DU60949
pX335 Srpk1 (mouse) ex3 KO Antisense B	MRC-PPU Reagents and Services	DU64462
pBabeD P U6 SRPK2 (mouse) ex5 KO Sense A	MRC-PPU Reagents and Services	DU64247
pX335 SRPK2 (mouse) ex5 KO Antisense A	MRC-PPU Reagents and Services	DU 64251
pBabeD P U6 ZFP42 (mouse) ex4 KO Sense A	MRC-PPU Reagents and Services	DU60065
pX335 ZFP42 (mouse) ex4 KO AntiSense A	MRC-PPU Reagents and Services	DU60072

**Software and Algorithms**		

ScanProsite	Hulo et al., 2006	https://prosite.expasy.org/scanprosite/
Image Studio	LICOR Biosciences	https://www.licor.com/bio/image-studio/
Image Lab	Bio-Rad	https://www.bio-rad.com/en-uk/product/image-lab-software?ID=KRE6P5E8Z
Kinoviewer	Brenes and Lamond, 2019	https://peptracker.com
Proteome Discoverer v.2.0	Thermofisher	https://www.thermofisher.com/order/catalog/product/OPTON-30812?SID=srch-srp-OPTON-30812#/OPTON-30812?SID=srch-srp-OPTON-30812
Mascot	Matrix Science	https://www.matrixscience.com/server.html
Image J	NIH	https://imagej.nih.gov/ij/download.html
STAR software (v2.7.1a)	Dobin et al., 2013	https://github.com/alexdobin/STAR
HTSeq (v0.11.2)	Anders et al., 2015	https://htseq.readthedocs.io/en/master/
SARTools (v1.6.9)	Varet et al., 2016	https://github.com/PF2-pasteur-fr/SARTools
DESeq2 (v1.24)	Love et al., 2014	https://bioconductor.org/packages/release/bioc/html/DESeq2.html
GOstats (v2.50.0)	Falcon and Gentleman, 2007	https://www.bioconductor.org/packages/release/bioc/html/GOstats.html
GraphPad Prism (v7.0c)	GraphPad Software Inc.	https://www.graphpad.com/scientific-software/prism/

### Resource Availability

#### Lead Contact

Further information and requests for resources and reagents should be directed to and will be fulfilled by the Lead Contact, Greg Findlay (g.m.findlay@dundee.ac.uk).

#### Materials Availability

Plasmids and antibodies generated in this study have been deposited to MRC-PPU Reagents & Services (http://mrcppureagents.dundee.ac.uk/).

#### Data and Code Availability

The accession number for the RNA sequencing dataset generated during this study is Gene Expression Omnibus (GEO): GSE149554 (https://www.ncbi.nlm.nih.gov/geo/query/acc.cgi?acc=GSE149554).

Original source data have been deposited to Mendeley Data: https://doi.org/10.17632/phjvpdzp57.1

### Experimental Model and Subject Details

#### Cell Lines

##### Mouse Embryonic Stem Cells (mESCs)

Wild-type and CRISPR Cas9 edited male mESCs (CCE line) were cultured in 0.1% gelatin [w/v] coated plates in DMEM containing 10% foetal calf serum [v/v], 5% Knock-Out serum replacement [v/v], 2 mM glutamine, 0.1 mM MEM non-essential amino acids, 1mM sodium pyruvate, and penicillin/streptomycin (all from Thermo Fisher Scientific), 0.1 mM beta-mercaptoethanol (Sigma Aldrich), and 100 ng/ml GST-tagged Leukaemia inhibitory factor (LIF) at 5% CO_2_ and 37°C. For 2i culture, mESCs were converted from LIF/FBS to 2i culture media composed of N2B27: 1% B27 supplement [v/v], 0.5% N2 supplement [v/v], 2 mM glutamine (all from Thermo Fisher Scientific), 0.1 mM β-mercaptoethanol (Sigma Aldrich), and penicillin/streptomycin in 1:1 DMEM/F12:Neurobasal medium (both from Thermo Fisher Scientific with 1 μM PD0325901 and 1 μM CHIR99021) and neural differentiation induced by culturing cells in N2B27. Cells were routinely authenticated via morphology and pluripotency gene expression analysis.

##### Human Induced Pluripotent Stem Cells (hiPSCs)

hiPSCs (CHiPS4 male cell line) were cultured in feeder-free conditions in TeSR medium supplemented with Noggin (10 ng/ml, Peprotech) and bFGF (30 ng/ml, Peprotech) on plates coated with Geltrex matrix (20 μg/cm^2^, Life Technologies) at 5% CO_2_ and 37°C. Cells were routinely authenticated via morphology and pluripotency gene expression analysis

##### Other Mammalian Cell Lines

Male mouse Neuro 2a and female human U2OS, HEK 293 and MCF7 cell lines were grown in DMEM containing 10% foetal calf serum [v/v] at 5% CO_2_ and 37°C. Cells were routinely authenticated via morphology analysis.

#### Animal Studies

##### Primary Mouse Cortical Neurons

E16.5 C57BL/6 female and male mice brains were placed in ice cold HBSS, meninges removed, and cortex dissected. Cortex tissue was incubated with 0.125% trypsin containing DNAse at 37°C for 30 minutes. Samples were centrifuged at 1,200 rpm for 5 minutes and resuspended in complete Neurobasal media (Neurobasal containing 2 mM Glutamax, 2% B27 supplement [v/v], 10% foetal calf serum [v/v] and penicillin/streptomycin) and filtered through a 40 μm pore filter. Cells were then centrifuged for 7 minutes at 700 rpm, resuspended in complete Neurobasal media and plated at 0.5 x 10^6^ cells/well on 6-well plates coated with 0.1 mg/ml poly-L-lysine (PLL; Sigma Aldrich). Neurons were cultured at 37°C in a humidified incubator with 5% CO_2_ and medium replaced every 5 days with fresh medium containing B27.

##### Mouse Organs

19-week-old male C57BL/6J mice were dissected, organs collected and wrapped in tinfoil and snap frozen in liquid nitrogen. Organs were then resuspended in lysis buffer and lysed using a Polytrone PT 1200 E homogeniser (Kinematica, Littau-Lucerne, Switzerland) on ice. Samples were then clarified for 20 min at 14,000 rpm at 4°C and subjected to immunoblot analysis.

##### Ethics

Mouse studies were approved by the University of Dundee ethical review committee, and further subjected to approved study plans by the Named Veterinary Surgeon and Compliance Officer (Dr. Ngaire Dennison) and performed under a UK Home Office project licence in accordance with the Animal Scientific Procedures Act (ASPA, 1986). Mice were housed in a SPF facility in temperature-controlled rooms at 21°C, with 45-65% relative humidity and 12-hour light/dark cycles. Mice had *ad libitum* access to food and water and regularly monitored by the School of Life Science Animal Unit Staff.

### Method Details

#### Serine-Arginine Motif Search

Proteins containing tandem Serine-Arginine motifs were identified by searching the ScanProsite tool (Hulo et al., 2006) (ExPASy, Swiss Institute of Bioinformatics) for a R-S-R-S-x(0,20)-R-S-R-S motif where x is any amino acid and (0,20) the numerical range of intervening amino acids. The motif was searched against the UniProtKB database for *Mus musculus* proteome (TaxID: 10090). The resulting proteins were categorised according to UniProt functional description and listed in Table S1.

#### Plasmid and siRNA Transfection

mESCs were transfected with Lipofectamine LTX (Thermo Fisher Scientific) according to manufacturer instructions. All cDNA plasmids generated and used in this study are summarised in the Key Resource Table and can be found at MRC-PPU Reagents and services website http://mrcppureagents.dundee.ac.uk/. mESCs were transfected with siRNA using Lipofectamine RNAiMAX reagent (Thermo Fisher Scientific). siRNA oligos are listed in the Key Resource Table.

#### CRISPR/Cas9 Gene Editing

*Rlim*^−^^/y^ mESCs were described previously (Bustos et al., 2018). To generate CRISPR Cas9 knockout mESC lines wild-type (for Srpk1 and Srpk2) or *Rlim*^−^^/y^ (for Zfp42) mESCs were transfected with pX335 and pKN7 vectors containing gRNA sequences targeting *Srpk1* exon 3, *Srpk2* exon 5 or *Zfp42* exon 4 (detailed in Key Resource Table). *Rlim* WT-IRES-GFP (RNF12 WT-KI) and R575C-IRES-GFP (RNF12 R575C-KI) knock-in mESCs were described previously (Bustos et al., 2018). To generate *Rlim* S212A S214A S227A S229A-IRES-GFP (RNF12 4xSA-KI), *Rlim* with amino acids 206-229 deleted IRES-GFP (RNF12 ΔSR-KI) and *Rlim* W576Y-IRES-GFP (RNF W576Y-KI) knock-in mESC lines, wild-type mESCs were transfected with pBABED Puro U6 and pX335 vectors encoding guide RNAs targeting *Rlim* gene (detailed in Key Resource Table) together with donor pMa vectors containing DNA sequence encoding RNF12 amino acids 84 to 600 harbouring the desired mutations followed by an IRES (internal ribosome entry site) and EGFP. Transfected cells were selected with 3 μg/ml puromycin for 48 h and subjected to single cell sorting. Expanded knock-out single mESC clones were screened via immunoblot. EGFP positive knock-in single mESCs were expanded and screened for EGFP expression and RNF12 size or phosphorylation via immunoblot. Mutations were confirmed by genomic DNA sequencing. All cDNA plasmids are detailed in the Key Resource Table. Guide RNA and primer sequences are detailed in Table S8.

#### Pharmacological Inhibitors

All compounds were diluted in DMSO and mESCs treated with 10 μM inhibitor for 4 h prior lysis unless indicated otherwise. For protein stability assays, protein synthesis was inhibited by treating mESCs with 350 μM cycloheximide (Sigma Aldrich). For proteasome inhibition mESCs were treated with 10 μM MG132 (Sigma Aldrich) for 6 h. All chemicals are listed in the Key Resource Table.

#### Kinase Inhibitor Profiling

SRPKIN-1 inhibition activity was analysed using *in vitro* kinase assays for 50 representative kinases (MRC-PPU International Centre for Kinase Profiling). Kinase activity towards specific peptides was assessed in comparison to DMSO control. Full details are available at http://www.kinase-screen.mrc.ac.uk/services/express-screen.

#### Immunoblotting and Phos-Tag Analysis

SDS-PAGE electrophoresis and immunoblotting was performed using standard methods. Cells were lysed in lysis buffer (20 mM Tris [pH 7.4], 150 mM NaCl, 1 mM EDTA, 1% NP-40 [v/v], 0.5% sodium deoxycholate [w/v], 10 mM β-glycerophosphate, 10 mM sodium pyrophosphate, 1 mM NaF, 2 mM Na_3_VO_4_, and Roche Complete Protease Inhibitor Cocktail Tablets). Phospho-specific antibodies were used at 1 μg/ml with 10 μg/ml of the corresponding non-phosphopeptide. After secondary antibody incubation, membranes were subjected to chemiluminescence detection with Immobilon Western Chemiluminescent HRP Substrate (Millipore) using a Gel-Doc XR+ System (Bio-Rad) or Infrared detection using a LI-COR Odyssey Clx system. REX1 protein levels were determined by immunoblotting REX1 immunoprecipitates using Clean-Blot IP Detection Reagent (Thermo Fisher Scientific).

Phos-tag analyses were performed by loading protein samples containing 10 mM MnCl_2_ in 8% polyacrylamide gels containing 50 μM Phos-tag reagent (MRC-PPU reagents and services) and 0.1 mM MnCl_2_. After electrophoresis, gels were washed three times for 10 mins in Transfer buffer (48 mM Tris, 39 mM Glycine, 20% Methanol) supplemented with 20 mM EDTA. Proteins were then transferred to Nitrocellulose membranes, blocked and probed with the indicated antibodies. All protein signals were quantified using Image Studio (LI-COR Biosciences) or Image Lab software (Bio-Rad). Primary antibodies are listed in the Key Resource Table.

#### Mass Spectrometry

For phospho-site identification samples were separated via SDS-PAGE electrophoresis, stained with Coomassie blue and gel pieces subjected to an in-gel digestion. First, gel pieces were washed in water, 50% acetonitrile (ACN)/water, 0.1 M NH_4_HCO_3_ and 50% ACN/50 mM NH_4_HCO_3_ and then with 10 mM DTT/0.1 M NH_4_HCO_3_ (All from Sigma-Aldrich). Proteins were alkylated with 50 mM iodoacetamide/0.1 M NH_4_HCO_3_ and then washed as above. Gel pieces were then shrunk in ACN and dried using Speed-Vac. Proteins were then trypsinised by incubating with 5 μg/ml trypsin in 25 mM triethylammonium bicarbonate (Sigma-Aldrich) overnight. Supernatants were separated and gel pieces resuspended in 50% ACN/2.5% formic acid and supernatants combined. Samples were then dried via Speed-Vac and then resuspended in 30 μl 0.1 % formic acid and subjected to liquid chromatography–mass spectrometry (LC-MS) analysis using an Ultimate 3000 RSLCnano system coupled to LTQ-Orbitrap VelosPro mass spectrometer (ThermoFisher Scientific) 10 μl samples were injected and peptides were loaded onto a nanoViper C18 Trap column (5 μm particle size, 100 μm x 2 cm) and separated in a C18 reversed phase Easy-spray column (2 μm particle size, 75 μm x 50 cm) (ThermoFisher Scientific) at a flow rate of 300 nl/min. A linear gradient was used, starting at 3% B and maintained for 5 min, from 3-35% B in 40 min, 35-99% B for 2 min, maintained at 99% B for 5 min, 99-3% B in 3 min and maintained at 3% B for 5 min. Solvents used were A: 0.1% formic acid and B: 80% acetonitrile (ACN) with 0.08% formic acid.

Mass Spectrometry data was acquired in data-dependent mode using the following parameters: MS1 spectra were acquired in the Orbitrap at a resolution of 60,000 (at 400 m/z) for a mass range of 375-1600 m/z with a FTMS full AGC target of 1e6. The top 20 most intense ions (with a minimal signal threshold of 2000) were selected for MS2 analysis on the linear ion trap (with a full AGC target of 5,000) and were fragmented (using CID with a collision energy of 35%), multistage activation, and neutral loss masses of 24.4942, 32.6590, 48.9885.

Data was analysed using Proteome Discoverer v.2.0 and Mascot using MRC_Database_1 (1,950 sequences). Parameters used were the following: Variable modifications: Oxidation (M), Dioxidation (M), Phospho (STY); Fixed modifications: Carbamidomethyl (C), Enzyme: Trypsin/P, Maximum missed cleavages: 3, Precursor tolerance: 10ppm, MS2 tolerance: 0.6Da, Minimum score peptides: 18. Phospho-site assignment probability was estimated via Mascot and PhosphoRS3.1 (Proteome Discoverer v.1.4-SP1) or ptmRS (Proteome Discoverer v.2.0).

Quantitative total mESC proteomics data covering around 10,000 proteins was previously described (Fernandez-Alonso et al., 2017). CMGC kinase expression from that dataset was generated using Kinoviewer (https://peptracker.com) (Brenes and Lamond, 2019). Quantitative total proteomics data from human induced pluripotent stem cells (hiPSC, bubh_3 line) was obtained from the human induced pluripotent stem cell initiative (HipSci) database (Brenes et al., 2020).

#### Protein Expression and Purification

All recombinant proteins were produced in *E*. *coli* or SF21 insect cells expression systems by MRC-PPU reagents and services and purified via standard protocols. Proteins used in this study are listed in the Key Resource Table and can be found at the MRC-PPU Reagents and services website http://mrcppureagents.dundee.ac.uk/.

#### *In Vitro* Kinase Assays

For SRPK Immunoprecipitation kinase assays, mESCs were treated with 10 μM SRPKIN-1 for 4 h. Cells were lysed, and 1.5 mg of protein immunoprecipitated with 2 μg of SRPK1 or SRPK2 antibodies (BD Biosciences). Immunoprecipitates were then washed with lysis buffer supplemented with 500 mM NaCl and half of the sample was resuspended in loading buffer. The remainder was subjected to *in vitro* phosphorylation assay containing 0.5 μg RNF12 and 2 mM ATP in kinase buffer (50 mM Tris-HCl [pH 7.5], 0.1 mM EGTA, 10 mM MgCl2, 2 mM DTT) and incubated at 30°C for 30 min. SRPK *in vitro* kinase assays were performed by incubating 200 mU kinase or equivalent μg of inactive kinase with 0.5 μg RNF12 and 2 mM ATP in kinase buffer. For radioactive *in vitro* kinase assays, reactions were supplemented with 1 μCi γ-^32^P ATP. Reactions were incubated at 30°C for 30 min in presence or absence of inhibitor as indicated and samples subjected to polyacrylamide electrophoresis and immunoblot or Coomassie blue staining and signal detected via ECL, infrared detection or autoradiography.

#### Immunofluorescence

Immunofluorescence and confocal analysis were performed as described. mESCs were plated in 0.1% gelatin [v/v] coated coverslips. Cortical neurons were plated at a density of 1.5 x 10^5^ cells/well on poly-L-lysine German Glass Coverslips 18mm #1½ (EMSdiasum). Primary antibodies used are listed in the Key Resource Table. Cells were mounted using Fluorsave reagent (Millipore) Images were acquired in a Zeiss 710 confocal microscope and images were processed using Image J (NIH) and Photoshop CS5.1 software (Adobe). Nuclear and cytosolic staining intensity was determined using ImageJ (NIH).

#### *In Vitro* Phospho-RNF12 Activity Assays

For substrate ubiquitylation assays, 0.5 μg RNF12 protein was subjected to a phosphorylation reaction containing 200 mU SRPK or equivalent μg of catalytically inactive kinase and 2 mM ATP in kinase buffer for 1 h at 37°C. 200 nM phosphorylated RNF12 was then incubated with a ubiquitylation mix containing 1.5 μg of REX1 or SMAD7, 0.1 μM UBE1, 0.05 μM UBE2D1, 2 μM Ub-IR^800^, 0.5 mM TCEP [pH 7.5], 5 mM ATP (both from Sigma Aldrich), 50 mM Tris-HCl [pH 7.5], 5 mM MgCl_2_ for 30 min at 30°C. Reactions were stopped with SDS sample buffer and boiled for 5 min. Samples were loaded in 4-12% Bis-Tris gradient gels (Thermo Fisher Scientific). Gels were then scanned using an Odyssey CLx Infrared Imaging System (LICOR Biosciences) for detection of fluorescently labelled ubiquitylated proteins. After scanning proteins were transferred to PVDF or nitrocellulose membranes and analysed via immunoblot and signal detected using ECL or infrared detection.

For UBE2D1 ubiquitin discharge assays 5 μg RNF12 protein was phosphorylated as above with 2U SRPK or equivalent μg of catalytically inactive kinase and 2 mM ATP in kinase buffer for 1 h at 37°C. ATP was depleted with 4.5 U/ml apyrase (New England Biolabs) for 10 min at room temperature. UBE2D1-ubiquitin thioester was prepared by incubating 100 μM UBE2D1 with 0.2 μM UBE1, 100 μM FLAG-ubiquitin, 3 mM ATP, 0.5 mM TCEP [pH 7.5] (both from Sigma Aldrich), 5 mM MgCl2, 50 mM Tris (pH 7.5), 150 mM NaCl for 20 min at 37 °C. The reaction was stopped by depleting ATP with 4.5 U/ml apyrase (New England Biolabs) for 10 min at room temperature. Then, 40 μM UBE2D1-ubiquitin were incubated with 1 μM phosphorylated RNF12 and 150 mM L-lysine in a buffer containing 50 mM Tris [pH 7.5], 150 mM NaCl, 0.5 mM TCEP, 0.1% [v/v] NP40 at room temperature. Reactions were stopped with non-reducing SDS loading buffer and analysed via immunoblotting and membranes scanned in an Odyssey CLx Infrared Imaging System (LI-COR Biosciences). Protein signals were quantified using Image Studio software (LI-COR Biosciences). Reaction rates were determined by extrapolating protein signals in a standard curve of known concentrations of UBE2D1-ubiquitin conjugate and plotting concentration over time.

#### Binding Assays

For protein immunoprecipitation, protein A or G beads were incubated with 2 μg antibody and 0.5-2 μg/μl protein sample in lysis buffer overnight at 4°C. Immunoprecipitates were then washed three times with lysis buffer supplemented with 500 μM NaCl, resuspended in 50% [v/v] loading buffer and boiled at 95°C for 5 minutes prior to immunoblotting analysis. For HA tagged protein immunoprecipitation, Anti-HA agarose conjugate (Sigma Aldrich) was used.

For GST pulldown assays, 0.5 μg of RNF12 was phosphorylated with 200 mU SRPK (or 19 ng SRPK1 WT or KD; 60 ng SRPK2 WT or KD) in presence of 2 mM ATP in kinase buffer for 1 h at 37°C. ATP was depleted with 4.5 U/ml apyrase (New England Biolabs) for 10 min at room temperature and samples mixed with 0.5 μg REX1 protein in 500 μl GST pulldown buffer (10 mM Tris pH=8.0, 150 mM NaCl, 10% Glycerol, 0.1% Triton X-100, and Roche Complete Protease Inhibitor Cocktail Tablets) overnight at 4°C. Complexes were then pulled down using GSH Sepharose 4B beads (Sigma Aldrich) for 2 h at 4°C. Beads were then washed and samples analysed by immunoblotting.

#### RNA-Sequencing and Gene Ontology Analysis

Total RNA was extracted using RNeasy Mini Kit (QIAGEN) and DNA libraries prepared using TruSeq Stranded Total RNA Sample Preparation kits (Illumina) according to manufacturer’s instructions. Sequencing was performed on Illumina NextSeq platform. Briefly, raw sequencing reads were trimmed by removing Illumina adapters sequences and low-quality bases. Trimmed reads were mapped using to mouse reference genome (mm10) using STAR software (v2.7.1a) (Dobin et al., 2013). The number of reads per transcript was counted using HTSeq (v0.11.2) (Anders et al., 2015). The differentially expressed genes (DEGs) were estimated using SARTools (v1.6.9) (Varet et al., 2016) and DESeq2 (v1.24) (Love et al., 2014) R packages. Gene Ontology (GO) analysis was carried out using the GOstats (v2.50.0) R package (Falcon and Gentleman, 2007). Raw and processed data can be accessed at Gene Expression Omnibus, GEO: GSE149554 (https://www.ncbi.nlm.nih.gov/geo/query/acc.cgi?acc=GSE149554).

SMART-seq v4 RNA-Sequencing data from single nuclei within the human cortex was previously described (Hodge et al., 2019). Gene expression represented as trimmed average counts per million (average expression of the middle 50% of the data from log_2_ (CPM (exons+introns) per gene) was obtained from the Allen Brain Atlas (https://portal.brain-map.org/atlases-and-data/rnaseq).

#### RNA Extraction and Quantitative RT-PCR

Total RNA extraction and reverse transcription was performed as described. Quantitative PCR reactions using SsoFast EvaGreen Supermix (Bio-Rad) were performed in a CFX384 real time PCR system (Bio-Rad). Relative RNA expression was calculated through the ΔΔCt method and normalised to *Gapdh* expression. Data was analysed in Excel (Microsoft) and statistical analysis performed in GraphPad Prism v7.0c software (GraphPad Software Inc.). Primer sequences are listed in the Table S8.

### Quantification and Statistical Analysis

Data is presented as mean ± standard error of the mean (S.E.M) of at least three biological replicates unless otherwise indicated. Statistical significance was estimated using ANOVA followed by Tukey’s post hoc test or t-student’s test. Significance was defined as p<0.05. Statistical details for individual experiments can be found in the figure legends.

## Supporting Citations

The following references appear in the Supplemental Information: Bao and Jin, 2006, Byrd and Meyers, 2005, Chen et al., 2012, Chen et al., 2014, Coles et al., 2006, Dixon et al., 1994, Duprez et al., 1999, Fukumoto et al., 2006, Gammill et al., 2006, Gopinathan et al., 2019, Hargrave et al., 1997, Hill et al., 2014, Huang et al., 2016, Humphreys et al., 2012, Jia et al., 2016, Kamalakar et al., 2019, Li et al., 2009, Li et al., 2018, Micaglio et al., 2019, Nikopoulos et al., 2007, Parada et al., 2015, Pilia et al., 1999, Roosenboom et al., 2018, Sela-Donenfeld and Kalcheim, 1999, Shin et al., 2012, Sock et al., 2004, Teng et al., 2017, Toyofuku et al., 2008, Wang and Astrof, 2016, Wu and Taneyhill, 2012, Yen et al., 2010, York et al., 2018, Yu and Moens, 2005, Zhang et al., 2016, Zhu et al., 2019, Zuniga et al., 2010.

## Supplemental Information

Document S1. Figures S1–S6 and Tables S1–S8

Document S2. Article plus Supplemental Information
