# Supplementary material for: Functional Diversification of SRSF Protein Kinase to Control Ubiquitin-Dependent Neurodevelopmental Signaling
Source: Dev Cell. 2020 Dec 7;55(5):629–647.e7. doi: 10.1016/j.devcel.2020.09.025 (PMC7725506; doi:10.1016/j.devcel.2020.09.025)
Supplement: Document S1. Figures S1–S6 and Tables S1–S8 [file mmc1.pdf]

Developmental Cell, Volume *55*

Supplemental Information

Functional Diversification of SRSF Protein Kinase to Control Ubiquitin-Dependent Neurodevelopmental Signaling

Francisco Bustos, Anna Segarra-Fas, Gino Nardocci, Andrew Cassidy, Odetta Antico, Lindsay Davidson, Lennart Brandenburg, Thomas J. Macartney, Rachel Toth, C. James Hastie, Jennifer Moran, Robert Gourlay, Joby Varghese, Renata F. Soares, Martin Montecino, and Greg M. Findlay

Figure S1

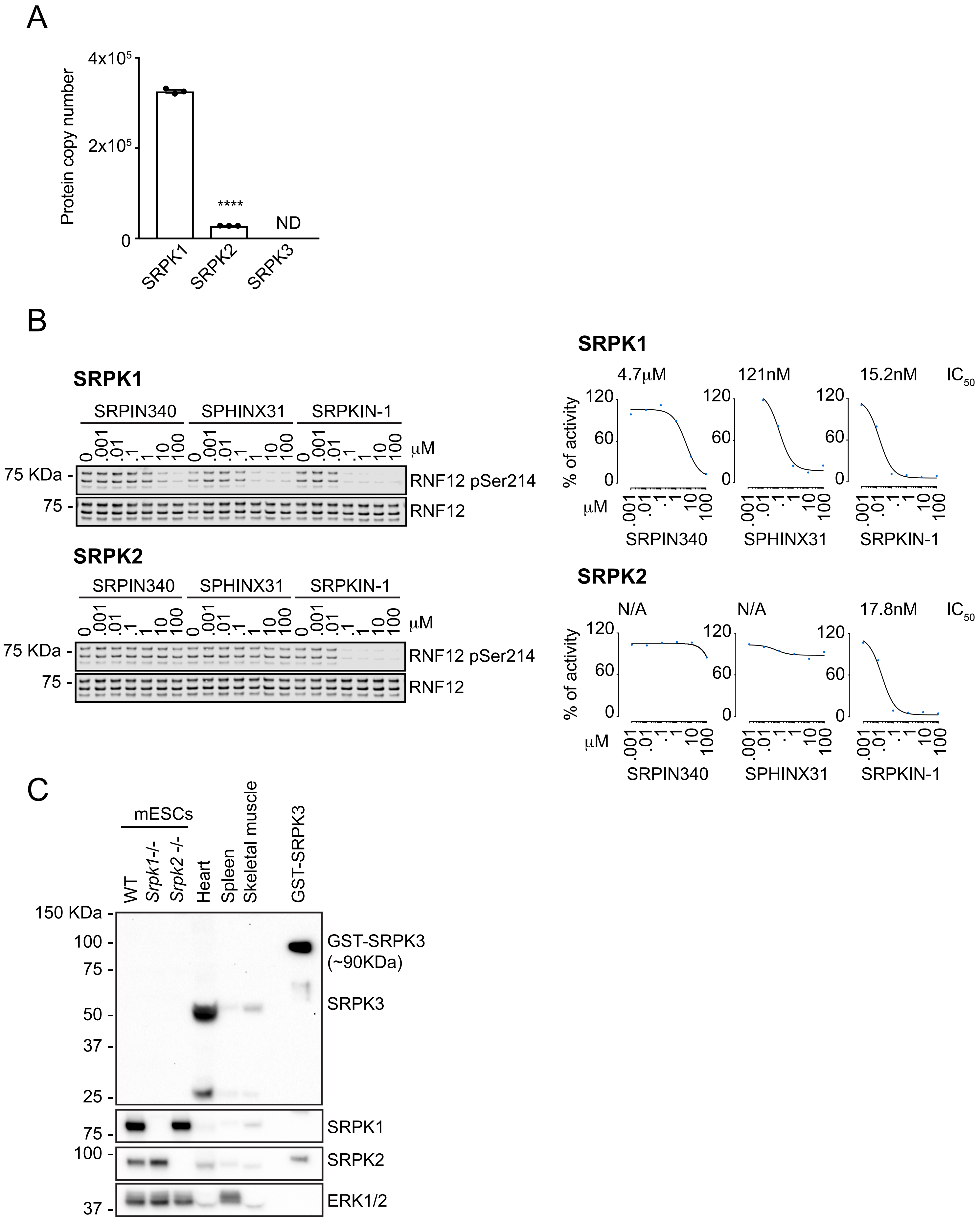

A
4x10^5
2x10^5
0
Protein copy number
****
ND
SRPK1
SRPK2
SRPK3
B
SRPK1
SRPIN340
SPHINX31
SRPKIN-1
0 .001 .01 .1 1 10 100
μM
75 KDa -
75 -
RNF12 pSer214
RNF12
SRPK2
4.7μM
121nM
15.2nM
IC50
N/A
17.8nM
120
60
0
% of activity
C
mESCs
WT
Srpk1-/-
Srpk2 -/-
Heart
Spleen
Skeletal muscle
GST-SRPK3
150 KDa -
100 -
75 -
50 -
37 -
25 -
GST-SRPK3
(~90KDa)
SRPK3
SRPK1
SRPK2
ERK1/2

Figure S2

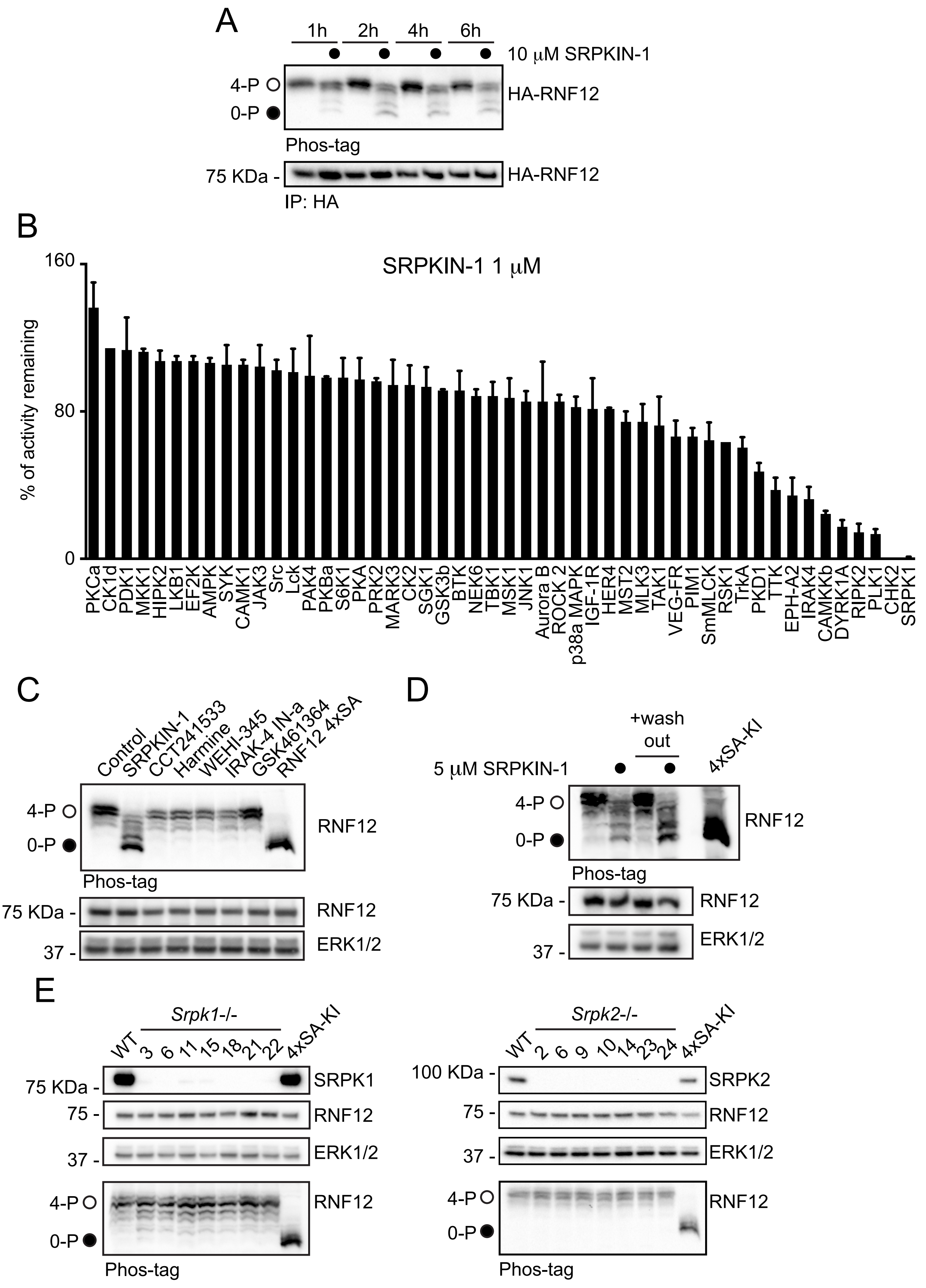

A
1h
2h
4h
6h
10 μM SRPKIN-1
4-P
0-P
HA-RNF12
Phos-tag
75 KDa -
HA-RNF12
IP: HA
B
SRPKIN-1 1 μM
% of activity remaining
160
80
0
PKCa
CK1d
PDK1
MKK1
HIPK2
LKB1
EF2K
AMPK
SYK
CAMK1
JAK3
Src
Lck
PAK4
PKBa
S6K1
PKA
PRK2
MARK3
CK2
SGK1
GSK3b
BTK
NEK6
TBK1
MSK1
JNK1
Aurora B
ROCK 2
p38a MAPK
IGF-1R
HER4
MST2
MLK3
TAK1
VEG-FR
PIM1
SmMLCK
RSK1
TrkA
PKD1
TTK
EPH-A2
IRAK4
CAMKKb
DYRK1A
RIPK2
PLK1
CHK2
SRPK1
C
Control
SRPKIN-1
CCT241533
Harmine
WEHI-345
IRAK-4 IN-a
GSK461364
RNF12 4xSA
4-P
0-P
RNF12
Phos-tag
75 KDa -
RNF12
37 -
ERK1/2
D
+wash out
5 μM SRPKIN-1
4xSA-KI
4-P
0-P
RNF12
Phos-tag
75 KDa -
RNF12
37 -
ERK1/2
E
Srpk1-/-
WT
3
6
11
15
18
21
22
4xSA-KI
75 KDa -
SRPK1
75 -
RNF12
37 -
ERK1/2
4-P
0-P
RNF12
Phos-tag
Srpk2-/-
WT
2
6
9
10
14
23
24
4xSA-KI
100 KDa -
SRPK2
75 -
RNF12
37 -
ERK1/2
4-P
0-P
RNF12
Phos-tag

Figure S3

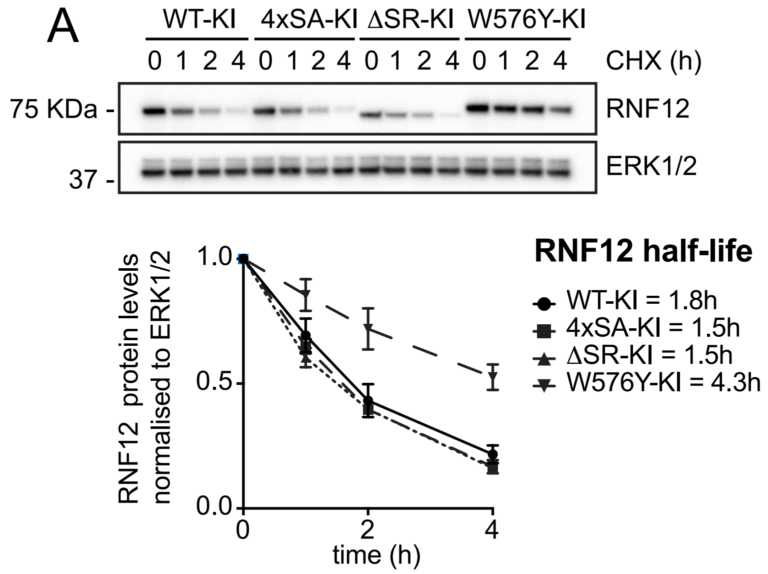

A
WT-KI
4xSA-KI
ΔSR-KI
W576Y-KI
0 1 2 4 0 1 2 4 0 1 2 4 0 1 2 4
CHX (h)
75 KDa -
RNF12
37 -
ERK1/2
RNF12 protein levels normalised to ERK1/2
1.0
0.5
0.0
0
2
4
time (h)
RNF12 half-life
WT-KI = 1.8h
4xSA-KI = 1.5h
ΔSR-KI = 1.5h
W576Y-KI = 4.3h

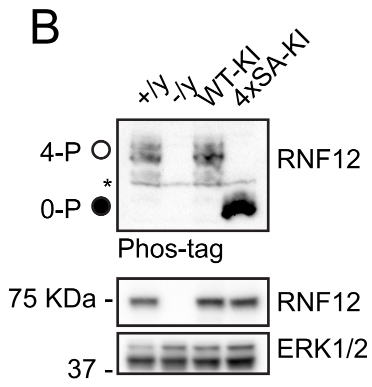

B
+/y
-/y
WT-KI
4xSA-KI
4-P
*
0-P
RNF12
Phos-tag
75 KDa -
RNF12
37 -
ERK1/2

Figure S4

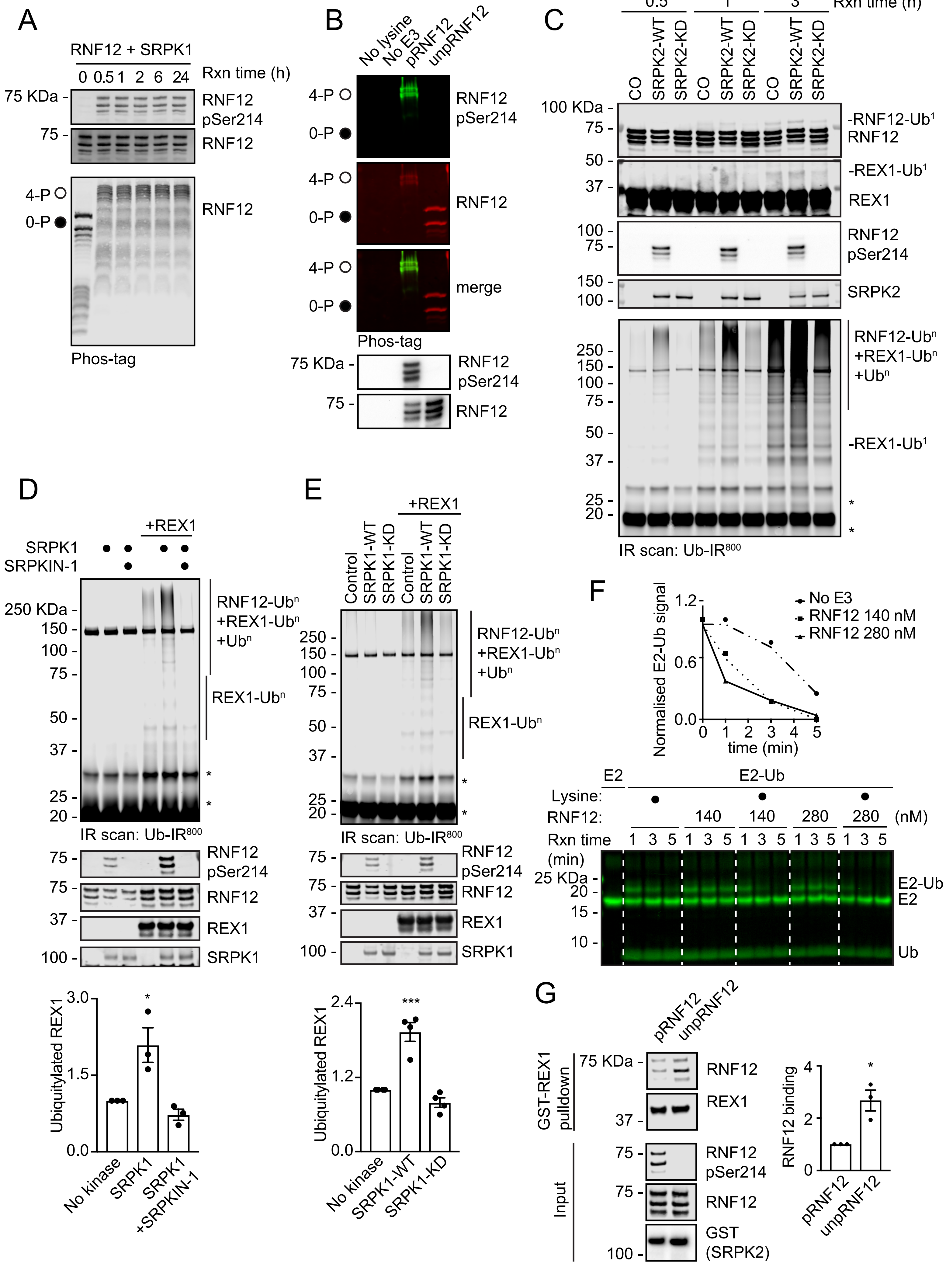
A
RNF12 + SRPK1
Rxn time (h)
0 0.5 1 2 6 24
75 KDa
RNF12 pSer214
75
RNF12
4-P
0-P
RNF12
Phos-tag
B
No lysine
No E3
pRNF12
unpRNF12
RNF12 pSer214
RNF12
merge
Phos-tag
75 KDa
RNF12 pSer214
75
RNF12
C
0.5 1 3 Rxn time (h)
CO SRPK2-WT SRPK2-KD
100 KDa
-RNF12-Ub1
RNF12
-REX1-Ub1
REX1
RNF12 pSer214
SRPK2
RNF12-Ubn +REX1-Ubn +Ubn
-REX1-Ub1
IR scan: Ub-IR800
D
+REX1
SRPK1
SRPKIN-1
250 KDa
RNF12-Ubn +REX1-Ubn +Ubn
REX1-Ubn
IR scan: Ub-IR800
RNF12 pSer214
RNF12
REX1
SRPK1
Ubiquitylated REX1
No kinase
SRPK1
SRPK1 +SRPKIN-1
E
+REX1
Control
SRPK1-WT
SRPK1-KD
RNF12-Ubn +REX1-Ubn +Ubn
REX1-Ubn
IR scan: Ub-IR800
RNF12 pSer214
RNF12
REX1
SRPK1
Ubiquitylated REX1
No kinase
SRPK1-WT
SRPK1-KD
F
Normalised E2-Ub signal
No E3
RNF12 140 nM
RNF12 280 nM
time (min)
E2
E2-Ub
Lysine:
RNF12:
140 140 280 280 (nM)
Rxn time (min)
1 3 5
25 KDa
E2-Ub
E2
Ub
G
pRNF12
unpRNF12
GST-REX1 pulldown
75 KDa
RNF12
REX1
Input
RNF12 pSer214
RNF12
GST (SRPK2)
RNF12 binding
pRNF12
unpRNF12

Figure S5

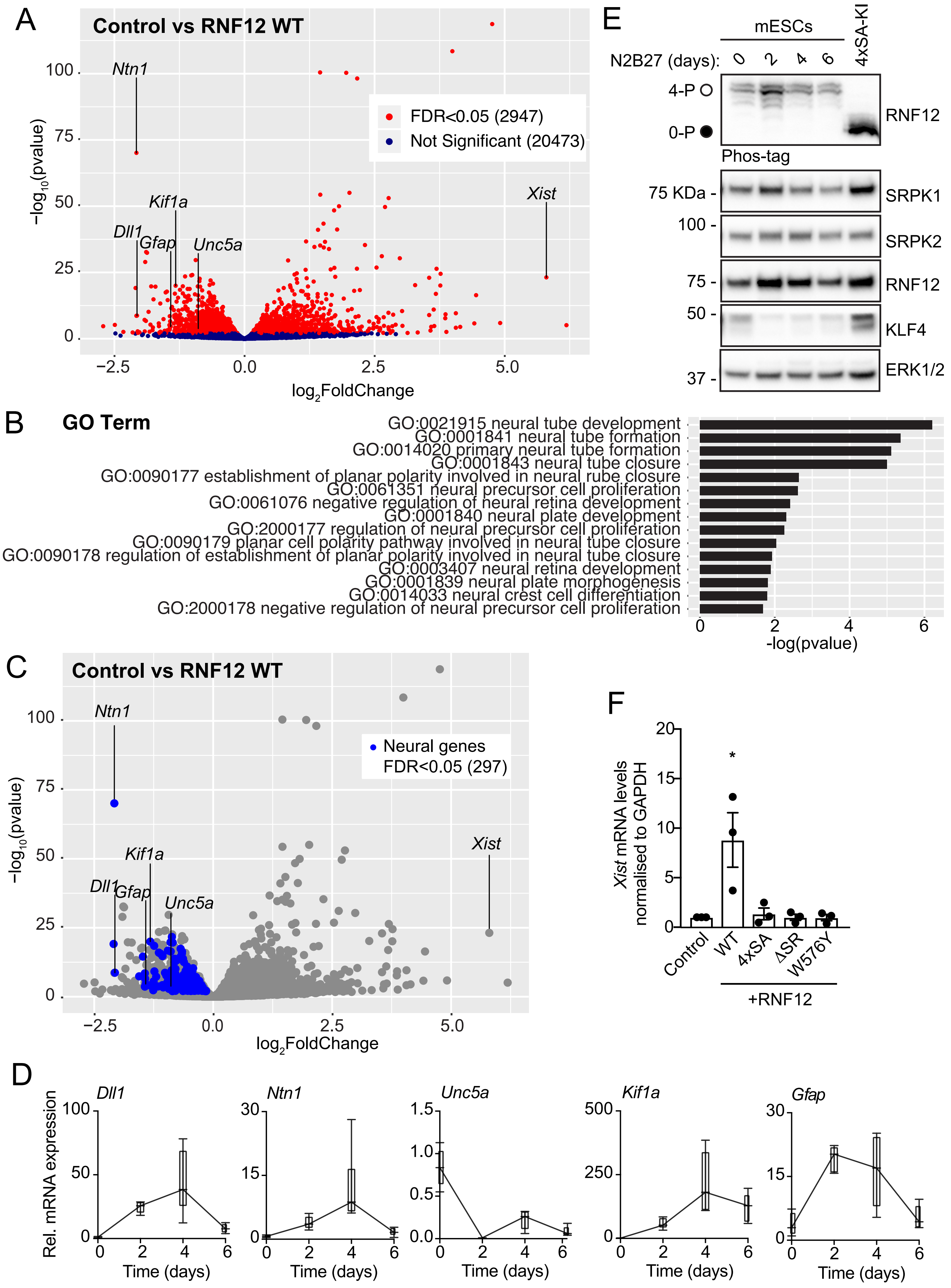

A
Control vs RNF12 WT
FDR<0.05 (2947)
Not Significant (20473)
Ntn1
Kif1a
Dll1
Gfap
Unc5a
Xist
−log10(pvalue)
log2FoldChange
E
mESCs
N2B27 (days): 0 2 4 6
4xSA-KI
4-P
0-P
Phos-tag
RNF12
75 KDa -
SRPK1
100 -
SRPK2
75 -
RNF12
50 -
KLF4
37 -
ERK1/2
B
GO Term
GO:0021915 neural tube development
GO:0001841 neural tube formation
GO:0014020 primary neural tube formation
GO:0001843 neural tube closure
GO:0090177 establishment of planar polarity involved in neural rube closure
GO:0061351 neural precursor cell proliferation
GO:0061076 negative regulation of neural retina development
GO:0001840 neural plate development
GO:2000177 regulation of neural precursor cell proliferation
GO:0090179 planar cell polarity pathway involved in neural tube closure
GO:0090178 regulation of establishment of planar polarity involved in neural tube closure
GO:0003407 neural retina development
GO:0001839 neural plate morphogenesis
GO:0014033 neural crest cell differentiation
GO:2000178 negative regulation of neural precursor cell proliferation
-log(pvalue)
C
Control vs RNF12 WT
Neural genes FDR<0.05 (297)
Ntn1
Kif1a
Dll1
Gfap
Unc5a
Xist
−log10(pvalue)
log2FoldChange
F
Xist mRNA levels normalised to GAPDH
Control
WT
4xSA
ΔSR
W576Y
+RNF12
D
Rel. mRNA expression
Dll1
Ntn1
Unc5a
Kif1a
Gfap
Time (days)

Figure S6

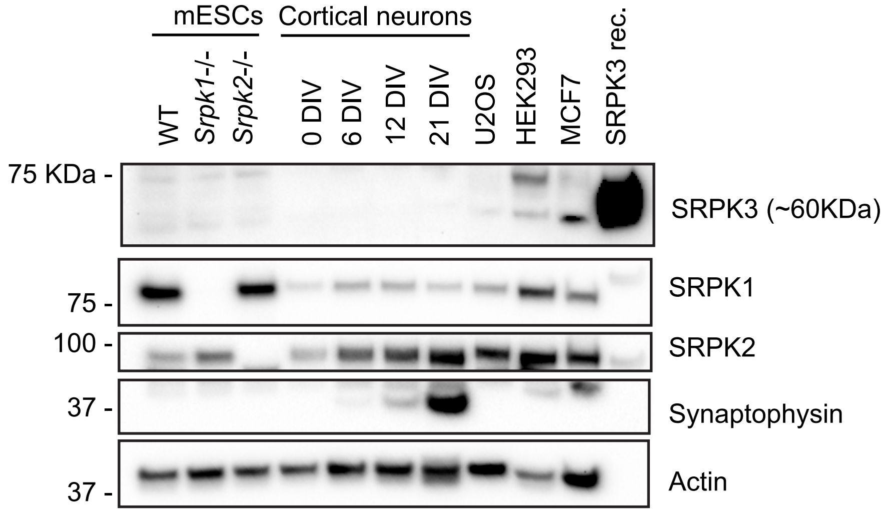
mESCs
Cortical neurons
WT
Srpk1-/-
Srpk2-/-
0 DIV
6 DIV
12 DIV
21 DIV
U2OS
HEK293
MCF7
SRPK3 rec.
75 KDa -
SRPK3 (~60KDa)
SRPK1
75 -
100 -
SRPK2
37 -
Synaptophysin
37 -
Actin

Supplemental Figure Legends

Figure S1. Differential inhibitor sensitivities and expression profiles of SRPK kinases (Related to Figure 1). (A) Wild-type (WT) mESCs were analysed for protein copy number by absolute quantitative proteomics. SRPK1, SRPK2 and SRPK3 protein copy numbers are shown. Data are represented as mean ± S.E.M. (n=3). Unpaired Student's t test, two-sided, confidence level 95%. (****) P<0.0001. ND = not detected. (B) Inhibition of RNF12 phosphorylation *in vitro* by SRPK1 and SRPK2 in the presence of varying concentrations of the indicated SRPK inhibitors was determined by immunoblotting for RNF12 phospho-Ser214 (Left). RNF12 levels are shown as a control. Immunoblots were quantified to generate SRPK inhibitor dose-response curves for inhibition of RNF12 phosphorylation by SRPK1 and SRPK2 *in vitro* (Right). (C) WT, *Srpk1*$^{-/-}$ and *Srpk2*$^{-/-}$ mESCs were cultured and SRPK protein expression was analysed via immunoblotting. Heart, spleen and skeletal muscle tissue lysates and SRPK3 recombinant protein are shown as positive controls for SRPK3 expression.

Figure S2. SRPK1/2 phosphorylate RNF12 SR-motif in mESCs (Related to Figure 2). (A) HA-RNF12 expressing *Rlim*$^{-/y}$ mESCs were treated with 10 µM SRPKIN-1 for the indicated times and SR-motif phosphorylation of HA-immunoprecipitated RNF12 analysed by phos-tag immunoblotting. HA-RNF12 levels are shown as a control. Fully phosphorylated (4-P) and unphosphorylated (0-P) RNF12 SR-motif is indicated by open (○) and closed (●) circles respectively. (B) SRPKIN-1 inhibition of 50 kinases was profiled *in vitro* (MRC-PPU International Centre for Kinase Profiling). Data are represented as mean ± S.D. (n=3). (C) RNF12 expressing mESCs were treated with 10 µM of the following inhibitors: SRPKIN-1 (SRPK inhibitor), CCT-241533 (CHK2 inhibitor), Harmine (DYRK1A inhibitor), WEHI-345 (RIPK2 inhibitor), IRAK-4-Inhibitor-a (IRAK4 inhibitor) and GSK-461364 (PLK1/2 inhibitor) for 4 h, and RNF12 phosphorylation analysed via phos-tag immunoblotting. HA-RNF12 and ERK1/2 levels are shown as a control. (D) RNF12 expressing mESCs were pre-treated with 5 µM SRPKIN-1 for 3 h, media changed and cells cultured for further 5 h (+ wash-out). RNF12 phosphorylation was analysed via phos-tag immunoblotting. ERK1/2 levels are shown as a loading control. (E) Multiple *Srpk1*$^{-/-}$ and *Srpk2*$^{-/-}$ mESC clones were

analysed for RNF12 phosphorylation via phos-tag immunoblotting. SRPK, RNF12 and ERK1/2 levels are shown as controls.

Figure S3. RNF12 protein stability is unaffected by SR-motif phosphorylation (Related to Figure 3). (A) The indicated RNF12 knock-in mESC lines were treated with 350 µM cycloheximide for the indicated times and analysed for RNF12 levels via immunoblotting. ERK1/2 levels are shown as loading control (Top). Quantification of RNF12 signal intensity and determination of protein half-life via immunoblotting and non-linear curve fitting (Bottom). Data are represented as mean ± S.E.M. (n=3) (B) Phos-tag immunoblot analysis of RNF12 SR-motif phosphorylation in the indicated mESC lines. RNF12 and ERK1/2 levels are shown as controls. Fully phosphorylated (4-P) and unphosphorylated (0-P) RNF12 SR-motif is indicated by open (○) and closed (●) circles respectively. (*) Indicates non-specific signal.

Figure S4. SRPK-mediated RNF12 SR-motif phosphorylation stimulates RNF12 E3 ubiquitin ligase activity (Related to Figure 4). (A) Time course of RNF12 phosphorylation by SRPK1 *in vitro,* analysed for SR-motif phosphorylation via RNF12 phospho-Ser214 infrared and phos-tag immunoblotting. Fully phosphorylated (4-P) and unphosphorylated (0-P) RNF12 SR-motif is indicated with open (○) and closed (●) circles respectively. RNF12 levels are shown as a control. (B) RNF12 phosphorylation by SRPK2 for 1 h *in vitro* was analysed via multiplex infrared Phos-tag and regular immunoblotting. This material is representative of samples used in E2 ubiquitin discharge assays displayed in Figure 4D. (C) Recombinant RNF12 was incubated with wild-type (WT) or kinase dead (KD) SRPK2 and subjected to REX1 ubiquitylation assays for the indicated reaction times. RNF12, REX1 RNF12 phospho-Ser214 and SRPK2 expression were determined by immunoblotting. Infrared scans of ubiquitylated substrate signal are shown. Monoubiquitylated RNF12 and REX1 signals are indicated as RNF12-Ub^1 and REX1-Ub^1 respectively. (D) Recombinant RNF12 was incubated with SRPK1 in absence or presence of SRPKIN-1 and subjected to REX1 fluorescent ubiquitylation assays. Infrared scans of ubiquitylated substrate signal, and phospho-Ser214 and total RNF12, REX1 and SRPK1 control infrared immunoblots are shown (Top) with graphical quantification (Bottom). Data are represented as mean ± S.E.M. (n=3).

One-way ANOVA followed by Tukey's multiple comparisons test; confidence level 95%. (*) P=0.0221 (n=3). (E) Recombinant RNF12 was incubated with WT or KD SRPK1 and subjected to REX1 fluorescent ubiquitylation assays. Infrared scans of ubiquitylated substrate signal, and phospho-Ser214 and total RNF12, REX1 and SRPK1 control infrared immunoblots are shown (Top) with graphical quantification (Bottom). Data are represented as mean ± S.E.M. (n=3). One-way ANOVA followed by Tukey's multiple comparisons test; confidence level 95%. (***) P=0.0002 (n=3). (F) The indicated concentration of recombinant RNF12 was assayed for UBE2D1 E2 ubiquitin discharge assay for the indicated reaction times. Normalised E2-ubiquitin conjugate signal quantification (Top) and infrared Coomassie gel staining scans (Bottom) are shown. (G) Recombinant RNF12 was incubated with WT or KD SRPK2 and then subjected to a GST-REX1 pulldown assay. Infrared immunoblots (Left) and RNF12-REX1 binding quantification are shown (Right). Data are represented as mean ± S.E.M. (n=3). Unpaired Student's t test, two-sided, confidence level 95%. (*) P= 0.0133.

Figure S5. RNF12 negatively regulates neurodevelopmental gene expression in mESCs (Related to Figure 5). (A) Volcano plot of RNA-SEQ comparing RNA expression of *Rlim*$^{-/y}$ mESCs transfected with control or WT RNF12. RNAs that are significantly altered by RNF12 are displayed in red (2947 genes). Key neurodevelopmental mRNAs that are inhibited by RNF12 E3 ubiquitin ligase activity are labelled (*Dll1, Ntn1, Gfap, Kif1a, Unc5a*). *Xist* is a known target of RNF12 activity. FDR = False discovery rate. (B) Gene Ontology analysis of RNF12 responsive genes identifies significant enrichment of genes related to neural development (65 genes). (C) Volcano plot of RNA-SEQ comparing RNA expression of *Rlim*$^{-/y}$ mESCs transfected with control or WT RNF12. Neuronal/neural genes negatively regulated by RNF12 identified via Gene Ontology are highlighted in blue (297 genes). (D) Selected neurodevelopmental mRNA expression was analysed by quantitative RT-PCR following mESC neural differentiation in N2B27 media for the indicated times. Data is represented in Box-and-whisker plots showing median, first and third quartiles, and maximum and minimum values (n=6). (E) mESCs were induced to undergo neural differentiation in N2B27 media for the indicated times, and RNF12 SR-motif phosphorylation was analysed by phos-tag immunoblotting. Fully phosphorylated (4-P) and unphosphorylated (0-P)

RNF12 SR-motif is indicated by open (○) and closed (●) circles respectively. SRPK1, SRPK2, RNF12, KLF4 and ERK1/2 levels were analysed by immunoblotting. KLF4 is shown as a pluripotency marker and ERK1/2 as a loading control. (F) mESCs were transfected with the indicated vectors and cultured for 72 h prior analysis of *Xist* RNA expression via quantitative RT-PCR. Data is represented as mean ± S.E.M. (n=3). One-way ANOVA followed by Tukey's multiple comparisons test; confidence level 95%. (*) P=0.0292.

Figure S6. SRPK1/2 are the major isoforms expressed in cultured mouse cortical neurons (Related to Figure 7). Primary cortical neurons isolated from E16.5 C57BL6 mice were cultured for the indicated number of days *in vitro* (DIV) and SRPK1, SRPK2, SRPK3, synaptophysin and actin expression analysed via immunoblotting alongside the indicated mESC lines. U2OS, HEK293, MCF7 and recombinant SRPK3 were used as positive controls for SRPK3 expression, synaptophysin as a neuronal maturation marker and actin as a loading control.

Supplemental Tables

Table S1 (Related to Figure 1). RSRS repeat-containing proteins functionally grouped

mRNA splicing	Other
Rbmx2	Rbbp6
Ccnl1	Ndrg1
Srsf2	Rbm26
Luc7l3	Ppargc1a
Clk2	Paf1
Prpf38a	Arglu1
Arl6ip4	Scaf8
Sfswap	Pdzd7
Rsrc1	Srrm3
Cwc25	Nktr
Rbm39	Pprc1
Srsf12	Snrnp70
Scaf4	Rlim
Srek1	Cherp
Pnn	Lbr
Clasrp	Nkap
Tra2b	Topors
Srsf5	Rsrc2
Thrap3	Rsrp1
Cactin	Sytl5
Srrm1	Erbb3
Scaf1	Bclaf1
Srsf7	Gpatch8
Acin1	Zc3h18
Srsf6	Luc7l
Znf638	Spata18
Prpf38b	Gtpbp4
Ddx46	Tjp2
Srrm2	Luc7l2
Srsf4	
Son	
Srsf1	
U2af2	
U2af1	
Ppig	
Tra2a	
Ccnl2	
Setd2	
Cir1	
Srsf3	
Dhx8	
Rnps1	
Pnisr	
Cdk13	
Snrnp27	
Srsf10	
Zranb2	
Prpf4b	

Table S2 (Related to Figure 1). Functional categorisation of RSRS repeat-containing proteins identified by ScanProsite

Entry	Gene names (primary)	Protein names	Function [CC]
Q8R0F5	Rbmx2	RNA-binding motif protein, X-linked 2	FUNCTION: Involved in pre-mRNA splicing as component of the activated spliceosome.
Q52KE7	Ccnl1	Cyclin-L1 (Cyclin-L) (Cyclin Ania-6a)	FUNCTION: Involved in pre-mRNA splicing. Functions in association with cyclin-dependent kinases (CDKs). May play a role in the regulation of RNA polymerase II (pol II). Inhibited by the CDK-specific inhibitor CDKN1A/p21.
P97868	Rbbp6	E3 ubiquitin-protein ligase RBBP6 (EC 2.3.2.27) (Proliferation potential-related protein) (Protein P2P-R) (RING-type E3 ubiquitin transferase RBBP6) (Retinoblastoma-binding protein 6) (p53-associated cellular protein of testis)	FUNCTION: E3 ubiquitin-protein ligase which promotes ubiquitination of YBX1, leading to its degradation by the proteasome (By similarity). May play a role as a scaffold protein to promote the assembly of the p53/TP53-MDM2 complex, resulting in increase of MDM2-mediated ubiquitination and degradation of p53/TP53; may function as negative regulator of p53/TP53, leading to both apoptosis and cell growth retardation (PubMed:17470788). Regulates DNA-replication and common fragile sites (CFS) stability in a ZBTB38- and MCM10-dependent manner. Controls ZBTB38 protein stability and abundance via ubiquitination and proteasomal degradation, and ZBTB38 in turn negatively regulates the expression of MCM10 which plays an important role in DNA-replication (PubMed:24726359).
Q62433	Ndrg1	Protein NDRG1 (N-myc downstream-regulated gene 1 protein) (Protein Ndr1)	FUNCTION: Stress-responsive protein involved in hormone responses, cell growth, and differentiation. Acts as a tumor suppressor in many cell types. Necessary but not sufficient for p53/TP53-mediated caspase activation and apoptosis. Required for vesicular recycling of CDH1 and TF. May also function in lipid trafficking. Protects cells from spindle disruption damage. Functions in p53/TP53-dependent mitotic spindle checkpoint. Regulates microtubule dynamics and maintains euploidy (By similarity). Has a role in cell trafficking notably of the Schwann cell and is necessary for the maintenance and development of the peripheral nerve myelin sheath.
Q6NZN0	Rbm26	RNA-binding protein 26 (Protein expressed in male leptotene and zygotene spermatocytes 393) (MLZ-393) (RNA-binding motif protein 26)	
O70343	Ppargc1a	Peroxisome proliferator-activated receptor gamma coactivator 1-alpha (PGC-1-alpha) (PPAR-gamma coactivator 1-alpha) (PPARGC-1-alpha)	FUNCTION: Transcriptional coactivator for steroid receptors and nuclear receptors. Greatly increases the transcriptional activity of PPARG and thyroid hormone receptor on the uncoupling protein promoter. Can regulate key mitochondrial genes that contribute to the program of adaptive thermogenesis. Plays an essential role in metabolic reprogramming in response to dietary availability through coordination of the expression of a wide array of genes involved in glucose and fatty acid metabolism. Induces the expression of PERM1 in the skeletal muscle in an ESRRA-dependent manner. Also involved in the integration of the circadian rhythms and energy metabolism. Required for oscillatory expression of clock genes, such as ARNTL/BMAL1 and NR1D1, through the coactivation of RORA and RORC, and metabolic genes, such as PDK4 and PEPCK. Isoform 4 specifically activates the expression of IGF1 and suppresses myostatin expression in skeletal muscle leading to muscle fiber hypertrophy.
Q8K2T8	Paf1	RNA polymerase II-associated factor 1 homolog	FUNCTION: Component of the PAF1 complex (PAF1C) which has multiple functions during transcription by RNA polymerase II and is implicated in regulation of development and maintenance of embryonic stem cell pluripotency. PAF1C associates with RNA polymerase II through interaction with POLR2A CTD non-phosphorylated and 'Ser-2'- and 'Ser-5'-phosphorylated forms and is involved in transcriptional elongation, acting both independently and synergistically with TCEA1 and in cooperation with the DSIF complex and HTATSF1. PAF1C is required for transcription of Hox and Wnt target genes. PAF1C is involved in hematopoiesis and stimulates transcriptional activity of KMT2A/MLL1. PAF1C is involved in histone modifications such as ubiquitination of histone H2B and methylation on histone H3 'Lys-4' (H3K4me3). PAF1C recruits the RNF20/40 E3 ubiquitin-protein ligase complex and the E2 enzyme UBE2A or UBE2B to chromatin which mediate monoubiquitination of 'Lys-120' of histone H2B (H2BK120ub1); UB2A/B-mediated H2B ubiquitination is proposed to be coupled to transcription. PAF1C is involved in mRNA 3' end formation probably through association with cleavage and poly(A) factors. Connects PAF1C with the RNF20/40 E3 ubiquitin-protein ligase complex. Involved in polyadenylation of mRNA precursors (By similarity).
Q62093	Srsf2	Serine/arginine-rich splicing factor 2 (Protein PR264) (Putative myelin regulatory factor 1) (MRF-1) (Splicing component, 35 kDa) (Splicing factor SC35) (SC-35) (Splicing factor, arginine/serine-rich 2)	FUNCTION: Necessary for the splicing of pre-mRNA. It is required for formation of the earliest ATP-dependent splicing complex and interacts with spliceosomal components bound to both the 5'- and 3'-splice sites during spliceosome assembly. It also is required for ATP-dependent interactions of both U1 and U2 snRNPs with pre-mRNA (By similarity). Can bind to the myelin basic protein (MBP) gene MB3 regulatory region and increase transcription of the mbp promoter in cells derived from the CNS. The phosphorylated form (by SRPK2) is required for cellular apoptosis in response to cisplatin treatment (By similarity).
Q5SUF2	Luc7l3	Luc7-like protein 3 (Cisplatin resistance-associated-overexpressed protein)	FUNCTION: Binds cAMP regulatory element DNA sequence. May play a role in RNA splicing (By similarity).
Q3UL36	Arglu1	Arginine and glutamate-rich protein 1	
O35491	Clk2	Dual specificity protein kinase CLK2 (EC 2.7.12.1) (CDC-like kinase 2)	FUNCTION: Dual specificity kinase acting on both serine/threonine and tyrosine-containing substrates. Phosphorylates serine- and arginine-rich (SR) proteins of the spliceosomal complex. May be a constituent of a network of regulatory mechanisms that enable SR proteins to control RNA splicing and can cause redistribution of SR proteins from speckles to a diffuse nucleoplasmic distribution. Acts as a suppressor of hepatic gluconeogenesis and glucose output by repressing PPARGC1A transcriptional activity on gluconeogenic genes via its phosphorylation. Phosphorylates PPP2R5B thereby stimulating the assembly of PP2A phosphatase with the PPP2R5B-AKT1 complex leading to dephosphorylation of AKT1. Phosphorylates: PTPN1, SRSF1 and SRSF3. Regulates the alternative splicing of tissue factor (F3) pre-mRNA in endothelial cells. Phosphorylates PAGE4 at several serine and threonine residues and this phosphorylation attenuates the ability of PAGE4 to potentiate the transcriptional activator activity of JUN (By similarity).
Q6DID3	Scaf8	SR-related and CTD-associated factor 8 (RNA-binding motif protein 16)	FUNCTION: Anti-terminator protein required to prevent early mRNA termination during transcription. Together with SCAF4, acts by suppressing the use of early, alternative poly(A) sites, thereby preventing the accumulation of non-functional truncated proteins. Mechanistically, associates with the phosphorylated C-terminal heptapeptide repeat domain (CTD) of the largest RNA polymerase II subunit (POLR2A), and subsequently binds nascent RNA upstream of early polyadenylation sites to prevent premature mRNA transcript cleavage and polyadenylation. Independently of SCAF4, also acts as a positive regulator of transcript elongation.
E9Q9W7	Pdzd7	PDZ domain-containing protein 7	FUNCTION: In cochlear developing hair cells, essential in organizing the USH2 complex at stereocilia ankle links (PubMed:24334608). Blocks inhibition of adenylate cyclase activity mediated by ADGRV1 (PubMed:24962568).
Q80WV7	Srrm3	Serine/arginine repetitive matrix protein 3	FUNCTION: May play a role in regulating breast cancer cell invasiveness. May be involved in RYBP-mediated breast cancer progression.
P30415	Nktr	NK-tumor recognition protein (NK-TR protein) (Natural-killer cells cyclophilin-related protein) (Peptidyl-prolyl cis-trans isomerase NKTR) (PPIase) (EC 5.2.1.8)	FUNCTION: PPIase that catalyzes the cis-trans isomerization of proline imidic peptide bonds in oligopeptides and may therefore assist protein folding. Component of a putative tumor-recognition complex involved in the function of NK cells.
Q4FK66	Prpf38a	Pre-mRNA-splicing factor 38A	FUNCTION: Involved in pre-mRNA splicing as a component of the spliceosome.
Q9JM93	Arl6ip4	ADP-ribosylation factor-like protein 6-interacting protein 4 (ARL-6-interacting protein 4) (Aip-4) (Splicing factor SRrp37)	FUNCTION: Involved in modulating alternative pre-mRNA splicing with either 5' distal site activation or preferential use of 3' proximal site.
Q3USH5	Sfswap	Splicing factor, suppressor of white-apricot homolog (Splicing factor, arginine/serine-rich 8) (Suppressor of white apricot protein homolog)	FUNCTION: Plays a role as an alternative splicing regulator. Regulates its own expression at the level of RNA processing. Also regulates the splicing of fibronectin and CD45 genes. May act, at least in part, by interaction with other R/S-containing splicing factors. Represses the splicing of MAPT/Tau exon 10 (By similarity).
Q6NZN1	Pprc1	Peroxisome proliferator-activated receptor gamma coactivator-related protein 1 (PGC-1-related coactivator) (PRC)	FUNCTION: Acts as a coactivator during transcriptional activation of nuclear genes related to mitochondrial biogenesis and cell growth. Involved in the transcription coactivation of CREB and NRF1 target genes (By similarity).
Q62376	Snrnp70	U1 small nuclear ribonucleoprotein 70 kDa (U1 snRNP 70 kDa) (U1-70K) (snRNP70)	FUNCTION: Component of the spliceosomal U1 snRNP, which is essential for recognition of the pre-mRNA 5' splice-site and the subsequent assembly of the spliceosome. SNRNP70 binds to the loop I region of U1-snRNA.; FUNCTION: [Isoform 2]: Truncated isoforms that lack the RRM domain cannot bind U1-snRNA.
Q9DBU6	Rsrc1	Serine/Arginine-related protein 53 (SRrp53) (Arginine/serine-rich coiled-coil protein 1)	FUNCTION: Plays a role in pre-mRNA splicing. Involved in both constitutive and alternative pre-mRNA splicing. May have a role in the recognition of the 3' splice site during the second step of splicing (By similarity).
Q9DBF7	Cwc25	Pre-mRNA-splicing factor CWC25 homolog (Coiled-coil domain-containing protein 49) (Spliceosome-associated protein homolog CWC25)	FUNCTION: Involved in pre-mRNA splicing as component of the spliceosome.
Q8VH51	Rbm39	RNA-binding protein 39 (Coactivator of activating protein 1 and estrogen receptors) (Coactivator of AP-1 and ERs) (RNA-binding motif protein 39) (RNA-binding region-containing protein 2) (Transcription coactivator CAPER)	FUNCTION: Transcriptional coactivator for steroid nuclear receptors ESR1/ER-alpha and ESR2/ER-beta, and JUN/AP-1. May be involved in pre-mRNA splicing process.
Q8C8K3	Srsf12	Serine/arginine-rich splicing factor 12 (Splicing factor, arginine/serine-rich 13B)	FUNCTION: Splicing factor that seems to antagonize SR proteins in pre-mRNA splicing regulation.
Q7TSH6	Scaf4	SR-related and CTD-associated factor 4 (CTD-binding SR-like protein RA4) (Splicing factor, arginine/serine-rich 15)	FUNCTION: Anti-terminator protein required to prevent early mRNA termination during transcription. Together with SCAF8, acts by suppressing the use of early, alternative poly(A) sites, thereby preventing the accumulation of non-functional truncated proteins. Mechanistically, associates with the phosphorylated C-terminal heptapeptide repeat domain (CTD) of the largest RNA polymerase II subunit (POLR2A), and subsequently binds nascent RNA upstream of early polyadenylation sites to prevent premature mRNA transcript cleavage and polyadenylation. Independently of SCAF8, also acts as a suppressor of transcriptional readthrough.
Q9WTV7	Rlim	E3 ubiquitin-protein ligase RLIM (EC 2.3.2.27) (LIM domain-interacting RING finger protein) (RING finger LIM domain-binding protein) (R-LIM) (RING finger protein 12) (RING-type E3 ubiquitin transferase RLIM)	FUNCTION: E3 ubiquitin-protein ligase that acts as a negative coregulator for LIM homeodomain transcription factors by mediating the ubiquitination and subsequent degradation of LIM cofactors LDB1 and LDB2 and by mediating the recruitment the SIN3a/histone deacetylase corepressor complex. Ubiquitination and degradation of LIM cofactors LDB1 and LDB2 allows DNA-bound LIM homeodomain transcription factors to interact with other protein partners such as RLIM. Plays a role in telomere length-mediated growth suppression by mediating the ubiquitination and degradation of TERF1. By targeting ZFP42 for degradation, acts as an activator of random inactivation of X chromosome in the embryo, a stochastic process in which one X chromosome is inactivated to minimize sex-related dosage differences of X-encoded genes in somatic cells of female placental mammals.
Q8BZX4	Srek1	Splicing regulatory glutamine/lysine-rich protein 1 (Serine/arginine-rich-splicing regulatory protein 86) (SRrp86) (Splicing factor, arginine/serine-rich 12)	FUNCTION: Participates in the regulation of alternative splicing by modulating the activity of other splice facors. Inhibits the splicing activity of SFRS1, SFRS2 and SFRS6. Augments the splicing activity of SFRS3 (By similarity).
O35691	Pnn	Pinin	FUNCTION: Transcriptional activator binding to the E-box 1 core sequence of the E-cadherin promoter gene; the core-binding sequence is 5'CAGGTG-3'. Capable of reversing CTBP1-mediated transcription repression. Auxiliary component of the splicing-dependent multiprotein exon junction complex (EJC) deposited at splice junction on mRNAs. The EJC is a dynamic structure consisting of core proteins and several peripheral nuclear and cytoplasmic associated factors that join the complex only transiently either during EJC assembly or during subsequent mRNA metabolism. Participates in the regulation of alternative pre-mRNA splicing. Associates to spliced mRNA within 60 nt upstream of the 5'-splice sites. Component of the PSAP complex which binds RNA in a sequence-independent manner and is proposed to be recruited to the EJC prior to or during the splicing process and to regulate specific excision of introns in specific transcription subsets. Involved in the establishment and maintenance of epithelia cell-cell adhesion (By similarity).
Q8CFC7	Clasrp	CLK4-associating serine/arginine rich protein (Clk4-associating SR-related protein) (Serine/arginine-rich splicing factor 16) (Splicing factor, arginine/serine-rich 16) (Suppressor of white-apricot homolog 2)	FUNCTION: Probably functions as an alternative splicing regulator. May regulate the mRNA splicing of genes such as CLK1. May act by regulating members of the CLK kinase family.
Q8CGZ0	Cherp	Calcium homeostasis endoplasmic reticulum protein (SR-related CTD-associated factor 6)	FUNCTION: Involved in calcium homeostasis, growth and proliferation.

Q3U9G9	Lbr	Delta(14)-sterol reductase LBR (Delta-14-SR) (EC 1.3.1.70) (3-beta-hydroxysterol Delta (14)-reductase) (C-14 sterol reductase) (C14SR) (Integral nuclear envelope inner membrane protein) (Lamin-B receptor) (Sterol C14-reductase)	FUNCTION: Catalyzes the reduction of the C14-unsaturated bond of lanosterol, as part of the metabolic pathway leading to cholesterol biosynthesis (PubMed:18785926). Plays a critical role in myeloid cell cholesterol biosynthesis which is essential to both myeloid cell growth and functional maturation (PubMed:22140257). Mediates the activation of NADPH oxidases, perhaps by maintaining critical levels of cholesterol required for membrane lipid raft formation during neutrophil differentiation (PubMed:22140257). Anchors the lamina and the heterochromatin to the inner nuclear membrane (By similarity).
P62996	Tra2b	Transformer-2 protein homolog beta (TRA-2 beta) (TRA2-beta) (Silica-induced gene 41 protein) (SIG-41) (Splicing factor, arginine/serine-rich 10) (Transformer-2 protein homolog B)	FUNCTION: Sequence-specific RNA-binding protein which participates in the control of pre-mRNA splicing. Can either activate or suppress exon inclusion. Acts additively with RBMX to promote exon 7 inclusion of the survival motor neuron SMN2. Activates the splicing of MAPT/Tau exon 10. Alters pre-mRNA splicing patterns by antagonizing the effects of splicing regulators, like RBMX. Binds to the AG-rich SE2 domain in the SMN exon 7 RNA. Binds to pre-mRNA (By similarity).
O35326	Srsf5	Serine/arginine-rich splicing factor 5 (Delayed-early protein HRS) (Pre-mRNA-splicing factor SRP40) (Splicing factor, arginine/serine-rich 5)	FUNCTION: May be required for progression through G1 and entry into S phase of cell growth. May play a regulatory role in pre-mRNA splicing. Autoregulates its own expression. Plays a role in constitutive splicing and can modulate the selection of alternative splice sites (By similarity).
Q569Z6	Thrap3	Thyroid hormone receptor-associated protein 3 (Thyroid hormone receptor-associated protein complex 150 kDa component) (Trap150)	FUNCTION: Involved in pre-mRNA splicing. Remains associated with spliced mRNA after splicing which probably involves interactions with the exon junction complex (EJC). Can trigger mRNA decay which seems to be independent of nonsense-mediated decay involving premature stop codons (PTC) recognition. May be involved in nuclear mRNA decay. Involved in regulation of signal-induced alternative splicing. During splicing of PTPRC/CD45 is proposed to sequester phosphorylated SFPQ from PTPRC/CD45 pre-mRNA in resting T-cells. Involved in cyclin-D1/CCND1 mRNA stability probably by acting as component of the SNARP complex which associates with both the 3'end of the CCND1 gene and its mRNA. Involved in response to DNA damage. Is excluded from DNA damage sites in a manner that parallels transcription inhibition; the function may involve the SNARP complex. Initially thought to play a role in transcriptional coactivation through its association with the TRAP complex; however, it is not regarded as a stable Mediator complex subunit. Cooperatively with HELZ2, enhances the transcriptional activation mediated by PPARG, maybe through the stabilization of the PPARG binding to DNA in presence of ligand. May play a role in the terminal stage of adipocyte differentiation. Plays a role in the positive regulation of the circadian clock. Acts as a coactivator of the CLOCK-ARNTL/BMAL1 heterodimer and promotes its transcriptional activator activity and binding to circadian target genes (PubMed:24043798).
Q9D0F4	Nkap	NF-kappa-B-activating protein	FUNCTION: Acts as a transcriptional repressor. Plays a role as a transcriptional corepressor of the Notch-mediated signaling required for T-cell development. Also involved in the TNF and IL-1 induced NF-kappa-B activation. Associates with chromatin at the Notch-regulated SKP2 promoter (By similarity).
Q80Z37	Topors	E3 ubiquitin-protein ligase Topors (EC 2.3.2.27) (RING-type E3 ubiquitin transferase Topors) (SUMO1-protein E3 ligase Topors) (Topoisomerase I-binding RING finger protein) (Topoisomerase I-binding arginine/serine-rich protein) (Tumor suppressor p53-binding protein 3) (p53-binding protein 3) (p53BP3)	FUNCTION: Functions as an E3 ubiquitin-protein ligase and as a E3 SUMO1-protein ligase. Probable tumor suppressor involved in cell growth, cell proliferation and apoptosis that regulates p53/TP53 stability through ubiquitin-dependent degradation. May regulate chromatin modification through sumoylation of several chromatin modification-associated proteins. May be involved in DNA-damage-induced cell death through IKBKE sumoylation.
A2RTL5	Rsrc2	Arginine/serine-rich coiled-coil protein 2	
Q3UC65	Rsrp1	Arginine/serine-rich protein 1	
Q80T23	Sytl5	Synaptotagmin-like protein 5	FUNCTION: May act as Rab effector protein and play a role in vesicle trafficking. Binds phospholipids (By similarity).
Q61526	Erbb3	Receptor tyrosine-protein kinase erbB-3 (EC 2.7.10.1) (Glial growth factor receptor) (Proto-oncogene-like protein c-ErbB-3)	FUNCTION: Tyrosine-protein kinase that plays an essential role as cell surface receptor for neuregulins. Binds to neuregulin-1 (NRG1) and is activated by it; ligand-binding increases phosphorylation on tyrosine residues and promotes its association with the p85 subunit of phosphatidylinositol 3-kinase. May also be activated by CSPG5. Involved in the regulation of myeloid cell differentiation.
Q9CS00	Cactin	Cactin	FUNCTION: Involved in the regulation of innate immune response. Acts as negative regulator of Toll-like receptor and interferon-regulatory factor (IRF) signaling pathways. Contributes to the regulation of transcriptional activation of NF-kappa-B target genes in response to endogenous proinflammatory stimuli. May play a role during early embryonic development. Probably involved in pre-mRNA splicing (By similarity).
Q52KI8	Srrm1	Serine/arginine repetitive matrix protein 1 (Plenty-of-prolines 101)	FUNCTION: Part of pre- and post-splicing multiprotein mRNP complexes. Involved in numerous pre-mRNA processing events. Promotes constitutive and exonic splicing enhancer (ESE)-dependent splicing activation by bridging together sequence-specific (SR family proteins, SFRS4, SFRS5 and TRA2B/SFRS10) and basal snRNP (SNRP70 and SNRPA1) factors of the spliceosome. Stimulates mRNA 3'-end cleavage independently of the formation of an exon junction complex. Binds both pre-mRNA and spliced mRNA 20-25 nt upstream of exon-exon junctions. Binds RNA and DNA with low sequence specificity and has similar preference for either double- or single-stranded nucleic acid substrates.
Q8K019	Bclaf1	Bcl-2-associated transcription factor 1 (Btf)	FUNCTION: Death-promoting transcriptional repressor. May be involved in cyclin-D1/CCND1 mRNA stability through the SNARP complex which associates with both the 3'end of the CCND1 gene and its mRNA (By similarity).
Q5U4C3	Scaf1	Splicing factor, arginine/serine-rich 19 (SR-related and CTD-associated factor 1)	FUNCTION: May function in pre-mRNA splicing.
Q8BL97	Srsf7	Serine/arginine-rich splicing factor 7 (Splicing factor, arginine/serine-rich 7)	FUNCTION: Required for pre-mRNA splicing. Represses the splicing of MAPT/Tau exon 10. May function as export adapter involved in mRNA nuclear export such as of histone H2A. Binds mRNA which is thought to be transferred to the NXF1-NXT1 heterodimer for export (TAP/NXF1 pathway); enhances NXF1-NXT1 RNA-binding activity. RNA-binding is semi-sequence specific (By similarity).
A2A6A1	Gpatch8	G patch domain-containing protein 8	
Q0P678	Zc3h18	Zinc finger CCCH domain-containing protein 18 (Nuclear protein NHN1)	
Q9JIX8	Acin1	Apoptotic chromatin condensation inducer in the nucleus (Acinus)	FUNCTION: Auxiliary component of the splicing-dependent multiprotein exon junction complex (EJC) deposited at splice junction on mRNAs. The EJC is a dynamic structure consisting of core proteins and several peripheral nuclear and cytoplasmic associated factors that join the complex only transiently either during EJC assembly or during subsequent mRNA metabolism. Component of the ASAP complexes which bind RNA in a sequence-independent manner and are proposed to be recruited to the EJC prior to or during the splicing process and to regulate specific excision of introns in specific transcription subsets; ACIN1 confers RNA-binding to the complex. The ASAP complex can inhibit RNA processing during in vitro splicing reactions. The ASAP complex promotes apoptosis and is disassembled after induction of apoptosis. Involved in the splicing modulation of BCL2L1/Bcl-X (and probably other apoptotic genes); specifically inhibits formation of proapoptotic isoforms such as Bcl-X(S); the activity is different from the established EJC assembly and function. Induces apoptotic chromatin condensation after activation by CASP3. Regulates cyclin A1, but not cyclin A2, expression in leukemia cells (By similarity).
Q3TWW8	Srsf6	Serine/arginine-rich splicing factor 6 (Pre-mRNA-splicing factor SRP55) (Splicing factor, arginine/serine-rich 6)	FUNCTION: Plays a role in constitutive splicing and modulates the selection of alternative splice sites. Plays a role in the alternative splicing of MAPT/Tau exon 10. Binds to alternative exons of TNC pre-mRNA and promotes the expression of alternatively spliced TNC. Plays a role in wound healing and in the regulation of keratinocyte differentiation and proliferation via its role in alternative splicing (By similarity).
Q61464	Znf638	Zinc finger protein 638 (Nuclear protein 220) (Zinc finger matrin-like protein)	FUNCTION: Transcription factor that binds to cytidine clusters in double-stranded DNA (By similarity). Plays a key role in the silencing of unintegrated retroviral DNA: some part of the retroviral DNA formed immediately after infection remains unintegrated in the host genome and is transcriptionally repressed (PubMed:30487602). Mediates transcriptional repression of unintegrated viral DNA by specifically binding to the cytidine clusters of retroviral DNA and mediating the recruitment of chromatin silencers, such as the HUSH complex, SETDB1 and the histone deacetylases HDAC1 and HDAC4 (PubMed:30487602). Acts as an early regulator of adipogenesis by acting as a transcription cofactor of CEBPs (CEBPA, CEBPD and/or CEBPG), controlling the expression of PPARG and probably of other proadipogenic genes, such as SREBF1 (PubMed:21602272). May also regulate alternative splicing of target genes during adipogenesis (PubMed:25024404).
Q80SY5	Prpf38b	Pre-mRNA-splicing factor 38B	FUNCTION: May be required for pre-mRNA splicing.
Q569Z5	Ddx46	Probable ATP-dependent RNA helicase DDX46 (EC 3.6.4.13) (DEAD box protein 46)	FUNCTION: Plays an essential role in splicing, either prior to, or during A complex formation.
Q8BTI8	Srrm2	Serine/arginine repetitive matrix protein 2	FUNCTION: Required for pre-mRNA splicing as component of the spliceosome.
Q8VE97	Srsf4	Serine/arginine-rich splicing factor 4 (Splicing factor, arginine/serine-rich 4)	FUNCTION: Plays a role in alternative splice site selection during pre-mRNA splicing. Represses the splicing of MAPT/Tau exon 10 (By similarity).
Q9QX47	Son	Protein SON (Negative regulatory element-binding protein) (NRE-binding protein)	FUNCTION: RNA-binding protein that acts as a mRNA splicing cofactor by promoting efficient splicing of transcripts that possess weak splice sites. Specifically promotes splicing of many cell-cycle and DNA-repair transcripts that possess weak splice sites, such as TUBG1, KATNB1, TUBGCP2, AURKB, PCNT, AKT1, RAD23A, and FANCG. Probably acts by facilitating the interaction between Serine/arginine-rich proteins such as SRSF2 and the RNA polymerase II. Also binds to DNA; binds to the consensus DNA sequence: 5'-GA[GT]AN[CG][AG]CC-3' (By similarity). Essential for correct RNA splicing of multiple genes critical for brain development, neuronal migration and metabolism, including TUBG1, FLNA, PNKP, WDR62, PSMD3, PCK2, PFKL, IDH2, and ACY1 (By similarity). May also regulate the ghrelin signaling in hypothalamic neuron by acting as a negative regulator of GHSR expression (PubMed:20876580).
Q6PDM2	Srsf1	Serine/arginine-rich splicing factor 1 (ASF/SF2) (Pre-mRNA-splicing factor SRp30a) (Splicing factor, arginine/serine-rich 1)	FUNCTION: Plays a role in preventing exon skipping, ensuring the accuracy of splicing and regulating alternative splicing. Interacts with other spliceosomal components, via the RS domains, to form a bridge between the 5'- and 3'-splice site binding components, U1 snRNP and U2AF. Can stimulate binding of U1 snRNP to a 5'-splice site-containing pre-mRNA. Binds to purine-rich RNA sequences, either the octamer, 5'-RGAAGAAC-3' (r=A or G) or the decamers, AGGACAGAGC/AGGACGAAGC. Binds preferentially to the 5'-CGAGGCG-3' motif in vitro. Three copies of the octamer constitute a powerful splicing enhancer in vitro, the ASF/SF2 splicing enhancer (ASE) which can specifically activate ASE-dependent splicing (By similarity). Specifically regulates alternative splicing of cardiac isoforms of CAMK2D, LDB3/CYPHER and TNNT2/CTNT during heart remodeling at the juvenile to adult transition. The inappropriate accumulation of a neonatal and neuronal isoform of CAMKD2 in the adult heart results in aberrant calcium handling and defective excitation-contraction coupling in cardiomyocytes. May function as export adapter involved in mRNA nuclear export through the TAP/NXF1 pathway (PubMed:15652482).
P26369	U2af2	Splicing factor U2AF 65 kDa subunit (U2 auxiliary factor 65 kDa subunit) (U2 snRNP auxiliary factor large subunit)	FUNCTION: Plays a role in pre-mRNA splicing and 3'-end processing. By recruiting PRPF19 and the PRP19C/Prp19 complex/NTC/Nineteen complex to the RNA polymerase II C-terminal domain (CTD), and thereby pre-mRNA, may couple transcription to splicing. Required for the export of mRNA out of the nucleus, even if the mRNA is encoded by an intron-less gene. Positively regulates pre-mRNA 3'-end processing by recruiting the CFIm complex to cleavage and polyadenylation signals.
Q9D883	U2af1	Splicing factor U2AF 35 kDa subunit (U2 auxiliary factor 35 kDa subunit) (U2 snRNP auxiliary factor small subunit)	FUNCTION: Plays a critical role in both constitutive and enhancer-dependent splicing by mediating protein-protein interactions and protein-RNA interactions required for accurate 3'-splice site selection. Recruits U2 snRNP to the branch point. Directly mediates interactions between U2AF2 and proteins bound to the enhancers and thus may function as a bridge between U2AF2 and the enhancer complex to recruit it to the adjacent intron (By similarity).
A2AR02	Ppig	Peptidyl-prolyl cis-trans isomerase G (PPIase G) (Peptidyl-prolyl isomerase G) (EC 5.2.1.8) (Cyclophilin G) (Rotamase G)	FUNCTION: PPIase that catalyzes the cis-trans isomerization of proline imidic peptide bonds in oligopeptides and may therefore assist protein folding. May be implicated in the folding, transport, and assembly of proteins. May play an important role in the regulation of pre-mRNA splicing.
Q6PFR5	Tra2a	Transformer-2 protein homolog alpha (TRA-2 alpha) (TRA2-alpha) (Transformer-2 protein homolog A)	FUNCTION: Sequence-specific RNA-binding protein which participates in the control of pre-mRNA splicing.
Q9JJA7	Ccnl2	Cyclin-L2 (Cyclin Ania-6b) (Paneth cell-enhanced expression protein) (PCEE)	FUNCTION: Involved in pre-mRNA splicing. May induce cell death, possibly by acting on the transcription and RNA processing of apoptosis-related factors.
E9Q5F9	Setd2	Histone-lysine N-methyltransferase SETD2 (EC 2.1.1.-) (Lysine N-methyltransferase 3A) (Protein-lysine N-methyltransferase SETD2) (EC 2.1.1.-) (SET domain-containing protein 2)	FUNCTION: Histone methyltransferase that specifically trimethylates 'Lys-36' of histone H3 (H3K36me3) using dimethylated 'Lys-36' (H3K36me2) as substrate (PubMed:18157086, PubMed:20133625). Represents the main enzyme generating H3K36me3, a specific tag for epigenetic transcriptional activation (PubMed:18157086, PubMed:20133625). Plays a role in chromatin structure modulation during elongation by coordinating recruitment of the FACT complex and by interacting with hyperphosphorylated POLR2A (By similarity). Acts as a key regulator of DNA mismatch repair in G1 and early S phase by generating H3K36me3, a mark required to recruit MSH6 subunit of the MutS alpha complex: early recruitment of the MutS alpha complex to chromatin to be replicated allows a quick identification of mismatch DNA to initiate the mismatch repair reaction (By similarity). Required for DNA double-strand break repair in response to DNA damage: acts by mediating formation of H3K36me3, promoting recruitment of RAD51 and DNA repair via homologous recombination (HR) (By similarity). Acts as a tumor suppressor (By similarity). H3K36me3 also plays an essential role in the maintenance of a heterochromatic state, by recruiting DNA methyltransferase DNMT3A (By similarity). H3K36me3 is also enhanced in intron-containing genes, suggesting that SETD2 recruitment is enhanced by splicing and that splicing is coupled to recruitment of elongating RNA polymerase (By similarity). Required during angiogenesis (PubMed:20133625). Required for endoderm development by promoting embryonic stem cell differentiation toward endoderm: acts by mediating formation of H3K36me3 in distal promoter regions of FGFR3, leading to regulate transcription initiation of FGFR3

			(PubMed:25242323). In addition to histones, also mediates methylation of other proteins, such as tubulins and STAT1 (PubMed:27518565). Trimethylates 'Lys-40' of alpha-tubulins such as TUBA1B (alpha-TubK40me3); alpha-TubK40me3 is required for normal mitosis and cytokinesis and may be a specific tag in cytoskeletal remodeling (PubMed:27518565). Involved in interferon-alpha-induced antiviral defense by mediating both monomethylation of STAT1 at 'Lys-525' and catalyzing H3K36me3 on promoters of some interferon-stimulated genes (ISGs) to activate gene transcription (By similarity).
Q9CYI4	Luc7l	Putative RNA-binding protein Luc7-like 1	FUNCTION: May bind to RNA via its Arg/Ser-rich domain.
Q0P557	Spata18	Mitochondria-eating protein (Spermatogenesis-associated protein 18)	FUNCTION: Key regulator of mitochondrial quality that mediates the repairing or degradation of unhealthy mitochondria in response to mitochondrial damage. Mediator of mitochondrial protein catabolic process (also named MALM) by mediating the degradation of damaged proteins inside mitochondria by promoting the accumulation in the mitochondrial matrix of hydrolases that are characteristic of the lysosomal lumen. Also involved in mitochondrion degradation of damaged mitochondria by promoting the formation of vacuole-like structures (named MIV), which engulf and degrade unhealthy mitochondria by accumulating lysosomes. May have a role in spermatogenesis, especially in cell differentiation from late elongate spermatids to mature spermatozoa (By similarity). The physical interaction of SPATA18/MIEAP, BNIP3 and BNIP3L/NIX at the mitochondrial outer membrane regulates the opening of a pore in the mitochondrial double membrane in order to mediate the translocation of lysosomal proteins from the cytoplasm to the mitochondrial matrix (By similarity).
Q9DA19	Cir1	Corepressor interacting with RBPJ 1 (CBF1-interacting corepressor)	FUNCTION: Regulates transcription and acts as corepressor for RBPJ. Recruits RBPJ to the Sin3-histone deacetylase complex (HDAC). Required for RBPJ-mediated repression of transcription (By similarity). May modulate splice site selection during alternative splicing of pre-mRNAs.
P84104	Srsf3	Serine/arginine-rich splicing factor 3 (Pre-mRNA-splicing factor SRP20) (Protein X16) (Splicing factor, arginine/serine-rich 3)	FUNCTION: Splicing factor that specifically promotes exon-inclusion during alternative splicing. Interaction with YTHDC1, a RNA-binding protein that recognizes and binds N6-methyladenosine (m6A)-containing RNAs, promotes recruitment of SRSF3 to its mRNA-binding elements adjacent to m6A sites, leading to exon-inclusion during alternative splicing. Also functions as export adapter involved in mRNA nuclear export. Binds mRNA which is thought to be transferred to the NXF1-NXT1 heterodimer for export (TAP/NXF1 pathway); enhances NXF1-NXT1 RNA-binding activity. Involved in nuclear export of m6A-containing mRNAs via interaction with YTHDC1: interaction with YTHDC1 facilitates m6A-containing mRNA-binding to both SRSF3 and NXF1, promoting mRNA nuclear export. RNA-binding is semi-sequence specific.
A2A4P0	Dhx8	ATP-dependent RNA helicase DHX8 (EC 3.6.4.13) (DEAH box protein 8)	FUNCTION: Involved in pre-mRNA splicing as component of the spliceosome. Facilitates nuclear export of spliced mRNA by releasing the RNA from the spliceosome.
Q99M28	Rnps1	RNA-binding protein with serine-rich domain 1	FUNCTION: Part of pre- and post-splicing multiprotein mRNP complexes. Auxiliary component of the splicing-dependent multiprotein exon junction complex (EJC) deposited at splice junction on mRNAs. The EJC is a dynamic structure consisting of core proteins and several peripheral nuclear and cytoplasmic associated factors that join the complex only transiently either during EJC assembly or during subsequent mRNA metabolism. Component of the ASAP and PSAP complexes which bind RNA in a sequence-independent manner and are proposed to be recruited to the EJC prior to or during the splicing process and to regulate specific excision of introns in specific transcription subsets. The ASAP complex can inhibit RNA processing during in vitro splicing reactions. The ASAP complex promotes apoptosis and is disassembled after induction of apoptosis. Enhances the formation of the ATP-dependent A complex of the spliceosome. Involved in both constitutive splicing and, in association with SRP54 and TRA2B/SFRS10, in distinctive modulation of alternative splicing in a substrate-dependent manner. Involved in the splicing modulation of BCL2L1/Bcl-X (and probably other apoptotic genes); specifically inhibits formation of proapoptotic isoforms such as Bcl-X(S); the activity is different from the established EJC assembly and function. Participates in mRNA 3'-end cleavage. Involved in UPF2-dependent nonsense-mediated decay (NMD) of mRNAs containing premature stop codons. Also mediates increase of mRNA abundance and translational efficiency. Binds spliced mRNA 20-25 nt upstream of exon-exon junctions (By similarity).
A2AJT4	Pnisr	Arginine/serine-rich protein PNISR (Serine/arginine-rich-splicing regulatory protein 130) (SRrp130) (Splicing factor, arginine/serine-rich 130) (Splicing factor, arginine/serine-rich 18)	
Q69ZA1	Cdk13	Cyclin-dependent kinase 13 (EC 2.7.11.22) (EC 2.7.11.23) (CDC2-related protein kinase 5) (Cell division cycle 2-like protein kinase 5) (Cell division protein kinase 13)	FUNCTION: Cyclin-dependent kinase which displays CTD kinase activity and is required for RNA splicing. Has CTD kinase activity by hyperphosphorylating the C-terminal heptapeptide repeat domain (CTD) of the largest RNA polymerase II subunit RPB1, thereby acting as a key regulator of transcription elongation. Required for RNA splicing, probably by phosphorylating SRSF1/SF2. Required during hematopoiesis.
Q99ME9	Gtpbp4	Nucleolar GTP-binding protein 1 (Chronic renal failure gene protein) (GTP-binding protein NGB)	FUNCTION: Involved in the biogenesis of the 60S ribosomal subunit.
Q8K194	Snrnp27	U4/U6.U5 small nuclear ribonucleoprotein 27 kDa protein (U4/U6.U5 snRNP 27 kDa protein) (U4/U6.U5-27K) (U4/U6.U5 tri-snRNP-associated protein 3)	FUNCTION: May play a role in mRNA splicing.
Q9R0U0	Srsf10	Serine/arginine-rich splicing factor 10 (FUS-interacting serine-arginine-rich protein 1) (Neural-salient serine/arginine-rich protein) (Neural-specific SR protein) (Splicing factor, arginine/serine-rich 13A) (TLS-associated protein with Ser-Arg repeats) (TASR) (TLS-associated protein with SR repeats) (TLS-associated serine-arginine protein) (TLS-associated SR protein)	FUNCTION: Splicing factor that in its dephosphorylated form acts as a general repressor of pre-mRNA splicing. Seems to interfere with the U1 snRNP 5'-splice recognition of SNRNP70. Required for splicing repression in M-phase cells and after heat shock. Also acts as a splicing factor that specifically promotes exon skipping during alternative splicing. Interaction with YTHDC1, a RNA-binding protein that recognizes and binds N6-methyladenosine (m6A)-containing RNAs, prevents SRSF10 from binding to its mRNA-binding sites close to m6A-containing regions, leading to inhibit exon skipping during alternative splicing (By similarity). May be involved in regulation of alternative splicing in neurons (PubMed:10583508).
Q9R020	Zranb2	Zinc finger Ran-binding domain-containing protein 2 (Zinc finger protein 265) (Zinc finger, splicing)	FUNCTION: Splice factor required for alternative splicing of TRA2B/SFRS10 transcripts. May interfere with constitutive 5'-splice site selection (By similarity).
Q61136	Prpf4b	Serine/threonine-protein kinase PRP4 homolog (EC 2.7.11.1) (PRP4 pre-mRNA-processing factor 4 homolog) (Pre-mRNA protein kinase)	FUNCTION: Has a role in pre-mRNA splicing. Phosphorylates SF2/ASF.
Q9Z0U1	Tjp2	Tight junction protein ZO-2 (Tight junction protein 2) (Zona occludens protein 2) (Zonula occludens protein 2)	FUNCTION: Plays a role in tight junctions and adherens junctions.
Q7TNC4	Luc7l2	Putative RNA-binding protein Luc7-like 2 (CGI-74 homolog)	FUNCTION: May bind to RNA via its Arg/Ser-rich domain.

Table S3 (Related to Figure 2). RNF12 phosphorylation sites identified by immunoprecipitation-mass spectrometry

**Experiment 1**					
**pep_exp_mz**	**pep_exp_mr**	**pep_score**	**pep_seq**	**pep_var_mod**	**residue**
516.5920	1546.7504	23	R.SRSPLQPTSEIPR.R	P (ST)	S227, S229
801.8524	1601.6868	(26)	R.RL**S**VENMESSSQR.Q	P (ST)	S163
809.8495	1617.6818	(27)	R.RL**S**VENMESSSQR.Q	O (M); P (ST)	S163
**Experiment 2**					
**pep_exp_mz**	**pep_exp_mr**	**pep_score**	**pep_seq**	**pep_var_mod**	**residue**
701.786	1401.5562	30	EGPPPQ**S**PDENR	P (ST)	S78
774.3826	1546.7504	40	SR**S**PLQPTSEIPR	P (ST)	S229
801.8502	1601.6868	46	RL**S**VENMESSSQR	P (ST)	S163
809.8483	1617.6818	62	RL**S**VENMESSSQR	O (M); P (ST)	S163
1230.5322	2459.0489	78	AGESSDDVTNSDSIIDWLNSVR	P (ST)	S88, S89
1255.2161	3762.6282	32	EGPPPQSPDENRAGESSDDVTNSDSIIDWLNSVR	P (ST)	S78, S88, S89
**Experiment 3**					
**pep_exp_mz**	**pep_exp_mr**	**pep_score**	**pep_seq**	**pep_var_mod**	**residue**
701.7868	1401.559	48	EGPPPQ**S**PDENR	P (ST)	S78
774.3839	1546.7531	44	SRSPLQPTSEIPR	P (ST)	S227
516.5918	1546.7536	27	SRSPLQPTSEIPR	P (ST)??	S227, S229, T234
809.8502	1617.6858	68	RL**S**VENMESSSQR	O (M); P (ST)	S163
814.3668	1626.719	37	**S**R**S**PLQPTSEIPR	2 P (ST)	S227, S229
661.9747	1982.9024	19	AERSRSPLQPTSEIPR	2 P (ST)??	S227, S229, T234, S235
**Experiment 4**					
**pep_exp_mz**	**pep_exp_mr**	**pep_score**	**pep_seq**	**pep_var_mod**	**residue**
474.7062	947.3979	29	SRSPEHR	P (ST)??	S212, S214
701.7858	1401.5571	30	EGPPPQ**S**PDENR	P (ST)	S78
774.3807	1546.7469	43	SR**S**PLQPTSEIPR	P (ST)	S229
809.8483	1617.6821	50	RLSVENMESSSQR	O (M); P (ST)??	S163
814.3668	1626.719	22	SRSPLQPTSEIPR	2 P (ST)??	S227, S229, T234, S235
905.9172	1809.8198	21	AERNSAEAVTEVPTTR	P (ST)??	S194, T199
1230.5315	2459.0484	73	AGESSDDVTNSDSIIDWLNSVR	P (ST)??	S88, S89, T93,
1255.2163	3762.6271	39	EGPPPQSPDENRAGESSDDVTNSDSIIDWLNSVR	P (ST)	S78

Phosphosite Localisation data obtained from Proteome Discoverer 1.4-SP1 –PhosphoRS3.1 or Proteome Discoverer 2.0-ptmRS. Underlined S T is interpretation of Mascot and MS2 data. Bold S T is a very good assignment, S T is used where identification is not certain, ?? means phosphorylation could be in any of the sites. pep_exp_mz: Observed or experimental m/z value, pep_exp_mr: Molecular mass calculated from experimental m/z value, pep_score: Mascot score for PSM (Peptide sequence match), pep_seq: Peptide sequence in 1 letter code, pep_var_mod: Variable modifications from all sources as list of names.

Table S4 (Related to Figure 2). RNF12 phosphorylation sites identified via SRPK in vitro phosphorylation and mass spectrometry

**SRPK1 5 min**				
**pep_exp_mz**	**pep_exp_mr**	**pep_score**	**pep_seq**	**pep_var_mod**
460.2178	918.4212	(24)	R.TYVSTIR.I	P (ST)
639.3107	1276.6176	(20)	R.QQI**S**GPELLGR.G	P (ST)
652.8156	1303.6173	(31)	R.**S**PLQPTSEIPR.R	P (ST)
516.5908	1546.7504	(22)	R.SRSPLQPTSEIPR.R	P (ST)
774.3820	1546.7504	(47)	R.SRSPLQPTSEIPR.R	P (ST)
543.2460	1626.7168	(26)	R.**S**R**S**PLQPTSEIPR.R	2 P (ST)
814.3652	1626.7168	(41)	R.**S**R**S**PLQPTSEIPR.R	2 P (ST)
838.3497	1674.6886	(46)	R.**S**QAPNNTVTYESER.G	P (ST)
959.9175	1917.8218	85	R.SR**S**QAPNNTVTYESER.G	P (ST)
640.2810	1917.8218	(48)	R.SRSQAPNNTVTYESER.G	P (ST)
992.4553	1982.8976	(28)	R.AERSRSPLQPTSEIPR.R	2 P (ST)
661.9727	1982.8976	40	R.AERSRSPLQPTSEIPR.R	2 P (ST)
1027.4431	2052.8749	(29)	R.RAPTLEQSSENEPEGSSR.T	P (ST)
685.2983	2052.8749	(60)	R.RAPTLEQSSENEPEGSSR.T	P (ST)
689.6444	2065.9205	(21)	R.DNNLLGTPGESTEEELLR.R	P (ST)
771.0147	2310.0237	38	R.RAPTLEQSSENEPEGSSRTR.H	P (ST)
**SRPK1 60 min**				
**pep_exp_mz**	**pep_exp_mr**	**pep_score**	**pep_seq**	**pep_var_mod**
460.2179	918.4212	(19)	R.TYVSTIR.I	P (ST)
514.6891	1027.3637	19	R.**S**R**S**PEHR.R	2 P (ST)
628.2581	1254.5020	21	R.AR**S**R**S**PEHR.R	2 P (ST)
516.5908	1546.7504	(27)	R.SRSPLQPTSEIPR.R	P (ST)
774.3821	1546.7504	50	R.SRSPLQPTSEIPR.R	P (ST)
814.3652	1626.7168	(41)	R.**S**R**S**PLQPTSEIPR.R	2 P (ST)
543.2462	1626.7168	(21)	R.**S**R**S**PLQPTSEIPR.R	2 P (ST)
838.3506	1674.6886	(69)	R.**S**QAPNNTVTYESER.G	P (ST)
949.3937	1896.7738	(50)	R.APTLEQSSENEPEGSSR.T	P (ST)
959.9170	1917.8218	82	R.SR**S**QAPNNTVTYESER.G	P (ST)
640.2809	1917.8218	(29)	R.SRSQAPNNTVTYESER.G	P (ST)
992.4556	1982.8976	39	R.AER**S**R**S**PLQPTSEIPR.R	2 P (ST)
1027.4439	2052.8749	(48)	R.RAPTLEQSSENEPEGSSR.T	P (ST)
685.2986	2052.8749	(50)	R.RAPTLEQSSENEPEGSSR.T	P (ST)
752.6523	2254.9369	35	R.TRSRSQAPNNTVTYESER.G	2 P (ST)
771.0147	2310.0237	43	R.RAPTLEQSSENEPEGSSRTR.H	P (ST)
578.5131	2310.0237	(19)	R.RAPTLEQSSENEPEGSSRTR.H	P (ST)
**SRPK2 5 min**				
**pep_exp_mz**	**pep_exp_mr**	**pep_score**	**pep_seq**	**pep_var_mod**
473.2131	944.4117	35	GLFAA**S**GSR	P (ST)
516.5906	1546.7499	33	SRSPLQPTSEIPR	P (ST)
543.2464	1626.7174	18	**S**R**S**PLQPTSEIPR	2 P (ST)
553.516	2210.0349	24	ARAERSRSPLQPTSEIPR	2 P (ST)
578.5132	2310.0236	38	RAPTLEQSSENEPEGSSRTR	P (ST)
635.3174	1902.9303	38	AERSRSPLQPTSEIPR	P (ST)
640.2807	1917.8203	38	SRSQAPNNTVTYESER	P (ST)
661.9725	1982.8958	29	AERSRSPLQPTSEIPR	2 P (ST)
685.2986	2052.8741	51	RAPTLEQSSENEPEGSSR	P (ST)
718.9813	2153.922	31	APTLEQSSENEPEGSSRTR	P (ST)
750.8328	1499.651	20	AVSRTNPNSGDFR	P (ST)
752.6522	2254.9348	42	TRSRSQAPNNTVTYESER	2 P (ST)
771.0148	2310.0225	51	RAPTLEQSSENEPEGSSRTR	P (ST)
774.382	1546.7495	47	SRSPLQPTSEIPR	P (ST)
814.3648	1626.7151	25	**S**R**S**PLQPTSEIPR	2 P (ST)
814.3654	1626.7162	21	SRSPLQPTSEIPR	2 P (ST)
838.3527	1674.6909	57	**S**QAPNNTVTYESER	P (ST)
959.9179	1917.8213	69	SRSQAPNNTVTYESER	P (ST)
992.455	1982.8955	27	AERSRSPLQPTSEIPR	2 P (ST)
1027.4439	2052.8731	36	RAPTLEQSSENEPEGSSR	P (ST)
1106.0253	2210.036	18	ARAERSRSPLQPTSEIPR	2 P (ST)
**SRPK2 60 min**				
**pep_exp_mz**	**pep_exp_mr**	**pep_score**	**pep_seq**	**pep_var_mod**
460.2179	918.4212	31	TYVSTIR	P (ST)
473.213	944.4115	55	GLFAA**S**GSR	P (ST)
493.2267	984.4388	20	DSIASRTR	P (ST)
500.8911	1499.6514	25	AVSRTNPNSGDFR	P (ST)
516.5907	1546.7503	36	SRSPLQPTSEIPR	P (ST)
534.7452	1067.4759	21	NVERVE**S**R	P (ST)
534.9023	1601.6852	21	RLSVENMESSSQR	P (ST)
540.2346	1617.6819	35	RL**S**VENMESSSQR	O (M); P (ST)
543.2468	1626.7187	28	**S**R**S**PLQPTSEIPR	2 P (ST)
578.5133	2310.0241	50	RAPTLEQSSENEPEGSSRTR	P (ST)
604.2824	1809.8254	29	AERN**S**AEAVTEVPTTR	P (ST)
633.2654	1896.7743	42	APTLEQSSENEPEGSSR	P (ST)
640.2813	1917.8221	45	SRSQAPNNTVTYESER	P (ST)
652.8159	1303.6172	25	SPLQPTSEIPR	P (ST)
661.9733	1982.8982	30	AERSRSPLQPTSEIPR	2 P (ST)
685.2987	2052.8743	55	RAPTLEQSSENEPEGSSR	P (ST)
711.9543	2132.8412	33	RAPTLEQSSENEPEGSSR	2 P (ST)
718.9818	2153.9236	27	APTLEQSSENEPEGSSRTR	P (ST)
725.9974	2174.9703	23	TRSRSQAPNNTVTYESER	P (ST)
750.8325	1499.6505	32	AVSRTNPNSGDFR	P (ST)
752.6528	2254.9365	49	TRSRSQAPNNTVTYESER	2 P (ST)
771.0143	2310.021	31	RAPTLEQSSENEPEGSSRTR	P (ST)
771.0155	2310.0247	26	RAPTLEQSSENEPEGSSRTR	P (ST)

774.382	1546.7495	47	SRSPLQPTSEIPR	P (ST)
801.8499	1601.6853	70	RL**S**VENMESSSQR	P (ST)
809.8482	1617.6819	40	RL**S**VENMESSSQR	O (M); P (ST)
814.3657	1626.7169	45	**S**R**S**PLQPTSEIPR	2 P (ST)
838.3528	1674.6911	58	**S**QAPNNTVTYESER	P (ST)
854.3486	1706.6827	19	SRSPLQPTSEIPR	3 P (ST)
949.3945	1896.7744	58	APTLEQSSENEPEGSSR	P (ST)
959.9175	1917.8204	79	SR**S**QAPNNTVTYESER	P (ST)
992.4556	1982.8966	24	AERSRSPLQPTSEIPR	2 P (ST)
1027.4443	2052.8741	61	RAPTLEQSSENEPEGSSR	P (ST)

Phosphosite Localisation data obtained from Proteome Discoverer 1.4-SP1 –PhosphoRS3.1 or Proteome Discoverer 2.0-ptmRS. Underlined S T is interpretation of Mascot and MS2 data. Bold S T is a very good assignment, S T is used where identification is not certain, ?? means phosphorylation could be in any of the sites. pep_exp_mz: Observed or experimental m/z value, pep_exp_mr: Molecular mass calculated from experimental m/z value, pep_score: Mascot score for PSM (Peptide sequence match), pep_seq: Peptide sequence in 1 letter code, pep_var_mod: Variable modifications from all sources as list of names.

Table S5 (Related to Figure 5). Genes assigned to terms related to 'Neuron' identified by Gene Ontology analysis

GO: 0048699	GO: 0030182	GO: 0031175	GO: 0048666	GO: 0048812	GO: 0045664	GO: 0010975	GO: 0048667	GO: 0045665	GO: 0010976	GO: 0010977	GO: 0097485	GO: 0045666	GO: 0051402	GO: 1990138
Pcsk9	Pcsk9	Inpp5f	Inpp5f	Unc5a	Inpp5f	Inpp5f	Unc5a	Inpp5f	Rrn3	Inpp5f	Unc5a	Rrn3	Pcsk9	Apoe
Inpp5f	Inpp5f	Rrn3	Rrn3	Foxp1	Rrn3	Rrn3	Foxp1	Rap1gap	Rapgef1	Apoe	Foxp1	Rapgef1	Arrb1	Ddr1
Rrn3	Rrn3	Unc5a	Unc5a	Adarb1	Rapgef1	Rapgef1	Adarb1	Apoe	Abl2	Cit	Agrn	Abl2	Adarb1	Dpysl2
Ttbk1	Unc5a	Rapgef1	Rapgef1	Arhgef28	Rap1gap	Abl2	Arhgef28	Cd24a	Ap2a1	Crmp1	Ank3	Ap2a1	Egln3	Dclk1
Unc5a	Rapgef1	Foxp1	Foxp1	Abl2	Abl2	Agrn	Abl2	Cit	Apoe	Epha4	Boc	Apoe	Agrn	Bcl11a
Rapgef1	Foxp1	Mcf2	Mcf2	Agrn	Agrn	Ap2a1	Agrn	Crmp1	Bmp4	Bcl11a	Apbb2	Bmp4	Apoe	Fn1
Foxp1	Mcf2	Adarb1	Adarb1	Ank3	Ap2a1	Apoe	Ank3	Dll1	Camk2b	Ptk2	Bmpr1b	Camk2b	Hyou1	Impact
Mcf2	Ehmt2	Arhgef28	Arhgef28	Boc	Apoe	Bmp4	Boc	Epha4	Cd24a	Gfap	Runx3	Cd24a	Cacna1a	Arhgap4
Ehmt2	Rap1gap	Abl2	Abl2	Apbb2	Bmp4	Cacna1a	Apbb2	Bcl11a	Cnr1	H2-D1	Crmp1	Cnr1	Casp3	Mag
Rap1gap	Adarb1	Agrn	Agrn	Apoe	Cacna1a	Camk2b	Apoe	Ptk2	Cobl	H2-K1	Dpysl2	Cobl	Cit	Map1b
Adarb1	Arhgef28	Ank3	Ank3	Bmpr1b	Camk2b	Cd24a	Atp2b2	Gfap	Crabp2	Lgals1	Enah	Crabp2	Coro1a	Myo5b
Arhgef28	Abl2	Boc	Boc	Cacna1a	Cd24a	Cit	Bmpr1b	H2-D1	Dlg4	Lrp1	Epha4	Dlg4	Fgf8	Ntn1
Abl2	Agrn	Ap2a1	Ap2a1	Ddr1	Cit	Cnr1	Cacna1a	H2-K1	Eef2k	Arhgap4	Epha8	Eef2k	Fzd9	Ntrk3
Agrn	Ank3	Apbb2	Apbb2	Camk2a	Cnr1	Cobl	Camk2a	Jag1	Enc1	Mag	Ephb3	Enc1	Gclm	Pak1
Ank3	Boc	Apoe	Apoe	Camk2b	Cobl	Crabp2	Camk2b	Lgals1	Epha4	Ngfr	Ext1	Epha4	Glp1r	Pou4f2
Boc	Ap2a1	Atp2b2	Atp2b2	Runx3	Crabp2	Crmp1	Runx3	Lrp1	Bcl11a	Ntn1	Fgf8	Bcl11a	Grik5	Ptprs
Ap2a1	Apbb2	Bmp4	Prdm1	Cit	Crmp1	Dpysl2	Cit	Arhgap4	Fgfr1	Pmp22	Flot1	Fgfr1	Lrp1	Sema3f
Apbb2	Apoe	Bmpr1b	Bmp4	Cobl	Dpysl2	Dlg4	Cobl	Mag	Fn1	Ptprs	Gap43	Fn1	Mag	Sema4a
Apoe	Atp2b2	Cacna1a	Bmpr1b	Crabp2	Dlg4	Eef2k	Crabp2	Ngfr	Ikbkb	Stmn2	Gbx2	Ikbkb	Mt3	Sema6b
Atp2b2	Prdm1	Ddr1	Cacna1a	Crmp1	Dll1	Enc1	Crmp1	Notch1	Map1b	Sema3f	Foxd1	Impact	Mybl2	Sema6c
Prdm1	Bmp4	Camk2a	Ddr1	Dpysl2	Eef2k	Epha4	Dpysl2	Ntn1	Myo5b	Sema4a	Matn2	Map1b	Nf1	Sema7a
Bmp4	Bmpr1b	Camk2b	Camk2a	Dclk1	Enc1	Ephb3	Dclk1	Pmp22	Neu1	Sema6b	Ngfr	Myo5b	Ngfr	Tiam1
Bmpr1b	Cacna1a	Casp3	Camk2b	Dlg4	Epha4	Bcl11a	Dlg4	Pou4f2	Nf1	Sema6c	Ntn1	Neu1	Prkcg	Nrn1l
Tspo	Ddr1	Runx3	Casp3	Eef2k	Ephb3	Ptk2	Eef2k	Ptprs	Ngfr	Sema7a	Etv4	Neurod1	Prkci	Twf2
Cacna1a	Camk2a	Cd24a	Runx3	Enah	Bcl11a	Fgfr1	Enah	Stmn2	Ntn1	Thy1	Pou4f2	Nf1	Lgmn	Cpne5
Ddr1	Camk2b	Cit	Cd24a	Epha4	Ptk2	Fn1	Epha4	Sema3f	Ntrk3	Tsc2	Ptch1	Ngfr	Sod1	Sema4g
Camk2a	Casp3	Cnr1	Cit	Epha8	Fgfr1	Gfap	Epha8	Sema4a	P2ry2	Dpysl3	Reln	Ntn1	Sod2	Cpne1
Camk2b	Runx3	Cobl	Cnr1	Ephb3	Fn1	H2-D1	Ephb3	Sema6b	Pak1	Vim	Robo1	Ntrk3	Srpk2	Dbn1
Casp3	Cd24a	Crabp2	Cobl	Bcl11a	Gfap	H2-K1	Bcl11a	Sema6c	Palm	Klk8	Sema3f	P2ry2	Hdac4	Map3k13
Runx3	Cit	Crmp1	Crabp2	Ext1	H2-D1	Ikbkb	Ext1	Sema7a	Prkci	Sema4g	Sema4a	Pak1	Stxbp1	Cyfip2
Cd24a	Cnr1	Dpysl2	Crmp1	Ptk2	H2-K1	Kif13b	Ptk2	Cntn2	Pou4f2	Rtn4rl2	Sema6b	Palm	Trp73	
Cdh1	Cobl	Dclk1	Dpysl2	Fgf8	Ikbkb	Lgals1	Fgf8	Thy1	Reln	Lrig2	Sema6c	Prkci	Vegfb	
Celsr1	Crabp2	Dlg4	Dclk1	Flot1	Impact	Lrp1	Flot1	Trp73	Robo1	Rap1gap2	Sema7a	Pou4f2	Wfs1	
Cit	Crmp1	Eef2k	Dlg4	Fn1	Jag1	Arhgap4	Fn1	Tsc2	Stmn2	Carm1	Cntn2	Rara	Axl	
Cnr1	Dpysl2	Enah	Eef2k	Gap43	Kif13b	Mag	Gap43	Dpysl3	Sema7a	Ctsz	Rnf165	Reln	Vstm2l	
Cobl	Dclk1	Enc1	Enah	Gbx2	Lgals1	Map1b	Gbx2	Vim	Skil	Itm2c	Plxnb1	Robo1	Ctsz	
Crabp2	Dlg4	Epha4	Enc1	Gja1	Lrp1	Myo5b	Foxd1	Klk8	Creb3l2	Nfatc4	Sema4g	Stmn2	Fam162a	
Crmp1	Dll1	Epha8	Epha4	Foxd1	Rac3	Neu1	Kif13b	Sema4g	Plk5		Pla2g10	Sema7a	Pigt	
Dpysl2	Eef2k	Ephb3	Epha8	Impact	Arhgap4	Nf1	Stmn1	Rtn4rl2	Ppp2r5d		Vstm2l	Skil	Trim2	
Dclk1	Enah	Bcl11a	Ephb3	Kif13b	Mag	Ngfr	Arhgap4	Lrig2	Tiam1		Nrcam	Sox11		
Dlg4	Enc1	Ext1	Bcl11a	Stmn1	Map1b	Ntn1	Mag	Rap1gap2	Tsc2		Ccdc141	Creb3l2		
Dll1	Epha2	Ptk2	Ext1	Lifr	Myo5b	Ntrk3	Matn2	Zhx2	Dpysl3		Bcl11b	Plk5		
Dll3	Epha4	Fgf8	Ptk2	Arhgap4	Neu1	P2ry2	Map1a	Carm1	Tbc1d24		Cyfip2	Ppp2r5d		
Eef2k	Epha8	Fgfr1	Fgf8	Mag	Neurod1	Pak1	Map1b	Ctsz	Zmynd8		Vangl2	Tiam1		
Enah	Ephb3	Flot1	Fgfr1	Matn2	Nf1	Palm	Myo5b	Itm2c	Plxnb1			Tsc2		
Enc1	Bcl11a	Fn1	Flot1	Map1a	Ngfr	Prkci	Ngfr	Casz1	Pacsin1			Dpysl3		
Epha2	Ext1	Gap43	Fn1	Map1b	Mycn	Pmp22	Notch1	Nfatc4	Twf2			Tbc1d24		
Epha4	Ptk2	Gbx2	Gap43	Myo5b	Notch1	Pou4f2	Ntn1		Cpne5			Zmynd8		
Epha8	Fgf8	Gfap	Gbx2	Ngfr	Ntn1	Ptprs	Tbc1d24		Ankrd27			Plxnb1		
Ephb3	Fgfr1	Gfra1	Gfap	Notch1	Ntrk3	Reln	Ntrk3		Mapk6			Pacsin1		
Bcl11a	Flot1	Gja1	Gfra1	Ntn1	P2ry2	Robo1	Pak1		Dbn1			Twf2		
Ext1	Fn1	H2-D1	Gja1	Tbc1d24	Pak1	Stmn2	Etv4		Shank3			Cpne5		
Ptk2	Gap43	H2-K1	Gnat2	Ntrk3	Palm	Sema3f	Pmp22		Actr2			Ankrd27		
Fgf8	Gbx2	Foxd1	H2-D1	Pak1	Prkci	Sema4a	Pou4f2		Dab2ip			Cpne1		
Fgfr1	Gfap	Ikbkb	H2-K1	Etv4	Pmp22	Sema6b	Ptch1		Ptk7			Mapk6		
Flot1	Gfra1	Impact	Foxd1	Pmp22	Pou4f2	Sema6c	Ptprs		Map3k13			Dbn1		
Fn1	Gja1	Atcay	Ikbkb	Pou4f2	Ptprs	Sema7a	Reln					Shank3		
Fzd9	Gnat2	Kif13b	Impact	Ptch1	Rara	Sfrp1	Robo1					Actr2		
Gap43	Nkx6-2	Stmn1	Atcay	Ptprs	Reln	Skil	Sema3f					Dab2ip		
Gas6	H2-D1	Lgals1	Kif13b	Rac2	Robo1	Creb3l2	Sema4a					Ptk7		
Gbx2	H2-K1	Lifr	Stmn1	Reln	Stmn2	Cntn2	Sema6b					Map3k13		
Gfap	Foxd1	Lrp1	Lgals1	Robo1	Sema3f	Plk5	Sema6c					Kdm4c		
Gfra1	Ikbkb	Rac3	Lifr	Sema3f	Sema4a	Ppp2r5d	Sema7a					Lin28a		
Gja1	Impact	Arhgap4	Lrp1	Sema4a	Sema6b	Thy1	Skil							
Gnat2	Jag1	Mag	Rac3	Sema6b	Sema6c	Tiam1	Sod1							
Nkx6-2	Atcay	Matn2	Arhgap4	Sema6c	Sema7a	Tsc2	Stxbp1							
H2-D1	Kif13b	Mmp2	Mag	Sema7a	Sfrp1	Dpysl3	Cntn2							
H2-K1	Stmn1	Map1a	Matn2	Skil	Skil	Vim	Thy1							
Hes2	Lgals1	Map1b	Mmp2	Stxbp1	Sox11	Tbc1d24	Tiam1							
Foxd1	Lifr	Myo5b	Map1a	Cntn2	Creb3l2	Zmynd8	Tsc2							
Ikbkb	Lrp1	Neu1	Map1b	Thy1	Cntn2	Arhgap33	Uchl1							
Il6st	Rac3	Nf1	Myo5b	Tiam1	Plk5	Plxnb1	Vim							
Impact	Mcoln3	Ngfr	Neu1	Tsc2	Ppp2r5d	Pacsin1	Tbc1d24							
Jag1	Arhgap4	Notch1	Neurod1	Uchl1	Thy1	Twf2	Rnf165							
Atcay	Mag	Ntn1	Nf1	Vim	Tiam1	Cpne5	Arhgap33							
Kif13b	Matn2	Tbc1d24	Ngfr	Tbc1d24	Trp73	Ankrd27	Plxnb1							
Stmn1	Mmp2	Ntrk3	Notch1	Rnf165	Tsc2	Klk8	Twf2							
Ldlr	Map1a	P2ry2	Ntn1	Arhgap33	Dpysl3	Sema4g	Ankrd27							
Lef1	Map1b	Pak1	Tbc1d24	Nrn1l	Vim	Rtn4rl2	Sema4g							
Lgals1	Myo5b	Palm	Ntrk3	Plxnb1	Tbc1d24	Lrig2	Pla2g10							
Lifr	Neu1	Etv4	P2ry2	Pacsin1	Zmynd8	Prex1	Vstm2l							
Lrp1	Neurod1	Prkci	Pak1	Twf2	Arhgap33	Nrcam	Nrcam							
Rac3	Nf1	Pmp22	Palm	Cpne5	Plxnb1	Rap1gap2	Adcy1							
Mcoln3	Ngfr	Pou4f2	Etv4	Kif20b	Pacsin1	Mapk6	Ccdc141							
Arhgap4	Mycn	Ptch1	Prkci	Nyap1	Twf2	Srcin1	Srcin1							
Mag	Notch1	Ptprs	Pmp22	Ankrd27	Cpne5	Dbn1	Dbn1							
Matn2	Ntn1	Rac2	Pou4f2	Klk8	Ankrd27	Shank3	Bcl11b							
Mmp2	Tbc1d24	Reln	Ptch1	Sema4g	Klk8	Carm1	Shank3							
Mt3	Ntrk3	Robo1	Ptprs	Pla2g10	Sema4g	Ctsz	Actr2							
Map1a	P2ry2	Stmn2	Rac2	Cpne1	Cpne1	Itm2c	Map3k13							
Map1b	Pak1	Sema3f	Reln	Vstm2l	Rtn4rl2	Actr2	Nfatc4							
Myo5b	Palm	Sema4a	Robo1	Nrcam	Lrig2	Dab2ip	Cyfip2							
Neu1	Etv4	Sema6b	Rpgr	Adcy1	Prex1	Ptk7	Vangl2							
Neurod1	Prkci	Sema6c	Stmn2	Ccdc141	Nrcam	Map3k13	Ophn1							
Nf1	Pmp22	Sema7a	Sema3f	Srcin1	Rap1gap2	Nfatc4								
Ngfr	Pou4f2	Sfrp1	Sema4a	Dbn1	Zhx2									
Mycn	Prom1	Skil	Sema6b	Bcl11b	Mapk6									
Notch1	Ptch1	Sod1	Sema6c	Shank3	Srcin1									
Ntn1	Ptprs	Creb3l2	Sema7a	Actr2	Dbn1									
Tbc1d24	Rac2	Stx3	Sfrp1	Dab2ip	Bcl11b									
Ntrk3	Rara	Stxbp1	Skil	Map3k13	Shank3									
P2ry2	Reln	Cntn2	Sod1	Nfatc4	Carm1									
Pak1	Robo1	Plk5	Sod2	Cyfip2	Ctsz									
Palm	Rpgr	Ppp2r5d	Creb3l2	Vangl2	Itm2c									
Etv4	Stmn2	Thy1	Stx3		Actr2									
Prkci	Sema3f	Tiam1	Stxbp1		Dab2ip									
Pmp22	Sema4a	Tsc2	Cntn2		Casz1									
Pou4f2	Sema6b	Tulp1	Plk5		Ptk7									
Prom1	Sema6c	Uchl1	Ppp2r5d		Map3k13									
Ptch1	Sema7a	Dpysl3	Thy1		Nfatc4									
Ptprs	Sfrp1	Vim	Tiam1		Kdm4c									
Rac2	Skil	Tbc1d24	Trp73		Lin28a									
Rara	Sod1	Rnf165	Tsc2											
Reln	Sod2	Zmynd8	Tulp1											
Robo1	Sox11	Arhgap33	Uchl1											
Rpgr	Sox4	Nrn1l	Dpysl3											
Stmn2	Stat3	Plxnb1	Vim											

Sema3f	Creb3l2	Pacsin1	Tbc1d24											
Sema4a	Stx3	Twf2	Rnf165											
Sema6b	Stxbp1	Cpne5	Zmynd8											
Sema6c	Cntn2	Kif20b	Arhgap33											
Sema7a	Plk5	Nyap1	Nrn1l											
Sfrp1	Ppp2r5d	Ankrd27	Plxnb1											
Skil	Tgm3	Klk8	C1ql1											
Sod1	Thy1	Sema4g	Pacsin1											
Sod2	Tiam1	Pla2g10	Twf2											
Sox11	Trp73	Cpne1	Cpne5											
Sox4	Tsc2	Rtn4rl2	Kif20b											
Stat3	Tulp1	Lrig2	Nyap1											
Creb3l2	Tulp3	Micall1	Ankrd27											
Stx3	Uchl1	Prex1	Klk8											
Stxbp1	Dpysl3	Vstm2l	Sema4g											
Cntn2	Vim	Nrcam	Pla2g10											
Nav1	Wnt5b	Rap1gap2	Cpne1											
Plk5	Wnt6	Adcy1	Rtn4rl2											
Ppp2r5d	Tbc1d24	Mapk6	Lrig2											
Tgfb1	Rnf165	Nptx2	Micall1											
Tgm3	Zmynd8	Ccdc141	Naglu											
Thy1	Arhgap33	Srcin1	Prex1											
Tiam1	Nrn1l	Dbn1	Vstm2l											
Trp73	Plxnb1	Slc12a5	Nrcam											
Tsc2	C1ql1	Bcl11b	Cngb1											
Tulp1	Pacsin1	Shank3	Rap1gap2											
Tulp3	Twf2	Carm1	Adcy1											
Uchl1	Cpne5	Trappc4	Mapk6											
Dpysl3	Kif20b	Ctsz	Nptx2											
Vim	Nyap1	Itm2c	Ccdc141											
Wnt5b	Ankrd27	Actr2	Srcin1											
Wnt6	Klk8	Cthrc1	Dbn1											
Tbc1d24	Sema4g	Dab2ip	Slc12a5											
Rnf165	Pla2g10	Ptk7	Bcl11b											
Zmynd8	Cpne1	Map3k13	Shank3											
Arhgap33	Rtn4rl2	Nfatc4	Carm1											
Nrn1l	Lrig2	Manf	Trappc4											
Plxnb1	Chd5	Cyfip2	Ctsz											
C1ql1	Micall1	Vangl2	Itm2c											
Pacsin1	Naglu	Clmn	Actr2											
Twf2	Prex1	Ophn1	Cthrc1											
Cpne5	Vstm2l	Efhd1	Dab2ip											
Kif20b	Nrcam		Ptk7											
Nyap1	Cngb1		Map3k13											
Ankrd27	Rap1gap2		Nfatc4											
Klk8	Zhx2		Manf											
Axl	Adcy1		Cyfip2											
Sema4g	Mapk6		Kcnip2											
Pla2g10	Nptx2		Vangl2											
Cpne1	Ccdc141		Clmn											
Rtn4rl2	Srcin1		Ophn1											
Ell3	Dbn1		Efhd1											
Lrig2	Slc12a5													
Chd5	Bcl11b													
Idh2	Shank3													
Micall1	Carm1													
Naglu	Trappc4													
Prex1	Ctsz													
Vstm2l	Itm2c													
Nrcam	Actr2													
Chd7	Cthrc1													
Cngb1	Dab2ip													
Rap1gap2	Casz1													
Zhx2	Ptk7													
Adcy1	Map3k13													
Mapk6	Gpc2													
Nptx2	Nfatc4													
Ccdc141	Manf													
Srcin1	Kdm4c													
Dbn1	Cyfip2													
Plag1	Pigt													
Slc12a5	Kcnip2													
Bcl11b	Lin28a													
Shank3	Smarca1													
Carm1	Vangl2													
Trappc4	Clmn													
Ctsz	Ophn1													
Itm2c	Efhd1													
Actr2														
Smarcd3														
Cthrc1														
Dab2ip														
Casz1														
Ptk7														
Map3k13														
Zcchc24														
Gpc2														
Nfatc4														
Manf														
Kdm4c														
Cyfip2														
Pigt														
Kcnip2														
Lin28a														
Hes7														
Smarca1														
Vangl2														
Clmn														
Ophn1														
Efhd1														

Table S6 (Related to Figure 5). Genes assigned to terms related to 'Neural' identified by Gene Ontology analysis

GO: 0021915	GO: 0001841	GO: 0014020	GO: 0001843	GO: 0090177	GO: 0061351	GO: 0061076	GO: 0001840	GO: 2000177	GO: 0090179	GO: 0090178	GO: 0003407	GO: 0001839	GO: 0014033	GO: 2000178
Abl2	Abl2	Abl2	Abl2	Celsr1	Rapgef1	Pou4f2	Fgf8	Rapgef1	Celsr1	Celsr1	Gnat2	Fgf8	Anxa6	Rapgef1
Bmp4	Bmp4	Bmp4	Bmp4	Sfrp1	Cd24a	Casz1	Ptch1	Cd24a	Sfrp1	Sfrp1	Neurod1	T	Bmp4	Cd24a
Celsr1	Celsr1	Celsr1	Celsr1	Cthrc1	Fgf8		T	Fzd9	Ptk7	Ptk7	Pou4f2	Vangl2	Fn1	Nf1
Cobl	Cobl	Cobl	Cobl	Ptk7	Fgfr1		Vangl2	Gli1	Vangl2	Vangl2	Prom1		Gbx2	Spint2
Enah	Enah	Enah	Enah	Vangl2	Fzd9			Nf1			Megf11		Jag1	Tgfb1
Epha2	Ptch1	Ptch1	Ptch1		Gbx2			Ngfr			Thy1		Lama5	
Fgf8	Rara	Rara	Rara		Kif1a			Notch1			Rab11fip4		Sema3f	
Gbx2	Sfrp1	Sfrp1	Sfrp1		Lef1			Spint2			Smarcd3		Sema4a	
Nf1	Sox11	Spint2	Spint2		Nf1			Tgfb1			Casz1		Sema6b	
Notch1	Sox4	T	T		Ngfr			Ell3					Sema6c	
Ptch1	Spint2	Tead2	Tead2		Notch1			Ctsz					Sema7a	
Rara	T	Tgfb1	Tgfb1		Ncor2			Smarcd3					Sfrp1	
Sfrp1	Tead2	Tsc2	Tsc2		Spint2			Smarca1					Sox11	
Sox11	Tgfb1	Tulp3	Tulp3		Tgfb1								Mapk3	
Sox4	Tsc2	Kif20b	Kif20b		Ell3								Sema4g	
Spint2	Tulp3	Luzp1	Dlc1		Tacc1									
T	Kif20b	Dlc1	Med12		Dbn1									
Tcf7	Luzp1	Med12	Cthrc1		Ctsz									
Tead2	Dlc1	Cthrc1	Ptk7		Smarcd3									
Itpk1	Med12	Ptk7	Vangl2		Smarca1									
Tgfb1	Cthrc1	Vangl2												
Tsc2	Ptk7													
Tulp3	Vangl2													
Kif20b														
Luzp1														
Dlc1														
Med12														
Cthrc1														
Atp6ap2														
Ptk7														
Vangl2														

Table S7 (Related to Figure 5). Disease association of RNF12-regulated genes from the neural crest cell differentiation GO term (GO:0014033).

Protein name	Gene name	Neural crest function	Craniofacial defects association
Annexin A6	Anxa6	Modulates chick cranial neural crest cell emigration (Wu and Taneyhill, 2012)	Associated to Treacher Collins syndrome (Dixon et al., 1994)
Bone morphogenetic protein 4	Bmp4	Regulates neural crest migration and differentiation (Li et al., 2018; Sela-Donenfeld and Kalcheim, 1999; Zhu et al., 2019)<br>Regulates tooth morphogenesis (Jia et al., 2016; Shin et al., 2012)	Associated to Non-syndromic cleft lip (Chen et al., 2012, 2014)
Fibronectin	Fn1	Participate in differentiation of NC cells into vascular smooth muscle cells (Wang and Astrof, 2016)	
Homeobox protein GBX-2	Gbx2	Required for pharyngeal arch and cardiovascular development (Byrd and Meyers, 2005)<br>Participates in neural crest induction (Li et al., 2009)	Associated to craniofacial microsomia (Zhang et al., 2016)
Protein jagged-1	Jag1	Induces neural crest stem cell self-renewal and osteoblast differentiation (Kamalakar et al., 2019; Nikopoulos et al., 2007)<br>Regulates face development (Zuniga et al., 2010)<br>Regulates specification of the coronal suture (Yen et al., 2010)<br>Regulates maxillary ossification (Hill et al., 2014)<br>Regulates bone patterning of middle ear (Teng et al., 2017)	Deleted in Alagille Syndrome (Humphreys et al., 2012; Micaglio et al., 2019; Pilia et al., 1999)
Laminin subunit alpha-5	Lama5	Regulates neural crest cell migration (Coles et al., 2006)<br>Required for tooth development (Fukumoto et al., 2006)	
Semaphorin-3F	Sema3f	Regulates cranial neural crest cell migration (York et al., 2018; Yu and Moens, 2005)<br>Regulates trunk neural crest migration (Gammill et al., 2006)	
Semaphorin-4A	Sema4a	Phylogenetic-based functional annotation	
Semaphorin-4G	Sema4g	Phylogenetic-based functional annotation	
Semaphorin-6B	Sema6b	Patterns cardiac neural crest migration (Toyofuku et al., 2008)	
Semaphorin-6C	Sema6c	Phylogenetic-based functional annotation	
Semaphorin-7A	Sema7a	Expressed in cranial and trunk neural crest cells (Bao and Jin, 2006)	Associated to craniofacial microsomia (Zhang et al., 2016)
Secreted frizzled-related protein 1	Sfrp1	Expressed in migrating neural crest cells (Duprez et al., 1999)<br>Regulates Periodontal Mineral Homeostasis (Gopinathan et al., 2019)	
Transcription factor SOX-11	Sox11	Expressed in neural crest and derivatives (Hargrave et al., 1997; Sock et al., 2004)<br>Ablation generates Clefting of the Secondary Palate (Huang et al., 2016)<br>Linked to cranial vault shape in humans (Roosenboom et al., 2018)	
Mitogen-activated protein kinase 3	Mapk3, Erk1	Expressed in neural crest and derivatives (Parada et al., 2015)	

Table S8 (Related to STAR Methods). Primer and Oligonucleotide sequences

Oligonucleotides		
qRT-PCR primers	Forward (5'-3')	Reverse (5'-3')
FoxP1 ex15-16	CACGTGGAAGAATG CAGTGCG	N/A
FoxP1 ex15-16b	CACGTGGAAGGGTG CCATTC	N/A
FoxP1 ex17	N/A	TGAGAGGTGTGCAG TAGGCG
Gapdh	CTCGTCCCGTAGAC AAAA	TGAATTTGCCGTGA GTGG
Ntn1	CGCAACTGTACCAG TGACCTCT	TTGCGGCAGTAGAT GAGGACGA
Dll1	ACCAAGTGCCAGTC ACAGAG	TCCATCTTACACCTC AGTCGC
Kif1a	CACCACTATTGTCAA CCCCAAA	CCCCAATGTCCCTG TAGACCT
Gfap	CAATGCTGGCTTCA AGGAGACACG	TCAGTTCAGCTGCC AGCGCCT
Unc5a	GTCTGGTGTGTGAC TGTAGGCA	CCGAGCATGGAGGT TGCAGTTG
Primers for genomic DNA sequencing	Forward (5'-3')	Reverse (5'-3')
Srpk1 (KO)	TGCACTAACAGGCA CTGTCAGG	CAGGCTCTGGTGAG ACCTAGC
Srpk2 (KO)	GTAGAGTAACTGTC TCTGTAAACTTGTGT ACTG	CTATAAAGCTGGAC CAGGAGAGGC
Zfp42/Rex1 (KO)	TCACCATGGGCTCT CGTATTGG	AGAAGACTCGAGAA GGGAACTCG
Rlim 4xSA/y (KI)	AGAGCAAGAGCTGA AAGAGCCAGGGCAC CTTTACAGCCAACA AGTGAAATTCC	GGTTCTACGATGCT CTGGGGCCCGGGC TCTTGCCCTCCTCT GAGCACG
Rlim ΔSR-motif/y (KI)	CCTTTACAGCCAAC AAGTGAAATTCC	ACGCGTAGTCGGCA CTTCTG
Rlim W576Y/y (KI), W576Y/y (KI), WT/y (KI)	ACAGCCTCAGCATC TTCTAGAGC	AGTGCATTGGAAAA GTACTGAGCC
gRNA sequences for CRISPR/Cas9	Sense gRNA (5'-3')	Antisense gRNA (5'-3')
Srpk1 (KO)	GAGCAGGAGGAGG AGATTCT	GCGGAGTGGGGTG CAGAGCCT
Srpk2 (KO)	GATTGATGACTTCAA GATCTC	GATGTCTTTGTTTGG GTCACT
Zfp42/Rex1 (KO)	GAGGAAGATGGCTT CCCTGA	GAATCTCACTTTCAT CCCGGA
Rlim 4xSA/y (KI)	GAGTTCGTCCTGGA GAATAC	GTACTTGAAGATCA AGAACTA
Rlim ΔSR-motif/y (KI)	GAAGCCGGAGCCCA GAGCAT	GCTCCTCTGAGCTC TGGTGGT
Rlim W576Y/y (KI), W576Y/y (KI), WT/y (KI)	GCAGGGCAGTCTTA TCTTCT	GTGGAATTCTCAGA CAACCAG
