# Supplementary material for: Functional Diversification of SRSF Protein Kinase to Control Ubiquitin-Dependent Neurodevelopmental Signaling
Source: Dev Cell. 2020 Dec 7;55(5):629–647.e7. doi: 10.1016/j.devcel.2020.09.025 (PMC7725506; doi:10.1016/j.devcel.2020.09.025)
Supplement: Document S2. Article plus Supplemental Information [file mmc2.pdf]

Article

Developmental Cell

Functional Diversification of SRSF Protein Kinase to Control Ubiquitin-Dependent Neurodevelopmental Signaling

Graphical Abstract

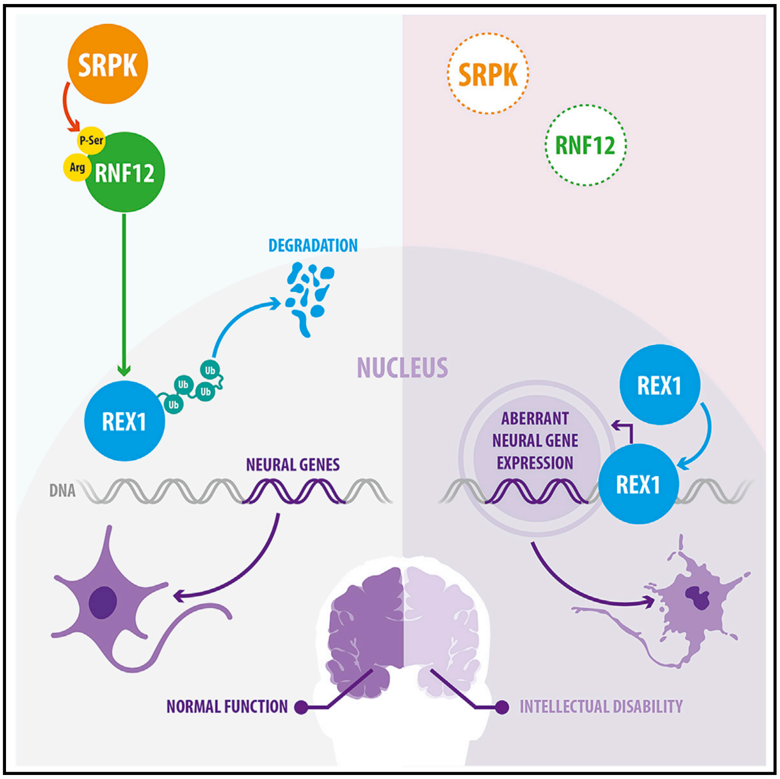

Highlights

- SRPK has acquired a developmental function regulating RNF12
- RNF12 phosphorylation by SRPK promotes E3 ligase activity and nuclear anchoring
- SRPK-RNF12 signaling to the REX1 transcription factor controls neural genes
- This signaling network is disrupted in neurodevelopmental disorders

Authors

Francisco Bustos, Anna Segarra-Fas, Gino Nardocci, ..., Renata F. Soares, Martin Montecino, Greg M. Findlay

Correspondence

g.m.findlay@dundee.ac.uk

In Brief

Bustos et al. show that SRPK splicing factor kinase has acquired a developmental function—phosphorylating the RNF12 E3 ubiquitin ligase to promote degradation of the transcription factor, REX1. This signaling pathway regulates a neurodevelopmental gene expression program and is mutated in patients with neurodevelopmental disorders.

Bustos et al., 2020, Developmental Cell 55, 629–647
December 7, 2020 © 2020 The Authors. Published by Elsevier Inc.
https://doi.org/10.1016/j.devcel.2020.09.025

CellPress

Developmental Cell

CellPress
OPEN ACCESS

Article

Functional Diversification of SRSF Protein Kinase to Control Ubiquitin-Dependent Neurodevelopmental Signaling

Francisco Bustos,[1] Anna Segarra-Fas,[1] Gino Nardocci,[2] Andrew Cassidy,[3] Odetta Antico,[1] Lindsay Davidson,[4] Lennart Brandenburg,[1] Thomas J. Macartney,[1] Rachel Toth,[1] C. James Hastie,[1] Jennifer Moran,[1] Robert Gourlay,[1] Joby Varghese,[1] Renata F. Soares,[1] Martin Montecino,[2] and Greg M. Findlay[1,5,*]

[1]The MRC Protein Phosphorylation and Ubiquitylation Unit, School of Life Sciences, the University of Dundee, Dundee DD1 5EH, UK

[2]Institute of Biomedical Sciences and FONDAP Center for Genome Regulation, Universidad Andrés Bello, Santiago, Chile

[3]Tayside Centre for Genomic Analysis, School of Medicine, University of Dundee, Dundee DD1 9SY, UK

[4]School of Life Sciences, The University of Dundee, Dundee DD1 5EH, UK

[5]Lead Contact

*Correspondence: g.m.findlay@dundee.ac.uk

https://doi.org/10.1016/j.devcel.2020.09.025

SUMMARY

Conserved protein kinases with core cellular functions have been frequently redeployed during metazoan evolution to regulate specialized developmental processes. The Ser/Arg (SR)-rich splicing factor (SRSF) protein kinase (SRPK), which is implicated in splicing regulation, is one such conserved eukaryotic kinase. Surprisingly, we show that SRPK has acquired the capacity to control a neurodevelopmental ubiquitin signaling pathway. In mammalian embryonic stem cells and cultured neurons, SRPK phosphorylates Ser-Arg motifs in RNF12/RLIM, a key developmental E3 ubiquitin ligase that is mutated in an intellectual disability syndrome. Processive phosphorylation by SRPK stimulates RNF12-dependent ubiquitylation of nuclear transcription factor substrates, thereby acting to restrain a neural gene expression program that is aberrantly expressed in intellectual disability. SRPK family genes are also mutated in intellectual disability disorders, and patient-derived SRPK point mutations impair RNF12 phosphorylation. Our data reveal unappreciated functional diversification of SRPK to regulate ubiquitin signaling that ensures correct regulation of neurodevelopmental gene expression.

INTRODUCTION

Signal transduction by protein kinases controls all aspects of eukaryotic biology (Cohen, 2002), from metabolism to complex developmental programs. As such, protein kinases involved in core eukaryotic processes have been redeployed during metazoan evolution to regulate specialized processes required for multicellular life. This is illustrated by acquisition of increasingly complex roles of the mitogen activated protein kinase (MAPK) signaling pathway from yeast to metazoans. In yeast, MAPK signaling controls simple unicellular functions, such as sensing mating pheromones and environmental stress (Chen and Thorner, 2007), while metazoan MAPK signaling has acquired the ability to regulate complex multicellular processes, including lineage-specific differentiation (Cowley et al., 1994; Traverse et al., 1992). Other highly conserved protein kinases may have undergone similar ‘‘functional diversification’’ to acquire new functions, thereby facilitating metazoan evolution.

In principle, functional diversification of protein kinases can be achieved via several non-mutually exclusive mechanisms: (1) evolutionary wiring of protein kinase pathways to newly evolved cell-cell communication systems that control metazoan biology, such as receptor tyrosine kinases (Lim and Pawson, 2010), (2) evolution of new kinase-substrate relationships, and (3) evolution of specific kinase activity or expression profiles that differ according to developmental time and tissue context. These mechanisms individually or in combination have the capacity to drive functional diversification, enabling highly conserved eukaryotic protein kinases to evolve novel functions in the control of key metazoan processes.

The Ser-Arg rich splicing factor (SRSF) protein kinase (SRPK) family represent a prominent case study for functional diversification, as they perform core functions in mRNA splicing regulation that are thought to be conserved throughout eukaryotes (Dagher and Fu, 2001; Gui et al., 1994b; Siebel et al., 1999; Yeakley et al., 1999). SRPKs phosphorylate SRSFs, modulating their subcellular localization and regulating spliceosome assembly (Cao et al., 1997; Koizumi et al., 1999; Mathew et al., 2008; Xiao and Manley, 1997). Few non-splicing functions of SRPKs have been reported (Gou et al., 2020; Hong et al., 2012; Wang et al., 2017), and it remains unclear whether SRPKs have evolved further regulatory roles in metazoans. However, SRPK family

© 2020 The Authors. Published by Elsevier Inc.
This is an open access article under the CC BY license (http://creativecommons.org/licenses/by/4.0/).

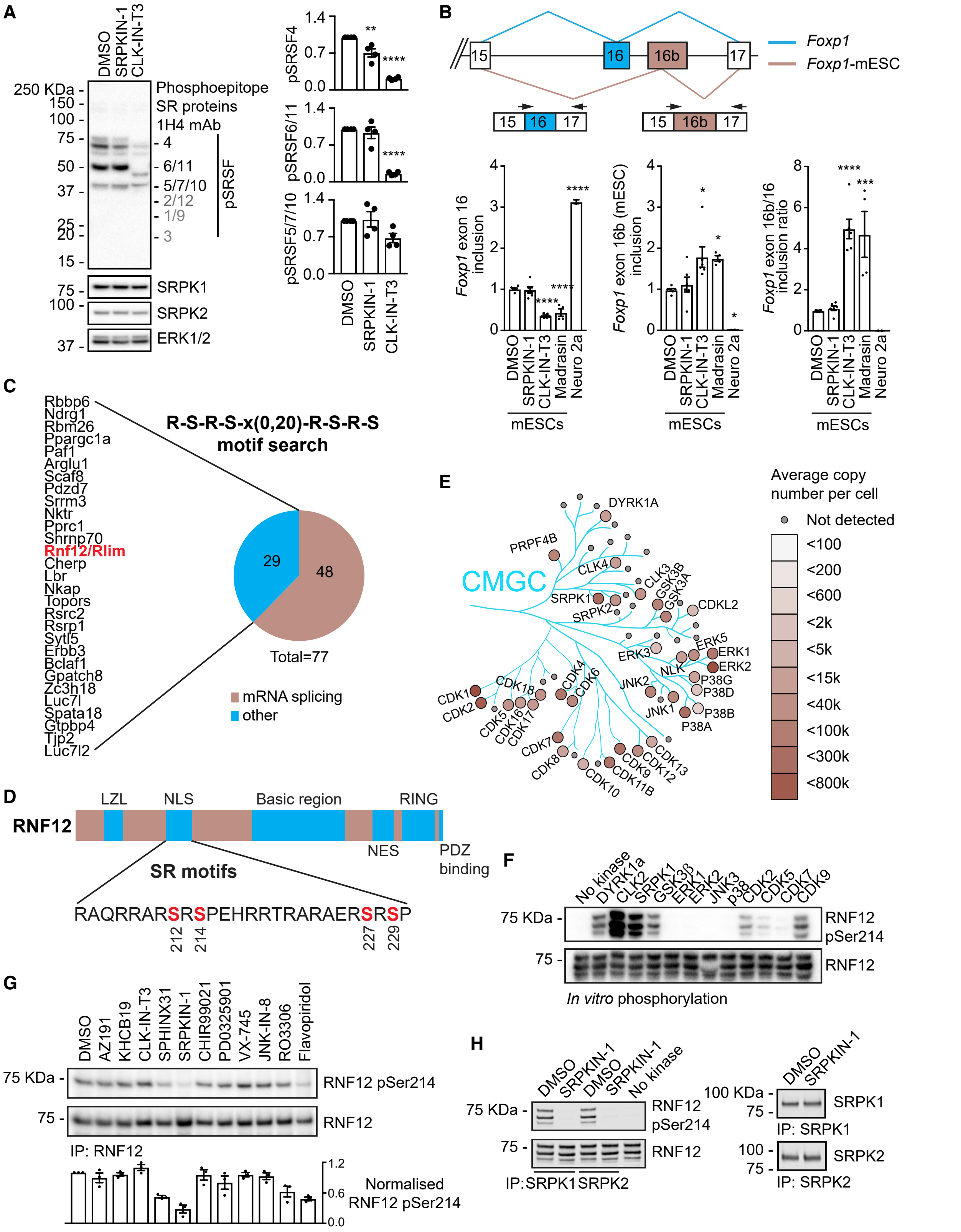

Figure 1. Functional Diversification of SRPK to Control Developmental Ubiquitin Signaling

(A) Wild-type (WT) mESCs were treated with 10 μM SRPKIN-1 or CLK-IN-T3 for 4 h, and phosphorylation of Ser-Arg rich splicing factors (SRSF) was assessed (Left). SRSF phosphorylation, SRPK1, SRPK2, and ERK1/2 levels were determined by immunoblotting. Expected positions of SRSFs that are not detected are

(legend continued on next page)

members exhibit highly tissue-specific expression profiles (Nakagawa et al., 2005; Wang et al., 1998), suggesting that these protein kinases may indeed perform specialized functions required for multicellular development.

Here, we show that SRPKs have undergone functional diversification to acquire a critical role in mammalian development. Surprisingly, SRPK activity does not make a major contribution to SRSF phosphorylation or to a key splicing switch in mammalian embryonic stem cells. Instead, SRPK controls a ubiquitin signaling pathway to regulate expression of neurodevelopmental genes. In this pathway, SRPK phosphorylates a Ser-Arg-rich regulatory motif on the E3 ubiquitin ligase RNF12/RLIM (Barakat et al., 2011; Shin et al., 2010, 2014; Zhang et al., 2012), which is mutated in the X-linked intellectual disability disorder Tonne-Kalscheuer syndrome (TOKAS) (Frints et al., 2019; Hu et al., 2016; Tønne et al., 2015). Processive RNF12 phosphorylation by SRPK stimulates ubiquitylation of transcription factor substrates to modulate expression of neural genes. Data mining indicates that SRPK family genes are also mutated in intellectual disability disorders, and SRPK3 point mutations, identified in patients, impair RNF12 phosphorylation. Thus, we uncover a previously unappreciated function for SRPK in neurodevelopmental signaling, indicating that functional diversification during eukaryotic evolution has enabled this highly conserved kinase family to govern complex metazoan processes beyond splicing regulation.

RESULTS

SRPK Activity Plays a Minor Role in Ser-Arg Rich Splicing Factor (SRSF) Phosphorylation in Embryonic Cells

SRPKs are thought to be key players in splicing regulation, controlling spliceosome assembly and activity (Dagher and Fu, 2001; Yeakley et al., 1999) via phosphorylation of SRSFs (Long and Caceres, 2009; Roscigno and Garcia-Blanco, 1995; Wu and Maniatis, 1993). Although splicing plays a critical role in stem cell regulation (Gabut et al., 2011; Salomonis et al., 2010), the first function of SRPK during early development in mammals has only recently been reported (Gou et al., 2020). This prompted us to examine the role of SRPK in mouse embryonic stem cells (mESCs). We first sought to confirm that SRPK activity is required for SRSF phosphorylation using an antibody that detects phosphorylated Ser-Arg-rich motifs. Surprisingly, in contrast with reports from somatic cells (Hatcher et al., 2018), phosphorylation of the major phosphorylated SRSF proteins in mESCs is either not significantly altered (SRSF6/11, SRSF5/7/10) or only slightly inhibited (SRSF4) by the selective pan-SRPK inhibitor, SRPKIN-1 (Hatcher et al., 2018) (Figure 1A). In contrast, treatment of mESCs with CLK-IN-T3, a selective inhibitor of the closely related CLK kinases (Funnell et al., 2017), which also phosphorylate SRSF splicing factors (Colwill et al., 1996), leads to widespread, robust inhibition of SRSF phosphorylation (Figure 1A). Our results therefore suggest that SRPKs are not the major SRSF kinases in mESCs.

This unexpected observation prompted us to examine whether SRPK activity is required for a key mESC alternative splicing switch, namely, inclusion of a specific exon within the developmental transcription factor FOXP1. mESCs express *Foxp1* mRNA that includes either exon 16b or exon 16, while differentiated somatic cells include only exon 16 (Figure 1B) (Gabut et al., 2011). As expected, the exon 16b-exon 16 switch requires mRNA splicing activity, as treatment of mESCs with the splicing inhibitor Madrasin (Pawellek et al., 2014) promotes inclusion of exon 16b over exon 16 (Figure 1B). However, selective inhibition of SRPK with SRPKIN-1 in mESCs has little effect on exon 16b-exon 16 inclusion (Figure 1B), consistent with the minor impact of SRPK inhibition on SRSF splicing factor phosphorylation. In contrast, selective inhibition of CLK by CLK-IN-T3 phenocopies splicing inhibition and promotes exon 16b inclusion while suppressing inclusion of exon 16 (Figure 1B). These data indicate that SRPK activity is not required for a FOXP1 alternative splicing switch in mESCs, implying that SRPK may have acquired other developmental function(s) during metazoan evolution.

Identification of SRPK Substrates and Functions in Embryonic Stem Cells

In order to shed light on further developmental functions of SRPKs, we sought to identify SRPK substrates. Previous studies have demonstrated that SRPKs directly phosphorylate Ser-Arg repeat (SR) motifs (Gui et al., 1994a, 1994b; Wang et al., 1998).

shown in gray. Quantification of SRSF phosphorylation (Right). Data represented as mean ± SEM (n = 4). One-way ANOVA followed by Tukey's multiple comparisons test; confidence level 95%. pSRSF4: (**) $p = 0.0032$, (****) $p < 0.0001$, pSRSF6/11: (****) $p < 0.0001$.

(B) Splice variants of *Foxp1* mRNA including mutually exclusive exons 16 (*Foxp1*, GenBank: NM_053202.2, cyan) or 16b (*Foxp1*-ESC, GenBank: XM_030255074.1, tan) (Top). mESCs were treated with 1 μM SRPKIN-1 or CLK-IN-T3, or 10 μM Madrasin for 8 h, and *Foxp1* exon 16-16b incorporation determined using specific quantitative RT-PCR primers. Neuro 2a is a control for exon 16b exclusion in differentiated cells (Bottom). Data represented as mean ± SEM (n = 3). One-way ANOVA followed by Tukey's multiple comparisons test; confidence level 95%. Exon 16 inclusion: (****) $p < 0.0001$, Exon 16b inclusion: (*) $p = 0.0164$, $p = 0.0485$, and $p = 0.0489$ (left to right). Ratio exon 16b/16: (****) $p < 0.0001$, (***) $p = 0.0003$.

(C) SRPK substrates predicted using ScanProsite and grouped according to UniProt functions.

(D) RNF12 phosphorylation sites detected by mass-spectrometry. LZL, leucine-zipper like; NLS, nuclear localization signal; NES, nuclear export signal; RING, RING E3 ubiquitin ligase catalytic domain.

(E) CMGC family kinase copy numbers in mESCs determined by quantitative proteomics and represented using Kinoviewer.

(F) CMGC kinase (200 mU) phosphorylation of the RNF12 SR-motif *in vitro* was determined by immunoblotting for RNF12 phospho-Ser214 and total RNF12.

(G) mESCs were treated with 10 μM of the following kinase inhibitors: AZ-191 (DYRK1B), KH-CB19 (CLK-DYRK), CLK-IN-T3 (CLK), SPHINX31 (SRPK1), SRPKIN-1 (pan-SRPK), CHIR-99021 (GSK-3), PD-0325901 (MEK1/2), VX-745 (p38), JNK-IN-8 (JNK), RO-3306 (CDK1), and flavopiridol (CDK7/9) for 4 h and RNF12 SR-motif phosphorylation determined by immunoblotting for RNF12 phospho-Ser214 and total RNF12. Normalized RNF12 Ser214 phosphorylation is shown below. Data represented as mean ± SEM (n = 3).

(H) SRPKIN-1 inhibition of SRPKs *in vivo* was determined by pre-treatment of mESCs with 10 μM SRPKIN-1 for 4 h followed by SRPK1 or SRPK2 immunoprecipitation kinase assay using RNF12 as a substrate. RNF12 SR-motif phosphorylation was analyzed by immunoblotting for RNF12 phospho-Ser214 and RNF12. SRPK1 and SRPK2 levels are shown as a loading control, Related to Figure S1; Tables S1 and S2.

Therefore, we interrogated the mouse proteome for characteristic SRPK consensus motifs of RSRS repeats separated by a linker of 0–20 residues using ScanProsite (https://prosite.expasy.org/scanprosite). A similar approach has been employed previously to identify a neural-specific splicing factor (Calarco et al., 2009). This analysis uncovered 77 predicted SRPK substrates, of which 48 have annotated splicing functions, while a smaller cohort of 29 is not known to participate in splicing regulation (Figure 1C; Tables S1 and S2). Interestingly, several have annotated developmental roles, including PAF1, which controls RNA PolII and stem cell pluripotency (Ding et al., 2009; Ponnusamy et al., 2009), and TJP2/ZO-2, a component of tight junctions. Also in this dataset is RNF12/RLIM, a RING-type E3 ubiquitin ligase (Figure 1C), which controls key developmental processes, including imprinted X-chromosome inactivation (Shin et al., 2014), and stem cell maintenance and differentiation (Bustos et al., 2018; Zhang et al., 2012). RNF12 variants cause an X-linked neurodevelopmental disorder termed as TOKAS (Frints et al., 2019; Hu et al., 2016; Tønne et al., 2015), which is underpinned by impaired RNF12 E3 ubiquitin ligase activity resulting in deregulated neuronal differentiation (Bustos et al., 2018). Thus, we hypothesized that SRPK phosphorylates and regulates RNF12, representing unappreciated functional diversification of SRPKs into developmental signaling.

The RNF12 SR-Motifs Are Phosphorylated by SRPK and Other CMGC Family Kinases

Previous work has shown that RNF12 is phosphorylated at the SR-motifs (Jiao et al., 2013), although the kinase(s) have not been identified. In order to confirm that RNF12 SR-motifs are phosphorylated *in vivo*, we performed immunoprecipitation mass spectrometry. RNF12 phosphorylation was robustly detected at two conserved sites in mESCs—the SR-motifs encompassing Ser212/214/227/229 and an unstudied Ser163 site (Figure 1D; Table S3)—confirming that the SR-motifs are major sites of RNF12 phosphorylation.

The RNF12 SR-motifs consist of tandem RpSRpSP sequences (Figure 1D) flanking a nuclear localization signal (NLS), which resemble sequences phosphorylated by SRPKs and several other CMGC kinase sub-families. Absolute quantitative proteomics shows that many CMGC family kinases, including SRPKs, are expressed in mESCs (Figures 1E and S1A). Thus, we employed a representative CMGC kinase panel to identify kinases that directly phosphorylate RNF12 *in vitro*. GSK-3β, CDK2, CDK9, and DYRK1A readily phosphorylate RNF12 at Ser214 within the SR-motifs (Figure 1F), while SRPK1 or the closely related kinase CLK2 give a higher level of RNF12 Ser214 phosphorylation (Figure 1F). The ERK subfamily of CMGC kinases, including ERK2, JNK, and p38, do not appreciably phosphorylate RNF12 at Ser214 (Figure 1F). These data identify SRPK and closely related kinases as strong candidates for catalyzing RNF12 SR-motif phosphorylation.

A Covalent SRPK Inhibitor Ablates RNF12 SR-Motif Phosphorylation

In order to identify the kinase that phosphorylates the RNF12 SR-motifs *in vivo*, we assembled a panel of kinase inhibitors that selectively inhibit CMGC family members. Of 12 CMGC family kinase inhibitors, a selective covalent inhibitor of SRPKs, SRPKIN-1 (Hatcher et al., 2018), had the greatest impact on the RNF12 phospho-Ser214/total ratio in mESCs (Figure 1G). The CDK7/9 inhibitor flavopiridol and the CDK1 inhibitor RO-3306 also have some effect, while the pan-CLK inhibitor CLK-IN-T3 has little impact on RNF12 phospho-Ser214/total ratio (Figure 1G). Interestingly, the structurally unrelated SRPK1 inhibitor SPHINX31 (Batson et al., 2017) has a minor effect on RNF12 Ser214 phosphorylation (Figure 1G), which is explained by the observation that SRPKIN-1 is ~10-fold and ~300-fold more potent toward SRPK1 than SPHINX31 and another commonly used SRPK inhibitor, SRPIN-340 (Fukuhara et al., 2006), respectively (Figure S1B). Furthermore, only SRPKIN-1 potently inhibited SRPK2 (Figure S1B), which is the other major SRPK isoform expressed in mESCs (Figures 1E, S1A, and S1C). Indeed, SRPK1 and SRPK2 are potently inhibited by SRPKIN-1 *in vivo*, as measured by the ability of SRPK1 or SRPK2 immunoprecipitates to phosphorylate RNF12 (Figure 1H). Our data therefore propose SRPK1/2 as candidate RNF12 SR-motif kinases.

Widespread, Selective RNF12 SR-Motif Phosphorylation by SRPK

Phosphoproteomic analysis suggests that RNF12 is phosphorylated at Ser212, Ser214, Ser227, and Ser229 within the SR-motifs (Jiao et al., 2013) (Figure 1D; Table S3). In order to globally assess phosphorylation of these sites, we devised a phos-tag approach, which retards the mobility of phosphorylated proteins on SDS-PAGE (Kinoshita et al., 2006). RNF12 is phosphorylated to high stoichiometry at all Ser residues within the SR-motif, as mutation of each increases RNF12 mobility (Figure 2A). Interestingly, mutation of Ser214 and Ser229 disrupts RNF12 phosphorylation to a similar extent as mutation of all four sites (4xSA; Figure 2A), suggesting that RNF12 SR-motifs undergo hierarchical phosphorylation with C- to N-terminal processivity characteristic of SRPK substrates (Ma et al., 2008; Ngo et al., 2008). Importantly, an RNF12 4xSA mutant displays phos-tag mobility similar to that of dephosphorylated RNF12 (Figure 2B).

In order to determine whether SRPKs and/or other kinases phosphorylate further sites within the RNF12 SR-motifs, we again screened our CMGC kinase inhibitor panel in combination with RNF12 phos-tag analysis. Of these, only SRPKIN-1 drove a major dephosphorylation of the RNF12 SR-motif (Figure 2C). In contrast, the SRPK1 selective inhibitor SPHINX31 and pan-CLK inhibitor CLK-IN-T3 showed a minor effect on RNF12 phosphorylation (Figure 2C), while the CDK7/9 inhibitor flavopiridol and the CDK1 inhibitor RO-3306, which also suppress the RNF12 phospho-Ser214/total ratio (Figure 1G), had little impact. SRPKIN-1 treatment led to RNF12 SR-motif de-phosphorylation at concentrations as low as 1 μM (Figure 2D) and within 1–2 h (Figure S2A). Furthermore, the high-mobility form of RNF12 was completely dephosphorylated at Ser214 upon SRPKIN-1 treatment (Figure 2E), indicating that SRPKs mediate widespread RNF12 SR-motif phosphorylation. In support of this notion, mass spectrometry indicated that SRPKs directly phosphorylate all four Ser residues within the RNF12 SR-motif *in vitro* (Figure 2F; Table S4). Furthermore, SRPK is highly selective for the RNF12 SR-motif, phosphorylating wild-type RNF12 but not a mutant in which the SR-motif is mutated (4xSA;

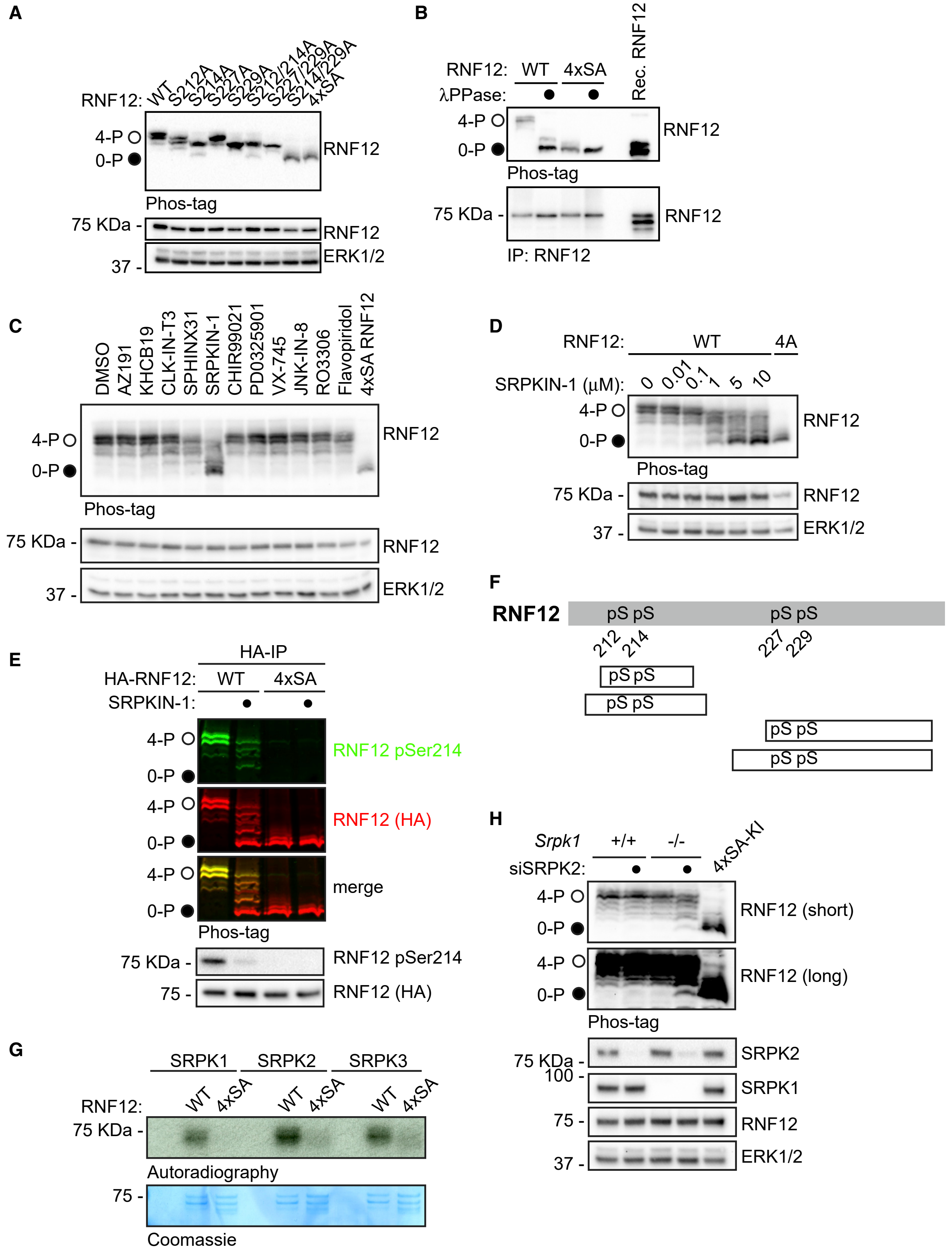

(legend on next page)

Figure 2G). In summary, our data uncovered a major role for SRPKs in phosphorylating the RNF12 SR-motif.

Further Evidence that SRPK1/2 Are RNF12 SR-Motif Kinases

In order to confirm that SRPK1/2 activity is responsible for RNF12 SR-motif phosphorylation, we first determined SRPKIN-1 kinase inhibition specificity. Consistent with previous kinase interaction data (Hatcher et al., 2018), SRPKIN-1 is highly specific for SRPK1 inhibition compared with 49 other kinases (Figure S2B). Furthermore, inhibitors of major SRPKIN-1 off-target kinases, including CHK2, PLK1, and DYRK1A, did not impact RNF12 SR-motif phosphorylation *in vivo* (Figure S2C). In addition, RNF12 SR-motif phosphorylation was inhibited by SRPKIN-1 in washout assays, where SRPKIN-1 remained covalently bound to SRPKs but off-target kinases were removed (Hatcher et al., 2018) (Figure S2D).

To further substantiate the role of SRPK1/2 in RNF12 SR-motif phosphorylation, we sought to generate Srpk1$^{-/-}$:Srpk2$^{-/-}$ mESC lines using CRISPR-Cas9. Although we were able to obtain Srpk1$^{-/-}$ and Srpk2$^{-/-}$ mESC lines (Figure S2E), no Srpk1$^{-/-}$:Srpk2$^{-/-}$ mESC lines were recovered, suggesting that SRPK1/2 perform redundant functions in mESCs. Accordingly, RNF12 SR-motif phosphorylation was unaffected in Srpk1$^{-/-}$ and Srpk2$^{-/-}$ mESCs (Figure S2E). We therefore sought to deplete SRPK2 in Srpk1$^{-/-}$ mESCs using siRNA. Partial depletion of SRPK2 expression in the absence of SRPK1 led to the appearance of a fraction of completely dephosphorylated RNF12 (Figure 2H), providing further evidence that SRPK1/2 phosphorylates the RNF12 SR-motif in mESCs. However, as several closely related CMGC family kinases, including CLK and DYRK, are expressed (Figure 1E) and able to phosphorylate RNF12 at Ser214 *in vitro* (Figure 1F), these kinases may also contribute to RNF12 SR-motif phosphorylation *in vivo*.

RNF12 SR-Motif Phosphorylation Drives Nuclear Anchoring

We then explored functions of the SRPK1/2-RNF12 pathway using RNF12 SR-motif knockin (KI) mutant mESCs. Employing CRISPR-Cas9, we engineered RNF12 4xSA-KI mESCs, which cannot be phosphorylated at the SR-motifs, and RNF12 ΔSR-KI mESCs, in which residues 206–229 of the SR-motif are deleted. We also engineered control RNF12 wild-type (WT)-KI mESCs and catalytically inactive RNF12 W576Y-KI mESCs. All mutants are expressed at similar levels and have a similar half-life (Figure S3A), but RNF12 4xSA is not phosphorylated at the SR-motifs (Figure S3B).

As RNF12 SR-motifs flank a NLS (Jiao et al., 2013), we used KI mutant mESC lines to investigate the role of SR-motif phosphorylation in subcellular localization. Wild-type RNF12 (RNF12 WT-KI) was localized entirely in the nucleus (Figure 3A), while RNF12 4xSA-KI and RNF12 ΔSR-KI showed significant staining in both the nucleus and cytosol (Figure 3A, nucleus/cytosol ratio: WT-KI = 13.11, 4xSA−KI = 1.39, ΔSR-KI = 0.84), indicating that RNF12-SR-motif phosphorylation promotes, but is not essential for, nuclear localization. In support of this, RNF12 4xSA was primarily nuclear in mESCs treated with the CRM nuclear export inhibitor leptomycin B (LMB) (Figure 3B, 4xSA-KI nucleus/cytosol ratio: Control = 1.63, LMB = 4.08). SRPK1 and SRPK2 were largely cytosolic, with some nuclear staining, particularly for SRPK2 (Figure 3C, cytosol/nucleus ratio: SRPK1 = 4.80, SRPK2 = 2.71), consistent with the notion that these kinases function outside the nucleus (Ding et al., 2006; Jang et al., 2009). Taken together, our data indicate that SRPK phosphorylation of the RNF12 SR-motif drives RNF12 nuclear anchoring but is not critical for nuclear translocation.

In light of these results, we tested whether RNF12 SR-motif phosphorylation is required for efficient degradation of nuclear substrates. A major RNF12 substrate is the REX1/ZFP42 transcription factor, which mediates RNF12 function in X-chromosome inactivation (Gontan et al., 2012, 2018). We first investigated the importance of RNF12 SR-motif phosphorylation for REX1 substrate engagement. RNF12-REX1 interaction was reduced in RNF12 4xSA-KI and RNF12 ΔSR-KI mESCs (Figure 3D), suggesting that SR-motif phosphorylation promotes RNF12 delivery to key nuclear substrates. Consistent with this notion, increased REX1 protein levels were observed in RNF12 4xSA-KI and RNF12 ΔSR-KI mESCs, to levels approaching that of catalytically inactive RNF12 W576Y-KI mESCs (Figure 3E). Furthermore, REX1 stability was increased in RNF12 4xSA KI, RNF12 ΔSR-KI, and RNF12 W576Y-KI mESCs, compared with RNF12 WT-KI control mESCs (Figure 3F). These data

Figure 2. RNF12/RLIM E3 Ubiquitin Ligase Is Selectively Phosphorylated by SRPKs at a SR-Rich Motif

(A) RNF12-deficient (*Rlim*$^{-/y}$) mESCs were transfected with WT RNF12 or the indicated point mutants and RNF12 SR-motif phosphorylation analyzed by phos-tag immunoblotting for RNF12. Fully phosphorylated (4-P) and unphosphorylated (0-P) RNF12 SR-motifs are indicated by open (○) and closed (●) circles, respectively. RNF12 4xSA = S212A/S214A/S227A/S229A.

(B) *Rlim*$^{-/y}$ mESCs were transfected with the indicated RNF12 constructs and lysates treated with λ-phosphatase and analyzed by phos-tag immunoblotting for RNF12. Unphosphorylated recombinant RNF12 is included as a control.

(C) mESCs were treated with 10 μM of the following kinase inhibitors: AZ-191 (DYRK1B), KH-CB19 (CLK-DYRK), CLK-IN-T3 (CLK), SPHINX31 (SRPK1), SRPKIN-1 (pan-SRPK), CHIR-99021 (GSK-3), PD-0325901 (MEK1/2), VX-745 (p38), JNK-IN-8 (JNK), RO-3306 (CDK1), and flavopiridol (CDK7/9) for 4 h and RNF12 SR-motif phosphorylation analyzed by phos-tag immunoblotting for RNF12. RNF12 4xSA is included as an unphosphorylated control.

(D) mESCs were treated with the indicated concentrations of SRPKIN-1 for 4 h and RNF12 SR-motif phosphorylation analyzed by phos-tag immunoblotting for RNF12.

(E) mESCs were treated with 10 μM SRPKIN-1 for 4 h and RNF12 phosphorylation analyzed from HA-RNF12 immunoprecipitates via RNF12 phos-tag and phospho-Ser214 immunoblotting using multiplex infrared immunoblot.

(F) Phosphorylated peptides detected by mass spectrometry following *in vitro* phosphorylation of RNF12 by SRPK1. pS, phospho-serine.

(G) Autoradiography of RNF12 WT or S212A/S214A/S227A/S229A (4xSA) following a radioactive kinase reaction with SRPK1, SRPK2, or SRPK3. RNF12 protein is detected by Coomassie staining.

(H) Srpk1$^{+/+}$ and Srpk1$^{-/-}$ mESCs were transfected with control or SRPK2 siRNA and RNF12 SR-motif phosphorylation analyzed by phos-tag immunoblotting for RNF12. SRPK2, SRPK1, RNF12, and ERK1/2 levels were determined by immunoblotting.

Related to Figure S2; Tables S3 and S4.

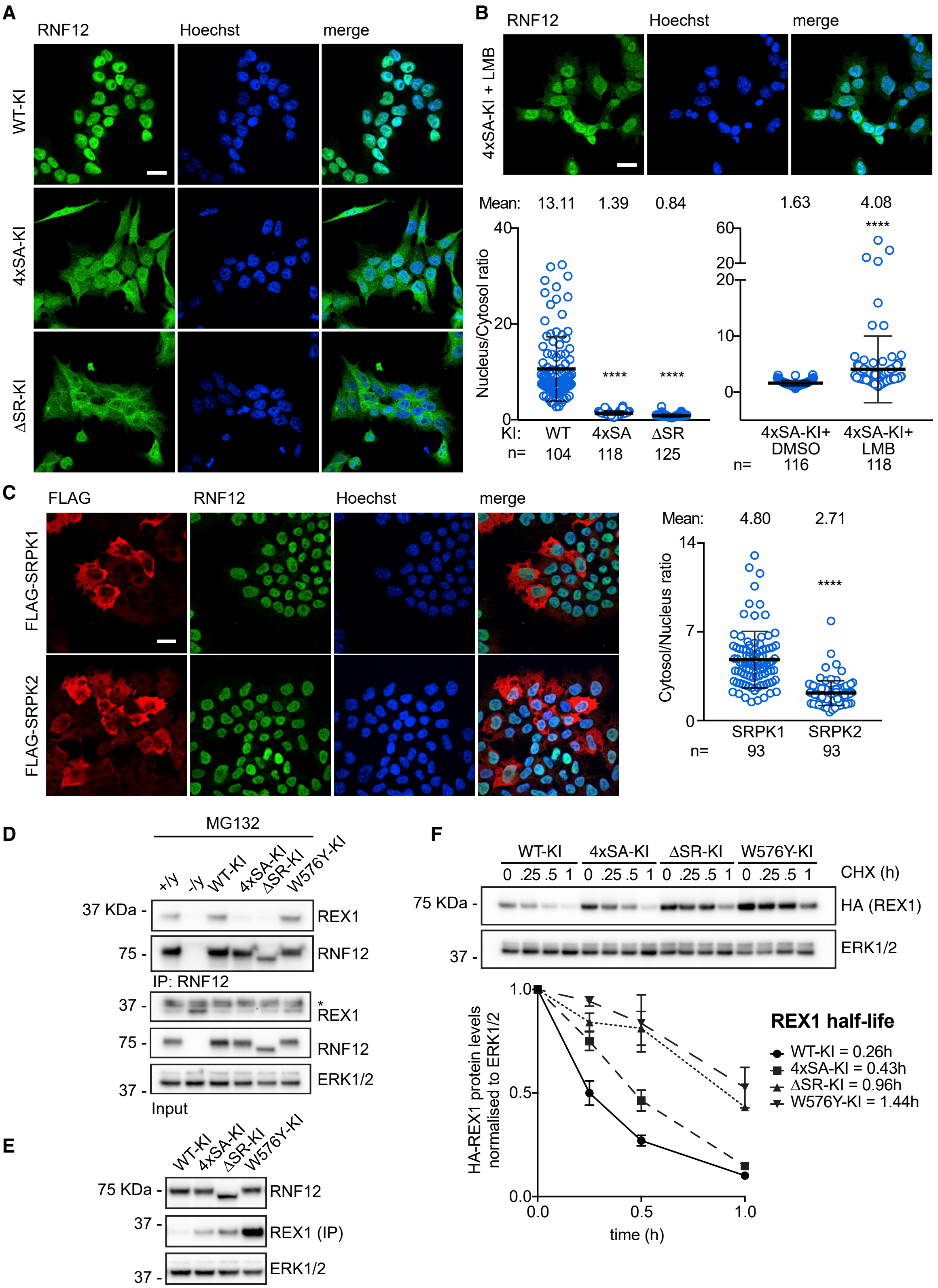

(legend on next page)

demonstrate that SRPK phosphorylation of RNF12 promotes REX1 targeting and degradation, and potentially that of other nuclear substrates.

RNF12 SR-Motif Phosphorylation by SRPK Stimulates E3 Ubiquitin Ligase Activity

As RNF12 SR-motif phosphorylation impacts substrate degradation, we investigated whether SRPK-mediated SR-motif phosphorylation also regulates RNF12 catalytic activity. We used SRPK to phosphorylate the RNF12 SR-motifs to high stoichiometry *in vitro* (Figures S4A and S4B) and compared the E3 ubiquitin ligase activity of phosphorylated and non-phosphorylated RNF12. Strikingly, REX1 ubiquitylation detected by fluorescently labeled ubiquitin was enhanced following RNF12 phosphorylation by SRPK2 (Figures 4A and 4B), which is not observed upon pre-incubation with SRPKIN-1 (Figure 4A), or with catalytically inactive SRPK2 (Figure 4B). We also used a REX1 antibody to directly visualize mono-ubiquitylated REX1 (Figure S4C). Similar results were obtained with SRPK1 (Figures S4D and S4E) and ubiquitylation of SMAD7 (Figure 4C), another reported RNF12 substrate (Zhang et al., 2012). Taken together, these results suggest that SRPK phosphorylation stimulates RNF12 substrate ubiquitylation. However, the impact on RNF12 substrate poly-ubiquitylation has not yet been directly demonstrated.

We then sought to determine the mechanism by which RNF12 SR-motif phosphorylation stimulates catalytic activity. The SR-motif resides proximal to a basic region implicated in RNF12 substrate ubiquitylation (Bustos et al., 2018), and as such could potentially regulate RNF12 engagement with E2 ubiquitin conjugating enzyme or substrate. First, we investigated the impact of SR-motif phosphorylation on RNF12-dependent discharge of ubiquitin from a loaded E2 conjugating enzyme onto free lysine. At a concentration where unphosphorylated RNF12 poorly discharges ubiquitin from UBE2D1 E2 (Figure S4F), phosphorylation by SRPK2 augments E2 discharge activity (Figure 4D). Therefore, RNF12 SR-motif phosphorylation enhances substrate-independent ubiquitin discharge from E2 ubiquitin conjugating enzyme.

We also explored the direct impact of RNF12 SR-motif phosphorylation on substrate interaction. *In vivo*, RNF12 SR-motif phosphorylation promotes ubiquitylation activity and delivery to nuclear substrates, such as REX1 (Figure 3). In contrast, the interaction between RNF12 and REX1 *in vitro* is destabilized by RNF12 SR-motif phosphorylation by SRPK1 (Figure 4E) or SRPK2 (Figure S4G), confirming that phosphorylation does not stimulate catalytic activity via increased substrate affinity. Taken together, our data indicate that RNF12 SR-motif phosphorylation by SRPK promotes delivery to nuclear substrates and stimulates intrinsic E3 ubiquitin ligase activity.

RNF12 E3 Ubiquitin Ligase Activity Controls a Neurodevelopmental Gene Expression Program

As SRPK-dependent phosphorylation of the SR-motif activates RNF12 and anchors it in the nucleus to promote degradation of transcription factor substrates, such as REX1, we sought to identify the gene expression program that is regulated by this emergent signaling pathway. To this end, we employed RNF12-deficient (*Rlim*$^{-/y}$) mESCs (Bustos et al., 2018) reconstituted with either wild-type RNF12 or an E3 ubiquitin ligase catalytic mutant (W576Y) and performed RNA sequencing (RNA-seq) to identify genes that are specifically regulated by RNF12. As validation of this experimental system, we show that REX1 degradation is restored by wild-type RNF12, but not RNF12 W576Y (Figure 5A). RNA-seq analysis reveals that RNF12 E3 ubiquitin ligase activity modulates expression of a significant cohort of RNAs (Figure 5B; 3,699 RNAs significantly altered, 19,721 RNAs not significantly altered). As proof of principle, the *Xist* long non-coding RNA, which has a key function in X-chromosome inactivation (Barakat et al., 2011), is regulated by RNF12 E3 ubiquitin ligase activity in the expected fashion (Figure 5B). Interestingly, additional comparison to control RNF12-deficient mESCs (Figure S5A) confirms that 1,032 RNAs are specifically suppressed by RNF12 in a manner dependent upon catalytic activity (Figure 5C).

In order to pinpoint functional groups of genes that are regulated by RNF12 E3 ubiquitin ligase activity, we employed Gene Ontology (GO) term analysis. Enriched within the cohort of RNF12-suppressed RNAs are those with GO terms associated with neuronal (Figure 5D; Table S5) and neural (Figure S5B; Table S6) development, differentiation, and function. This is consistent with a role for RNF12 in restricting mESC differentiation to neurons (Bustos et al., 2018). Genes assigned to neuron and/or neural GO terms are highlighted on a further plot of RNAs that are specifically regulated by RNF12 re-expression (Figure S5C). Interestingly, RNF12 suppresses expression of genes assigned

Figure 3. SRPK Phosphorylation of RNF12 Regulates Nuclear Anchoring and E3 Ubiquitin Ligase Activity

(A) RNF12 localization in wild-type knockin (WT-KI), SR-motif phosphorylation site knockin (4xSA-KI), or SR-motif deletion (ΔSR-KI) mESCs was determined by immunofluorescence. Scalebar: 20 μm (Left). Quantification of the Nucleus/cytosol fluorescence intensity ratio (Right). Data represented as mean ± SEM. One-way ANOVA followed by Tukey's multiple comparisons test; confidence level 95%. (****) $p < 0.0001$.

(B) RNF12 4xSA-KI mESCs were treated with 30 nM leptomycin B for 6 h and RNF12 localization analyzed by immunofluorescence. Scale bar: 20 μm (Top). Quantification of the nucleus/cytosol fluorescence intensity ratio (Bottom). Data represented as mean ± SEM Unpaired Student's t test, two-sided, confidence level 95%. (****) $p < 0.0001$.

(C) FLAG-tagged SRPK1 and SRPK2 were expressed in mESCs and localization of SRPKs and RNF12 analyzed by immunofluorescence. Scale bar: 20 μm (Left). Quantification of the cytosol/nucleus fluorescence intensity ratio (Right). Data represented as mean ± SEM Unpaired Student's t test, two-sided, confidence level 95%. (****) $p < 0.0001$.

(D) WT, *Rlim*$^{-/y}$, RNF12 WT-KI, 4xSA-KI, ΔSR-KI, and W576Y-KI mESCs were treated with 10 μM MG132 for 6 h and RNF12-REX1 co-immunoprecipitation analyzed. RNF12, REX1 and ERK1/2 were detected by immunoblotting. (*) indicates non-specific signal.

(E) REX1 levels were analyzed in RNF12 WT-KI, 4xSA-KI, ΔSR-KI, and W576Y-KI mESCs by immunoprecipitation followed by immunoblotting. ERK1/2 levels were detected by immunoblotting.

(F) REX1 half-life was determined in RNF12 WT-KI, 4xSA-KI, ΔSR-KI, and W576Y-KI mESCs by immunoblotting. (Top) quantification of HA-REX1 protein levels normalized to ERK1/2 and calculated protein half-life (Bottom). Data represented as mean ± SEM (n = 3). Related to Figure S3.

CellPress
OPEN ACCESS

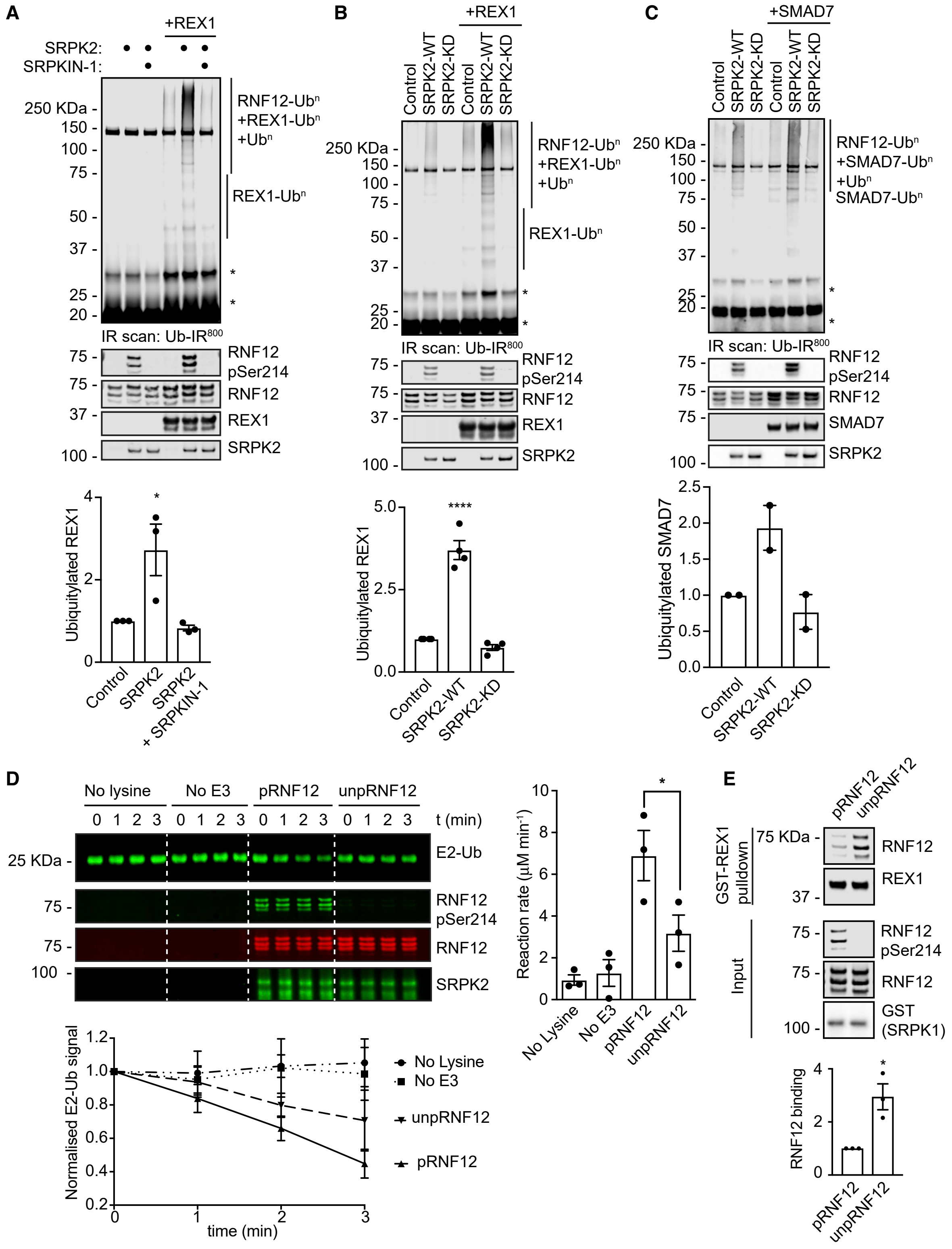

Figure 4. SRPK Phosphorylation Directly Stimulates RNF12 E3 Ubiquitin Ligase Activity

(A) Recombinant RNF12 was incubated with SRPK2 ± 10 μM SRPKIN-1 and REX1 ubiquitylation assessed. Infrared scans of ubiquitylated substrate signal (Top) and quantification (Bottom). Data represented as mean ± SEM (n = 3). One-way ANOVA followed by Tukey's multiple comparisons test; confidence level 95%. (*) p = 0.0350. Phospho-Ser214 and total RNF12, REX1, and SRPK2 infrared immunoblots are shown. * = non-specific fluorescent signal.

(legend continued on next page)

to the “neural crest cell differentiation” GO term (GO: 0014033), which are linked to craniofacial abnormalities associated with neurodevelopmental syndromes (Table S7). In summary, we uncovered a neural and/or neuronal gene expression program that is suppressed by RNF12, providing a molecular framework for RNF12-dependent regulation of neurodevelopmental processes (Bustos et al., 2018).

SRPK Signaling to RNF12 Regulates Neurodevelopmental Genes

These results prompted us to investigate the function of SRPK-RNF12 signaling in regulating expression of RNF12-responsive genes that have key functions in neural development. These are Delta-like 1 (*Dll1*), a regulator of Notch signaling in neural stem cells (Grandbarbe et al., 2003), Netrin-1 (*Ntn1*) and *Unc5a*, an axon guidance system essential for coordination of neuronal connections (Ackerman et al., 1997; Leonardo et al., 1997; Serafini et al., 1996), *Kif1a*, a motor protein for axonal transport (Okada and Hirokawa, 1999) and *Gfap*, an marker of astrocytes and radial glial cells (Middeldorp and Hol, 2011). Expression of each of these mRNAs, with the exception of *Unc5a*, increases during *in vitro* neural differentiation (Figure S5D), when the RNF12 SR-motif is phosphorylated (Figure S5E). Consistent with our RNA-seq data (Figure 5B), *Dll1*, *Ntn1*, *Unc5a*, and *Gfap* are expressed at low levels in control RNF12 WT-KI mESCs, and this was augmented in catalytically inactive RNF12 W576Y-KI mESCs (Figure 5E). *Kif1a* is expressed as at least 7 different splice isoforms in mouse, which likely explains conflicting results between RNA-seq and quantitative RT-PCR analysis. Nevertheless, our data confirm that RNF12-regulated neural genes are controlled by endogenous RNF12 E3 ubiquitin ligase activity in mESCs.

We next employed RNF12 KI mESC lines to determine the importance of SRPK signaling to RNF12 in regulation of neural gene expression. Compared with RNF12 WT-KI mESCs, neural gene expression is generally augmented by mutation of the SR-motif phosphorylation sites (RNF12 4xSA KI), deletion of the entire motif (RNF12 ΔSR-KI), or disruption of E3 ubiquitin ligase activity (RNF12 W576Y-KI; Figure 5E). Therefore, SRPK phosphorylation of RNF12 regulates key neural genes, implicating the SRPK-RNF12 pathway in the control of neurodevelopmental processes. As further evidence of the importance of the SR-motif for RNF12-dependent transcriptional regulation, induction of the known RNF12 target gene *Xist* was similarly disrupted by SR-motif mutation or deletion (Figure S5F).

The SRPK-RNF12 Pathway Regulates Gene Expression by Promoting REX1 Degradation

As RNF12 SR-motif phosphorylation is required for efficient substrate ubiquitylation and target gene regulation, we sought to further define the molecular pathway. The REX1 transcription factor substrate plays a critical role in RNF12-dependent regulation of *Xist* gene expression and X-chromosome activation (Gontan et al., 2012, 2018). Thus, we hypothesized that REX1 ubiquitylation and degradation is the mechanism by which RNF12 modulates neural gene expression. We generated RNF12/REX1 double knockout mESCs (*Rlim*$^{-/y}$:Zfp42$^{-/-}$; Figure 5F) to investigate whether REX1 disruption reverses the gene expression changes observed in RNF12-deficient mESCs (*Rlim*$^{-/y}$). Neural gene expression was augmented in RNF12-deficient mESCs, while additional knockout of REX1 (*Rlim*$^{-/y}$:Zfp42$^{-/-}$) reverses this gene expression profile (Figure 5F). These data illuminate REX1 as a key substrate that controls neurodevelopmental gene expression downstream of SRPK-RNF12 signaling.

Human Intellectual Disability Mutations in the SRPK-RNF12 Pathway Lead to a Deregulated Neurodevelopmental Gene Expression Program

Hereditable variants in RNF12 cause a neurodevelopmental disorder termed as TOKAS, which is a syndromic form of X-linked intellectual disability (Frints et al., 2019; Hu et al., 2016; Tønne et al., 2015). We showed previously that TOKAS mutations specifically impair RNF12 E3 ubiquitin ligase activity leading to deregulated neuronal differentiation (Bustos et al., 2018). In order to determine whether aberrant SRPK-RNF12 dependent neurodevelopmental gene expression might be relevant for TOKAS etiology, we examined expression of neural genes in mESCs harboring an RNF12 TOKAS patient mutation (mouse R575C—equivalent to human R599C) (Bustos et al., 2018). Expressions of *Dll1* and *Kif1a* were significantly increased in TOKAS mutant mESCs, with *Ntn1*, *Unc5a*, and *Gfap* also showing a tendency toward increased expression (Figure 6A). Thus, RNF12 TOKAS mutation partially phenocopies RNF12 SR-motif mutation with respect to the regulation of neurodevelopmental genes (Figure 5E).

As the SRPK-RNF12 signaling axis is disrupted in intellectual disability, we hypothesized that SRPK variants might cause related developmental syndromes. We mined molecular genetic databases of gene variants found in developmental disorders (Deciphering Developmental Disorders Study, 2015, 2017; Hu et al., 2016; Niranjan et al., 2015). A number of SRPK mutations have been identified in patients with intellectual disabilities or

(B) Recombinant RNF12 was incubated with WT or kinase dead (KD) SRPK2 and subjected to REX1 fluorescent ubiquitylation assays. Infrared scans of ubiquitylated substrate signal (Top) and quantification (Bottom). Data represented as mean ± SEM (n = 3). One-way ANOVA followed by Tukey’s multiple comparisons test; confidence level 95%. (****) p < 0.0001. Phospho-Ser214 and total RNF12, REX1, and SRPK2 infrared immunoblots are shown. * = non-specific fluorescent signal.

(C) Recombinant RNF12 was incubated with WT or KD SRPK2 and SMAD7 ubiquitylation assessed. Infrared scans of ubiquitylated substrate signal (Top) and quantification (Bottom). Data represented as mean ± SD (n = 2). Phospho-Ser214 and total RNF12, REX1, and SRPK2 infrared immunoblots are shown. * = non-specific fluorescent signal.

(D) Recombinant RNF12 was incubated with WT (pRNF12) or KD (unpRNF12) SRPK2 and subjected to E2 ubiquitin discharge assay. Infrared immunoblot scans (Top Left), reaction rate determinations (Top Right) and normalized quantification of E2-ubiquitin conjugate signal (Bottom) are shown. Data represented as mean ± SEM (n = 3). One-way ANOVA followed by Tukey’s multiple comparisons test; confidence level 95%. (*) p = 0.0490.

(E) Recombinant RNF12 was incubated with WT or KD SRPK1 and subjected to GST-REX1 pull-down assay. RNF12, REX1, phospho-Ser214 RNF12 and SRPK1 infrared immunoblots (Top) and RNF12-REX1 binding quantification (bottom) are shown. Data represented as mean ± SEM (n = 3). Unpaired Student’s t test, two-sided, confidence level 95%. (*) p = 0.0162. Related to Figure S4.

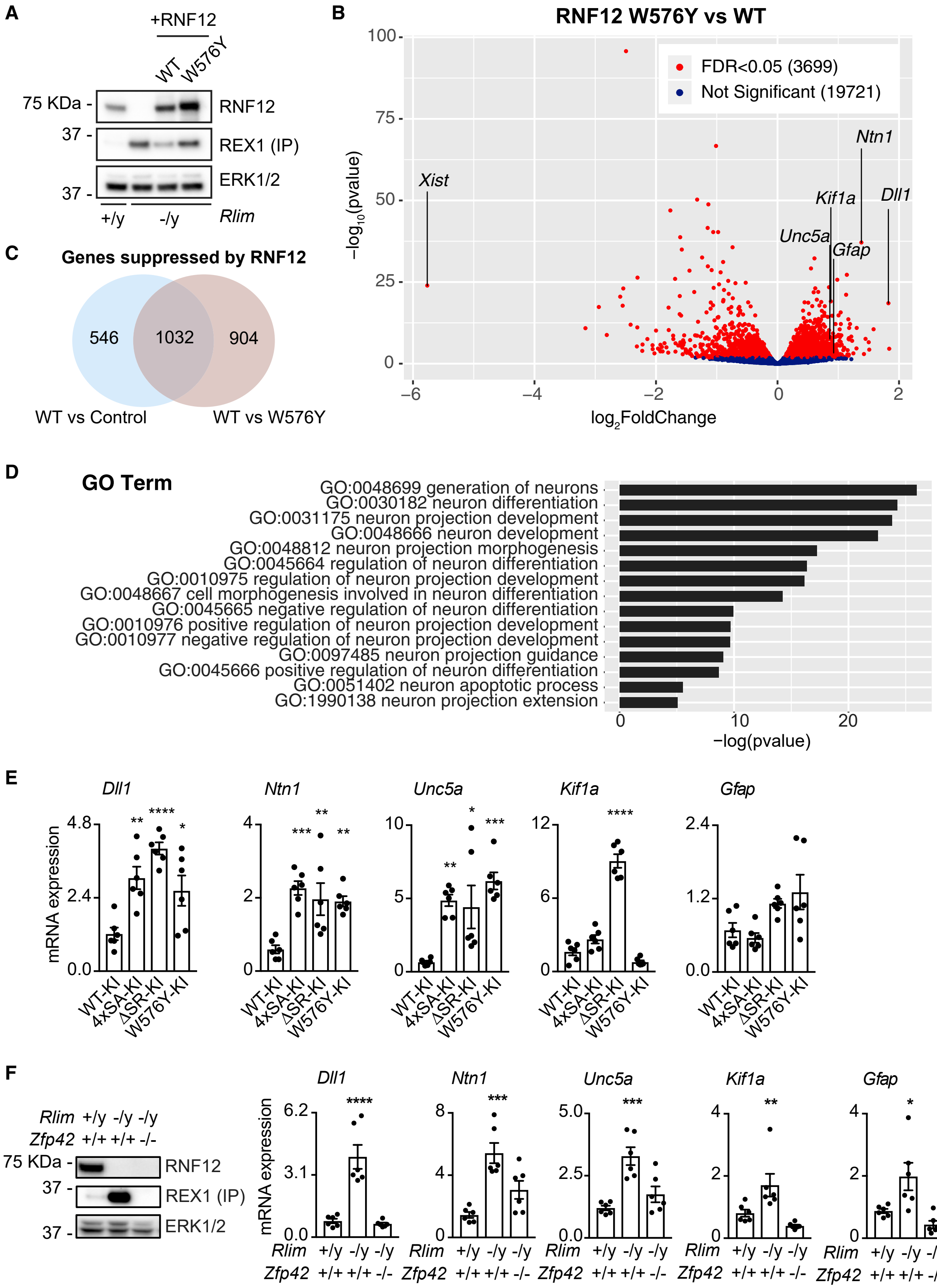

Figure 5. RNF12-REX1 Signaling Controls a Neurodevelopmental Gene Expression Program

(A) *Rlim*$^{-/y}$ mESCs were transfected with WT or catalytically inactive (W576Y) RNF12. REX1 levels were analyzed by immunoprecipitation and immunoblotting, RNF12 and ERK1/2 levels were determined by immunoblotting.

(legend continued on next page)

similar developmental abnormalities (Figure 6B, top). Of those, *SRPK2* is mainly deleted, suggesting that loss of SRPK2 expression may be a feature of these disorders. A number of duplications of the X-linked *SRPK3* gene were identified (Figure 6B), which is likely explained by frequent X-chromosome duplications in developmental disorders. Interestingly, several point mutations within the SRPK3 kinase domain (Figure 6B, bottom) have been reported in X-linked intellectual disability (Hu et al., 2016). We tested the effect of these mutations on the ability of SRPK3 to phosphorylate RNF12. SRPK3, H159D, and T211M mutations strongly impaired SRPK3 phosphorylation of RNF12, while K270M disrupted RNF12 phosphorylation to a lesser extent (Figure 6C). Thus, variants found in intellectual disability patients impair the ability of SRPK to phosphorylate RNF12, suggesting that SRPK function may be disrupted in intellectual disability disorders.

These findings prompted us to investigate the expression and function of SRPK family members in human pluripotent stem cells and the brain. SRPK1, SRPK2, and RNF12 are expressed in human induced pluripotent stem cells (hiPSCs; Figure 6D), and quantitative total proteomic analysis confirmed the expression of these components and REX1 (Figure 6E). Furthermore, the pathway is active in human pluripotent cells, as treatment of hiPSCs with the SRPK inhibitor SRPKIN-1 promotes RNF12 SR-motif dephosphorylation (Figure 6F). Mining single nuclei RNA-seq sequencing data (Hodge et al., 2019) revealed that *SRPK1* and *SRPK2* are broadly expressed in human cortical neurons, while *SRPK3* is specifically expressed in two GABAergic inhibitory neuron populations (Figure 6G), which have been implicated in intellectual disability (Sgadò et al., 2011; Smith-Hicks, 2013). RNF12, SRPK1, and SRPK2 are also robustly expressed in the adult mouse brain (Figure 6H). Therefore, the SRPK-RNF12 pathway is expressed and active in human pluripotent stem cells, and the components are expressed in adult human cortical neurons and mouse brain. Taken together, our data suggest that SRPK-RNF12 signaling is conserved during mouse and human neuronal development.

SRPK Phosphorylates the RNF12 SR-Motif in Neurons

Finally, we investigated the function of the SRPK-RNF12 pathway in neurons. Consistent with gene expression data from human cortical neurons (Figure 6G) and adult mouse brain (Figure 6H), RNF12, SRPK1, and SRPK2 were robustly expressed during maturation of isolated mouse fetal cortical neural progenitors *in vitro* (Figures 7A and S6). In contrast, SRPK3 (Figure S6) and REX1 (Figure 7B) were not detected in cultured mouse cortical neurons. RNF12 was predominantly localized to the nucleus in these neurons (Figure 7C), and phos-tag analysis indicated that the RNF12 SR-motif was heavily phosphorylated throughout a time course of neuronal maturation (Figure 7D). Furthermore, treatment of mature mouse cortical neurons with the selective SRPK inhibitor SRPKIN-1 suppressed RNF12 phosphorylation, as measured by phos-tag (Figure 7E). These data confirm that SRPKs phosphorylate the RNF12 SR-motif during neuronal maturation *in vitro*, suggesting that SRPK activity regulates RNF12 function in the nervous system.

DISCUSSION

Functional diversification of protein kinases is a key evolutionary tool, employing pre-existing signaling cassettes for regulation of complex cellular processes. However, the importance of functional diversification in the regulation of multi-cellularity remains unclear. Here, we show that SRSF protein kinase (SRPK), a highly conserved kinase family implicated in mRNA splicing, has undergone functional diversification to control developmental ubiquitin signaling. In mammalian embryonic stem cells, we found that SRPK activity is not required for splicing regulation. Instead, SRPK phosphorylates the E3 ubiquitin ligase RNF12/RLIM to control neurodevelopmental gene expression (Figure 7F). This function may have initially evolved to enable coordinated control of core cellular processes, such as RNA splicing, with key developmental events in multicellular organisms.

Our studies reveal that RNF12 SR-motif phosphorylation by SRPK drives delivery to nuclear substrates and increases substrate-independent ubiquitin discharge by a cognate E2-conjugating enzyme, indicating that phosphorylation of these motifs is required for maximal catalytic activity. Although RNF12 SR-motifs are distal to the catalytic RING domain, previous work confirms that distal non-RING regulatory elements play important roles in RNF12 catalysis (Bustos et al., 2018; Frints et al., 2019). Indeed, phosphorylation of distal non-RING elements in another RING E3 c-CBL mediates enzymatic activation (Dou et al., 2013). Structural investigations of full-length RNF12 in complex with cognate E2, ubiquitin, and substrate will be required to determine how phosphorylation drives enzymatic activation at the atomic level.

Our findings propose a critical role for SRPK in regulating developmental processes, although functional redundancy within the mammalian SRPK family has precluded genetic interrogation of SRPK functions during development. Nevertheless, a functional genomic screening indicated that SRPK2 is required

(B) Volcano plot of RNA-seq analysis comparing *Rlim*$^{-/y}$ mESCs transfected with WT or W576Y RNF12. RNAs that are significantly altered by RNF12 E3 ubiquitin ligase activity are displayed in red. Selected neurodevelopmental mRNAs are labeled (*Dll1*, *Ntn1*, *Unc5a*, *Kif1a*, *Gfap*). *Xist* is a positive control for RNF12 E3 ubiquitin ligase activity. FDR, false discovery rate.

(C) Venn diagram displaying total number of RNAs negatively regulated by RNF12 catalytic activity. Intersection (1,032 genes) represents RNAs whose expression is significantly altered when comparing control versus WT RNF12, and WT RNF12 versus W576Y catalytic mutant.

(D) GO category enrichment analysis of genes/RNAs related to the GO term "neuron" whose expression is inhibited by RNF12 (232 genes).

(E) RNF12 WT-KI, 4xSA-KI, ΔSR-KI, and W576Y-KI mESCs were subjected to quantitative RT-PCR analysis of relative mRNA expression. Data represented as mean ± SEM (n = 3). One-way ANOVA followed by Tukey's multiple comparisons test; confidence level 95%. *Dll1* (**) p = 0.0058, (****) p < 0.0001, (*) p = 0.0377; *Ntn1* (***) p = 0.0008, (**) p = 0.0057, (**) p = 0.0082; *Unc5a* (**) p = 0.0079, (*) p = 0.0188, (***) p = 0.0006. *Kif1a* (****) p < 0.0001.

(F) RNF12, REX1, and ERK1/2 protein levels in WT, *Rlim*$^{-/y}$ and *Rlim*$^{-/y}$:Zfp42$^{-/-}$ mESCs were determined by immunoblotting (RNF12 and ERK1/2) and immunoprecipitation followed by immunoblotting (REX1) (Left). WT, *Rlim*$^{-/y}$ and *Rlim*$^{-/y}$:Zfp42$^{-/-}$ mESCs were analyzed for relative mRNA expression by quantitative RT-PCR (Right). Data represented as mean ± SEM (n = 3). One-way ANOVA followed by Tukey's multiple comparisons test; confidence level 95%. *Dll1* (****) p < 0.0001; *Ntn1* (***) p = 0.0002; *Unc5a* (***) p = 0.0003; *Kif1a* (*) p = 0.0316; *Gfap* (*) p = 0.0261, Related to Figure S5; Tables S5–S7.

CellPress
OPEN ACCESS

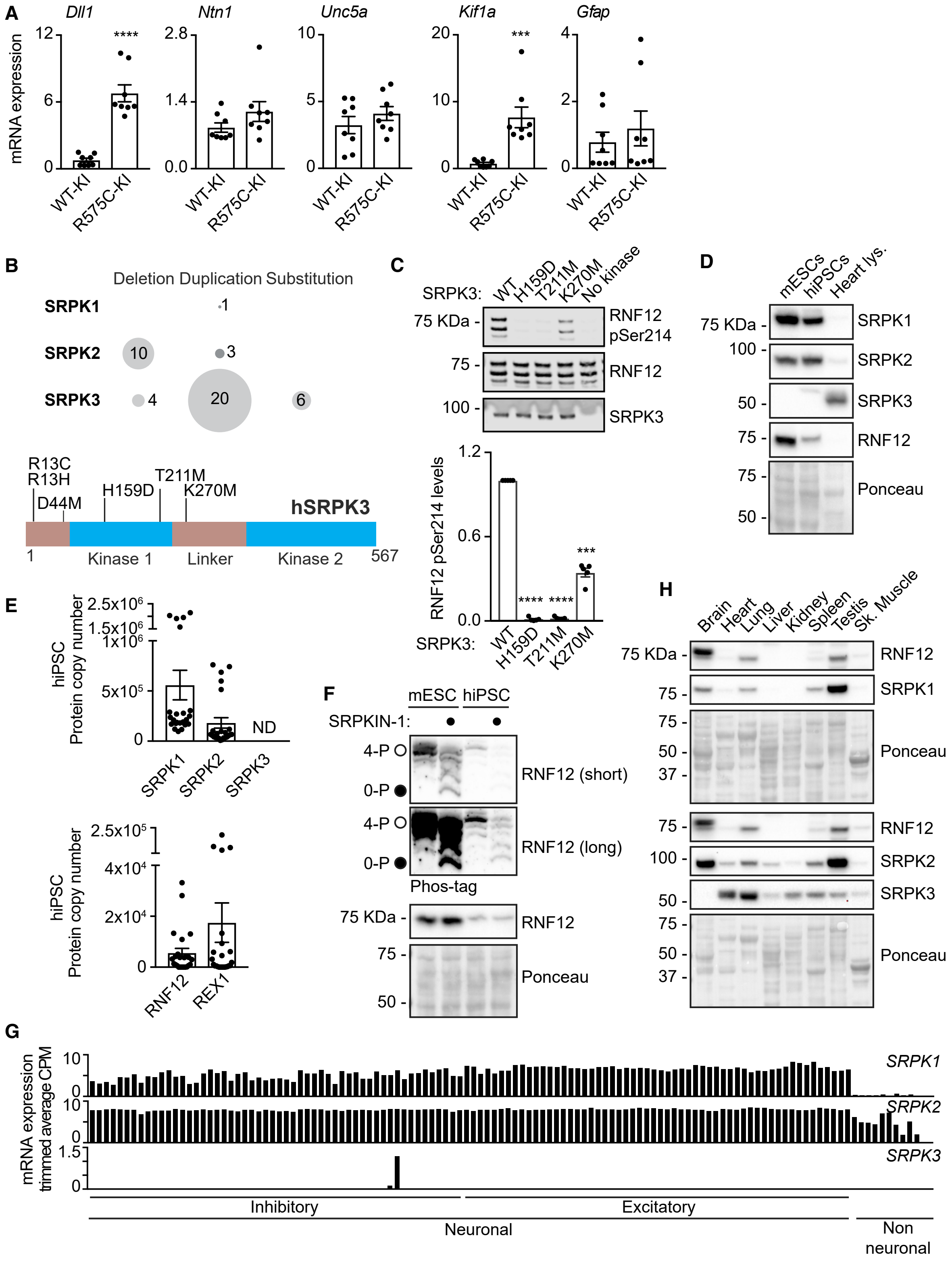

Figure 6. The SRPK-RNF12 Signaling Pathway Is Deregulated in Human Intellectual Disability

(A) RNF12 WT-KI or R575C-KI mESCs were analyzed for relative mRNA expression by quantitative RT-PCR. Data represented as mean ± SEM (n = 3). Unpaired Student's t test, two-sided, confidence level 95%. *Dll1* (****) $p < 0.0001$; *Kif1a* (***) $p = 0.0005$.

(legend continued on next page)

for efficient X-chromosome inactivation (Chan et al., 2011), which is a key developmental function of RNF12. Furthermore, a recent study showed that SRPK1 initiates zygotic genome activation by phosphorylating protamine (Gou et al., 2020). Therefore, emerging evidence provides support for the notion that SRPKs perform key developmental functions.

SRPK signaling to RNF12 may ensure correct regulation of neural development. SRPK2 is highly expressed in the brain (Wang et al., 1998) and regulates processes relevant to neurodegeneration (Hong et al., 2012; Wang et al., 2017), suggesting a role for SRPK in development and maintenance of the nervous system. Additionally, we demonstrate that SRPK3 is expressed in sub-sets of human GABAergic neurons. Therefore, we propose that SRPK2 deletion or SRPK3 mutation may disrupt RNF12 function during development or maintenance of specific neuronal populations, leading to intellectual disability. A systematic analysis of SRPK and RNF12 expression during nervous system development is now required to identify specific cell populations in which SRPK-RNF12 signaling is relevant and potentially disrupted in intellectual disability.

Regulation of SRPK in a developmental context also remains unexplored. Previous work suggests that SRPKs are constitutively activated (Ngo et al., 2007), with additional regulatory inputs from the AKT-mTOR pathway (Jang et al., 2009; Lee et al., 2017). Diverse temporal and tissue-specific SRPK expression patterns also suggest that transcriptional regulation may be a key mechanism to ensure that SRPK phosphorylates substrates, such as RNF12, within the correct developmental time and space.

Finally, a key question relates to the function of RNF12 substrates in neuronal development. Our data indicate that RNF12 controls neural gene expression by ubiquitylating the REX1 transcription factor. The SRPK-RNF12 axis therefore appears to act as a safeguard to prevent aberrant REX1 accumulation and expression of neuronal genes in pluripotent stem cells. Although REX1 has not previously been implicated in the regulation of neuronal development and is undetectable in neurons, pathological REX1 accumulation upon RNF12 pathway mutation may unleash neomorphic transcriptional functions that are detrimental to neuronal development. This system could influence neuronal development by (1) transcriptional suppression of neural genes in non-neural cells, (2) modulating the timing and levels of neural gene expression in the developing neuroepithelium, or (3) acting to regulate a specific gene at the top of the neurogenesis signaling cascade. These findings suggest that approaches to activate SRPKs or normalize expression of REX1, for example using protein degradation technologies, such as proteolysis targeting chimeras (PROTACs), might provide therapeutic benefit in patients with neurodevelopmental disorders underpinned by deregulated SRPK-RNF12 signaling.

STAR★METHODS

Detailed methods are provided in the online version of this paper and include the following:

- KEY RESOURCES TABLE
- RESOURCE AVAILABILITY
  - ○ Lead Contact
  - ○ Materials Availability
  - ○ Data and Code Availability
- EXPERIMENTAL MODEL AND SUBJECT DETAILS
  - ○ Cell Lines
  - ○ Animal Studies
- METHOD DETAILS
  - ○ Serine-Arginine Motif Search
  - ○ Plasmid and siRNA Transfection
  - ○ CRISPR/Cas9 Gene Editing
  - ○ Pharmacological Inhibitors
  - ○ Kinase Inhibitor Profiling
  - ○ Immunoblotting and Phos-Tag Analysis
  - ○ Mass Spectrometry
  - ○ Protein Expression and Purification
  - ○ *In Vitro* Kinase Assays
  - ○ Immunofluorescence
  - ○ *In Vitro* Phospho-RNF12 Activity Assays
  - ○ Binding Assays
  - ○ RNA-Sequencing and Gene Ontology Analysis
  - ○ RNA Extraction and Quantitative RT-PCR
- QUANTIFICATION AND STATISTICAL ANALYSIS

SUPPLEMENTAL INFORMATION

Supplemental Information can be found online at https://doi.org/10.1016/j.devcel.2020.09.025.

ACKNOWLEDGMENTS

We thank Nathanael Gray and Tinghu Zhang (Dana-Farber Cancer Institute) for providing SRPKIN-1, Sam Aparicio (University of British Columbia) for CLK-IN-T3, Helen Walden and Viduth Chaugule (University of Glasgow) for fluorescent ubiquitin, and Francisca Cornejo (Universidad Mayor) for hiPSC extracts. We also thank the following colleagues from the University of Dundee: Angus Lamond and Andrea Pawellek for Madrasin, Ron Hay and Emma Branigan for

(B) Graphical representation of SRPK intellectual disability variants reported in literature grouped by type of chromosomal mutation (Top) and position within the SRPK3 protein (Bottom).
(C) RNF12 phosphorylation *in vitro* by WT SRPK3 or the indicated mutants was analyzed by immunoblotting for RNF12 phospho-Ser214 and total RNF12 (Top). Quantification of infrared RNF12 phospho-Ser214 immunoblotting blotting signal normalized to total RNF12 (Bottom). Data represented as mean ± SEM (n = 3). One-way ANOVA followed by Tukey's multiple comparisons test; confidence level 95%. (****) $p > 0.0001$, (***) $p = 0.0001$.
(D) SRPK1, SRPK2, SRPK3, and RNF12 levels in mESCs, hiPSCs (CHiPS4 cell line), and mouse heart lysate were analyzed by immunoblotting.
(E) hiPSC (bubh_3 line) extracts were analyzed for average protein copy number via quantitative proteomics. Data were obtained from the human induced pluripotent stem cell initiative database (http://www.hipsci.org/) and represented as mean ± SEM (n = 24), ND, not detected.
(F) hiPSCs (CHiPS4 cell line) and mESCs were treated with 10 μM SRPKIN-1 for 4 h and RNF12 SR-motif phosphorylation analyzed by phos-tag immunoblotting for RNF12. Fully phosphorylated (4-P) and unphosphorylated (0-P) RNF12 SR-motif is indicated with open (○) and closed (●) circles, respectively.
(G) Single nuclei isolated from post-mortem human brain cortex neurosurgery were analyzed via SMART-seq v4 RNA-seq (data from Hodge et al., 2019). Each bar represents a distinct neuronal sub-type or non-neuronal cell. Trimmed average counts per million (CPM) for *SRPK1*, *SRPK2*, and *SRPK3* are shown.
(H) Expression of RNF12, SRPK1, SRPK2, and SRPK3 in adult mouse tissues was analyzed by immunoblotting. Ponceau S staining is shown as a loading control.

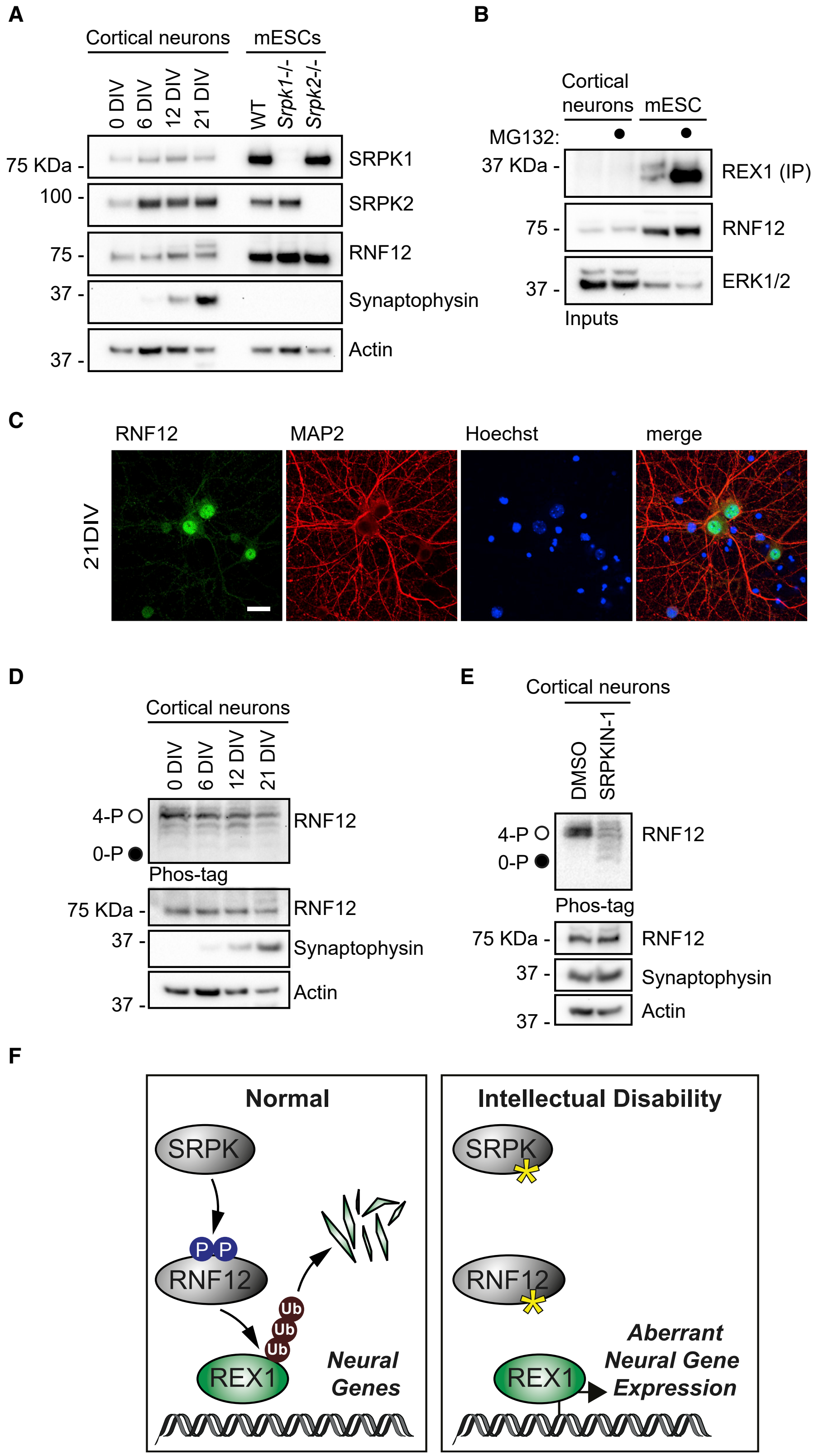

Figure 7. The SRPK-RNF12 Signaling Pathway Operates in Neurons

(A) Primary cortical neurons isolated from E16.5 C57BL6 mice were cultured for the indicated number of days in vitro (DIV) and RNF12, SRPK1, and SRPK2, synaptophysin and actin levels analyzed via immunoblotting alongside the indicated mESC lines.

(legend continued on next page)

UBE2D1, Miratul Muqit for expertise in mouse neuronal culture, Vicky Cowling and Joana Silva for mouse tissue extracts, Alejandro Brenes for HipSci proteomics data, and Kate Storey for critical reading of the manuscript. G.M.F is supported by a Wellcome Trust/Royal Society Sir Henry Dale fellowship (211209/Z/18/Z) and a Medical Research Council NEW Investigator award (MR/N000609/1). A.S.-F. is supported by a MRC-PPU prize studentship. G.N. and M.M. are supported by research grants ANID/FONDAP/15090007 and FONDECYT/11190998.

AUTHOR CONTRIBUTIONS

F.B. and G.M.F. conceived the study and designed the experiments. F.B., A.S.-F., A.C., O.A., L.B., L.D., J.M., R.G., J.V., and G.M.F. performed experiments. G.N. and M.M. analyzed data and prepared figures. T.M. and R.T. performed DNA cloning and CRISPR-Cas9 design. C.J.H. generated reagents. R.S. analyzed mass-spectrometry data. F.B. and G.M.F. wrote the paper.

DECLARATION OF INTERESTS

The authors declare no competing interests.

Received: March 23, 2020
Revised: August 17, 2020
Accepted: September 25, 2020
Published: October 19, 2020

SUPPORTING CITATIONS

The following references appear in the Supplemental Information: Bao and Jin, 2006, Byrd and Meyers, 2005, Chen et al., 2012, Chen et al., 2014, Coles et al., 2006, Dixon et al., 1994, Duprez et al., 1999, Fukumoto et al., 2006, Gammill et al., 2006, Gopinathan et al., 2019, Hargrave et al., 1997, Hill et al., 2014, Huang et al., 2016, Humphreys et al., 2012, Jia et al., 2016, Kamalakar et al., 2019, Li et al., 2009, Li et al., 2018, Micaglio et al., 2019, Nikopoulos et al., 2007, Parada et al., 2015, Pilia et al., 1999, Roosenboom et al., 2018, Sela-Donenfeld and Kalcheim, 1999, Shin et al., 2012, Sock et al., 2004, Teng et al., 2017, Toyofuku et al., 2008, Wang and Astrof, 2016, Wu and Taneyhill, 2012, Yen et al., 2010, York et al., 2018, Yu and Moens, 2005, Zhang et al., 2016, Zhu et al., 2019, Zuniga et al., 2010.

REFERENCES

Ackerman, S.L., Kozak, L.P., Przyborski, S.A., Rund, L.A., Boyer, B.B., and Knowles, B.B. (1997). The mouse rostral cerebellar malformation gene encodes an UNC-5-like protein. Nature *386*, 838–842.

Anders, S., Pyl, P.T., and Huber, W. (2015). HTSeq–a python framework to work with high-throughput sequencing data. Bioinformatics *31*, 166–169.

Bao, Z.Z., and Jin, Z. (2006). Sema3D and Sema7A have distinct expression patterns in chick embryonic development. Dev. Dyn. *235*, 2282–2289.

Barakat, T.S., Gunhanlar, N., Pardo, C.G., Achame, E.M., Ghazvini, M., Boers, R., Kenter, A., Rentmeester, E., Grootegoed, J.A., and Gribnau, J. (2011). RNF12 activates Xist and is essential for X chromosome inactivation. PLoS Genet. *7*, e1002001.

Batson, J., Toop, H.D., Redondo, C., Babaei-Jadidi, R., Chaikuad, A., Wearmouth, S.F., Gibbons, B., Allen, C., Tallant, C., Zhang, J., et al. (2017). Development of potent, selective SRPK1 inhibitors as potential topical therapeutics for neovascular eye disease. ACS Chem. Biol. *12*, 825–832.

Brenes, A., and Lamond, A.I. (2019). The encyclopedia of proteome dynamics: the KinoViewer. Bioinformatics *35*, 1441–1442.

Brenes, A.J., Yoshikawa, H., Bensaddek, D., Mirauta, B., Seaton, D.D., Hukelmann, J.L., Jiang, H., Stegle, O., and Lamond, A.I. (2020). Erosion of human X chromosome inactivation causes major remodelling of the iPSC proteome. bioRxiv biorxiv.org/content/10.1101/2020.03.18.997049v2.

Bustos, F., Segarra-Fas, A., Chaugule, V.K., Brandenburg, L., Branigan, E., Toth, R., Macartney, T., Knebel, A., Hay, R.T., Walden, H., and Findlay, G.M. (2018). RNF12 X-linked intellectual disability mutations disrupt E3 ligase activity and neural differentiation. Cell Rep. *23*, 1599–1611.

Byrd, N.A., and Meyers, E.N. (2005). Loss of Gbx2 results in neural crest cell patterning and pharyngeal arch artery defects in the mouse embryo. Dev. Biol. *284*, 233–245.

Calarco, J.A., Superina, S., O'Hanlon, D., Gabut, M., Raj, B., Pan, Q., Skalska, U., Clarke, L., Gelinas, D., van der Kooy, D., et al. (2009). Regulation of vertebrate nervous system alternative splicing and development by an SR-related protein. Cell *138*, 898–910.

Cao, W., Jamison, S.F., and Garcia-Blanco, M.A. (1997). Both phosphorylation and dephosphorylation of ASF/SF2 are required for pre-mRNA splicing in vitro. RNA *3*, 1456–1467.

Chan, K.M., Zhang, H., Malureanu, L., van Deursen, J., and Zhang, Z. (2011). Diverse factors are involved in maintaining X chromosome inactivation. Proc. Natl. Acad. Sci. USA *108*, 16699–16704.

Chen, Q., Wang, H., Hetmanski, J.B., Zhang, T., Ruczinski, I., Schwender, H., Liang, K.Y., Fallin, M.D., Redett, R.J., Raymond, G.V., et al. (2012). BMP4 was associated with NSCL/P in an Asian population. PLoS One *7*, e35347.

Chen, Q., Wang, H., Schwender, H., Zhang, T., Hetmanski, J.B., Chou, Y.H., Ye, X., Yeow, V., Chong, S.S., Zhang, B., et al. (2014). Joint testing of genotypic and gene-environment interaction identified novel association for BMP4 with non-syndromic CL/P in an Asian population using data from an International Cleft Consortium. PLoS One *9*, e109038.

Chen, R.E., and Thorner, J. (2007). Function and regulation in MAPK signaling pathways: lessons learned from the yeast Saccharomyces cerevisiae. Biochim. Biophys. Acta *1773*, 1311–1340.

Cohen, P. (2002). The origins of protein phosphorylation. Nat. Cell Biol. *4*, E127–E130.

Coles, E.G., Gammill, L.S., Miner, J.H., and Bronner-Fraser, M. (2006). Abnormalities in neural crest cell migration in laminin alpha5 mutant mice. Dev. Biol. *289*, 218–228.

Colwill, K., Pawson, T., Andrews, B., Prasad, J., Manley, J.L., Bell, J.C., and Duncan, P.I. (1996). The Clk/Sty protein kinase phosphorylates SR splicing factors and regulates their intranuclear distribution. EMBO J. *15*, 265–275.

Cowley, S., Paterson, H., Kemp, P., and Marshall, C.J. (1994). Activation of MAP kinase kinase is necessary and sufficient for PC12 differentiation and for transformation of NIH 3T3 cells. Cell *77*, 841–852.

(B) Cortical neurons were cultured for 21 days and treated with 10 μM MG132 and protein levels analyzed by immunoprecipitation and immunoblotting (REX1) and immunoblotting (RNF12 and ERK1/2).
(C) Cortical neurons were cultured *in vitro* for the indicated number of days (DIV) and RNF12 and MAP2 neuron specific marker analyzed by immunofluorescence. Scale bar: 20 μm.
(D) RNF12 SR-motif phosphorylation during *in vitro* mouse cortical neuron maturation was analyzed via phos-tag immunoblotting for RNF12. Fully phosphorylated (4-P) and unphosphorylated (0-P) RNF12 SR-motifs are indicated by open (○) and closed (●) circles, respectively. Synaptophysin and actin levels were determined by immunoblotting.
(E) Cortical neurons were cultured for 21 days and treated with 10 μM SRPKIN-1 for 4 h RNF12 SR-motif phosphorylation was analyzed by phos-tag immunoblotting for RNF12. Fully phosphorylated (4-P) and unphosphorylated (0-P) RNF12 SR-motif is indicated with open (○) by closed (●) circles, respectively. Synaptophysin and actin levels were determined by immunoblotting.
(F) The SRPK-RNF12-REX1 signaling pathway regulates neural gene expression and is disrupted in intellectual disability disorders. SRPK phosphorylates the RNF12 SR-motif to promote REX1 ubiquitylation and proteasomal degradation, which acts as a "brake" for neural gene expression in self-renewing pluripotent stem cells. In intellectual disability, inactivating mutations in SRPKs or RNF12 lead to REX1 accumulation and aberrant induction of neural genes.

CellPress
OPEN ACCESS

Dagher, S.F., and Fu, X.D. (2001). Evidence for a role of Sky1p-mediated phosphorylation in 3′ splice site recognition involving both Prp8 and Prp17/Slu4. RNA *7*, 1284–1297.

Deciphering Developmental Disorders Study. (2015). Large-scale discovery of novel genetic causes of developmental disorders. Nature *519*, 223–228.

Deciphering Developmental Disorders Study. (2017). Prevalence and architecture of de novo mutations in developmental disorders. Nature *542*, 433–438.

Ding, J.H., Zhong, X.Y., Hagopian, J.C., Cruz, M.M., Ghosh, G., Feramisco, J., Adams, J.A., and Fu, X.D. (2006). Regulated cellular partitioning of SR protein-specific kinases in mammalian cells. Mol. Biol. Cell *17*, 876–885.

Ding, L., Paszkowski-Rogacz, M., Nitzsche, A., Slabicki, M.M., Heninger, A.K., de Vries, I., Kittler, R., Junqueira, M., Shevchenko, A., Schulz, H., et al. (2009). A genome-scale RNAi screen for Oct4 modulators defines a role of the Paf1 complex for embryonic stem cell identity. Cell Stem Cell *4*, 403–415.

Dixon, J., Gladwin, A.J., Loftus, S.K., Riley, J.H., Perveen, R., Wasmuth, J.J., Anand, R., and Dixon, M.J. (1994). A YAC contig encompassing the Treacher Collins syndrome critical region at 5q31.3-32. Am. J. Hum. Genet. *55*, 372–378.

Dobin, A., Davis, C.A., Schlesinger, F., Drenkow, J., Zaleski, C., Jha, S., Batut, P., Chaisson, M., and Gingeras, T.R. (2013). STAR: ultrafast universal RNA-seq aligner. Bioinformatics *29*, 15–21.

Dou, H., Buetow, L., Sibbet, G.J., Cameron, K., and Huang, D.T. (2013). Essentiality of a non-RING element in priming donor ubiquitin for catalysis by a monomeric E3. Nat. Struct. Mol. Biol. *20*, 982–986.

Duprez, D., Leyns, L., Bonnin, M.A., Lapointe, F., Etchevers, H., De Robertis, E.M., and Le Douarin, N. (1999). Expression of Frzb-1 during chick development. Mech. Dev. *89*, 179–183.

Falcon, S., and Gentleman, R. (2007). Using GOstats to test gene lists for GO term association. Bioinformatics *23*, 257–258.

Fernandez-Alonso, R., Davidson, L., Hukelmann, J., Zengerle, M., Prescott, A.R., Lamond, A., Ciulli, A., Sapkota, G.P., and Findlay, G.M. (2017). Brd4-Brd2 isoform switching coordinates pluripotent exit and Smad2-dependent lineage specification. EMBO Rep. *18*, 1108–1122.

Frints, S.G.M., Ozanturk, A., Rodríguez Criado, G., Grasshoff, U., de Hoon, B., Field, M., Manouvrier-Hanu, S., E Hickey, S., Kammoun, M., Gripp, K.W., et al. (2019). Pathogenic variants in E3 ubiquitin ligase RLIM/RNF12 lead to a syndromic X-linked intellectual disability and behavior disorder. Mol. Psychiatry *24*, 1748–1768.

Fukuhara, T., Hosoya, T., Shimizu, S., Sumi, K., Oshiro, T., Yoshinaka, Y., Suzuki, M., Yamamoto, N., Herzenberg, L.A., Herzenberg, L.A., and Hagiwara, M. (2006). Utilization of host SR protein kinases and RNA-splicing machinery during viral replication. Proc. Natl. Acad. Sci. USA *103*, 11329–11333.

Fukumoto, S., Miner, J.H., Ida, H., Fukumoto, E., Yuasa, K., Miyazaki, H., Hoffman, M.P., and Yamada, Y. (2006). Laminin alpha5 is required for dental epithelium growth and polarity and the development of tooth bud and shape. J. Biol. Chem. *281*, 5008–5016.

Funnell, T., Tasaki, S., Oloumi, A., Araki, S., Kong, E., Yap, D., Nakayama, Y., Hughes, C.S., Cheng, S.-W.G., Tozaki, H., et al. (2017). CLK-dependent exon recognition and conjoined gene formation revealed with a novel small molecule inhibitor. Nat. Commun. *8*, 7.

Gabut, M., Samavarchi-Tehrani, P., Wang, X., Slobodeniuc, V., O'Hanlon, D., Sung, H.K., Alvarez, M., Talukder, S., Pan, Q., Mazzoni, E.O., et al. (2011). An alternative splicing switch regulates embryonic stem cell pluripotency and reprogramming. Cell *147*, 132–146.

Gammill, L.S., Gonzalez, C., Gu, C., and Bronner-Fraser, M. (2006). Guidance of trunk neural crest migration requires neuropilin 2/semaphorin 3F signaling. Development *133*, 99–106.

Gontan, C., Achame, E.M., Demmers, J., Barakat, T.S., Rentmeester, E., van IJcken, W., Grootegoed, J.A., and Gribnau, J. (2012). RNF12 initiates X-chromosome inactivation by targeting REX1 for degradation. Nature *485*, 386–390.

Gontan, C., Mira-Bontenbal, H., Magaraki, A., Dupont, C., Barakat, T.S., Rentmeester, E., Demmers, J., and Gribnau, J. (2018). REX1 is the critical target of RNF12 in imprinted X chromosome inactivation in mice. Nat. Commun. *9*, 4752.

Gopinathan, G., Foyle, D., Luan, X., and Diekwisch, T.G.H. (2019). The Wnt antagonist SFRP1: a key regulator of periodontal mineral homeostasis. Stem Cells Dev. *28*, 1004–1014.

Gou, L.T., Lim, D.H., Ma, W., Aubol, B.E., Hao, Y., Wang, X., Zhao, J., Liang, Z., Shao, C., Zhang, X., et al. (2020). Initiation of parental genome reprogramming in fertilized oocyte by splicing kinase SRPK1-catalyzed protamine phosphorylation. Cell *180*, 1212–1227.e14.

Grandbarbe, L., Bouissac, J., Rand, M., Hrabé de Angelis, M., Artavanis-Tsakonas, S., and Mohier, E. (2003). Delta-notch signaling controls the generation of neurons/glia from neural stem cells in a stepwise process. Development *130*, 1391–1402.

Gui, J.F., Lane, W.S., and Fu, X.D. (1994a). A serine kinase regulates intracellular localization of splicing factors in the cell cycle. Nature *369*, 678–682.

Gui, J.F., Tronchère, H., Chandler, S.D., and Fu, X.D. (1994b). Purification and characterization of a kinase specific for the serine- and arginine-rich pre-mRNA splicing factors. Proc. Natl. Acad. Sci. USA *91*, 10824–10828.

Hargrave, M., Wright, E., Kun, J., Emery, J., Cooper, L., and Koopman, P. (1997). Expression of the Sox11 gene in mouse embryos suggests roles in neuronal maturation and epithelio-mesenchymal induction. Dev. Dyn. *210*, 79–86.

Hatcher, J.M., Wu, G., Zeng, C., Zhu, J., Meng, F., Patel, S., Wang, W., Ficarro, S.B., Leggett, A.L., Powell, C.E., et al. (2018). SRPKIN-1: a covalent SRPK1/2 inhibitor that potently converts VEGF from pro-angiogenic to anti-angiogenic isoform. Cell Chem. Biol. *25*, 460–470.e6.

Hill, C.R., Yuasa, M., Schoenecker, J., and Goudy, S.L. (2014). Jagged1 is essential for osteoblast development during maxillary ossification. Bone *62*, 10–21.

Hodge, R.D., Bakken, T.E., Miller, J.A., Smith, K.A., Barkan, E.R., Graybuck, L.T., Close, J.L., Long, B., Johansen, N., Penn, O., et al. (2019). Conserved cell types with divergent features in human versus mouse cortex. Nature *573*, 61–68.

Hong, Y., Chan, C.B., Kwon, I.S., Li, X., Song, M., Lee, H.P., Liu, X., Sompol, P., Jin, P., Lee, H.-G., et al. (2012). SRPK2 phosphorylates tau and mediates the cognitive defects in Alzheimer's disease. J. Neurosci. *32*, 17262–17272.

Hu, H., Haas, S.A., Chelly, J., Van Esch, H., Raynaud, M., de Brouwer, A.P., Weinert, S., Froyen, G., Frints, S.G., Laumonnier, F., et al. (2016). X-exome sequencing of 405 unresolved families identifies seven novel intellectual disability genes. Mol. Psychiatry *21*, 133–148.

Huang, H., Yang, X., Bao, M., Cao, H., Miao, X., Zhang, X., Gan, L., Qiu, M., and Zhang, Z. (2016). Ablation of the Sox11 gene results in clefting of the secondary palate resembling the Pierre Robin sequence. J. Biol. Chem. *291*, 7107–7118.

Hulo, N., Bairoch, A., Bulliard, V., Cerutti, L., De Castro, E., Langendijk-Genevaux, P.S., Pagni, M., and Sigrist, C.J. (2006). The PROSITE database. Nucleic Acids Res. *34*, D227–D230.

Humphreys, R., Zheng, W., Prince, L.S., Qu, X., Brown, C., Loomes, K., Huppert, S.S., Baldwin, S., and Goudy, S. (2012). Cranial neural crest ablation of Jagged1 recapitulates the craniofacial phenotype of Alagille syndrome patients. Hum. Mol. Genet. *21*, 1374–1383.

Jang, S.W., Liu, X., Fu, H., Rees, H., Yepes, M., Levey, A., and Ye, K. (2009). Interaction of Akt-phosphorylated SRPK2 with 14-3-3 mediates cell cycle and cell death in neurons. J. Biol. Chem. *284*, 24512–24525.

Jia, S., Kwon, H.E., Lan, Y., Zhou, J., Liu, H., and Jiang, R. (2016). Bmp4-Msx1 signaling and Osr2 control tooth organogenesis through antagonistic regulation of secreted Wnt antagonists. Dev. Biol. *420*, 110–119.

Jiao, B., Taniguchi-Ishigaki, N., Güngör, C., Peters, M.A., Chen, Y.W., Riethdorf, S., Drung, A., Ahronian, L.G., Shin, J., Pagnis, R., et al. (2013). Functional activity of RLIM/Rnf12 is regulated by phosphorylation-dependent nucleocytoplasmic shuttling. Mol. Biol. Cell *24*, 3085–3096.

Kamalakar, A., Oh, M.S., Stephenson, Y.C., Ballestas-Naissir, S.A., Davis, M.E., Willett, N.J., Drissi, H.M., and Goudy, S.L. (2019). A non-canonical

JAGGED1 signal to JAK2 mediates osteoblast commitment in cranial neural crest cells. Cell. Signal. *54*, 130–138.

Kinoshita, E., Kinoshita-Kikuta, E., Takiyama, K., and Koike, T. (2006). Phosphate-binding tag, a new tool to visualize phosphorylated proteins. Mol. Cell. Proteomics *5*, 749–757.

Koizumi, J., Okamoto, Y., Onogi, H., Mayeda, A., Krainer, A.R., and Hagiwara, M. (1999). The subcellular localization of SF2/ASF is regulated by direct interaction with SR protein kinases (SRPKs). J. Biol. Chem. *274*, 11125–11131.

Lee, G., Zheng, Y., Cho, S., Jang, C., England, C., Dempsey, J.M., Yu, Y., Liu, X., He, L., Cavaliere, P.M., et al. (2017). Post-transcriptional regulation of de novo lipogenesis by mTORC1-S6K1-SRPK2 signaling. Cell *171*, 1545–1558.e18.

Leonardo, E.D., Hinck, L., Masu, M., Keino-Masu, K., Ackerman, S.L., and Tessier-Lavigne, M. (1997). Vertebrate homologues of C. elegans UNC-5 are candidate netrin receptors. Nature *386*, 833–838.

Li, B., Kuriyama, S., Moreno, M., and Mayor, R. (2009). The posteriorizing gene Gbx2 is a direct target of Wnt signalling and the earliest factor in neural crest induction. Development *136*, 3267–3278.

Li, H.B., Jin, X.Q., Jin, X., Guo, Z.H., Ding, X.H., Wang, Q., and Liu, R.Z. (2018). BMP4 knockdown of NCSCs leads to aganglionosis in the middle embryonic stage. Mol. Med. Rep. *17*, 5423–5427.

Lim, W.A., and Pawson, T. (2010). Phosphotyrosine signaling: evolving a new cellular communication system. Cell *142*, 661–667.

Long, J.C., and Caceres, J.F. (2009). The SR protein family of splicing factors: master regulators of gene expression. Biochem. J. *417*, 15–27.

Love, M.I., Huber, W., and Anders, S. (2014). Moderated estimation of fold change and dispersion for RNA-seq data with DESeq2. Genome Biol. *15*, 550.

Ma, C.T., Velazquez-Dones, A., Hagopian, J.C., Ghosh, G., Fu, X.D., and Adams, J.A. (2008). Ordered multi-site phosphorylation of the splicing factor ASF/SF2 by SRPK1. J. Mol. Biol. *376*, 55–68.

Mathew, R., Hartmuth, K., Möhlmann, S., Urlaub, H., Ficner, R., and Lührmann, R. (2008). Phosphorylation of human PRP28 by SRPK2 is required for integration of the U4/U6-U5 tri-snRNP into the spliceosome. Nat. Struct. Mol. Biol. *15*, 435–443.

Micaglio, E., Andronache, A.A., Carrera, P., Monasky, M.M., Locati, E.T., Pirola, B., Presi, S., Carminati, M., Ferrari, M., Giamberti, A., and Pappone, C. (2019). Novel JAG1 deletion variant in patient with atypical Alagille syndrome. Int. J. Mol. Sci. *20*.

Middeldorp, J., and Hol, E.M. (2011). GFAP in health and disease. Prog. Neurobiol. *93*, 421–443.

Nakagawa, O., Arnold, M., Nakagawa, M., Hamada, H., Shelton, J.M., Kusano, H., Harris, T.M., Childs, G., Campbell, K.P., Richardson, J.A., et al. (2005). Centronuclear myopathy in mice lacking a novel muscle-specific protein kinase transcriptionally regulated by MEF2. Genes Dev. *19*, 2066–2077.

Ngo, J.C., Giang, K., Chakrabarti, S., Ma, C.T., Huynh, N., Hagopian, J.C., Dorrestein, P.C., Fu, X.D., Adams, J.A., and Ghosh, G. (2008). A sliding docking interaction is essential for sequential and processive phosphorylation of an SR protein by SRPK1. Mol. Cell *29*, 563–576.

Ngo, J.C., Gullingsrud, J., Giang, K., Yeh, M.J., Fu, X.D., Adams, J.A., McCammon, J.A., and Ghosh, G. (2007). SR protein kinase 1 is resilient to inactivation. Structure *15*, 123–133.

Nikopoulos, G.N., Duarte, M., Kubu, C.J., Bellum, S., Friesel, R., Maciag, T., Prudovsky, I., and Verdi, J.M. (2007). Soluble Jagged1 attenuates lateral inhibition, allowing for the clonal expansion of neural crest stem cells. Stem Cells *25*, 3133–3142.

Niranjan, T.S., Skinner, C., May, M., Turner, T., Rose, R., Stevenson, R., Schwartz, C.E., and Wang, T. (2015). Affected kindred analysis of human X chromosome exomes to identify novel X-linked intellectual disability genes. PLoS One *10*, e0116454.

Okada, Y., and Hirokawa, N. (1999). A processive single-headed motor: kinesin superfamily protein KIF1A. Science *283*, 1152–1157.

Parada, C., Han, D., Grimaldi, A., Sarrión, P., Park, S.S., Pelikan, R., Sanchez-Lara, P.A., and Chai, Y. (2015). Disruption of the ERK/MAPK pathway in neural crest cells as a potential cause of Pierre Robin sequence. Development *142*, 3734–3745.

Pawellek, A., McElroy, S., Samatov, T., Mitchell, L., Woodland, A., Ryder, U., Gray, D., Lührmann, R., and Lamond, A.I. (2014). Identification of small molecule inhibitors of pre-mRNA splicing. J. Biol. Chem. *289*, 34683–34698.

Pilia, G., Uda, M., Macis, D., Frau, F., Crisponi, L., Balli, F., Barbera, C., Colombo, C., Frediani, T., Gatti, R., et al. (1999). Jagged-1 mutation analysis in Italian Alagille syndrome patients. Hum. Mutat *14*, 394–400.

Ponnusamy, M.P., Deb, S., Dey, P., Chakraborty, S., Rachagani, S., Senapati, S., and Batra, S.K. (2009). RNA polymerase II associated factor 1/PD2 maintains self-renewal by its interaction with Oct3/4 in mouse embryonic stem cells. Stem Cells *27*, 3001–3011.

Roosenboom, J., Lee, M.K., Hecht, J.T., Heike, C.L., Wehby, G.L., Christensen, K., Feingold, E., Marazita, M.L., Maga, A.M., Shaffer, J.R., Weinberg, S.M., et al. (2018). Mapping genetic variants for cranial vault shape in humans. PLoS One *13*, e0196148.

Roscigno, R.F., and Garcia-Blanco, M.A. (1995). SR proteins escort the U4/U6.U5 tri-snRNP to the spliceosome. RNA *1*, 692–706.

Salomonis, N., Schlieve, C.R., Pereira, L., Wahlquist, C., Colas, A., Zambon, A.C., Vranizan, K., Spindler, M.J., Pico, A.R., Cline, M.S., et al. (2010). Alternative splicing regulates mouse embryonic stem cell pluripotency and differentiation. Proc. Natl. Acad. Sci. USA *107*, 10514–10519.

Sela-Donenfeld, D., and Kalcheim, C. (1999). Regulation of the onset of neural crest migration by coordinated activity of BMP4 and Noggin in the dorsal neural tube. Development *126*, 4749–4762.

Serafini, T., Colamarino, S.A., Leonardo, E.D., Wang, H., Beddington, R., Skarnes, W.C., and Tessier-Lavigne, M. (1996). Netrin-1 is required for commissural axon guidance in the developing vertebrate nervous system. Cell *87*, 1001–1014.

Sgadò, P., Dunleavy, M., Genovesi, S., Provenzano, G., and Bozzi, Y. (2011). The role of GABAergic system in neurodevelopmental disorders: a focus on autism and epilepsy. Int. J. Physiol. Pathophysiol. Pharmacol. *3*, 223–235.

Shin, J., Bossenz, M., Chung, Y., Ma, H., Byron, M., Taniguchi-Ishigaki, N., Zhu, X., Jiao, B., Hall, L.L., Green, M.R., et al. (2010). Maternal Rnf12/RLIM is required for imprinted X-chromosome inactivation in mice. Nature *467*, 977–981.

Shin, J., Wallingford, M.C., Gallant, J., Marcho, C., Jiao, B., Byron, M., Bossenz, M., Lawrence, J.B., Jones, S.N., Mager, J., and Bach, I. (2014). RLIM is dispensable for X-chromosome inactivation in the mouse embryonic epiblast. Nature *511*, 86–89.

Shin, J.-O., Kim, E.-J., Cho, K.-W., Nakagawa, E., Kwon, H.-J., Cho, S.-W., and Jung, H.-S. (2012). BMP4 signaling mediates Zeb family in developing mouse tooth. Histochem. Cell Biol. *137*, 791–800.

Siebel, C.W., Feng, L., Guthrie, C., and Fu, X.D. (1999). Conservation in budding yeast of a kinase specific for SR splicing factors. Proc. Natl. Acad. Sci. USA *96*, 5440–5445.

Smith-Hicks, C.L. (2013). GABAergic dysfunction in pediatric neuro-developmental disorders. Front. Cell. Neurosci. *7*, 269.

Sock, E., Rettig, S.D., Enderich, J., Bösl, M.R., Tamm, E.R., and Wegner, M. (2004). Gene targeting reveals a widespread role for the high-mobility-group transcription factor Sox11 in tissue remodeling. Mol. Cell. Biol. *24*, 6635–6644.

Teng, C.S., Yen, H.Y., Barske, L., Smith, B., Llamas, J., Segil, N., Go, J., Sanchez-Lara, P.A., Maxson, R.E., and Crump, J.G. (2017). Requirement for Jagged1-Notch2 signaling in patterning the bones of the mouse and human middle ear. Sci. Rep. *7*, 2497.

Tønne, E., Holdhus, R., Stansberg, C., Stray-Pedersen, A., Petersen, K., Brunner, H.G., Gilissen, C., Hoischen, A., Prescott, T., Steen, V.M., and Fiskerstrand, T. (2015). Syndromic X-linked intellectual disability segregating with a missense variant in RLIM. Eur. J. Hum. Genet. *23*, 1652–1656.

Toyofuku, T., Yoshida, J., Sugimoto, T., Yamamoto, M., Makino, N., Takamatsu, H., Takegahara, N., Suto, F., Hori, M., Fujisawa, H., et al. (2008). Repulsive and attractive semaphorins cooperate to direct the navigation of cardiac neural crest cells. Dev. Biol. *321*, 251–262.

CellPress
OPEN ACCESS

Traverse, S., Gomez, N., Paterson, H., Marshall, C., and Cohen, P. (1992). Sustained activation of the mitogen-activated protein (MAP) kinase cascade may be required for differentiation of PC12 cells. Comparison of the effects of nerve growth factor and epidermal growth factor. Biochem. J. *288*, 351–355.

Varet, H., Brillet-Guéguen, L., Coppée, J.Y., and Dillies, M.A. (2016). SARTools: a DESeq2- and EdgeR-based R pipeline for comprehensive differential analysis of RNA-seq data. PLoS One *11*, e0157022.

Wang, H.Y., Lin, W., Dyck, J.A., Yeakley, J.M., Songyang, Z., Cantley, L.C., and Fu, X.D. (1998). SRPK2: a differentially expressed SR protein-specific kinase involved in mediating the interaction and localization of pre-mRNA splicing factors in mammalian cells. J. Cell Biol. *140*, 737–750.

Wang, X., and Astrof, S. (2016). Neural crest cell-autonomous roles of fibronectin in cardiovascular development. Development *143*, 88–100.

Wang, Z.H., Liu, P., Liu, X., Manfredsson, F.P., Sandoval, I.M., Yu, S.P., Wang, J.Z., and Ye, K. (2017). Delta-secretase phosphorylation by SRPK2 enhances its enzymatic activity, provoking pathogenesis in Alzheimer's disease. Mol. Cell *67*, 812–825.e5.

Wu, C.Y., and Taneyhill, L.A. (2012). Annexin a6 modulates chick cranial neural crest cell emigration. PLoS One *7*, e44903.

Wu, J.Y., and Maniatis, T. (1993). Specific interactions between proteins implicated in splice site selection and regulated alternative splicing. Cell *75*, 1061–1070.

Xiao, S.H., and Manley, J.L. (1997). Phosphorylation of the ASF/SF2 RS domain affects both protein-protein and protein-RNA interactions and is necessary for splicing. Genes Dev. *11*, 334–344.

Yeakley, J.M., Tronchère, H., Olesen, J., Dyck, J.A., Wang, H.Y., and Fu, X.D. (1999). Phosphorylation regulates in vivo interaction and molecular targeting of serine/arginine-rich pre-mRNA splicing factors. J. Cell Biol. *145*, 447–455.

Yen, H.Y., Ting, M.C., and Maxson, R.E. (2010). Jagged1 functions downstream of Twist1 in the specification of the coronal suture and the formation of a boundary between osteogenic and non-osteogenic cells. Dev. Biol. *347*, 258–270.

York, J.R., Yuan, T., Lakiza, O., and McCauley, D.W. (2018). An ancestral role for Semaphorin3F-Neuropilin signaling in patterning neural crest within the new vertebrate head. Development *145*.

Yu, H.H., and Moens, C.B. (2005). Semaphorin signaling guides cranial neural crest cell migration in zebrafish. Dev. Biol. *280*, 373–385.

Zhang, L., Huang, H., Zhou, F., Schimmel, J., Pardo, C.G., Zhang, T., Barakat, T.S., Sheppard, K.A., Mickanin, C., Porter, J.A., et al. (2012). RNF12 controls embryonic stem cell fate and morphogenesis in zebrafish embryos by targeting Smad7 for degradation. Mol. Cell *46*, 650–661.

Zhang, Y.B., Hu, J., Zhang, J., Zhou, X., Li, X., Gu, C., Liu, T., Xie, Y., Liu, J., Gu, M., et al. (2016). Genome-wide association study identifies multiple susceptibility loci for craniofacial microsomia. Nat. Commun. *7*, 10605.

Zhu, S., Liu, W., Ding, H.F., Cui, H., and Yang, L. (2019). BMP4 and neuregulin regulate the direction of mouse neural crest cell differentiation. Exp. Ther. Med. *17*, 3883–3890.

Zuniga, E., Stellabotte, F., and Crump, J.G. (2010). Jagged-notch signaling ensures dorsal skeletal identity in the vertebrate face. Development *137*, 1843–1852.

STAR★METHODS

KEY RESOURCES TABLE

REAGENT or RESOURCE	SOURCE	IDENTIFIER
Antibodies		
RNF12	Novus Biologicals	Cat#H00051132-M01; RRID: AB_547742
ERK1	BD Biosciences	Cat#610408; RRID: AB_397790
SRPK1	BD Biosciences	Cat#611072; RRID: AB_398385
SRPK2	BD Biosciences	Cat#611118; RRID: AB_398429
HA-tag	Abcam	Cat#ab9110; RRID: AB_307019
REX1	Abcam	Cat#ab28141; RRID: AB_882332
SRPK3	R&D Systems	Cat#MAB7230-SP
FLAG	Sigma Aldrich	Cat#F1804-50UG; RRID: AB_262044
HA-HRP	Roche	Cat#12013819001; RRID: AB_390917
Synaptophysin	Cell Signaling Technologies	Cat#5461 (D35E4); RRID: AB_10698743
beta-actin	Cell Signaling Technologies	Cat#4970 (13E5); RRID: AB_2223172
RNF12 (1-271)	MRC-PPU Reagents and Services	Cat#S691D third bleed
RNF12 pSer212/214 (QRRARpSRpSPEHRR)	MRC-PPU Reagents and Services	Cat#SA310 fourth bleed
GST	MRC-PPU Reagents and Services	Cat#S902A third bleed
Phosphoepitope SR proteins	Millipore	Cat#MABE50 clone 1H4; RRID: AB_10807429
KLF4	R&D Systems	Cat#AF3158; RRID: AB_2130245
MAP2	Sigma Aldrich	Cat#M2320; RRID: AB_609904
HA-tag	Sigma Aldrich	Cat#A2095 agarose beads; RRID: AB_257974
Chemicals, Peptides, and Recombinant Proteins		
AZ191	Tocris	Cat#5232
KH-CB19	Merck Millipore	Cat#219511
CLK-IN-T3	Aobious	Cat#AOB8827
SPHINX31	Axon Medchem	Cat#Axon 2714
CHIR99021	Axon Medchem	Cat#Axon 1386
PD0325901	Axon Medchem	Cat#Axon 1408
VX-745	Selleckchem	Cat#S1458
JNK-IN-8	Selleckchem	Cat#S4901
RO-3306	Sigma Aldrich	Cat#SML0569
Flavopiridol	Stratech	Cat#S2679
CCT241533	Cayman	Cat#CAY19178
Harmine	Sigma Aldrich	Cat#286044
WEHI-345	Cayman	Cat#CAY23023
IRAK-4 Ina	MRC-PPU Reagents and Services	N/A
GSK461364	Cayman	Cat#CAY18099
SRPIN340	Sigma Aldrich	Cat#SML1088
MG132	Sigma Aldrich	Cat#C2211
Cycloheximide	Sigma Aldrich	Cat#C7698
Leptomycin B	Sigma Aldrich	Cat#L2913
Madrasin (DDD00107587)	Kind gift of Dr. Andrea Pawellek (University of Dundee)	N/A
Phos-Tag	MRC-PPU Reagents and Services	N/A
RNF12	MRC-PPU Reagents and Services	DU61098
RNF12 S212A S214A S227A S229A	MRC-PPU Reagents and Services	DU53249
DYRK1a	MRC-PPU Reagents and Services	DU19040

(*Continued on next page*)

CellPress
OPEN ACCESS

Continued

REAGENT or RESOURCE	SOURCE	IDENTIFIER
CLK2	MRC-PPU Reagents and Services	DU16987
GSK3beta	MRC-PPU Reagents and Services	DU899
ERK1 (MAPK3)	MRC-PPU Reagents and Services	DU1509
ERK2 (MAPK1)	MRC-PPU Reagents and Services	DU650
JNK3 alpha 1 (SAPK1b)	MRC-PPU Reagents and Services	DU1511
p38 alpha (SAPK2a)	MRC-PPU Reagents and Services	DU979
CDK2 - CyclinA	MRC-PPU Reagents and Services	DU43557
CDK5 - p35	MRC-PPU Reagents and Services	DU39816
CDK7 - MAT1 - Cyclin H	MRC-PPU Reagents and Services	DU49574
CDK9 - Cyclin T1	MRC-PPU Reagents and Services	DU31050
REX1	MRC-PPU Reagents and Services	DU53244
SMAD7	MRC-PPU Reagents and Services	DU19219
SRPK1	MRC-PPU Reagents and Services	DU967
SRPK2	MRC-PPU Reagents and Services	DU36135
SRPK3	MRC-PPU Reagents and Services	DU967
SRPK1 D497A	MRC-PPU Reagents and Services	DU66208
SRPK2 D541A	MRC-PPU Reagents and Services	DU66209
SRPK3 H159D	MRC-PPU Reagents and Services	DU61121
SRPK3 T211M	MRC-PPU Reagents and Services	DU61140
SRPK3 K270M	MRC-PPU Reagents and Services	DU61135
Ube1	MRC-PPU Reagents and Services	DU32888
UBE2D1 (UbcH5a)	MRC-PPU Reagents and Services	DU4315
FLAG-Ubiquitin	MRC-PPU Reagents and Services	DU46789
Ubiquitin	MRC-PPU Reagents and Services	DU20027
Ubiquitin IR-800	Walden lab (Uni of Glasgow)	N/A
Critical Commercial Assays		
SRPKIN-1 kinase inhibitor profiling	This paper	http://www.kinase-screen.mrc.ac.uk/services/express-screen
Deposited Data		
Raw RNA-SEQ data	This paper	GEO: GSE149554
Raw Brain Cortex Single Nucleus RNA-SEQ data	Hodge et al., 2019	dbGAP: phs001790
Raw hiPSC mass-spectrometry data	Brenes et al., 2020	PRIDE: PXD010557
Experimental Models: Cell Lines		
Mouse: Embryonic Stem Cells	Laboratory of Janet Rossant, SickKids Research Institute, Toronto	CCE line
Human: induced Pluripotent Stem Cells	Cellartis AB Human Pluripotent Stem Cell Core Facility, School of Life Sciences, University of Dundee	CHiPS4 line
Human: Neuro2A	ATCC	Cat#CCL-131
Human: HEK 293	ATCC	Cat#CRL-1573
Human: U2OS	ATCC	Cat#HTB-96
Human: MCF7	ATCC	Cat#CRL-3435
Experimental Models: Organisms/Strains		
Mouse: C57B6/J	Animal Facility, School of Life Sciences, University of Dundee	N/A
Oligonucleotides		
Primers for qRT-PCR, see Table S8	This paper	N/A
Primers for genomic DNA sequencing, see Table S8	This paper	N/A
gRNA sequences for CRISPR/Cas9, see Table S8	This paper	N/A

(*Continued on next page*)

Continued		
REAGENT or RESOURCE	SOURCE	IDENTIFIER
ON-TARGETplus Srpk2 siRNA 06	Horizon Discovery	Cat#J-055142-06-0010
Non-targeting Pool siRNA	Horizon Discovery	Cat#D-001810-10-05
Recombinant DNA		
pCAGGS PURO RNF12	MRC-PPU Reagents and Services	DU50610
pCAGGS PURO RNF12 S212A	MRC-PPU Reagents and Services	DU53528
pCAGGS PURO RNF12 S214A	MRC-PPU Reagents and Services	DU50796
pCAGGS PURO RNF12 S227A	MRC-PPU Reagents and Services	DU53591
pCAGGS PURO RNF12 S229A	MRC-PPU Reagents and Services	DU53592
pCAGGS PURO RNF12 S212A S214A	MRC-PPU Reagents and Services	DU53518
pCAGGS PURO RNF12 S227A S229A	MRC-PPU Reagents and Services	DU53514
pCAGGS PURO RNF12 S214A S229A	MRC-PPU Reagents and Services	DU53593
pCAGGS PURO RNF12 S212A S214A S227A S229A	MRC-PPU Reagents and Services	DU50797
pCAGGS PURO RNF12 delta SR-motif	MRC-PPU Reagents and Services	DU53413
pCAGGS PURO HA-RNF12	MRC-PPU Reagents and Services	DU50854
pCAGGS PURO HA-RNF12 S212A S214A S227A S229A	MRC-PPU Reagents and Services	DU58741
pCAGGS PURO RNF12 W576Y	MRC-PPU Reagents and Services	DU50800
pCAGGS PURO FLAG SRPK1	MRC-PPU Reagents and Services	DU53820
pCAGGS PURO FLAG SRPK2	MRC-PPU Reagents and Services	DU53821
pKN7 RLIM ex5 KO Sense A	MRC-PPU Reagents and Services	DU52037
pX335 RLIM ex5 KO antisense A + Cas9n	MRC-PPU Reagents and Services	DU52046
pBabeD P U6 RLIM (mouse) Cter KI Sense A	MRC-PPU Reagents and Services	DU57881
pX335 RLIM (mouse) Cter KI AntiSense A	MRC-PPU Reagents and Services	DU57891
pMA RLIM Cter R575C IRES-GFP donor	MRC-PPU Reagents and Services	DU57963
pMA RLIM Cter del 206-229 IRES-GFP donor	MRC-PPU Reagents and Services	DU57964
pMA RLIM Cter S212A S214A S227A S229A IRES-GFP donor	MRC-PPU Reagents and Services	DU57966
pMA RLIM Cter wt control IRES-GFP donor	MRC-PPU Reagents and Services	DU57967
pMA RLIM Cter W576Y IRES-GFP donor	MRC-PPU Reagents and Services	DU60290
pBabeD P U6 Srpk1 (mouse) ex3 KO Sense B	MRC-PPU Reagents and Services	DU60949
pX335 Srpk1 (mouse) ex3 KO Antisense B	MRC-PPU Reagents and Services	DU64462
pBabeD P U6 SRPK2 (mouse) ex5 KO Sense A	MRC-PPU Reagents and Services	DU64247
pX335 SRPK2 (mouse) ex5 KO Antisense A	MRC-PPU Reagents and Services	DU 64251
pBabeD P U6 ZFP42 (mouse) ex4 KO Sense A	MRC-PPU Reagents and Services	DU60065
pX335 ZFP42 (mouse) ex4 KO AntiSense A	MRC-PPU Reagents and Services	DU60072
Software and Algorithms		
ScanProsite	Hulo et al., 2006	https://prosite.expasy.org/scanprosite/
Image Studio	LICOR Biosciences	https://www.licor.com/bio/image-studio/
Image Lab	Bio-Rad	https://www.bio-rad.com/en-uk/product/image-lab-software?ID=KRE6P5E8Z
Kinoviewer	Brenes and Lamond, 2019	https://peptracker.com
Proteome Discoverer v.2.0	Thermofisher	https://www.thermofisher.com/order/catalog/product/OPTON-30812?SID=srch-srp-OPTON-30812#/OPTON-30812?SID=srch-srp-OPTON-30812
Mascot	Matrix Science	https://www.matrixscience.com/server.html
Image J	NIH	https://imagej.nih.gov/ij/download.html
STAR software (v2.7.1a)	Dobin et al., 2013	https://github.com/alexdobin/STAR
HTSeq (v0.11.2)	Anders et al., 2015	https://htseq.readthedocs.io/en/master/

(*Continued on next page*)

Continued

REAGENT or RESOURCE	SOURCE	IDENTIFIER
SARTools (v1.6.9)	Varet et al., 2016	https://github.com/PF2-pasteur-fr/SARTools
DESeq2 (v1.24)	Love et al., 2014	https://bioconductor.org/packages/release/bioc/html/DESeq2.html
GOstats (v2.50.0)	Falcon and Gentleman, 2007	https://www.bioconductor.org/packages/release/bioc/html/GOstats.html
GraphPad Prism (v7.0c)	GraphPad Software Inc.	https://www.graphpad.com/scientific-software/prism/

RESOURCE AVAILABILITY

Lead Contact

Further information and requests for resources and reagents should be directed to and will be fulfilled by the Lead Contact, Greg Findlay (g.m.findlay@dundee.ac.uk).

Materials Availability

Plasmids and antibodies generated in this study have been deposited to MRC-PPU Reagents & Services (http://mrcppureagents.dundee.ac.uk/).

Data and Code Availability

The accession number for the RNA sequencing dataset generated during this study is Gene Expression Omnibus (GEO): GSE149554 (https://www.ncbi.nlm.nih.gov/geo/query/acc.cgi?acc=GSE149554).

Original source data have been deposited to Mendeley Data: https://doi.org/10.17632/phjvpdzp57.1

EXPERIMENTAL MODEL AND SUBJECT DETAILS

Cell Lines

Mouse Embryonic Stem Cells (mESCs)

Wild-type and CRISPR Cas9 edited male mESCs (CCE line) were cultured in 0.1% gelatin [w/v] coated plates in DMEM containing 10% foetal calf serum [v/v], 5% Knock-Out serum replacement [v/v], 2 mM glutamine, 0.1 mM MEM non-essential amino acids, 1mM sodium pyruvate, and penicillin/streptomycin (all from Thermo Fisher Scientific), 0.1 mM beta-mercaptoethanol (Sigma Aldrich), and 100 ng/ml GST-tagged Leukaemia inhibitory factor (LIF) at 5% CO_2 and 37°C. For 2i culture, mESCs were converted from LIF/FBS to 2i culture media composed of N2B27: 1% B27 supplement [v/v], 0.5% N2 supplement [v/v], 2 mM glutamine (all from Thermo Fisher Scientific), 0.1 mM β-mercaptoethanol (Sigma Aldrich), and penicillin/streptomycin in 1:1 DMEM/F12:Neurobasal medium (both from Thermo Fisher Scientific with 1 μM PD0325901 and 1 μM CHIR99021) and neural differentiation induced by culturing cells in N2B27. Cells were routinely authenticated via morphology and pluripotency gene expression analysis.

Human Induced Pluripotent Stem Cells (hiPSCs)

hiPSCs (CHiPS4 male cell line) were cultured in feeder-free conditions in TeSR medium supplemented with Noggin (10 ng/ml, Peprotech) and bFGF (30 ng/ml, Peprotech) on plates coated with Geltrex matrix (20 μg/cm^2, Life Technologies) at 5% CO_2 and 37°C. Cells were routinely authenticated via morphology and pluripotency gene expression analysis

Other Mammalian Cell Lines

Male mouse Neuro 2a and female human U2OS, HEK 293 and MCF7 cell lines were grown in DMEM containing 10% foetal calf serum [v/v] at 5% CO_2 and 37°C. Cells were routinely authenticated via morphology analysis.

Animal Studies

Primary Mouse Cortical Neurons

E16.5 C57BL/6 female and male mice brains were placed in ice cold HBSS, meninges removed, and cortex dissected. Cortex tissue was incubated with 0.125% trypsin containing DNAse at 37°C for 30 minutes. Samples were centrifuged at 1,200 rpm for 5 minutes and resuspended in complete Neurobasal media (Neurobasal containing 2 mM Glutamax, 2% B27 supplement [v/v], 10% foetal calf serum [v/v] and penicillin/streptomycin) and filtered through a 40 μm pore filter. Cells were then centrifuged for 7 minutes at 700 rpm, resuspended in complete Neurobasal media and plated at 0.5 x 10^6 cells/well on 6-well plates coated with 0.1 mg/ml poly-L-lysine (PLL; Sigma Aldrich). Neurons were cultured at 37°C in a humidified incubator with 5% CO_2 and medium replaced every 5 days with fresh medium containing B27.

Mouse Organs

19-week-old male C57BL/6J mice were dissected, organs collected and wrapped in tinfoil and snap frozen in liquid nitrogen. Organs were then resuspended in lysis buffer and lysed using a Polytrone PT 1200 E homogeniser (Kinematica, Littau-Lucerne, Switzerland) on ice. Samples were then clarified for 20 min at 14,000 rpm at 4°C and subjected to immunoblot analysis.

Ethics

Mouse studies were approved by the University of Dundee ethical review committee, and further subjected to approved study plans by the Named Veterinary Surgeon and Compliance Officer (Dr. Ngaire Dennison) and performed under a UK Home Office project licence in accordance with the Animal Scientific Procedures Act (ASPA, 1986). Mice were housed in a SPF facility in temperature-controlled rooms at 21°C, with 45-65% relative humidity and 12-hour light/dark cycles. Mice had *ad libitum* access to food and water and regularly monitored by the School of Life Science Animal Unit Staff.

METHOD DETAILS

Serine-Arginine Motif Search

Proteins containing tandem Serine-Arginine motifs were identified by searching the ScanProsite tool (Hulo et al., 2006) (ExPASy, Swiss Institute of Bioinformatics) for a R-S-R-S-x(0,20)-R-S-R-S motif where x is any amino acid and (0,20) the numerical range of intervening amino acids. The motif was searched against the UniProtKB database for *Mus musculus* proteome (TaxID: 10090). The resulting proteins were categorised according to UniProt functional description and listed in Table S1.

Plasmid and siRNA Transfection

mESCs were transfected with Lipofectamine LTX (Thermo Fisher Scientific) according to manufacturer instructions. All cDNA plasmids generated and used in this study are summarised in the Key Resource Table and can be found at MRC-PPU Reagents and services website http://mrcppureagents.dundee.ac.uk/. mESCs were transfected with siRNA using Lipofectamine RNAiMAX reagent (Thermo Fisher Scientific). siRNA oligos are listed in the Key Resource Table.

CRISPR/Cas9 Gene Editing

Rlim$^{-/y}$ mESCs were described previously (Bustos et al., 2018). To generate CRISPR Cas9 knockout mESC lines wild-type (for Srpk1 and Srpk2) or *Rlim*$^{-/y}$ (for Zfp42) mESCs were transfected with pX335 and pKN7 vectors containing gRNA sequences targeting *Srpk1* exon 3, *Srpk2* exon 5 or *Zfp42* exon 4 (detailed in Key Resource Table). *Rlim* WT-IRES-GFP (RNF12 WT-KI) and R575C-IRES-GFP (RNF12 R575C-KI) knock-in mESCs were described previously (Bustos et al., 2018). To generate *Rlim* S212A S214A S227A S229A-IRES-GFP (RNF12 4xSA-KI), *Rlim* with amino acids 206-229 deleted IRES-GFP (RNF12 ΔSR-KI) and *Rlim* W576Y-IRES-GFP (RNF W576Y-KI) knock-in mESC lines, wild-type mESCs were transfected with pBABED Puro U6 and pX335 vectors encoding guide RNAs targeting *Rlim* gene (detailed in Key Resource Table) together with donor pMa vectors containing DNA sequence encoding RNF12 amino acids 84 to 600 harbouring the desired mutations followed by an IRES (internal ribosome entry site) and EGFP. Transfected cells were selected with 3 μg/ml puromycin for 48 h and subjected to single cell sorting. Expanded knock-out single mESC clones were screened via immunoblot. EGFP positive knock-in single mESCs were expanded and screened for EGFP expression and RNF12 size or phosphorylation via immunoblot. Mutations were confirmed by genomic DNA sequencing. All cDNA plasmids are detailed in the Key Resource Table. Guide RNA and primer sequences are detailed in Table S8.

Pharmacological Inhibitors

All compounds were diluted in DMSO and mESCs treated with 10 μM inhibitor for 4 h prior lysis unless indicated otherwise. For protein stability assays, protein synthesis was inhibited by treating mESCs with 350 μM cycloheximide (Sigma Aldrich). For proteasome inhibition mESCs were treated with 10 μM MG132 (Sigma Aldrich) for 6 h. All chemicals are listed in the Key Resource Table.

Kinase Inhibitor Profiling

SRPKIN-1 inhibition activity was analysed using *in vitro* kinase assays for 50 representative kinases (MRC-PPU International Centre for Kinase Profiling). Kinase activity towards specific peptides was assessed in comparison to DMSO control. Full details are available at http://www.kinase-screen.mrc.ac.uk/services/express-screen.

Immunoblotting and Phos-Tag Analysis

SDS-PAGE electrophoresis and immunoblotting was performed using standard methods. Cells were lysed in lysis buffer (20 mM Tris [pH 7.4], 150 mM NaCl, 1 mM EDTA, 1% NP-40 [v/v], 0.5% sodium deoxycholate [w/v], 10 mM β-glycerophosphate, 10 mM sodium pyrophosphate, 1 mM NaF, 2 mM Na_3VO_4, and Roche Complete Protease Inhibitor Cocktail Tablets). Phospho-specific antibodies were used at 1 μg/ml with 10 μg/ml of the corresponding non-phosphopeptide. After secondary antibody incubation, membranes were subjected to chemiluminescence detection with Immobilon Western Chemiluminescent HRP Substrate (Millipore) using a Gel-Doc XR+ System (Bio-Rad) or Infrared detection using a LI-COR Odyssey Clx system. REX1 protein levels were determined by immunoblotting REX1 immunoprecipitates using Clean-Blot IP Detection Reagent (Thermo Fisher Scientific).

Phos-tag analyses were performed by loading protein samples containing 10 mM $MnCl_2$ in 8% polyacrylamide gels containing 50 μM Phos-tag reagent (MRC-PPU reagents and services) and 0.1 mM $MnCl_2$. After electrophoresis, gels were washed three times

for 10 mins in Transfer buffer (48 mM Tris, 39 mM Glycine, 20% Methanol) supplemented with 20 mM EDTA. Proteins were then transferred to Nitrocellulose membranes, blocked and probed with the indicated antibodies. All protein signals were quantified using Image Studio (LI-COR Biosciences) or Image Lab software (Bio-Rad). Primary antibodies are listed in the Key Resource Table.

Mass Spectrometry

For phospho-site identification samples were separated via SDS-PAGE electrophoresis, stained with Coomassie blue and gel pieces subjected to an in-gel digestion. First, gel pieces were washed in water, 50% acetonitrile (ACN)/water, 0.1 M NH_4HCO_3 and 50% ACN/50 mM NH_4HCO_3 and then with 10 mM DTT/0.1 M NH_4HCO_3 (All from Sigma-Aldrich). Proteins were alkylated with 50 mM iodoacetamide/0.1 M NH_4HCO_3 and then washed as above. Gel pieces were then shrunk in ACN and dried using Speed-Vac. Proteins were then trypsinised by incubating with 5 μg/ml trypsin in 25 mM triethylammonium bicarbonate (Sigma-Aldrich) overnight. Supernatants were separated and gel pieces resuspended in 50% ACN/2.5% formic acid and supernatants combined. Samples were then dried via Speed-Vac and then resuspended in 30 μl 0.1 % formic acid and subjected to liquid chromatography–mass spectrometry (LC-MS) analysis using an Ultimate 3000 RSLCnano system coupled to LTQ-Orbitrap VelosPro mass spectrometer (ThermoFisher Scientific) 10 μl samples were injected and peptides were loaded onto a nanoViper C18 Trap column (5 μm particle size, 100 μm x 2 cm) and separated in a C18 reversed phase Easy-spray column (2 μm particle size, 75 μm x 50 cm) (ThermoFisher Scientific) at a flow rate of 300 nl/min. A linear gradient was used, starting at 3% B and maintained for 5 min, from 3-35% B in 40 min, 35-99% B for 2 min, maintained at 99% B for 5 min, 99-3% B in 3 min and maintained at 3% B for 5 min. Solvents used were A: 0.1% formic acid and B: 80% acetonitrile (ACN) with 0.08% formic acid.

Mass Spectrometry data was acquired in data-dependent mode using the following parameters: MS1 spectra were acquired in the Orbitrap at a resolution of 60,000 (at 400 m/z) for a mass range of 375-1600 m/z with a FTMS full AGC target of 1e6. The top 20 most intense ions (with a minimal signal threshold of 2000) were selected for MS2 analysis on the linear ion trap (with a full AGC target of 5,000) and were fragmented (using CID with a collision energy of 35%), multistage activation, and neutral loss masses of 24.4942, 32.6590, 48.9885.

Data was analysed using Proteome Discoverer v.2.0 and Mascot using MRC_Database_1 (1,950 sequences). Parameters used were the following: Variable modifications: Oxidation (M), Dioxidation (M), Phospho (STY); Fixed modifications: Carbamidomethyl (C), Enzyme: Trypsin/P, Maximum missed cleavages: 3, Precursor tolerance: 10ppm, MS2 tolerance: 0.6Da, Minimum score peptides: 18. Phospho-site assignment probability was estimated via Mascot and PhosphoRS3.1 (Proteome Discoverer v.1.4-SP1) or ptmRS (Proteome Discoverer v.2.0).

Quantitative total mESC proteomics data covering around 10,000 proteins was previously described (Fernandez-Alonso et al., 2017). CMGC kinase expression from that dataset was generated using Kinoviewer (https://peptracker.com) (Brenes and Lamond, 2019). Quantitative total proteomics data from human induced pluripotent stem cells (hiPSC, bubh_3 line) was obtained from the human induced pluripotent stem cell initiative (HipSci) database (Brenes et al., 2020).

Protein Expression and Purification

All recombinant proteins were produced in *E. coli* or SF21 insect cells expression systems by MRC-PPU reagents and services and purified via standard protocols. Proteins used in this study are listed in the Key Resource Table and can be found at the MRC-PPU Reagents and services website http://mrcppureagents.dundee.ac.uk/.

In Vitro Kinase Assays

For SRPK Immunoprecipitation kinase assays, mESCs were treated with 10 μM SRPKIN-1 for 4 h. Cells were lysed, and 1.5 mg of protein immunoprecipitated with 2 μg of SRPK1 or SRPK2 antibodies (BD Biosciences). Immunoprecipitates were then washed with lysis buffer supplemented with 500 mM NaCl and half of the sample was resuspended in loading buffer. The remainder was subjected to *in vitro* phosphorylation assay containing 0.5 μg RNF12 and 2 mM ATP in kinase buffer (50 mM Tris-HCl [pH 7.5], 0.1 mM EGTA, 10 mM MgCl2, 2 mM DTT) and incubated at 30°C for 30 min. SRPK *in vitro* kinase assays were performed by incubating 200 mU kinase or equivalent μg of inactive kinase with 0.5 μg RNF12 and 2 mM ATP in kinase buffer. For radioactive *in vitro* kinase assays, reactions were supplemented with 1 μCi γ-^{32}P ATP. Reactions were incubated at 30°C for 30 min in presence or absence of inhibitor as indicated and samples subjected to polyacrylamide electrophoresis and immunoblot or Coomassie blue staining and signal detected via ECL, infrared detection or autoradiography.

Immunofluorescence

Immunofluorescence and confocal analysis were performed as described. mESCs were plated in 0.1% gelatin [v/v] coated coverslips. Cortical neurons were plated at a density of 1.5 x 10^5 cells/well on poly-L-lysine German Glass Coverslips 18mm #1½ (EMS-diasum). Primary antibodies used are listed in the Key Resource Table. Cells were mounted using Fluorsave reagent (Millipore) Images were acquired in a Zeiss 710 confocal microscope and images were processed using Image J (NIH) and Photoshop CS5.1 software (Adobe). Nuclear and cytosolic staining intensity was determined using ImageJ (NIH).

In Vitro Phospho-RNF12 Activity Assays

For substrate ubiquitylation assays, 0.5 μg RNF12 protein was subjected to a phosphorylation reaction containing 200 mU SRPK or equivalent μg of catalytically inactive kinase and 2 mM ATP in kinase buffer for 1 h at 37°C. 200 nM phosphorylated RNF12 was then

incubated with a ubiquitylation mix containing 1.5 μg of REX1 or SMAD7, 0.1 μM UBE1, 0.05 μM UBE2D1, 2 μM Ub-IR^{800}, 0.5 mM TCEP [pH 7.5], 5 mM ATP (both from Sigma Aldrich), 50 mM Tris-HCl [pH 7.5], 5 mM $MgCl_2$ for 30 min at 30°C. Reactions were stopped with SDS sample buffer and boiled for 5 min. Samples were loaded in 4-12% Bis-Tris gradient gels (Thermo Fisher Scientific). Gels were then scanned using an Odyssey CLx Infrared Imaging System (LICOR Biosciences) for detection of fluorescently labelled ubiquitylated proteins. After scanning proteins were transferred to PVDF or nitrocellulose membranes and analysed via immunoblot and signal detected using ECL or infrared detection.

For UBE2D1 ubiquitin discharge assays 5 μg RNF12 protein was phosphorylated as above with 2U SRPK or equivalent μg of catalytically inactive kinase and 2 mM ATP in kinase buffer for 1 h at 37°C. ATP was depleted with 4.5 U/ml apyrase (New England Biolabs) for 10 min at room temperature. UBE2D1-ubiquitin thioester was prepared by incubating 100 μM UBE2D1 with 0.2 μM UBE1, 100 μM FLAG-ubiquitin, 3 mM ATP, 0.5 mM TCEP [pH 7.5] (both from Sigma Aldrich), 5 mM MgCl2, 50 mM Tris (pH 7.5), 150 mM NaCl for 20 min at 37 °C. The reaction was stopped by depleting ATP with 4.5 U/ml apyrase (New England Biolabs) for 10 min at room temperature. Then, 40 μM UBE2D1-ubiquitin were incubated with 1 μM phosphorylated RNF12 and 150 mM L-lysine in a buffer containing 50 mM Tris [pH 7.5], 150 mM NaCl, 0.5 mM TCEP, 0.1% [v/v] NP40 at room temperature. Reactions were stopped with non-reducing SDS loading buffer and analysed via immunoblotting and membranes scanned in an Odyssey CLx Infrared Imaging System (LI-COR Biosciences). Protein signals were quantified using Image Studio software (LI-COR Biosciences). Reaction rates were determined by extrapolating protein signals in a standard curve of known concentrations of UBE2D1-ubiquitin conjugate and plotting concentration over time.

Binding Assays

For protein immunoprecipitation, protein A or G beads were incubated with 2 μg antibody and 0.5-2 μg/μl protein sample in lysis buffer overnight at 4°C. Immunoprecipitates were then washed three times with lysis buffer supplemented with 500 μM NaCl, resuspended in 50% [v/v] loading buffer and boiled at 95°C for 5 minutes prior to immunoblotting analysis. For HA tagged protein immunoprecipitation, Anti-HA agarose conjugate (Sigma Aldrich) was used.

For GST pulldown assays, 0.5 μg of RNF12 was phosphorylated with 200 mU SRPK (or 19 ng SRPK1 WT or KD; 60 ng SRPK2 WT or KD) in presence of 2 mM ATP in kinase buffer for 1 h at 37°C. ATP was depleted with 4.5 U/ml apyrase (New England Biolabs) for 10 min at room temperature and samples mixed with 0.5 μg REX1 protein in 500 μl GST pulldown buffer (10 mM Tris pH=8.0, 150 mM NaCl, 10% Glycerol, 0.1% Triton X-100, and Roche Complete Protease Inhibitor Cocktail Tablets) overnight at 4°C. Complexes were then pulled down using GSH Sepharose 4B beads (Sigma Aldrich) for 2 h at 4°C. Beads were then washed and samples analysed by immunoblotting.

RNA-Sequencing and Gene Ontology Analysis

Total RNA was extracted using RNeasy Mini Kit (QIAGEN) and DNA libraries prepared using TruSeq Stranded Total RNA Sample Preparation kits (Illumina) according to manufacturer's instructions. Sequencing was performed on Illumina NextSeq platform. Briefly, raw sequencing reads were trimmed by removing Illumina adapters sequences and low-quality bases. Trimmed reads were mapped using to mouse reference genome (mm10) using STAR software (v2.7.1a) (Dobin et al., 2013). The number of reads per transcript was counted using HTSeq (v0.11.2) (Anders et al., 2015). The differentially expressed genes (DEGs) were estimated using SARTools (v1.6.9) (Varet et al., 2016) and DESeq2 (v1.24) (Love et al., 2014) R packages. Gene Ontology (GO) analysis was carried out using the GOstats (v2.50.0) R package (Falcon and Gentleman, 2007). Raw and processed data can be accessed at Gene Expression Omnibus, GEO: GSE149554 (https://www.ncbi.nlm.nih.gov/geo/query/acc.cgi?acc=GSE149554).

SMART-seq v4 RNA-Sequencing data from single nuclei within the human cortex was previously described (Hodge et al., 2019). Gene expression represented as trimmed average counts per million (average expression of the middle 50% of the data from $\log_2$ (CPM (exons+introns) per gene) was obtained from the Allen Brain Atlas (https://portal.brain-map.org/atlases-and-data/rnaseq).

RNA Extraction and Quantitative RT-PCR

Total RNA extraction and reverse transcription was performed as described. Quantitative PCR reactions using SsoFast EvaGreen Supermix (Bio-Rad) were performed in a CFX384 real time PCR system (Bio-Rad). Relative RNA expression was calculated through the ΔΔCt method and normalised to *Gapdh* expression. Data was analysed in Excel (Microsoft) and statistical analysis performed in GraphPad Prism v7.0c software (GraphPad Software Inc.). Primer sequences are listed in the Table S8.

QUANTIFICATION AND STATISTICAL ANALYSIS

Data is presented as mean ± standard error of the mean (S.E.M) of at least three biological replicates unless otherwise indicated. Statistical significance was estimated using ANOVA followed by Tukey's post hoc test or t-student's test. Significance was defined as $p<0.05$. Statistical details for individual experiments can be found in the figure legends.

Developmental Cell, Volume *55*

Supplemental Information

Functional Diversification of SRSF Protein Kinase to Control Ubiquitin-Dependent Neurodevelopmental Signaling

Francisco Bustos, Anna Segarra-Fas, Gino Nardocci, Andrew Cassidy, Odetta Antico, Lindsay Davidson, Lennart Brandenburg, Thomas J. Macartney, Rachel Toth, C. James Hastie, Jennifer Moran, Robert Gourlay, Joby Varghese, Renata F. Soares, Martin Montecino, and Greg M. Findlay

Figure S1

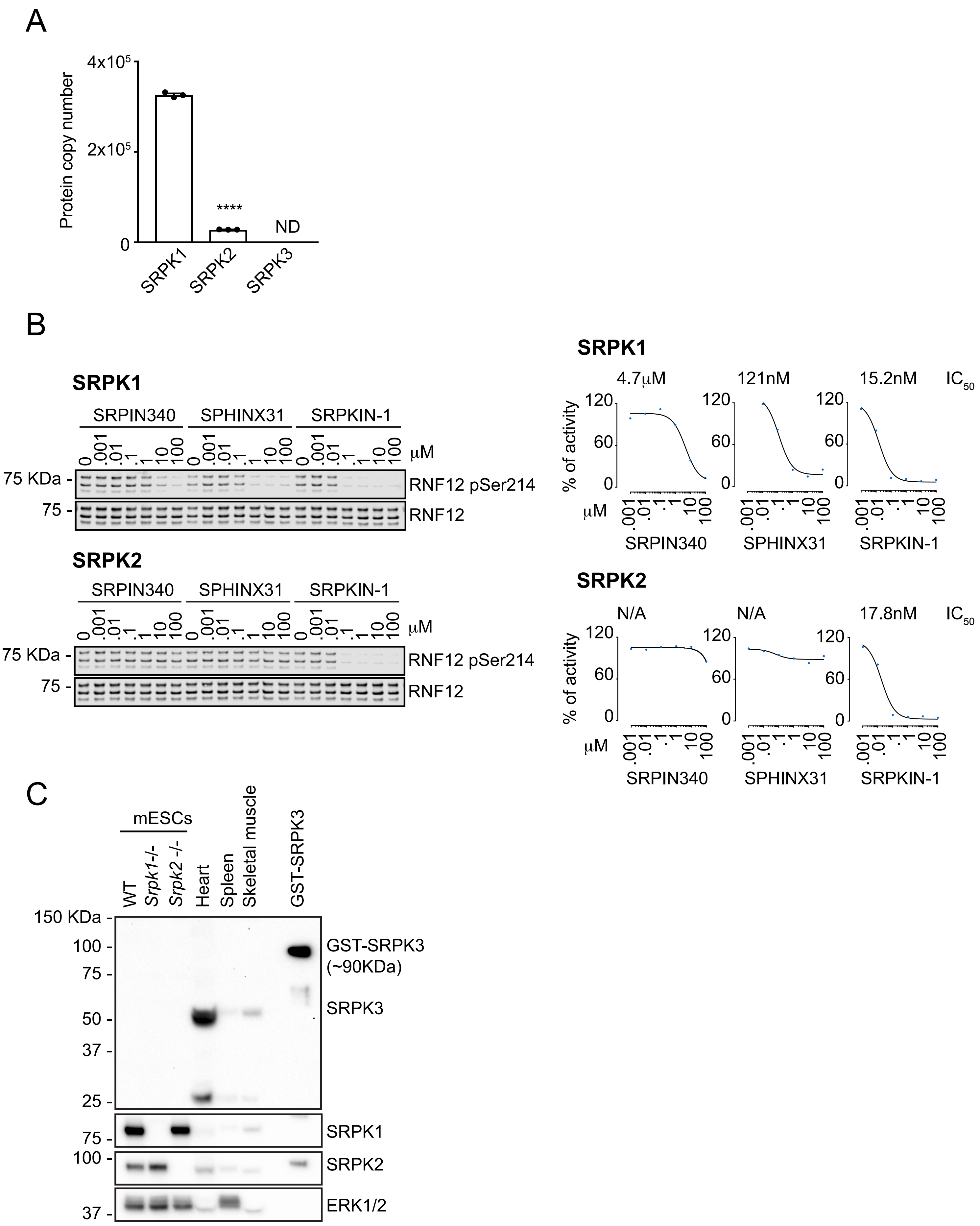
A
Protein copy number
4x10^5
2x10^5
0
****
ND
SRPK1
SRPK2
SRPK3
B
SRPK1
SRPIN340
SPHINX31
SRPKIN-1
0 .001 .01 .1 1 10 100 μM
75 KDa -
RNF12 pSer214
75 -
RNF12
SRPK2
SRPIN340
SPHINX31
SRPKIN-1
0 .001 .01 .1 1 10 100 μM
75 KDa -
RNF12 pSer214
75 -
RNF12
SRPK1
4.7μM
121nM
15.2nM
IC50
% of activity
120
60
0
μM
.001 .01 .1 1 10 100
SRPIN340
SPHINX31
SRPKIN-1
SRPK2
N/A
N/A
17.8nM
IC50
% of activity
120
60
0
μM
.001 .01 .1 1 10 100
SRPIN340
SPHINX31
SRPKIN-1
C
mESCs
WT
Srpk1-/-
Srpk2 -/-
Heart
Spleen
Skeletal muscle
GST-SRPK3
150 KDa -
100 -
75 -
50 -
37 -
25 -
GST-SRPK3
(~90KDa)
SRPK3
75 -
SRPK1
100 -
SRPK2
37 -
ERK1/2

Figure S2

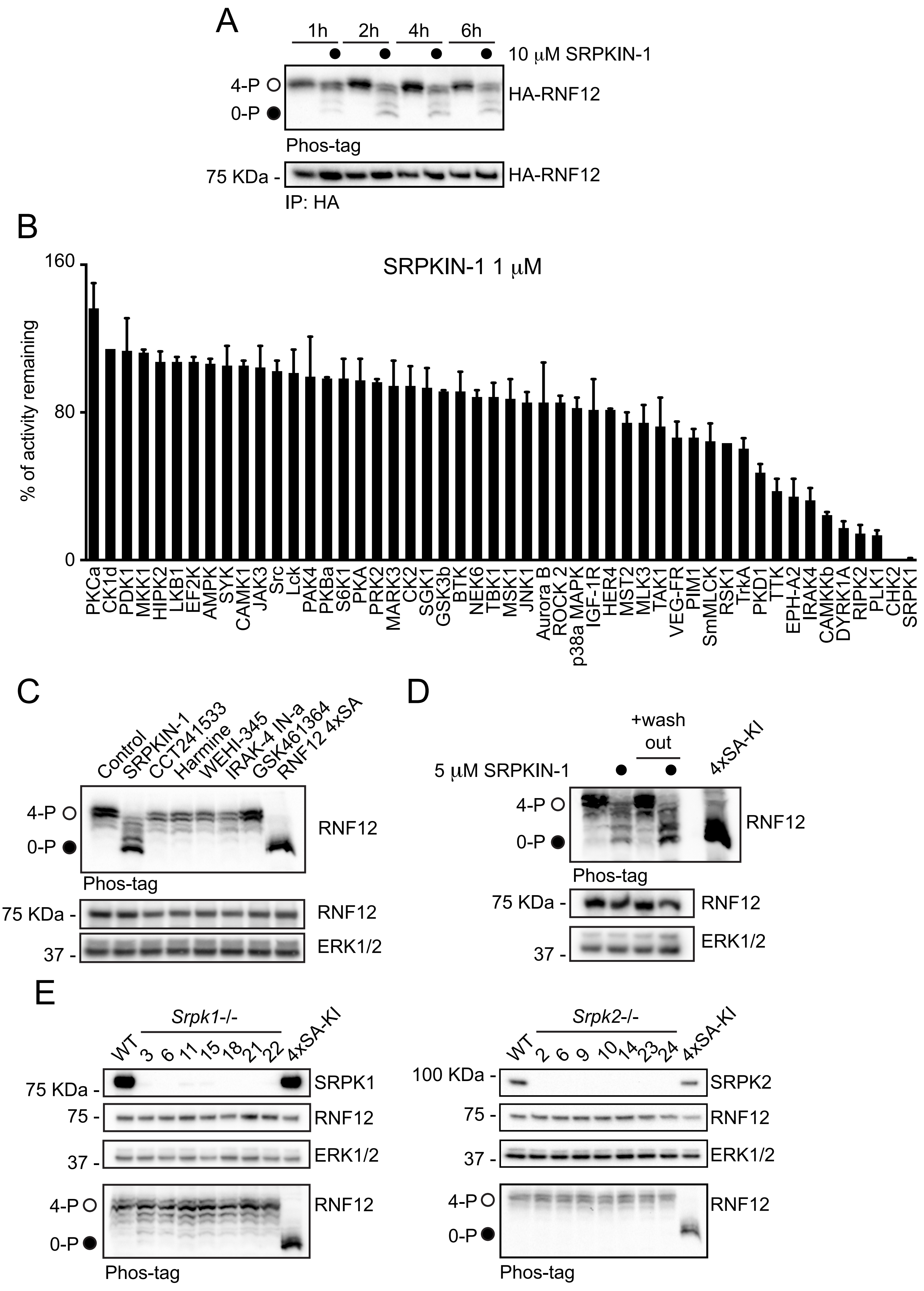
A
1h
2h
4h
6h
10 μM SRPKIN-1
4-P
0-P
HA-RNF12
Phos-tag
75 KDa -
HA-RNF12
IP: HA
B
SRPKIN-1 1 μM
% of activity remaining
160
80
0
PKCa
CK1d
PDK1
MKK1
HIPK2
LKB1
EF2K
AMPK
SYK
CAMK1
JAK3
Src
Lck
PAK4
PKBa
S6K1
PKA
PRK2
MARK3
CK2
SGK1
GSK3b
BTK
NEK6
TBK1
MSK1
JNK1
Aurora B
ROCK 2
p38a MAPK
IGF-1R
HER4
MST2
MLK3
TAK1
VEG-FR
PIM1
SmMLCK
RSK1
TrkA
PKD1
TTK
EPH-A2
IRAK4
CAMKKb
DYRK1A
RIPK2
PLK1
CHK2
SRPK1
C
Control
SRPKIN-1
CCT241533
Harmine
WEHI-345
IRAK-4 IN-a
GSK461364
RNF12 4xSA
4-P
0-P
RNF12
Phos-tag
75 KDa -
RNF12
37 -
ERK1/2
D
+wash out
5 μM SRPKIN-1
4xSA-KI
4-P
0-P
RNF12
Phos-tag
75 KDa -
RNF12
37 -
ERK1/2
E
Srpk1-/-
WT
3
6
11
15
18
21
22
4xSA-KI
75 KDa -
SRPK1
75 -
RNF12
37 -
ERK1/2
4-P
0-P
RNF12
Phos-tag
Srpk2-/-
WT
2
6
9
10
14
23
24
4xSA-KI
100 KDa -
SRPK2
75 -
RNF12
37 -
ERK1/2
4-P
0-P
RNF12
Phos-tag

Figure S3

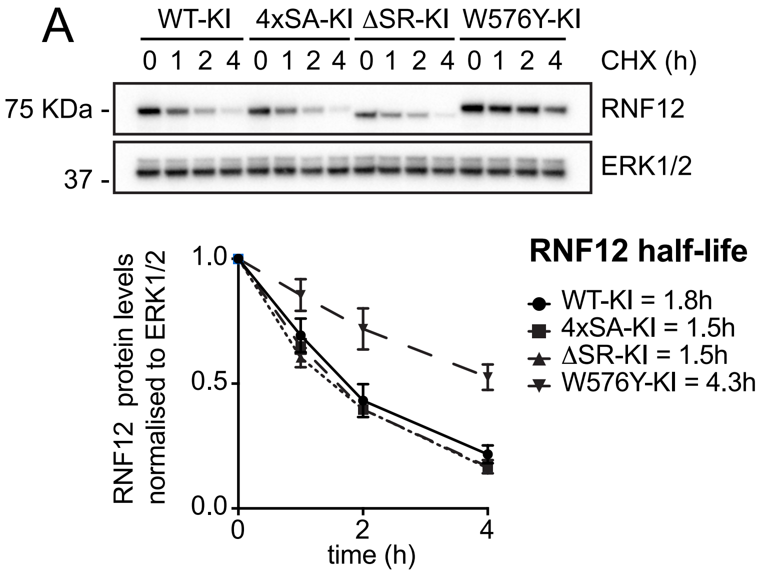

A
WT-KI
4xSA-KI
ΔSR-KI
W576Y-KI
0 1 2 4 0 1 2 4 0 1 2 4 0 1 2 4
CHX (h)
75 KDa -
RNF12
37 -
ERK1/2
RNF12 protein levels normalised to ERK1/2
1.0
0.5
0.0
0
2
4
time (h)
RNF12 half-life
WT-KI = 1.8h
4xSA-KI = 1.5h
ΔSR-KI = 1.5h
W576Y-KI = 4.3h

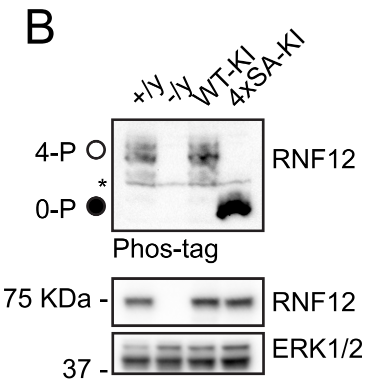

B
+/y
-/y
WT-KI
4xSA-KI
4-P
*
0-P
RNF12
Phos-tag
75 KDa -
RNF12
37 -
ERK1/2

Figure S4

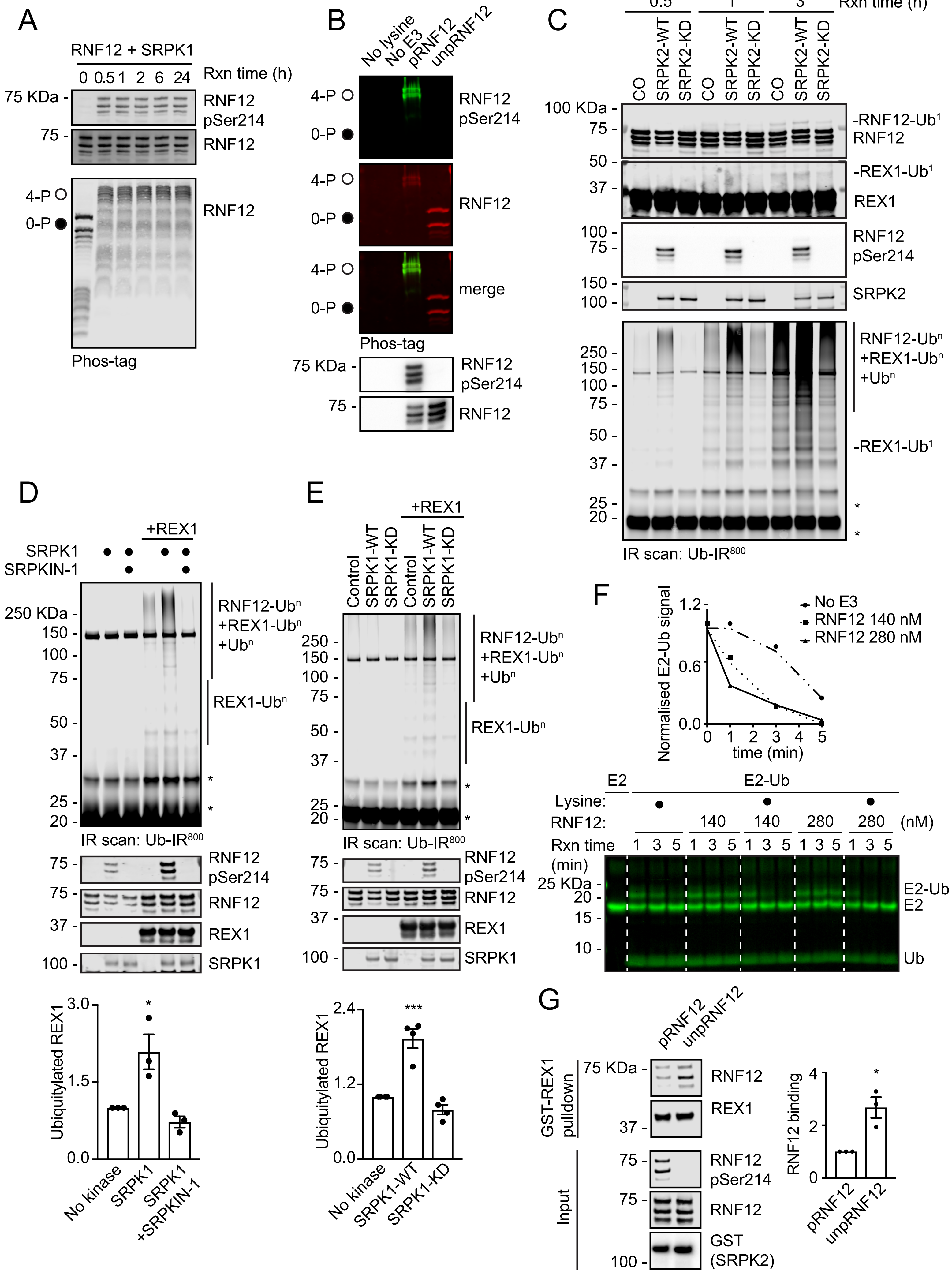
A
RNF12 + SRPK1
Rxn time (h)
0 0.5 1 2 6 24
75 KDa -
RNF12 pSer214
75 -
RNF12
4-P
0-P
RNF12
Phos-tag
B
No lysine
No E3
pRNF12
unpRNF12
RNF12 pSer214
RNF12
merge
Phos-tag
75 KDa -
RNF12 pSer214
75 -
RNF12
C
0.5 1 3 Rxn time (h)
CO SRPK2-WT SRPK2-KD CO SRPK2-WT SRPK2-KD CO SRPK2-WT SRPK2-KD
100 KDa -
-RNF12-Ub¹
RNF12
-REX1-Ub¹
REX1
RNF12 pSer214
SRPK2
RNF12-Ubⁿ +REX1-Ubⁿ +Ubⁿ
-REX1-Ub¹
IR scan: Ub-IR800
D
+REX1
SRPK1
SRPKIN-1
250 KDa -
RNF12-Ubⁿ +REX1-Ubⁿ +Ubⁿ
REX1-Ubⁿ
IR scan: Ub-IR800
RNF12 pSer214
RNF12
REX1
SRPK1
Ubiquitylated REX1
No kinase
SRPK1
SRPK1 +SRPKIN-1
E
+REX1
Control SRPK1-WT SRPK1-KD Control SRPK1-WT SRPK1-KD
RNF12-Ubⁿ +REX1-Ubⁿ +Ubⁿ
REX1-Ubⁿ
IR scan: Ub-IR800
RNF12 pSer214
RNF12
REX1
SRPK1
Ubiquitylated REX1
No kinase
SRPK1-WT
SRPK1-KD
F
Normalised E2-Ub signal
No E3
RNF12 140 nM
RNF12 280 nM
time (min)
E2
E2-Ub
Lysine:
RNF12:
140 140 280 280 (nM)
Rxn time (min)
1 3 5 1 3 5 1 3 5 1 3 5 1 3 5
25 KDa -
E2-Ub
E2
Ub
G
pRNF12
unpRNF12
GST-REX1 pulldown
75 KDa -
RNF12
REX1
Input
RNF12 pSer214
RNF12
GST (SRPK2)
RNF12 binding
pRNF12
unpRNF12

Figure S5

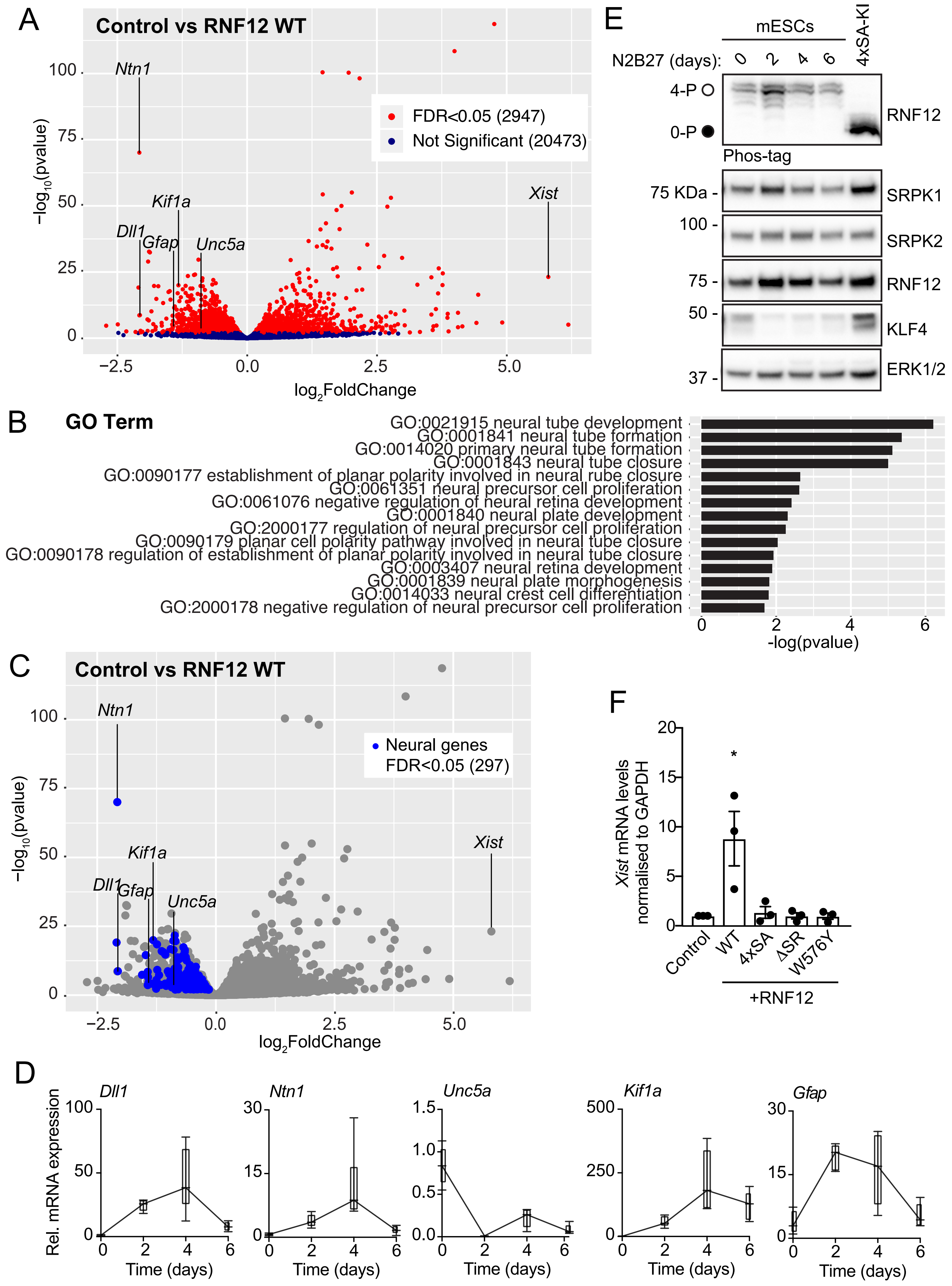
A
Control vs RNF12 WT
FDR<0.05 (2947)
Not Significant (20473)
Ntn1
Kif1a
Dll1
Gfap
Unc5a
Xist
-log10(pvalue)
log2FoldChange
B
GO Term
GO:0021915 neural tube development
GO:0001841 neural tube formation
GO:0014020 primary neural tube formation
GO:0001843 neural tube closure
GO:0090177 establishment of planar polarity involved in neural rube closure
GO:0061351 neural precursor cell proliferation
GO:0061076 negative regulation of neural retina development
GO:0001840 neural plate development
GO:2000177 regulation of neural precursor cell proliferation
GO:0090179 planar cell polarity pathway involved in neural tube closure
GO:0090178 regulation of establishment of planar polarity involved in neural tube closure
GO:0003407 neural retina development
GO:0001839 neural plate morphogenesis
GO:0014033 neural crest cell differentiation
GO:2000178 negative regulation of neural precursor cell proliferation
-log(pvalue)
C
Control vs RNF12 WT
Neural genes FDR<0.05 (297)
D
Rel. mRNA expression
Time (days)
E
mESCs
N2B27 (days): 0 2 4 6
4xSA-KI
4-P
0-P
Phos-tag
RNF12
SRPK1
SRPK2
KLF4
ERK1/2
75 KDa
F
Xist mRNA levels normalised to GAPDH
Control
WT
4xSA
ΔSR
W576Y
+RNF12

Figure S6

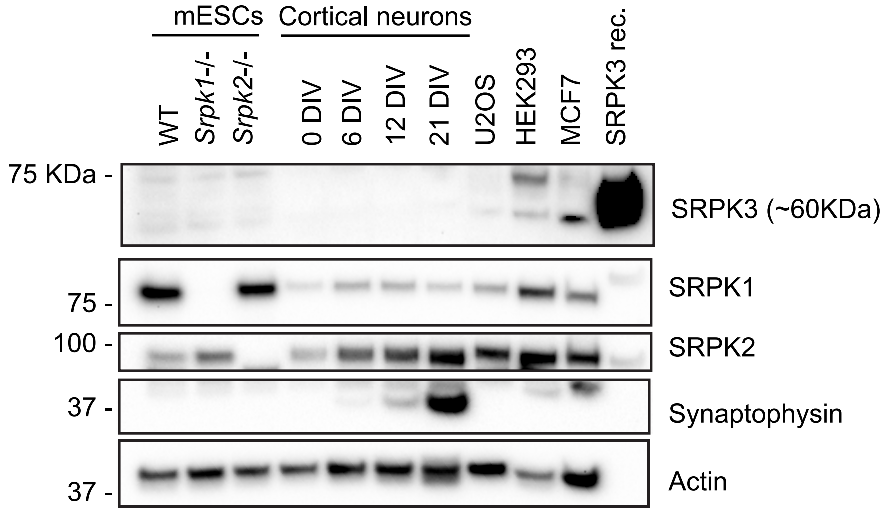
mESCs
Cortical neurons
WT
Srpk1-/-
Srpk2-/-
0 DIV
6 DIV
12 DIV
21 DIV
U2OS
HEK293
MCF7
SRPK3 rec.
75 KDa -
SRPK3 (~60KDa)
75 -
SRPK1
100 -
SRPK2
37 -
Synaptophysin
37 -
Actin

Supplemental Figure Legends

Figure S1. Differential inhibitor sensitivities and expression profiles of SRPK kinases (Related to Figure 1). (A) Wild-type (WT) mESCs were analysed for protein copy number by absolute quantitative proteomics. SRPK1, SRPK2 and SRPK3 protein copy numbers are shown. Data are represented as mean ± S.E.M. (n=3). Unpaired Student's t test, two-sided, confidence level 95%. (****) P<0.0001. ND = not detected. (B) Inhibition of RNF12 phosphorylation *in vitro* by SRPK1 and SRPK2 in the presence of varying concentrations of the indicated SRPK inhibitors was determined by immunoblotting for RNF12 phospho-Ser214 (Left). RNF12 levels are shown as a control. Immunoblots were quantified to generate SRPK inhibitor dose-response curves for inhibition of RNF12 phosphorylation by SRPK1 and SRPK2 *in vitro* (Right). (C) WT, *Srpk1*$^{-/-}$ and *Srpk2*$^{-/-}$ mESCs were cultured and SRPK protein expression was analysed via immunoblotting. Heart, spleen and skeletal muscle tissue lysates and SRPK3 recombinant protein are shown as positive controls for SRPK3 expression.

Figure S2. SRPK1/2 phosphorylate RNF12 SR-motif in mESCs (Related to Figure 2). (A) HA-RNF12 expressing *Rlim*$^{-/y}$ mESCs were treated with 10 µM SRPKIN-1 for the indicated times and SR-motif phosphorylation of HA-immunoprecipitated RNF12 analysed by phos-tag immunoblotting. HA-RNF12 levels are shown as a control. Fully phosphorylated (4-P) and unphosphorylated (0-P) RNF12 SR-motif is indicated by open (○) and closed (●) circles respectively. (B) SRPKIN-1 inhibition of 50 kinases was profiled *in vitro* (MRC-PPU International Centre for Kinase Profiling). Data are represented as mean ± S.D. (n=3). (C) RNF12 expressing mESCs were treated with 10 µM of the following inhibitors: SRPKIN-1 (SRPK inhibitor), CCT-241533 (CHK2 inhibitor), Harmine (DYRK1A inhibitor), WEHI-345 (RIPK2 inhibitor), IRAK-4-Inhibitor-a (IRAK4 inhibitor) and GSK-461364 (PLK1/2 inhibitor) for 4 h, and RNF12 phosphorylation analysed via phos-tag immunoblotting. HA-RNF12 and ERK1/2 levels are shown as a control. (D) RNF12 expressing mESCs were pre-treated with 5 µM SRPKIN-1 for 3 h, media changed and cells cultured for further 5 h (+ wash-out). RNF12 phosphorylation was analysed via phos-tag immunoblotting. ERK1/2 levels are shown as a loading control. (E) Multiple *Srpk1*$^{-/-}$ and *Srpk2*$^{-/-}$ mESC clones were

analysed for RNF12 phosphorylation via phos-tag immunoblotting. SRPK, RNF12 and ERK1/2 levels are shown as controls.

Figure S3. RNF12 protein stability is unaffected by SR-motif phosphorylation (Related to Figure 3). (A) The indicated RNF12 knock-in mESC lines were treated with 350 μM cycloheximide for the indicated times and analysed for RNF12 levels via immunoblotting. ERK1/2 levels are shown as loading control (Top). Quantification of RNF12 signal intensity and determination of protein half-life via immunoblotting and non-linear curve fitting (Bottom). Data are represented as mean ± S.E.M. (n=3) (B) Phos-tag immunoblot analysis of RNF12 SR-motif phosphorylation in the indicated mESC lines. RNF12 and ERK1/2 levels are shown as controls. Fully phosphorylated (4-P) and unphosphorylated (0-P) RNF12 SR-motif is indicated by open (○) and closed (●) circles respectively. (*) Indicates non-specific signal.

Figure S4. SRPK-mediated RNF12 SR-motif phosphorylation stimulates RNF12 E3 ubiquitin ligase activity (Related to Figure 4). (A) Time course of RNF12 phosphorylation by SRPK1 *in vitro,* analysed for SR-motif phosphorylation via RNF12 phospho-Ser214 infrared and phos-tag immunoblotting. Fully phosphorylated (4-P) and unphosphorylated (0-P) RNF12 SR-motif is indicated with open (○) and closed (●) circles respectively. RNF12 levels are shown as a control. (B) RNF12 phosphorylation by SRPK2 for 1 h *in vitro* was analysed via multiplex infrared Phos-tag and regular immunoblotting. This material is representative of samples used in E2 ubiquitin discharge assays displayed in Figure 4D. (C) Recombinant RNF12 was incubated with wild-type (WT) or kinase dead (KD) SRPK2 and subjected to REX1 ubiquitylation assays for the indicated reaction times. RNF12, REX1 RNF12 phospho-Ser214 and SRPK2 expression were determined by immunoblotting. Infrared scans of ubiquitylated substrate signal are shown. Monoubiquitylated RNF12 and REX1 signals are indicated as RNF12-Ub^1 and REX1-Ub^1 respectively. (D) Recombinant RNF12 was incubated with SRPK1 in absence or presence of SRPKIN-1 and subjected to REX1 fluorescent ubiquitylation assays. Infrared scans of ubiquitylated substrate signal, and phospho-Ser214 and total RNF12, REX1 and SRPK1 control infrared immunoblots are shown (Top) with graphical quantification (Bottom). Data are represented as mean ± S.E.M. (n=3).

One-way ANOVA followed by Tukey's multiple comparisons test; confidence level 95%. (*) P=0.0221 (n=3). (E) Recombinant RNF12 was incubated with WT or KD SRPK1 and subjected to REX1 fluorescent ubiquitylation assays. Infrared scans of ubiquitylated substrate signal, and phospho-Ser214 and total RNF12, REX1 and SRPK1 control infrared immunoblots are shown (Top) with graphical quantification (Bottom). Data are represented as mean ± S.E.M. (n=3). One-way ANOVA followed by Tukey's multiple comparisons test; confidence level 95%. (***) P=0.0002 (n=3). (F) The indicated concentration of recombinant RNF12 was assayed for UBE2D1 E2 ubiquitin discharge assay for the indicated reaction times. Normalised E2-ubiquitin conjugate signal quantification (Top) and infrared Coomassie gel staining scans (Bottom) are shown. (G) Recombinant RNF12 was incubated with WT or KD SRPK2 and then subjected to a GST-REX1 pulldown assay. Infrared immunoblots (Left) and RNF12-REX1 binding quantification are shown (Right). Data are represented as mean ± S.E.M. (n=3). Unpaired Student's t test, two-sided, confidence level 95%. (*) P= 0.0133.

Figure S5. RNF12 negatively regulates neurodevelopmental gene expression in mESCs (Related to Figure 5). (A) Volcano plot of RNA-SEQ comparing RNA expression of *Rlim*$^{-/y}$ mESCs transfected with control or WT RNF12. RNAs that are significantly altered by RNF12 are displayed in red (2947 genes). Key neurodevelopmental mRNAs that are inhibited by RNF12 E3 ubiquitin ligase activity are labelled (*Dll1, Ntn1, Gfap, Kif1a, Unc5a*). *Xist* is a known target of RNF12 activity. FDR = False discovery rate. (B) Gene Ontology analysis of RNF12 responsive genes identifies significant enrichment of genes related to neural development (65 genes). (C) Volcano plot of RNA-SEQ comparing RNA expression of *Rlim*$^{-/y}$ mESCs transfected with control or WT RNF12. Neuronal/neural genes negatively regulated by RNF12 identified via Gene Ontology are highlighted in blue (297 genes). (D) Selected neurodevelopmental mRNA expression was analysed by quantitative RT-PCR following mESC neural differentiation in N2B27 media for the indicated times. Data is represented in Box-and-whisker plots showing median, first and third quartiles, and maximum and minimum values (n=6). (E) mESCs were induced to undergo neural differentiation in N2B27 media for the indicated times, and RNF12 SR-motif phosphorylation was analysed by phos-tag immunoblotting. Fully phosphorylated (4-P) and unphosphorylated (0-P)

RNF12 SR-motif is indicated by open (○) and closed (●) circles respectively. SRPK1, SRPK2, RNF12, KLF4 and ERK1/2 levels were analysed by immunoblotting. KLF4 is shown as a pluripotency marker and ERK1/2 as a loading control. (F) mESCs were transfected with the indicated vectors and cultured for 72 h prior analysis of *Xist* RNA expression via quantitative RT-PCR. Data is represented as mean ± S.E.M. (n=3). One-way ANOVA followed by Tukey's multiple comparisons test; confidence level 95%. (*) P=0.0292.

Figure S6. SRPK1/2 are the major isoforms expressed in cultured mouse cortical neurons (Related to Figure 7). Primary cortical neurons isolated from E16.5 C57BL6 mice were cultured for the indicated number of days *in vitro* (DIV) and SRPK1, SRPK2, SRPK3, synaptophysin and actin expression analysed via immunoblotting alongside the indicated mESC lines. U2OS, HEK293, MCF7 and recombinant SRPK3 were used as positive controls for SRPK3 expression, synaptophysin as a neuronal maturation marker and actin as a loading control.

Supplemental Tables

Table S1 (Related to Figure 1). RSRS repeat-containing proteins functionally grouped

mRNA splicing	Other
Rbmx2	Rbbp6
Ccnl1	Ndrg1
Srsf2	Rbm26
Luc7l3	Ppargc1a
Clk2	Paf1
Prpf38a	Arglu1
Arl6ip4	Scaf8
Sfswap	Pdzd7
Rsrc1	Srrm3
Cwc25	Nktr
Rbm39	Pprc1
Srsf12	Snrnp70
Scaf4	Rlim
Srek1	Cherp
Pnn	Lbr
Clasrp	Nkap
Tra2b	Topors
Srsf5	Rsrc2
Thrap3	Rsrp1
Cactin	Sytl5
Srrm1	Erbb3
Scaf1	Bclaf1
Srsf7	Gpatch8
Acin1	Zc3h18
Srsf6	Luc7l
Znf638	Spata18
Prpf38b	Gtpbp4
Ddx46	Tjp2
Srrm2	Luc7l2
Srsf4	
Son	
Srsf1	
U2af2	
U2af1	
Ppig	
Tra2a	
Ccnl2	
Setd2	
Cir1	
Srsf3	
Dhx8	
Rnps1	
Pnisr	
Cdk13	
Snrnp27	
Srsf10	
Zranb2	
Prpf4b	

Table S2 (Related to Figure 1). Functional categorisation of RSRS repeat-containing proteins identified by ScanProsite

Entry	Gene names (primary)	Protein names	Function [CC]
Q8R0F5	Rbmx2	RNA-binding motif protein, X-linked 2	FUNCTION: Involved in pre-mRNA splicing as component of the activated spliceosome.
Q52KE7	Ccnl1	Cyclin-L1 (Cyclin-L) (Cyclin Ania-6a)	FUNCTION: Involved in pre-mRNA splicing. Functions in association with cyclin-dependent kinases (CDKs). May play a role in the regulation of RNA polymerase II (pol II). Inhibited by the CDK-specific inhibitor CDKN1A/p21.
P97868	Rbbp6	E3 ubiquitin-protein ligase RBBP6 (EC 2.3.2.27) (Proliferation potential-related protein) (Protein P2P-R) (RING-type E3 ubiquitin transferase RBBP6) (Retinoblastoma-binding protein 6) (p53-associated cellular protein of testis)	FUNCTION: E3 ubiquitin-protein ligase which promotes ubiquitination of YBX1, leading to its degradation by the proteasome (By similarity). May play a role as a scaffold protein to promote the assembly of the p53/TP53-MDM2 complex, resulting in increase of MDM2-mediated ubiquitination and degradation of p53/TP53; may function as negative regulator of p53/TP53, leading to both apoptosis and cell growth retardation (PubMed:17470788). Regulates DNA-replication and common fragile sites (CFS) stability in a ZBTB38- and MCM10-dependent manner. Controls ZBTB38 protein stability and abundance via ubiquitination and proteasomal degradation, and ZBTB38 in turn negatively regulates the expression of MCM10 which plays an important role in DNA-replication (PubMed:24726359).
Q62433	Ndrg1	Protein NDRG1 (N-myc downstream-regulated gene 1 protein) (Protein Ndr1)	FUNCTION: Stress-responsive protein involved in hormone responses, cell growth, and differentiation. Acts as a tumor suppressor in many cell types. Necessary but not sufficient for p53/TP53-mediated caspase activation and apoptosis. Required for vesicular recycling of CDH1 and TF. May also function in lipid trafficking. Protects cells from spindle disruption damage. Functions in p53/TP53-dependent mitotic spindle checkpoint. Regulates microtubule dynamics and maintains euploidy (By similarity). Has a role in cell trafficking notably of the Schwann cell and is necessary for the maintenance and development of the peripheral nerve myelin sheath.
Q6NZN0	Rbm26	RNA-binding protein 26 (Protein expressed in male leptotene and zygotene spermatocytes 393) (MLZ-393) (RNA-binding motif protein 26)	
O70343	Ppargc1a	Peroxisome proliferator-activated receptor gamma coactivator 1-alpha (PGC-1-alpha) (PPAR-gamma coactivator 1-alpha) (PPARGC-1-alpha)	FUNCTION: Transcriptional coactivator for steroid receptors and nuclear receptors. Greatly increases the transcriptional activity of PPARG and thyroid hormone receptor on the uncoupling protein promoter. Can regulate key mitochondrial genes that contribute to the program of adaptive thermogenesis. Plays an essential role in metabolic reprogramming in response to dietary availability through coordination of the expression of a wide array of genes involved in glucose and fatty acid metabolism. Induces the expression of PERM1 in the skeletal muscle in an ESRRA-dependent manner. Also involved in the integration of the circadian rhythms and energy metabolism. Required for oscillatory expression of clock genes, such as ARNTL/BMAL1 and NR1D1, through the coactivation of RORA and RORC, and metabolic genes, such as PDK4 and PEPCK. Isoform 4 specifically activates the expression of IGF1 and suppresses myostatin expression in skeletal muscle leading to muscle fiber hypertrophy.
Q8K2T8	Paf1	RNA polymerase II-associated factor 1 homolog	FUNCTION: Component of the PAF1 complex (PAF1C) which has multiple functions during transcription by RNA polymerase II and is implicated in regulation of development and maintenance of embryonic stem cell pluripotency. PAF1C associates with RNA polymerase II through interaction with POLR2A CTD non-phosphorylated and 'Ser-2'- and 'Ser-5'-phosphorylated forms and is involved in transcriptional elongation, acting both independently and synergistically with TCEA1 and in cooperation with the DSIF complex and HTATSF1. PAF1C is required for transcription of Hox and Wnt target genes. PAF1C is involved in hematopoiesis and stimulates transcriptional activity of KMT2A/MLL1. PAF1C is involved in histone modifications such as ubiquitination of histone H2B and methylation on histone H3 'Lys-4' (H3K4me3). PAF1C recruits the RNF20/40 E3 ubiquitin-protein ligase complex and the E2 enzyme UBE2A or UBE2B to chromatin which mediate monoubiquitination of 'Lys-120' of histone H2B (H2BK120ub1); UB2A/B-mediated H2B ubiquitination is proposed to be coupled to transcription. PAF1C is involved in mRNA 3' end formation probably through association with cleavage and poly(A) factors. Connects PAF1C with the RNF20/40 E3 ubiquitin-protein ligase complex. Involved in polyadenylation of mRNA precursors (By similarity).
Q62093	Srsf2	Serine/arginine-rich splicing factor 2 (Protein PR264) (Putative myelin regulatory factor 1) (MRF-1) (Splicing component, 35 kDa) (Splicing factor SC35) (SC-35) (Splicing factor, arginine/serine-rich 2)	FUNCTION: Necessary for the splicing of pre-mRNA. It is required for formation of the earliest ATP-dependent splicing complex and interacts with spliceosomal components bound to both the 5'- and 3'-splice sites during spliceosome assembly. It also is required for ATP-dependent interactions of both U1 and U2 snRNPs with pre-mRNA (By similarity). Can bind to the myelin basic protein (MBP) gene MB3 regulatory region and increase transcription of the mbp promoter in cells derived from the CNS. The phosphorylated form (by SRPK2) is required for cellular apoptosis in response to cisplatin treatment (By similarity).
Q5SUF2	Luc7l3	Luc7-like protein 3 (Cisplatin resistance-associated-overexpressed protein)	FUNCTION: Binds cAMP regulatory element DNA sequence. May play a role in RNA splicing (By similarity).
Q3UL36	Arglu1	Arginine and glutamate-rich protein 1	
O35491	Clk2	Dual specificity protein kinase CLK2 (EC 2.7.12.1) (CDC-like kinase 2)	FUNCTION: Dual specificity kinase acting on both serine/threonine and tyrosine-containing substrates. Phosphorylates serine- and arginine-rich (SR) proteins of the spliceosomal complex. May be a constituent of a network of regulatory mechanisms that enable SR proteins to control RNA splicing and can cause redistribution of SR proteins from speckles to a diffuse nucleoplasmic distribution. Acts as a suppressor of hepatic gluconeogenesis and glucose output by repressing PPARGC1A transcriptional activity on gluconeogenic genes via its phosphorylation. Phosphorylates PPP2R5B thereby stimulating the assembly of PP2A phosphatase with the PPP2R5B-AKT1 complex leading to dephosphorylation of AKT1. Phosphorylates: PTPN1, SRSF1 and SRSF3. Regulates the alternative splicing of tissue factor (F3) pre-mRNA in endothelial cells. Phosphorylates PAGE4 at several serine and threonine residues and this phosphorylation attenuates the ability of PAGE4 to potentiate the transcriptional activator activity of JUN (By similarity).
Q6DID3	Scaf8	SR-related and CTD-associated factor 8 (RNA-binding motif protein 16)	FUNCTION: Anti-terminator protein required to prevent early mRNA termination during transcription. Together with SCAF4, acts by suppressing the use of early, alternative poly(A) sites, thereby preventing the accumulation of non-functional truncated proteins. Mechanistically, associates with the phosphorylated C-terminal heptapeptide repeat domain (CTD) of the largest RNA polymerase II subunit (POLR2A), and subsequently binds nascent RNA upstream of early polyadenylation sites to prevent premature mRNA transcript cleavage and polyadenylation. Independently of SCAF4, also acts as a positive regulator of transcript elongation.
E9Q9W7	Pdzd7	PDZ domain-containing protein 7	FUNCTION: In cochlear developing hair cells, essential in organizing the USH2 complex at stereocilia ankle links (PubMed:24334608). Blocks inhibition of adenylate cyclase activity mediated by ADGRV1 (PubMed:24962568).
Q80WV7	Srrm3	Serine/arginine repetitive matrix protein 3	FUNCTION: May play a role in regulating breast cancer cell invasiveness. May be involved in RYBP-mediated breast cancer progression.
P30415	Nktr	NK-tumor recognition protein (NK-TR protein) (Natural-killer cells cyclophilin-related protein) (Peptidyl-prolyl cis-trans isomerase NKTR) (PPIase) (EC 5.2.1.8)	FUNCTION: PPIase that catalyzes the cis-trans isomerization of proline imidic peptide bonds in oligopeptides and may therefore assist protein folding. Component of a putative tumor-recognition complex involved in the function of NK cells.
Q4FK66	Prpf38a	Pre-mRNA-splicing factor 38A	FUNCTION: Involved in pre-mRNA splicing as a component of the spliceosome.
Q9JM93	Arl6ip4	ADP-ribosylation factor-like protein 6-interacting protein 4 (ARL-6-interacting protein 4) (Aip-4) (Splicing factor SRrp37)	FUNCTION: Involved in modulating alternative pre-mRNA splicing with either 5' distal site activation or preferential use of 3' proximal site.
Q3USH5	Sfswap	Splicing factor, suppressor of white-apricot homolog (Splicing factor, arginine/serine-rich 8) (Suppressor of white apricot protein homolog)	FUNCTION: Plays a role as an alternative splicing regulator. Regulates its own expression at the level of RNA processing. Also regulates the splicing of fibronectin and CD45 genes. May act, at least in part, by interaction with other R/S-containing splicing factors. Represses the splicing of MAPT/Tau exon 10 (By similarity).
Q6NZN1	Pprc1	Peroxisome proliferator-activated receptor gamma coactivator-related protein 1 (PGC-1-related coactivator) (PRC)	FUNCTION: Acts as a coactivator during transcriptional activation of nuclear genes related to mitochondrial biogenesis and cell growth. Involved in the transcription coactivation of CREB and NRF1 target genes (By similarity).
Q62376	Snrnp70	U1 small nuclear ribonucleoprotein 70 kDa (U1 snRNP 70 kDa) (U1-70K) (snRNP70)	FUNCTION: Component of the spliceosomal U1 snRNP, which is essential for recognition of the pre-mRNA 5' splice-site and the subsequent assembly of the spliceosome. SNRNP70 binds to the loop I region of U1-snRNA.; FUNCTION: [Isoform 2]: Truncated isoforms that lack the RRM domain cannot bind U1-snRNA.
Q9DBU6	Rsrc1	Serine/Arginine-related protein 53 (SRrp53) (Arginine/serine-rich coiled-coil protein 1)	FUNCTION: Plays a role in pre-mRNA splicing. Involved in both constitutive and alternative pre-mRNA splicing. May have a role in the recognition of the 3' splice site during the second step of splicing (By similarity).
Q9DBF7	Cwc25	Pre-mRNA-splicing factor CWC25 homolog (Coiled-coil domain-containing protein 49) (Spliceosome-associated protein homolog CWC25)	FUNCTION: Involved in pre-mRNA splicing as component of the spliceosome.
Q8VH51	Rbm39	RNA-binding protein 39 (Coactivator of activating protein 1 and estrogen receptors) (Coactivator of AP-1 and ERs) (RNA-binding motif protein 39) (RNA-binding region-containing protein 2) (Transcription coactivator CAPER)	FUNCTION: Transcriptional coactivator for steroid nuclear receptors ESR1/ER-alpha and ESR2/ER-beta, and JUN/AP-1. May be involved in pre-mRNA splicing process.
Q8C8K3	Srsf12	Serine/arginine-rich splicing factor 12 (Splicing factor, arginine/serine-rich 13B)	FUNCTION: Splicing factor that seems to antagonize SR proteins in pre-mRNA splicing regulation.
Q7TSH6	Scaf4	SR-related and CTD-associated factor 4 (CTD-binding SR-like protein RA4) (Splicing factor, arginine/serine-rich 15)	FUNCTION: Anti-terminator protein required to prevent early mRNA termination during transcription. Together with SCAF8, acts by suppressing the use of early, alternative poly(A) sites, thereby preventing the accumulation of non-functional truncated proteins. Mechanistically, associates with the phosphorylated C-terminal heptapeptide repeat domain (CTD) of the largest RNA polymerase II subunit (POLR2A), and subsequently binds nascent RNA upstream of early polyadenylation sites to prevent premature mRNA transcript cleavage and polyadenylation. Independently of SCAF8, also acts as a suppressor of transcriptional readthrough.
Q9WTV7	Rlim	E3 ubiquitin-protein ligase RLIM (EC 2.3.2.27) (LIM domain-interacting RING finger protein) (RING finger LIM domain-binding protein) (R-LIM) (RING finger protein 12) (RING-type E3 ubiquitin transferase RLIM)	FUNCTION: E3 ubiquitin-protein ligase that acts as a negative coregulator for LIM homeodomain transcription factors by mediating the ubiquitination and subsequent degradation of LIM cofactors LDB1 and LDB2 and by mediating the recruitment the SIN3a/histone deacetylase corepressor complex. Ubiquitination and degradation of LIM cofactors LDB1 and LDB2 allows DNA-bound LIM homeodomain transcription factors to interact with other protein partners such as RLIM. Plays a role in telomere length-mediated growth suppression by mediating the ubiquitination and degradation of TERF1. By targeting ZFP42 for degradation, acts as an activator of random inactivation of X chromosome in the embryo, a stochastic process in which one X chromosome is inactivated to minimize sex-related dosage differences of X-encoded genes in somatic cells of female placental mammals.
Q8BZX4	Srek1	Splicing regulatory glutamine/lysine-rich protein 1 (Serine/arginine-rich-splicing regulatory protein 86) (SRrp86) (Splicing factor, arginine/serine-rich 12)	FUNCTION: Participates in the regulation of alternative splicing by modulating the activity of other splice facors. Inhibits the splicing activity of SFRS1, SFRS2 and SFRS6. Augments the splicing activity of SFRS3 (By similarity).
O35691	Pnn	Pinin	FUNCTION: Transcriptional activator binding to the E-box 1 core sequence of the E-cadherin promoter gene; the core-binding sequence is 5'CAGGTG-3'. Capable of reversing CTBP1-mediated transcription repression. Auxiliary component of the splicing-dependent multiprotein exon junction complex (EJC) deposited at splice junction on mRNAs. The EJC is a dynamic structure consisting of core proteins and several peripheral nuclear and cytoplasmic associated factors that join the complex only transiently either during EJC assembly or during subsequent mRNA metabolism. Participates in the regulation of alternative pre-mRNA splicing. Associates to spliced mRNA within 60 nt upstream of the 5'-splice sites. Component of the PSAP complex which binds RNA in a sequence-independent manner and is proposed to be recruited to the EJC prior to or during the splicing process and to regulate specific excision of introns in specific transcription subsets. Involved in the establishment and maintenance of epithelia cell-cell adhesion (By similarity).
Q8CFC7	Clasrp	CLK4-associating serine/arginine rich protein (Clk4-associating SR-related protein) (Serine/arginine-rich splicing factor 16) (Splicing factor, arginine/serine-rich 16) (Suppressor of white-apricot homolog 2)	FUNCTION: Probably functions as an alternative splicing regulator. May regulate the mRNA splicing of genes such as CLK1. May act by regulating members of the CLK kinase family.
Q8CGZ0	Cherp	Calcium homeostasis endoplasmic reticulum protein (SR-related CTD-associated factor 6)	FUNCTION: Involved in calcium homeostasis, growth and proliferation.

Q3U9G9	Lbr	Delta(14)-sterol reductase LBR (Delta-14-SR) (EC 1.3.1.70) (3-beta-hydroxysterol Delta (14)-reductase) (C-14 sterol reductase) (C14SR) (Integral nuclear envelope inner membrane protein) (Lamin-B receptor) (Sterol C14-reductase)	FUNCTION: Catalyzes the reduction of the C14-unsaturated bond of lanosterol, as part of the metabolic pathway leading to cholesterol biosynthesis (PubMed:18785926). Plays a critical role in myeloid cell cholesterol biosynthesis which is essential to both myeloid cell growth and functional maturation (PubMed:22140257). Mediates the activation of NADPH oxidases, perhaps by maintaining critical levels of cholesterol required for membrane lipid raft formation during neutrophil differentiation (PubMed:22140257). Anchors the lamina and the heterochromatin to the inner nuclear membrane (By similarity).
P62996	Tra2b	Transformer-2 protein homolog beta (TRA-2 beta) (TRA2-beta) (Silica-induced gene 41 protein) (SIG-41) (Splicing factor, arginine/serine-rich 10) (Transformer-2 protein homolog B)	FUNCTION: Sequence-specific RNA-binding protein which participates in the control of pre-mRNA splicing. Can either activate or suppress exon inclusion. Acts additively with RBMX to promote exon 7 inclusion of the survival motor neuron SMN2. Activates the splicing of MAPT/Tau exon 10. Alters pre-mRNA splicing patterns by antagonizing the effects of splicing regulators, like RBMX. Binds to the AG-rich SE2 domain in the SMN exon 7 RNA. Binds to pre-mRNA (By similarity).
O35326	Srsf5	Serine/arginine-rich splicing factor 5 (Delayed-early protein HRS) (Pre-mRNA-splicing factor SRP40) (Splicing factor, arginine/serine-rich 5)	FUNCTION: May be required for progression through G1 and entry into S phase of cell growth. May play a regulatory role in pre-mRNA splicing. Autoregulates its own expression. Plays a role in constitutive splicing and can modulate the selection of alternative splice sites (By similarity).
Q569Z6	Thrap3	Thyroid hormone receptor-associated protein 3 (Thyroid hormone receptor-associated protein complex 150 kDa component) (Trap150)	FUNCTION: Involved in pre-mRNA splicing. Remains associated with spliced mRNA after splicing which probably involves interactions with the exon junction complex (EJC). Can trigger mRNA decay which seems to be independent of nonsense-mediated decay involving premature stop codons (PTC) recognition. May be involved in nuclear mRNA decay. Involved in regulation of signal-induced alternative splicing. During splicing of PTPRC/CD45 is proposed to sequester phosphorylated SFPQ from PTPRC/CD45 pre-mRNA in resting T-cells. Involved in cyclin-D1/CCND1 mRNA stability probably by acting as component of the SNARP complex which associates with both the 3'end of the CCND1 gene and its mRNA. Involved in response to DNA damage. Is excluded from DNA damage sites in a manner that parallels transcription inhibition; the function may involve the SNARP complex. Initially thought to play a role in transcriptional coactivation through its association with the TRAP complex; however, it is not regarded as a stable Mediator complex subunit. Cooperatively with HELZ2, enhances the transcriptional activation mediated by PPARG, maybe through the stabilization of the PPARG binding to DNA in presence of ligand. May play a role in the terminal stage of adipocyte differentiation. Plays a role in the positive regulation of the circadian clock. Acts as a coactivator of the CLOCK-ARNTL/BMAL1 heterodimer and promotes its transcriptional activator activity and binding to circadian target genes (PubMed:24043798).
Q9D0F4	Nkap	NF-kappa-B-activating protein	FUNCTION: Acts as a transcriptional repressor. Plays a role as a transcriptional corepressor of the Notch-mediated signaling required for T-cell development. Also involved in the TNF and IL-1 induced NF-kappa-B activation. Associates with chromatin at the Notch-regulated SKP2 promoter (By similarity).
Q80Z37	Topors	E3 ubiquitin-protein ligase Topors (EC 2.3.2.27) (RING-type E3 ubiquitin transferase Topors) (SUMO1-protein E3 ligase Topors) (Topoisomerase I-binding RING finger protein) (Topoisomerase I-binding arginine/serine-rich protein) (Tumor suppressor p53-binding protein 3) (p53-binding protein 3) (p53BP3)	FUNCTION: Functions as an E3 ubiquitin-protein ligase and as a E3 SUMO1-protein ligase. Probable tumor suppressor involved in cell growth, cell proliferation and apoptosis that regulates p53/TP53 stability through ubiquitin-dependent degradation. May regulate chromatin modification through sumoylation of several chromatin modification-associated proteins. May be involved in DNA-damage-induced cell death through IKBKE sumoylation.
A2RTL5	Rsrc2	Arginine/serine-rich coiled-coil protein 2	
Q3UC65	Rsrp1	Arginine/serine-rich protein 1	
Q80T23	Sytl5	Synaptotagmin-like protein 5	FUNCTION: May act as Rab effector protein and play a role in vesicle trafficking. Binds phospholipids (By similarity).
Q61526	Erbb3	Receptor tyrosine-protein kinase erbB-3 (EC 2.7.10.1) (Glial growth factor receptor) (Proto-oncogene-like protein c-ErbB-3)	FUNCTION: Tyrosine-protein kinase that plays an essential role as cell surface receptor for neuregulins. Binds to neuregulin-1 (NRG1) and is activated by it; ligand-binding increases phosphorylation on tyrosine residues and promotes its association with the p85 subunit of phosphatidylinositol 3-kinase. May also be activated by CSPG5. Involved in the regulation of myeloid cell differentiation.
Q9CS00	Cactin	Cactin	FUNCTION: Involved in the regulation of innate immune response. Acts as negative regulator of Toll-like receptor and interferon-regulatory factor (IRF) signaling pathways. Contributes to the regulation of transcriptional activation of NF-kappa-B target genes in response to endogenous proinflammatory stimuli. May play a role during early embryonic development. Probably involved in pre-mRNA splicing (By similarity).
Q52KI8	Srrm1	Serine/arginine repetitive matrix protein 1 (Plenty-of-prolines 101)	FUNCTION: Part of pre- and post-splicing multiprotein mRNP complexes. Involved in numerous pre-mRNA processing events. Promotes constitutive and exonic splicing enhancer (ESE)-dependent splicing activation by bridging together sequence-specific (SR family proteins, SFRS4, SFRS5 and TRA2B/SFRS10) and basal snRNP (SNRP70 and SNRPA1) factors of the spliceosome. Stimulates mRNA 3'-end cleavage independently of the formation of an exon junction complex. Binds both pre-mRNA and spliced mRNA 20-25 nt upstream of exon-exon junctions. Binds RNA and DNA with low sequence specificity and has similar preference for either double- or single-stranded nucleic acid substrates.
Q8K019	Bclaf1	Bcl-2-associated transcription factor 1 (Btf)	FUNCTION: Death-promoting transcriptional repressor. May be involved in cyclin-D1/CCND1 mRNA stability through the SNARP complex which associates with both the 3'end of the CCND1 gene and its mRNA (By similarity).
Q5U4C3	Scaf1	Splicing factor, arginine/serine-rich 19 (SR-related and CTD-associated factor 1)	FUNCTION: May function in pre-mRNA splicing.
Q8BL97	Srsf7	Serine/arginine-rich splicing factor 7 (Splicing factor, arginine/serine-rich 7)	FUNCTION: Required for pre-mRNA splicing. Represses the splicing of MAPT/Tau exon 10. May function as export adapter involved in mRNA nuclear export such as of histone H2A. Binds mRNA which is thought to be transferred to the NXF1-NXT1 heterodimer for export (TAP/NXF1 pathway); enhances NXF1-NXT1 RNA-binding activity. RNA-binding is semi-sequence specific (By similarity).
A2A6A1	Gpatch8	G patch domain-containing protein 8	
Q0P678	Zc3h18	Zinc finger CCCH domain-containing protein 18 (Nuclear protein NHN1)	
Q9JIX8	Acin1	Apoptotic chromatin condensation inducer in the nucleus (Acinus)	FUNCTION: Auxiliary component of the splicing-dependent multiprotein exon junction complex (EJC) deposited at splice junction on mRNAs. The EJC is a dynamic structure consisting of core proteins and several peripheral nuclear and cytoplasmic associated factors that join the complex only transiently either during EJC assembly or during subsequent mRNA metabolism. Component of the ASAP complexes which bind RNA in a sequence-independent manner and are proposed to be recruited to the EJC prior to or during the splicing process and to regulate specific excision of introns in specific transcription subsets; ACIN1 confers RNA-binding to the complex. The ASAP complex can inhibit RNA processing during in vitro splicing reactions. The ASAP complex promotes apoptosis and is disassembled after induction of apoptosis. Involved in the splicing modulation of BCL2L1/Bcl-X (and probably other apoptotic genes); specifically inhibits formation of proapoptotic isoforms such as Bcl-X(S); the activity is different from the established EJC assembly and function. Induces apoptotic chromatin condensation after activation by CASP3. Regulates cyclin A1, but not cyclin A2, expression in leukemia cells (By similarity).
Q3TWW8	Srsf6	Serine/arginine-rich splicing factor 6 (Pre-mRNA-splicing factor SRP55) (Splicing factor, arginine/serine-rich 6)	FUNCTION: Plays a role in constitutive splicing and modulates the selection of alternative splice sites. Plays a role in the alternative splicing of MAPT/Tau exon 10. Binds to alternative exons of TNC pre-mRNA and promotes the expression of alternatively spliced TNC. Plays a role in wound healing and in the regulation of keratinocyte differentiation and proliferation via its role in alternative splicing (By similarity).
Q61464	Znf638	Zinc finger protein 638 (Nuclear protein 220) (Zinc finger matrin-like protein)	FUNCTION: Transcription factor that binds to cytidine clusters in double-stranded DNA (By similarity). Plays a key role in the silencing of unintegrated retroviral DNA: some part of the retroviral DNA formed immediately after infection remains unintegrated in the host genome and is transcriptionally repressed (PubMed:30487602). Mediates transcriptional repression of unintegrated viral DNA by specifically binding to the cytidine clusters of retroviral DNA and mediating the recruitment of chromatin silencers, such as the HUSH complex, SETDB1 and the histone deacetylases HDAC1 and HDAC4 (PubMed:30487602). Acts as an early regulator of adipogenesis by acting as a transcription cofactor of CEBPs (CEBPA, CEBPD and/or CEBPG), controlling the expression of PPARG and probably of other proadipogenic genes, such as SREBF1 (PubMed:21602272). May also regulate alternative splicing of target genes during adipogenesis (PubMed:25024404).
Q80SY5	Prpf38b	Pre-mRNA-splicing factor 38B	FUNCTION: May be required for pre-mRNA splicing.
Q569Z5	Ddx46	Probable ATP-dependent RNA helicase DDX46 (EC 3.6.4.13) (DEAD box protein 46)	FUNCTION: Plays an essential role in splicing, either prior to, or during A complex formation.
Q8BTI8	Srrm2	Serine/arginine repetitive matrix protein 2	FUNCTION: Required for pre-mRNA splicing as component of the spliceosome.
Q8VE97	Srsf4	Serine/arginine-rich splicing factor 4 (Splicing factor, arginine/serine-rich 4)	FUNCTION: Plays a role in alternative splice site selection during pre-mRNA splicing. Represses the splicing of MAPT/Tau exon 10 (By similarity).
Q9QX47	Son	Protein SON (Negative regulatory element-binding protein) (NRE-binding protein)	FUNCTION: RNA-binding protein that acts as a mRNA splicing cofactor by promoting efficient splicing of transcripts that possess weak splice sites. Specifically promotes splicing of many cell-cycle and DNA-repair transcripts that possess weak splice sites, such as TUBG1, KATNB1, TUBGCP2, AURKB, PCNT, AKT1, RAD23A, and FANCG. Probably acts by facilitating the interaction between Serine/arginine-rich proteins such as SRSF2 and the RNA polymerase II. Also binds to DNA; binds to the consensus DNA sequence: 5'-GA[GT]AN[CG][AG]CC-3' (By similarity). Essential for correct RNA splicing of multiple genes critical for brain development, neuronal migration and metabolism, including TUBG1, FLNA, PNKP, WDR62, PSMD3, PCK2, PFKL, IDH2, and ACY1 (By similarity). May also regulate the ghrelin signaling in hypothalamic neuron by acting as a negative regulator of GHSR expression (PubMed:20876580).
Q6PDM2	Srsf1	Serine/arginine-rich splicing factor 1 (ASF/SF2) (Pre-mRNA-splicing factor SRp30a) (Splicing factor, arginine/serine-rich 1)	FUNCTION: Plays a role in preventing exon skipping, ensuring the accuracy of splicing and regulating alternative splicing. Interacts with other spliceosomal components, via the RS domains, to form a bridge between the 5'- and 3'-splice site binding components, U1 snRNP and U2AF. Can stimulate binding of U1 snRNP to a 5'-splice site-containing pre-mRNA. Binds to purine-rich RNA sequences, either the octamer, 5'-RGAAGAAC-3' (r=A or G) or the decamers, AGGACAGAGC/AGGACGAAGC. Binds preferentially to the 5'-CGAGGCG-3' motif in vitro. Three copies of the octamer constitute a powerful splicing enhancer in vitro, the ASF/SF2 splicing enhancer (ASE) which can specifically activate ASE-dependent splicing (By similarity). Specifically regulates alternative splicing of cardiac isoforms of CAMK2D, LDB3/CYPHER and TNNT2/CTNT during heart remodeling at the juvenile to adult transition. The inappropriate accumulation of a neonatal and neuronal isoform of CAMKD2 in the adult heart results in aberrant calcium handling and defective excitation-contraction coupling in cardiomyocytes. May function as export adapter involved in mRNA nuclear export through the TAP/NXF1 pathway (PubMed:15652482).
P26369	U2af2	Splicing factor U2AF 65 kDa subunit (U2 auxiliary factor 65 kDa subunit) (U2 snRNP auxiliary factor large subunit)	FUNCTION: Plays a role in pre-mRNA splicing and 3'-end processing. By recruiting PRPF19 and the PRP19C/Prp19 complex/NTC/Nineteen complex to the RNA polymerase II C-terminal domain (CTD), and thereby pre-mRNA, may couple transcription to splicing. Required for the export of mRNA out of the nucleus, even if the mRNA is encoded by an intron-less gene. Positively regulates pre-mRNA 3'-end processing by recruiting the CFIm complex to cleavage and polyadenylation signals.
Q9D883	U2af1	Splicing factor U2AF 35 kDa subunit (U2 auxiliary factor 35 kDa subunit) (U2 snRNP auxiliary factor small subunit)	FUNCTION: Plays a critical role in both constitutive and enhancer-dependent splicing by mediating protein-protein interactions and protein-RNA interactions required for accurate 3'-splice site selection. Recruits U2 snRNP to the branch point. Directly mediates interactions between U2AF2 and proteins bound to the enhancers and thus may function as a bridge between U2AF2 and the enhancer complex to recruit it to the adjacent intron (By similarity).
A2AR02	Ppig	Peptidyl-prolyl cis-trans isomerase G (PPIase G) (Peptidyl-prolyl isomerase G) (EC 5.2.1.8) (Cyclophilin G) (Rotamase G)	FUNCTION: PPIase that catalyzes the cis-trans isomerization of proline imidic peptide bonds in oligopeptides and may therefore assist protein folding. May be implicated in the folding, transport, and assembly of proteins. May play an important role in the regulation of pre-mRNA splicing.
Q6PFR5	Tra2a	Transformer-2 protein homolog alpha (TRA-2 alpha) (TRA2-alpha) (Transformer-2 protein homolog A)	FUNCTION: Sequence-specific RNA-binding protein which participates in the control of pre-mRNA splicing.
Q9JJA7	Ccnl2	Cyclin-L2 (Cyclin Ania-6b) (Paneth cell-enhanced expression protein) (PCEE)	FUNCTION: Involved in pre-mRNA splicing. May induce cell death, possibly by acting on the transcription and RNA processing of apoptosis-related factors.
E9Q5F9	Setd2	Histone-lysine N-methyltransferase SETD2 (EC 2.1.1.-) (Lysine N-methyltransferase 3A) (Protein-lysine N-methyltransferase SETD2) (EC 2.1.1.-) (SET domain-containing protein 2)	FUNCTION: Histone methyltransferase that specifically trimethylates 'Lys-36' of histone H3 (H3K36me3) using dimethylated 'Lys-36' (H3K36me2) as substrate (PubMed:18157086, PubMed:20133625). Represents the main enzyme generating H3K36me3, a specific tag for epigenetic transcriptional activation (PubMed:18157086, PubMed:20133625). Plays a role in chromatin structure modulation during elongation by coordinating recruitment of the FACT complex and by interacting with hyperphosphorylated POLR2A (By similarity). Acts as a key regulator of DNA mismatch repair in G1 and early S phase by generating H3K36me3, a mark required to recruit MSH6 subunit of the MutS alpha complex: early recruitment of the MutS alpha complex to chromatin to be replicated allows a quick identification of mismatch DNA to initiate the mismatch repair reaction (By similarity). Required for DNA double-strand break repair in response to DNA damage: acts by mediating formation of H3K36me3, promoting recruitment of RAD51 and DNA repair via homologous recombination (HR) (By similarity). Acts as a tumor suppressor (By similarity). H3K36me3 also plays an essential role in the maintenance of a heterochromatic state, by recruiting DNA methyltransferase DNMT3A (By similarity). H3K36me3 is also enhanced in intron-containing genes, suggesting that SETD2 recruitment is enhanced by splicing and that splicing is coupled to recruitment of elongating RNA polymerase (By similarity). Required during angiogenesis (PubMed:20133625). Required for endoderm development by promoting embryonic stem cell differentiation toward endoderm: acts by mediating formation of H3K36me3 in distal promoter regions of FGFR3, leading to regulate transcription initiation of FGFR3

			(PubMed:25242323). In addition to histones, also mediates methylation of other proteins, such as tubulins and STAT1 (PubMed:27518565). Trimethylates 'Lys-40' of alpha-tubulins such as TUBA1B (alpha-TubK40me3); alpha-TubK40me3 is required for normal mitosis and cytokinesis and may be a specific tag in cytoskeletal remodeling (PubMed:27518565). Involved in interferon-alpha-induced antiviral defense by mediating both monomethylation of STAT1 at 'Lys-525' and catalyzing H3K36me3 on promoters of some interferon-stimulated genes (ISGs) to activate gene transcription (By similarity).
Q9CYI4	Luc7l	Putative RNA-binding protein Luc7-like 1	FUNCTION: May bind to RNA via its Arg/Ser-rich domain.
Q0P557	Spata18	Mitochondria-eating protein (Spermatogenesis-associated protein 18)	FUNCTION: Key regulator of mitochondrial quality that mediates the repairing or degradation of unhealthy mitochondria in response to mitochondrial damage. Mediator of mitochondrial protein catabolic process (also named MALM) by mediating the degradation of damaged proteins inside mitochondria by promoting the accumulation in the mitochondrial matrix of hydrolases that are characteristic of the lysosomal lumen. Also involved in mitochondrion degradation of damaged mitochondria by promoting the formation of vacuole-like structures (named MIV), which engulf and degrade unhealthy mitochondria by accumulating lysosomes. May have a role in spermatogenesis, especially in cell differentiation from late elongate spermatids to mature spermatozoa (By similarity). The physical interaction of SPATA18/MIEAP, BNIP3 and BNIP3L/NIX at the mitochondrial outer membrane regulates the opening of a pore in the mitochondrial double membrane in order to mediate the translocation of lysosomal proteins from the cytoplasm to the mitochondrial matrix (By similarity).
Q9DA19	Cir1	Corepressor interacting with RBPJ 1 (CBF1-interacting corepressor)	FUNCTION: Regulates transcription and acts as corepressor for RBPJ. Recruits RBPJ to the Sin3-histone deacetylase complex (HDAC). Required for RBPJ-mediated repression of transcription (By similarity). May modulate splice site selection during alternative splicing of pre-mRNAs.
P84104	Srsf3	Serine/arginine-rich splicing factor 3 (Pre-mRNA-splicing factor SRP20) (Protein X16) (Splicing factor, arginine/serine-rich 3)	FUNCTION: Splicing factor that specifically promotes exon-inclusion during alternative splicing. Interaction with YTHDC1, a RNA-binding protein that recognizes and binds N6-methyladenosine (m6A)-containing RNAs, promotes recruitment of SRSF3 to its mRNA-binding elements adjacent to m6A sites, leading to exon-inclusion during alternative splicing. Also functions as export adapter involved in mRNA nuclear export. Binds mRNA which is thought to be transferred to the NXF1-NXT1 heterodimer for export (TAP/NXF1 pathway); enhances NXF1-NXT1 RNA-binding activity. Involved in nuclear export of m6A-containing mRNAs via interaction with YTHDC1: interaction with YTHDC1 facilitates m6A-containing mRNA-binding to both SRSF3 and NXF1, promoting mRNA nuclear export. RNA-binding is semi-sequence specific.
A2A4P0	Dhx8	ATP-dependent RNA helicase DHX8 (EC 3.6.4.13) (DEAH box protein 8)	FUNCTION: Involved in pre-mRNA splicing as component of the spliceosome. Facilitates nuclear export of spliced mRNA by releasing the RNA from the spliceosome.
Q99M28	Rnps1	RNA-binding protein with serine-rich domain 1	FUNCTION: Part of pre- and post-splicing multiprotein mRNP complexes. Auxiliary component of the splicing-dependent multiprotein exon junction complex (EJC) deposited at splice junction on mRNAs. The EJC is a dynamic structure consisting of core proteins and several peripheral nuclear and cytoplasmic associated factors that join the complex only transiently either during EJC assembly or during subsequent mRNA metabolism. Component of the ASAP and PSAP complexes which bind RNA in a sequence-independent manner and are proposed to be recruited to the EJC prior to or during the splicing process and to regulate specific excision of introns in specific transcription subsets. The ASAP complex can inhibit RNA processing during in vitro splicing reactions. The ASAP complex promotes apoptosis and is disassembled after induction of apoptosis. Enhances the formation of the ATP-dependent A complex of the spliceosome. Involved in both constitutive splicing and, in association with SRP54 and TRA2B/SFRS10, in distinctive modulation of alternative splicing in a substrate-dependent manner. Involved in the splicing modulation of BCL2L1/Bcl-X (and probably other apoptotic genes); specifically inhibits formation of proapoptotic isoforms such as Bcl-X(S); the activity is different from the established EJC assembly and function. Participates in mRNA 3'-end cleavage. Involved in UPF2-dependent nonsense-mediated decay (NMD) of mRNAs containing premature stop codons. Also mediates increase of mRNA abundance and translational efficiency. Binds spliced mRNA 20-25 nt upstream of exon-exon junctions (By similarity).
A2AJT4	Pnisr	Arginine/serine-rich protein PNISR (Serine/arginine-rich-splicing regulatory protein 130) (SRrp130) (Splicing factor, arginine/serine-rich 130) (Splicing factor, arginine/serine-rich 18)	
Q69ZA1	Cdk13	Cyclin-dependent kinase 13 (EC 2.7.11.22) (EC 2.7.11.23) (CDC2-related protein kinase 5) (Cell division cycle 2-like protein kinase 5) (Cell division protein kinase 13)	FUNCTION: Cyclin-dependent kinase which displays CTD kinase activity and is required for RNA splicing. Has CTD kinase activity by hyperphosphorylating the C-terminal heptapeptide repeat domain (CTD) of the largest RNA polymerase II subunit RPB1, thereby acting as a key regulator of transcription elongation. Required for RNA splicing, probably by phosphorylating SRSF1/SF2. Required during hematopoiesis.
Q99ME9	Gtpbp4	Nucleolar GTP-binding protein 1 (Chronic renal failure gene protein) (GTP-binding protein NGB)	FUNCTION: Involved in the biogenesis of the 60S ribosomal subunit.
Q8K194	Snrnp27	U4/U6.U5 small nuclear ribonucleoprotein 27 kDa protein (U4/U6.U5 snRNP 27 kDa protein) (U4/U6.U5-27K) (U4/U6.U5 tri-snRNP-associated protein 3)	FUNCTION: May play a role in mRNA splicing.
Q9R0U0	Srsf10	Serine/arginine-rich splicing factor 10 (FUS-interacting serine-arginine-rich protein 1) (Neural-salient serine/arginine-rich protein) (Neural-specific SR protein) (Splicing factor, arginine/serine-rich 13A) (TLS-associated protein with Ser-Arg repeats) (TASR) (TLS-associated protein with SR repeats) (TLS-associated serine-arginine protein) (TLS-associated SR protein)	FUNCTION: Splicing factor that in its dephosphorylated form acts as a general repressor of pre-mRNA splicing. Seems to interfere with the U1 snRNP 5'-splice recognition of SNRNP70. Required for splicing repression in M-phase cells and after heat shock. Also acts as a splicing factor that specifically promotes exon skipping during alternative splicing. Interaction with YTHDC1, a RNA-binding protein that recognizes and binds N6-methyladenosine (m6A)-containing RNAs, prevents SRSF10 from binding to its mRNA-binding sites close to m6A-containing regions, leading to inhibit exon skipping during alternative splicing (By similarity). May be involved in regulation of alternative splicing in neurons (PubMed:10583508).
Q9R020	Zranb2	Zinc finger Ran-binding domain-containing protein 2 (Zinc finger protein 265) (Zinc finger, splicing)	FUNCTION: Splice factor required for alternative splicing of TRA2B/SFRS10 transcripts. May interfere with constitutive 5'-splice site selection (By similarity).
Q61136	Prpf4b	Serine/threonine-protein kinase PRP4 homolog (EC 2.7.11.1) (PRP4 pre-mRNA-processing factor 4 homolog) (Pre-mRNA protein kinase)	FUNCTION: Has a role in pre-mRNA splicing. Phosphorylates SF2/ASF.
Q9Z0U1	Tjp2	Tight junction protein ZO-2 (Tight junction protein 2) (Zona occludens protein 2) (Zonula occludens protein 2)	FUNCTION: Plays a role in tight junctions and adherens junctions.
Q7TNC4	Luc7l2	Putative RNA-binding protein Luc7-like 2 (CGI-74 homolog)	FUNCTION: May bind to RNA via its Arg/Ser-rich domain.

Table S3 (Related to Figure 2). RNF12 phosphorylation sites identified by immunoprecipitation-mass spectrometry

**Experiment 1**					
**pep_exp_mz**	**pep_exp_mr**	**pep_score**	**pep_seq**	**pep_var_mod**	**residue**
516.5920	1546.7504	23	R.SRSPLQPTSEIPR.R	P (ST)	S227, S229
801.8524	1601.6868	(26)	R.RL**S**VENMESSSQR.Q	P (ST)	S163
809.8495	1617.6818	(27)	R.RL**S**VENMESSSQR.Q	O (M); P (ST)	S163
**Experiment 2**					
**pep_exp_mz**	**pep_exp_mr**	**pep_score**	**pep_seq**	**pep_var_mod**	**residue**
701.786	1401.5562	30	EGPPPQ**S**PDENR	P (ST)	S78
774.3826	1546.7504	40	SR**S**PLQPTSEIPR	P (ST)	S229
801.8502	1601.6868	46	RL**S**VENMESSSQR	P (ST)	S163
809.8483	1617.6818	62	RL**S**VENMESSSQR	O (M); P (ST)	S163
1230.5322	2459.0489	78	AGESSDDVTNSDSIIDWLNSVR	P (ST)	S88, S89
1255.2161	3762.6282	32	EGPPPQSPDENRAGESSDDVTNSDSIIDWLNSVR	P (ST)	S78, S88, S89
**Experiment 3**					
**pep_exp_mz**	**pep_exp_mr**	**pep_score**	**pep_seq**	**pep_var_mod**	**residue**
701.7868	1401.559	48	EGPPPQ**S**PDENR	P (ST)	S78
774.3839	1546.7531	44	SRSPLQPTSEIPR	P (ST)	S227
516.5918	1546.7536	27	SRSPLQPTSEIPR	P (ST)??	S227, S229, T234
809.8502	1617.6858	68	RL**S**VENMESSSQR	O (M); P (ST)	S163
814.3668	1626.719	37	**S**R**S**PLQPTSEIPR	2 P (ST)	S227, S229
661.9747	1982.9024	19	AERSRSPLQPTSEIPR	2 P (ST)??	S227, S229, T234, S235
**Experiment 4**					
**pep_exp_mz**	**pep_exp_mr**	**pep_score**	**pep_seq**	**pep_var_mod**	**residue**
474.7062	947.3979	29	SRSPEHR	P (ST)??	S212, S214
701.7858	1401.5571	30	EGPPPQ**S**PDENR	P (ST)	S78
774.3807	1546.7469	43	SR**S**PLQPTSEIPR	P (ST)	S229
809.8483	1617.6821	50	RLSVENMESSSQR	O (M); P (ST)??	S163
814.3668	1626.719	22	SRSPLQPTSEIPR	2 P (ST)??	S227, S229, T234, S235
905.9172	1809.8198	21	AERNSAEAVTEVPTTR	P (ST)??	S194, T199
1230.5315	2459.0484	73	AGESSDDVTNSDSIIDWLNSVR	P (ST)??	S88, S89, T93,
1255.2163	3762.6271	39	EGPPPQSPDENRAGESSDDVTNSDSIIDWLNSVR	P (ST)	S78

Phosphosite Localisation data obtained from Proteome Discoverer 1.4-SP1 –PhosphoRS3.1 or Proteome Discoverer 2.0-ptmRS. Underlined S T is interpretation of Mascot and MS2 data. Bold S T is a very good assignment, S T is used where identification is not certain, ?? means phosphorylation could be in any of the sites. pep_exp_mz: Observed or experimental m/z value, pep_exp_mr: Molecular mass calculated from experimental m/z value, pep_score: Mascot score for PSM (Peptide sequence match), pep_seq: Peptide sequence in 1 letter code, pep_var_mod: Variable modifications from all sources as list of names.

Table S4 (Related to Figure 2). RNF12 phosphorylation sites identified via SRPK in vitro phosphorylation and mass spectrometry

**SRPK1 5 min**				
**pep_exp_mz**	**pep_exp_mr**	**pep_score**	**pep_seq**	**pep_var_mod**
460.2178	918.4212	(24)	R.TYVSTIR.I	P (ST)
639.3107	1276.6176	(20)	R.QQI**S**GPELLGR.G	P (ST)
652.8156	1303.6173	(31)	R.**S**PLQPTSEIPR.R	P (ST)
516.5908	1546.7504	(22)	R.SRSPLQPTSEIPR.R	P (ST)
774.3820	1546.7504	(47)	R.SRSPLQPTSEIPR.R	P (ST)
543.2460	1626.7168	(26)	R.**S**R**S**PLQPTSEIPR.R	2 P (ST)
814.3652	1626.7168	(41)	R.**S**R**S**PLQPTSEIPR.R	2 P (ST)
838.3497	1674.6886	(46)	R.**S**QAPNNTVTYESER.G	P (ST)
959.9175	1917.8218	85	R.SR**S**QAPNNTVTYESER.G	P (ST)
640.2810	1917.8218	(48)	R.SRSQAPNNTVTYESER.G	P (ST)
992.4553	1982.8976	(28)	R.AERSRSPLQPTSEIPR.R	2 P (ST)
661.9727	1982.8976	40	R.AERSRSPLQPTSEIPR.R	2 P (ST)
1027.4431	2052.8749	(29)	R.RAPTLEQSSENEPEGSSR.T	P (ST)
685.2983	2052.8749	(60)	R.RAPTLEQSSENEPEGSSR.T	P (ST)
689.6444	2065.9205	(21)	R.DNNLLGTPGESTEEELLR.R	P (ST)
771.0147	2310.0237	38	R.RAPTLEQSSENEPEGSSRTR.H	P (ST)
**SRPK1 60 min**				
**pep_exp_mz**	**pep_exp_mr**	**pep_score**	**pep_seq**	**pep_var_mod**
460.2179	918.4212	(19)	R.TYVSTIR.I	P (ST)
514.6891	1027.3637	19	R.**S**R**S**PEHR.R	2 P (ST)
628.2581	1254.5020	21	R.AR**S**R**S**PEHR.R	2 P (ST)
516.5908	1546.7504	(27)	R.SRSPLQPTSEIPR.R	P (ST)
774.3821	1546.7504	50	R.SRSPLQPTSEIPR.R	P (ST)
814.3652	1626.7168	(41)	R.**S**R**S**PLQPTSEIPR.R	2 P (ST)
543.2462	1626.7168	(21)	R.**S**R**S**PLQPTSEIPR.R	2 P (ST)
838.3506	1674.6886	(69)	R.**S**QAPNNTVTYESER.G	P (ST)
949.3937	1896.7738	(50)	R.APTLEQSSENEPEGSSR.T	P (ST)
959.9170	1917.8218	82	R.SR**S**QAPNNTVTYESER.G	P (ST)
640.2809	1917.8218	(29)	R.SRSQAPNNTVTYESER.G	P (ST)
992.4556	1982.8976	39	R.AER**S**R**S**PLQPTSEIPR.R	2 P (ST)
1027.4439	2052.8749	(48)	R.RAPTLEQSSENEPEGSSR.T	P (ST)
685.2986	2052.8749	(50)	R.RAPTLEQSSENEPEGSSR.T	P (ST)
752.6523	2254.9369	35	R.TRSRSQAPNNTVTYESER.G	2 P (ST)
771.0147	2310.0237	43	R.RAPTLEQSSENEPEGSSRTR.H	P (ST)
578.5131	2310.0237	(19)	R.RAPTLEQSSENEPEGSSRTR.H	P (ST)
**SRPK2 5 min**				
**pep_exp_mz**	**pep_exp_mr**	**pep_score**	**pep_seq**	**pep_var_mod**
473.2131	944.4117	35	GLFAA**S**GSR	P (ST)
516.5906	1546.7499	33	SRSPLQPTSEIPR	P (ST)
543.2464	1626.7174	18	**S**R**S**PLQPTSEIPR	2 P (ST)
553.516	2210.0349	24	ARAERSRSPLQPTSEIPR	2 P (ST)
578.5132	2310.0236	38	RAPTLEQSSENEPEGSSRTR	P (ST)
635.3174	1902.9303	38	AERSRSPLQPTSEIPR	P (ST)
640.2807	1917.8203	38	SRSQAPNNTVTYESER	P (ST)
661.9725	1982.8958	29	AERSRSPLQPTSEIPR	2 P (ST)
685.2986	2052.8741	51	RAPTLEQSSENEPEGSSR	P (ST)
718.9813	2153.922	31	APTLEQSSENEPEGSSRTR	P (ST)
750.8328	1499.651	20	AVSRTNPNSGDFR	P (ST)
752.6522	2254.9348	42	TRSRSQAPNNTVTYESER	2 P (ST)
771.0148	2310.0225	51	RAPTLEQSSENEPEGSSRTR	P (ST)
774.382	1546.7495	47	SRSPLQPTSEIPR	P (ST)
814.3648	1626.7151	25	**S**R**S**PLQPTSEIPR	2 P (ST)
814.3654	1626.7162	21	SRSPLQPTSEIPR	2 P (ST)
838.3527	1674.6909	57	**S**QAPNNTVTYESER	P (ST)
959.9179	1917.8213	69	SRSQAPNNTVTYESER	P (ST)
992.455	1982.8955	27	AERSRSPLQPTSEIPR	2 P (ST)
1027.4439	2052.8731	36	RAPTLEQSSENEPEGSSR	P (ST)
1106.0253	2210.036	18	ARAERSRSPLQPTSEIPR	2 P (ST)
**SRPK2 60 min**				
**pep_exp_mz**	**pep_exp_mr**	**pep_score**	**pep_seq**	**pep_var_mod**
460.2179	918.4212	31	TYVSTIR	P (ST)
473.213	944.4115	55	GLFAA**S**GSR	P (ST)
493.2267	984.4388	20	DSIASRTR	P (ST)
500.8911	1499.6514	25	AVSRTNPNSGDFR	P (ST)
516.5907	1546.7503	36	SRSPLQPTSEIPR	P (ST)
534.7452	1067.4759	21	NVERVE**S**R	P (ST)
534.9023	1601.6852	21	RLSVENMESSSQR	P (ST)
540.2346	1617.6819	35	RL**S**VENMESSSQR	O (M); P (ST)
543.2468	1626.7187	28	**S**R**S**PLQPTSEIPR	2 P (ST)
578.5133	2310.0241	50	RAPTLEQSSENEPEGSSRTR	P (ST)
604.2824	1809.8254	29	AERN**S**AEAVTEVPTTR	P (ST)
633.2654	1896.7743	42	APTLEQSSENEPEGSSR	P (ST)
640.2813	1917.8221	45	SRSQAPNNTVTYESER	P (ST)
652.8159	1303.6172	25	SPLQPTSEIPR	P (ST)
661.9733	1982.8982	30	AERSRSPLQPTSEIPR	2 P (ST)
685.2987	2052.8743	55	RAPTLEQSSENEPEGSSR	P (ST)
711.9543	2132.8412	33	RAPTLEQSSENEPEGSSR	2 P (ST)
718.9818	2153.9236	27	APTLEQSSENEPEGSSRTR	P (ST)
725.9974	2174.9703	23	TRSRSQAPNNTVTYESER	P (ST)
750.8325	1499.6505	32	AVSRTNPNSGDFR	P (ST)
752.6528	2254.9365	49	TRSRSQAPNNTVTYESER	2 P (ST)
771.0143	2310.021	31	RAPTLEQSSENEPEGSSRTR	P (ST)
771.0155	2310.0247	26	RAPTLEQSSENEPEGSSRTR	P (ST)

774.382	1546.7495	47	<u>S</u>R<u>S</u>PLQPTSEIPR	P (ST)
801.8499	1601.6853	70	RL<u>**S**</u>VENMESSSQR	P (ST)
809.8482	1617.6819	40	RL<u>**S**</u>VENMESSSQR	O (M); P (ST)
814.3657	1626.7169	45	<u>**S**</u>R<u>**S**</u>PLQPTSEIPR	2 P (ST)
838.3528	1674.6911	58	<u>**S**</u>QAPNNTVTYESER	P (ST)
854.3486	1706.6827	19	<u>S</u>R<u>S</u>PLQP<u>TS</u>EIPR	3 P (ST)
949.3945	1896.7744	58	APTLEQSSENEPEG<u>SS</u>R	P (ST)
959.9175	1917.8204	79	SR<u>**S**</u>QAPNNTVTYESER	P (ST)
992.4556	1982.8966	24	AER<u>S</u>R<u>S</u>PLQP<u>TS</u>EIPR	2 P (ST)
1027.4443	2052.8741	61	RAPTLEQSSENEPEG<u>SS</u>R	P (ST)

Phosphosite Localisation data obtained from Proteome Discoverer 1.4-SP1 –PhosphoRS3.1 or Proteome Discoverer 2.0-ptmRS. Underlined S T is interpretation of Mascot and MS2 data. Bold S T is a very good assignment, S T is used where identification is not certain, ?? means phosphorylation could be in any of the sites. pep_exp_mz: Observed or experimental m/z value, pep_exp_mr: Molecular mass calculated from experimental m/z value, pep_score: Mascot score for PSM (Peptide sequence match), pep_seq: Peptide sequence in 1 letter code, pep_var_mod: Variable modifications from all sources as list of names.

Table S5 (Related to Figure 5). Genes assigned to terms related to 'Neuron' identified by Gene Ontology analysis

GO: 0048699	GO: 0030182	GO: 0031175	GO: 0048666	GO: 0048812	GO: 0045664	GO: 0010975	GO: 0048667	GO: 0045665	GO: 0010976	GO: 0010977	GO: 0097485	GO: 0045666	GO: 0051402	GO: 1990138
Pcsk9	Pcsk9	Inpp5f	Inpp5f	Unc5a	Inpp5f	Inpp5f	Unc5a	Inpp5f	Rrn3	Inpp5f	Unc5a	Rrn3	Pcsk9	Apoe
Inpp5f	Inpp5f	Rrn3	Rrn3	Foxp1	Rrn3	Rrn3	Foxp1	Rap1gap	Rapgef1	Apoe	Foxp1	Rapgef1	Arrb1	Ddr1
Rrn3	Rrn3	Unc5a	Unc5a	Adarb1	Rapgef1	Rapgef1	Adarb1	Apoe	Abl2	Cit	Agrn	Abl2	Adarb1	Dpysl2
Ttbk1	Unc5a	Rapgef1	Rapgef1	Arhgef28	Rap1gap	Abl2	Arhgef28	Cd24a	Ap2a1	Crmp1	Ank3	Ap2a1	Egln3	Dclk1
Unc5a	Rapgef1	Foxp1	Foxp1	Abl2	Abl2	Agrn	Abl2	Cit	Apoe	Epha4	Boc	Apoe	Agrn	Bcl11a
Rapgef1	Foxp1	Mcf2	Mcf2	Agrn	Agrn	Ap2a1	Agrn	Crmp1	Bmp4	Bcl11a	Apbb2	Bmp4	Apoe	Fn1
Foxp1	Mcf2	Adarb1	Adarb1	Ank3	Ap2a1	Apoe	Ank3	Dll1	Camk2b	Ptk2	Bmpr1b	Camk2b	Hyou1	Impact
Mcf2	Ehmt2	Arhgef28	Arhgef28	Boc	Apoe	Bmp4	Boc	Epha4	Cd24a	Gfap	Runx3	Cd24a	Cacna1a	Arhgap4
Ehmt2	Rap1gap	Abl2	Abl2	Apbb2	Bmp4	Cacna1a	Apbb2	Bcl11a	Cnr1	H2-D1	Crmp1	Cnr1	Casp3	Mag
Rap1gap	Adarb1	Agrn	Agrn	Apoe	Cacna1a	Camk2b	Apoe	Ptk2	Cobl	H2-K1	Dpysl2	Cobl	Cit	Map1b
Adarb1	Arhgef28	Ank3	Ank3	Bmpr1b	Camk2b	Cd24a	Atp2b2	Gfap	Crabp2	Lgals1	Enah	Crabp2	Coro1a	Myo5b
Arhgef28	Abl2	Boc	Boc	Cacna1a	Cd24a	Cit	Bmpr1b	H2-D1	Dlg4	Lrp1	Epha4	Dlg4	Fgf8	Ntn1
Abl2	Agrn	Ap2a1	Ap2a1	Ddr1	Cit	Cnr1	Cacna1a	H2-K1	Eef2k	Arhgap4	Epha8	Eef2k	Fzd9	Ntrk3
Agrn	Ank3	Apbb2	Apbb2	Camk2a	Cnr1	Cobl	Camk2a	Jag1	Enc1	Mag	Ephb3	Enc1	Gclm	Pak1
Ank3	Boc	Apoe	Apoe	Camk2b	Cobl	Crabp2	Camk2b	Lgals1	Epha4	Ngfr	Ext1	Epha4	Glp1r	Pou4f2
Boc	Ap2a1	Atp2b2	Atp2b2	Runx3	Crabp2	Crmp1	Runx3	Lrp1	Bcl11a	Ntn1	Fgf8	Bcl11a	Grik5	Ptprs
Ap2a1	Apbb2	Bmp4	Prdm1	Cit	Crmp1	Dpysl2	Cit	Arhgap4	Fgfr1	Pmp22	Flot1	Fgfr1	Lrp1	Sema3f
Apbb2	Apoe	Bmpr1b	Bmp4	Cobl	Dpysl2	Dlg4	Cobl	Mag	Fn1	Ptprs	Gap43	Fn1	Mag	Sema4a
Apoe	Atp2b2	Cacna1a	Bmpr1b	Crabp2	Dlg4	Eef2k	Crabp2	Ngfr	Ikbkb	Stmn2	Gbx2	Ikbkb	Mt3	Sema6b
Atp2b2	Prdm1	Ddr1	Cacna1a	Crmp1	Dll1	Enc1	Crmp1	Notch1	Map1b	Sema3f	Foxd1	Impact	Mybl2	Sema6c
Prdm1	Bmp4	Camk2a	Ddr1	Dpysl2	Eef2k	Epha4	Dpysl2	Ntn1	Myo5b	Sema4a	Matn2	Map1b	Nf1	Sema7a
Bmp4	Bmpr1b	Camk2b	Camk2a	Dclk1	Enc1	Ephb3	Dclk1	Pmp22	Neu1	Sema6b	Ngfr	Myo5b	Ngfr	Tiam1
Bmpr1b	Cacna1a	Casp3	Camk2b	Dlg4	Epha4	Bcl11a	Dlg4	Pou4f2	Nf1	Sema6c	Ntn1	Neu1	Prkcg	Nrn1l
Tspo	Ddr1	Runx3	Casp3	Eef2k	Ephb3	Ptk2	Eef2k	Ptprs	Ngfr	Sema7a	Etv4	Neurod1	Prkci	Twf2
Cacna1a	Camk2a	Cd24a	Runx3	Enah	Bcl11a	Fgfr1	Enah	Stmn2	Ntn1	Thy1	Pou4f2	Nf1	Lgmn	Cpne5
Ddr1	Camk2b	Cit	Cd24a	Epha4	Ptk2	Fn1	Epha4	Sema3f	Ntrk3	Tsc2	Ptch1	Ngfr	Sod1	Sema4g
Camk2a	Casp3	Cnr1	Cit	Epha8	Fgfr1	Gfap	Epha8	Sema4a	P2ry2	Dpysl3	Reln	Ntn1	Sod2	Cpne1
Camk2b	Runx3	Cobl	Cnr1	Ephb3	Fn1	H2-D1	Ephb3	Sema6b	Pak1	Vim	Robo1	Ntrk3	Srpk2	Dbn1
Casp3	Cd24a	Crabp2	Cobl	Bcl11a	Gfap	H2-K1	Bcl11a	Sema6c	Palm	Klk8	Sema3f	P2ry2	Hdac4	Map3k13
Runx3	Cit	Crmp1	Crabp2	Ext1	H2-D1	Ikbkb	Ext1	Sema7a	Prkci	Sema4g	Sema4a	Pak1	Stxbp1	Cyfip2
Cd24a	Cnr1	Dpysl2	Crmp1	Ptk2	H2-K1	Kif13b	Ptk2	Cntn2	Pou4f2	Rtn4rl2	Sema6b	Palm	Trp73	
Cdh1	Cobl	Dclk1	Dpysl2	Fgf8	Ikbkb	Lgals1	Fgf8	Thy1	Reln	Lrig2	Sema6c	Prkci	Vegfb	
Celsr1	Crabp2	Dlg4	Dclk1	Flot1	Impact	Lrp1	Flot1	Trp73	Robo1	Rap1gap2	Sema7a	Pou4f2	Wfs1	
Cit	Crmp1	Eef2k	Dlg4	Fn1	Jag1	Arhgap4	Fn1	Tsc2	Stmn2	Carm1	Cntn2	Rara	Axl	
Cnr1	Dpysl2	Enah	Eef2k	Gap43	Kif13b	Mag	Gap43	Dpysl3	Sema7a	Ctsz	Rnf165	Reln	Vstm2l	
Cobl	Dclk1	Enc1	Enah	Gbx2	Lgals1	Map1b	Gbx2	Vim	Skil	Itm2c	Plxnb1	Robo1	Ctsz	
Crabp2	Dlg4	Epha4	Enc1	Gja1	Lrp1	Myo5b	Foxd1	Klk8	Creb3l2	Nfatc4	Sema4g	Stmn2	Fam162a	
Crmp1	Dll1	Epha8	Epha4	Foxd1	Rac3	Neu1	Kif13b	Sema4g	Plk5		Pla2g10	Sema7a	Pigt	
Dpysl2	Eef2k	Ephb3	Epha8	Impact	Arhgap4	Nf1	Stmn1	Rtn4rl2	Ppp2r5d		Vstm2l	Skil	Trim2	
Dclk1	Enah	Bcl11a	Ephb3	Kif13b	Mag	Ngfr	Arhgap4	Lrig2	Tiam1		Nrcam	Sox11		
Dlg4	Enc1	Ext1	Bcl11a	Stmn1	Map1b	Ntn1	Mag	Rap1gap2	Tsc2		Ccdc141	Creb3l2		
Dll1	Epha2	Ptk2	Ext1	Lifr	Myo5b	Ntrk3	Matn2	Zhx2	Dpysl3		Bcl11b	Plk5		
Dll3	Epha4	Fgf8	Ptk2	Arhgap4	Neu1	P2ry2	Map1a	Carm1	Tbc1d24		Cyfip2	Ppp2r5d		
Eef2k	Epha8	Fgfr1	Fgf8	Mag	Neurod1	Pak1	Map1b	Ctsz	Zmynd8		Vangl2	Tiam1		
Enah	Ephb3	Flot1	Fgfr1	Matn2	Nf1	Palm	Myo5b	Itm2c	Plxnb1			Tsc2		
Enc1	Bcl11a	Fn1	Flot1	Map1a	Ngfr	Prkci	Ngfr	Casz1	Pacsin1			Dpysl3		
Epha2	Ext1	Gap43	Fn1	Map1b	Mycn	Pmp22	Notch1	Nfatc4	Twf2			Tbc1d24		
Epha4	Ptk2	Gbx2	Gap43	Myo5b	Notch1	Pou4f2	Ntn1		Cpne5			Zmynd8		
Epha8	Fgf8	Gfap	Gbx2	Ngfr	Ntn1	Ptprs	Tbc1d24		Ankrd27			Plxnb1		
Ephb3	Fgfr1	Gfra1	Gfap	Notch1	Ntrk3	Reln	Ntrk3		Mapk6			Pacsin1		
Bcl11a	Flot1	Gja1	Gfra1	Ntn1	P2ry2	Robo1	Pak1		Dbn1			Twf2		
Ext1	Fn1	H2-D1	Gja1	Tbc1d24	Pak1	Stmn2	Etv4		Shank3			Cpne5		
Ptk2	Gap43	H2-K1	Gnat2	Ntrk3	Palm	Sema3f	Pmp22		Actr2			Ankrd27		
Fgf8	Gbx2	Foxd1	H2-D1	Pak1	Prkci	Sema4a	Pou4f2		Dab2ip			Cpne1		
Fgfr1	Gfap	Ikbkb	H2-K1	Etv4	Pmp22	Sema6b	Ptch1		Ptk7			Mapk6		
Flot1	Gfra1	Impact	Foxd1	Pmp22	Pou4f2	Sema6c	Ptprs		Map3k13			Dbn1		
Fn1	Gja1	Atcay	Ikbkb	Pou4f2	Ptprs	Sema7a	Reln					Shank3		
Fzd9	Gnat2	Kif13b	Impact	Ptch1	Rara	Sfrp1	Robo1					Actr2		
Gap43	Nkx6-2	Stmn1	Atcay	Ptprs	Reln	Skil	Sema3f					Dab2ip		
Gas6	H2-D1	Lgals1	Kif13b	Rac2	Robo1	Creb3l2	Sema4a					Ptk7		
Gbx2	H2-K1	Lifr	Stmn1	Reln	Stmn2	Cntn2	Sema6b					Map3k13		
Gfap	Foxd1	Lrp1	Lgals1	Robo1	Sema3f	Plk5	Sema6c					Kdm4c		
Gfra1	Ikbkb	Rac3	Lifr	Sema3f	Sema4a	Ppp2r5d	Sema7a					Lin28a		
Gja1	Impact	Arhgap4	Lrp1	Sema4a	Sema6b	Thy1	Skil							
Gnat2	Jag1	Mag	Rac3	Sema6b	Sema6c	Tiam1	Sod1							
Nkx6-2	Atcay	Matn2	Arhgap4	Sema6c	Sema7a	Tsc2	Stxbp1							
H2-D1	Kif13b	Mmp2	Mag	Sema7a	Sfrp1	Dpysl3	Cntn2							
H2-K1	Stmn1	Map1a	Matn2	Skil	Skil	Vim	Thy1							
Hes2	Lgals1	Map1b	Mmp2	Stxbp1	Sox11	Tbc1d24	Tiam1							
Foxd1	Lifr	Myo5b	Map1a	Cntn2	Creb3l2	Zmynd8	Tsc2							
Ikbkb	Lrp1	Neu1	Map1b	Thy1	Cntn2	Arhgap33	Uchl1							
Il6st	Rac3	Nf1	Myo5b	Tiam1	Plk5	Plxnb1	Vim							
Impact	Mcoln3	Ngfr	Neu1	Tsc2	Ppp2r5d	Pacsin1	Tbc1d24							
Jag1	Arhgap4	Notch1	Neurod1	Uchl1	Thy1	Twf2	Rnf165							
Atcay	Mag	Ntn1	Nf1	Vim	Tiam1	Cpne5	Arhgap33							
Kif13b	Matn2	Tbc1d24	Ngfr	Tbc1d24	Trp73	Ankrd27	Plxnb1							
Stmn1	Mmp2	Ntrk3	Notch1	Rnf165	Tsc2	Klk8	Twf2							
Ldlr	Map1a	P2ry2	Ntn1	Arhgap33	Dpysl3	Sema4g	Ankrd27							
Lef1	Map1b	Pak1	Tbc1d24	Nrn1l	Vim	Rtn4rl2	Sema4g							
Lgals1	Myo5b	Palm	Ntrk3	Plxnb1	Tbc1d24	Lrig2	Pla2g10							
Lifr	Neu1	Etv4	P2ry2	Pacsin1	Zmynd8	Prex1	Vstm2l							
Lrp1	Neurod1	Prkci	Pak1	Twf2	Arhgap33	Nrcam	Nrcam							
Rac3	Nf1	Pmp22	Palm	Cpne5	Plxnb1	Rap1gap2	Adcy1							
Mcoln3	Ngfr	Pou4f2	Etv4	Kif20b	Pacsin1	Mapk6	Ccdc141							
Arhgap4	Mycn	Ptch1	Prkci	Nyap1	Twf2	Srcin1	Srcin1							
Mag	Notch1	Ptprs	Pmp22	Ankrd27	Cpne5	Dbn1	Dbn1							
Matn2	Ntn1	Rac2	Pou4f2	Klk8	Ankrd27	Shank3	Bcl11b							
Mmp2	Tbc1d24	Reln	Ptch1	Sema4g	Klk8	Carm1	Shank3							
Mt3	Ntrk3	Robo1	Ptprs	Pla2g10	Sema4g	Ctsz	Actr2							
Map1a	P2ry2	Stmn2	Rac2	Cpne1	Cpne1	Itm2c	Map3k13							
Map1b	Pak1	Sema3f	Reln	Vstm2l	Rtn4rl2	Actr2	Nfatc4							
Myo5b	Palm	Sema4a	Robo1	Nrcam	Lrig2	Dab2ip	Cyfip2							
Neu1	Etv4	Sema6b	Rpgr	Adcy1	Prex1	Ptk7	Vangl2							
Neurod1	Prkci	Sema6c	Stmn2	Ccdc141	Nrcam	Map3k13	Ophn1							
Nf1	Pmp22	Sema7a	Sema3f	Srcin1	Rap1gap2	Nfatc4								
Ngfr	Pou4f2	Sfrp1	Sema4a	Dbn1	Zhx2									
Mycn	Prom1	Skil	Sema6b	Bcl11b	Mapk6									
Notch1	Ptch1	Sod1	Sema6c	Shank3	Srcin1									
Ntn1	Ptprs	Creb3l2	Sema7a	Actr2	Dbn1									
Tbc1d24	Rac2	Stx3	Sfrp1	Dab2ip	Bcl11b									
Ntrk3	Rara	Stxbp1	Skil	Map3k13	Shank3									
P2ry2	Reln	Cntn2	Sod1	Nfatc4	Carm1									
Pak1	Robo1	Plk5	Sod2	Cyfip2	Ctsz									
Palm	Rpgr	Ppp2r5d	Creb3l2	Vangl2	Itm2c									
Etv4	Stmn2	Thy1	Stx3		Actr2									
Prkci	Sema3f	Tiam1	Stxbp1		Dab2ip									
Pmp22	Sema4a	Tsc2	Cntn2		Casz1									
Pou4f2	Sema6b	Tulp1	Plk5		Ptk7									
Prom1	Sema6c	Uchl1	Ppp2r5d		Map3k13									
Ptch1	Sema7a	Dpysl3	Thy1		Nfatc4									
Ptprs	Sfrp1	Vim	Tiam1		Kdm4c									
Rac2	Skil	Tbc1d24	Trp73		Lin28a									
Rara	Sod1	Rnf165	Tsc2											
Reln	Sod2	Zmynd8	Tulp1											
Robo1	Sox11	Arhgap33	Uchl1											
Rpgr	Sox4	Nrn1l	Dpysl3											
Stmn2	Stat3	Plxnb1	Vim											

Sema3f	Creb3l2	Pacsin1	Tbc1d24											
Sema4a	Stx3	Twf2	Rnf165											
Sema6b	Stxbp1	Cpne5	Zmynd8											
Sema6c	Cntn2	Kif20b	Arhgap33											
Sema7a	Plk5	Nyap1	Nrn1l											
Sfrp1	Ppp2r5d	Ankrd27	Plxnb1											
Skil	Tgm3	Klk8	C1ql1											
Sod1	Thy1	Sema4g	Pacsin1											
Sod2	Tiam1	Pla2g10	Twf2											
Sox11	Trp73	Cpne1	Cpne5											
Sox4	Tsc2	Rtn4rl2	Kif20b											
Stat3	Tulp1	Lrig2	Nyap1											
Creb3l2	Tulp3	Micall1	Ankrd27											
Stx3	Uchl1	Prex1	Klk8											
Stxbp1	Dpysl3	Vstm2l	Sema4g											
Cntn2	Vim	Nrcam	Pla2g10											
Nav1	Wnt5b	Rap1gap2	Cpne1											
Plk5	Wnt6	Adcy1	Rtn4rl2											
Ppp2r5d	Tbc1d24	Mapk6	Lrig2											
Tgfb1	Rnf165	Nptx2	Micall1											
Tgm3	Zmynd8	Ccdc141	Naglu											
Thy1	Arhgap33	Srcin1	Prex1											
Tiam1	Nrn1l	Dbn1	Vstm2l											
Trp73	Plxnb1	Slc12a5	Nrcam											
Tsc2	C1ql1	Bcl11b	Cngb1											
Tulp1	Pacsin1	Shank3	Rap1gap2											
Tulp3	Twf2	Carm1	Adcy1											
Uchl1	Cpne5	Trappc4	Mapk6											
Dpysl3	Kif20b	Ctsz	Nptx2											
Vim	Nyap1	Itm2c	Ccdc141											
Wnt5b	Ankrd27	Actr2	Srcin1											
Wnt6	Klk8	Cthrc1	Dbn1											
Tbc1d24	Sema4g	Dab2ip	Slc12a5											
Rnf165	Pla2g10	Ptk7	Bcl11b											
Zmynd8	Cpne1	Map3k13	Shank3											
Arhgap33	Rtn4rl2	Nfatc4	Carm1											
Nrn1l	Lrig2	Manf	Trappc4											
Plxnb1	Chd5	Cyfip2	Ctsz											
C1ql1	Micall1	Vangl2	Itm2c											
Pacsin1	Naglu	Clmn	Actr2											
Twf2	Prex1	Ophn1	Cthrc1											
Cpne5	Vstm2l	Efhd1	Dab2ip											
Kif20b	Nrcam		Ptk7											
Nyap1	Cngb1		Map3k13											
Ankrd27	Rap1gap2		Nfatc4											
Klk8	Zhx2		Manf											
Axl	Adcy1		Cyfip2											
Sema4g	Mapk6		Kcnip2											
Pla2g10	Nptx2		Vangl2											
Cpne1	Ccdc141		Clmn											
Rtn4rl2	Srcin1		Ophn1											
Ell3	Dbn1		Efhd1											
Lrig2	Slc12a5													
Chd5	Bcl11b													
Idh2	Shank3													
Micall1	Carm1													
Naglu	Trappc4													
Prex1	Ctsz													
Vstm2l	Itm2c													
Nrcam	Actr2													
Chd7	Cthrc1													
Cngb1	Dab2ip													
Rap1gap2	Casz1													
Zhx2	Ptk7													
Adcy1	Map3k13													
Mapk6	Gpc2													
Nptx2	Nfatc4													
Ccdc141	Manf													
Srcin1	Kdm4c													
Dbn1	Cyfip2													
Plag1	Pigt													
Slc12a5	Kcnip2													
Bcl11b	Lin28a													
Shank3	Smarca1													
Carm1	Vangl2													
Trappc4	Clmn													
Ctsz	Ophn1													
Itm2c	Efhd1													
Actr2														
Smarcd3														
Cthrc1														
Dab2ip														
Casz1														
Ptk7														
Map3k13														
Zcchc24														
Gpc2														
Nfatc4														
Manf														
Kdm4c														
Cyfip2														
Pigt														
Kcnip2														
Lin28a														
Hes7														
Smarca1														
Vangl2														
Clmn														
Ophn1														
Efhd1														

Table S6 (Related to Figure 5). Genes assigned to terms related to 'Neural' identified by Gene Ontology analysis

GO: 0021915	GO: 0001841	GO: 0014020	GO: 0001843	GO: 0090177	GO: 0061351	GO: 0061076	GO: 0001840	GO: 2000177	GO: 0090179	GO: 0090178	GO: 0003407	GO: 0001839	GO: 0014033	GO: 2000178
Abl2	Abl2	Abl2	Abl2	Celsr1	Rapgef1	Pou4f2	Fgf8	Rapgef1	Celsr1	Celsr1	Gnat2	Fgf8	Anxa6	Rapgef1
Bmp4	Bmp4	Bmp4	Bmp4	Sfrp1	Cd24a	Casz1	Ptch1	Cd24a	Sfrp1	Sfrp1	Neurod1	T	Bmp4	Cd24a
Celsr1	Celsr1	Celsr1	Celsr1	Cthrc1	Fgf8		T	Fzd9	Ptk7	Ptk7	Pou4f2	Vangl2	Fn1	Nf1
Cobl	Cobl	Cobl	Cobl	Ptk7	Fgfr1		Vangl2	Gli1	Vangl2	Vangl2	Prom1		Gbx2	Spint2
Enah	Enah	Enah	Enah	Vangl2	Fzd9			Nf1			Megf11		Jag1	Tgfb1
Epha2	Ptch1	Ptch1	Ptch1		Gbx2			Ngfr			Thy1		Lama5	
Fgf8	Rara	Rara	Rara		Kif1a			Notch1			Rab11fip4		Sema3f	
Gbx2	Sfrp1	Sfrp1	Sfrp1		Lef1			Spint2			Smarcd3		Sema4a	
Nf1	Sox11	Spint2	Spint2		Nf1			Tgfb1			Casz1		Sema6b	
Notch1	Sox4	T	T		Ngfr			Ell3					Sema6c	
Ptch1	Spint2	Tead2	Tead2		Notch1			Ctsz					Sema7a	
Rara	T	Tgfb1	Tgfb1		Ncor2			Smarcd3					Sfrp1	
Sfrp1	Tead2	Tsc2	Tsc2		Spint2			Smarca1					Sox11	
Sox11	Tgfb1	Tulp3	Tulp3		Tgfb1								Mapk3	
Sox4	Tsc2	Kif20b	Kif20b		Ell3								Sema4g	
Spint2	Tulp3	Luzp1	Dlc1		Tacc1									
T	Kif20b	Dlc1	Med12		Dbn1									
Tcf7	Luzp1	Med12	Cthrc1		Ctsz									
Tead2	Dlc1	Cthrc1	Ptk7		Smarcd3									
Itpk1	Med12	Ptk7	Vangl2		Smarca1									
Tgfb1	Cthrc1	Vangl2												
Tsc2	Ptk7													
Tulp3	Vangl2													
Kif20b														
Luzp1														
Dlc1														
Med12														
Cthrc1														
Atp6ap2														
Ptk7														
Vangl2														

Table S7 (Related to Figure 5). Disease association of RNF12-regulated genes from the neural crest cell differentiation GO term (GO:0014033).

**Protein name**	**Gene name**	**Neural crest function**	**Craniofacial defects association**
Annexin A6	Anxa6	Modulates chick cranial neural crest cell emigration (Wu and Taneyhill, 2012)	Associated to Treacher Collins syndrome (Dixon et al., 1994)
Bone morphogenetic protein 4	Bmp4	Regulates neural crest migration and differentiation (Li et al., 2018; Sela-Donenfeld and Kalcheim, 1999; Zhu et al., 2019)<br>Regulates tooth morphogenesis (Jia et al., 2016; Shin et al., 2012)	Associated to Non-syndromic cleft lip (Chen et al., 2012, 2014)
Fibronectin	Fn1	Participate in differentiation of NC cells into vascular smooth muscle cells (Wang and Astrof, 2016)	
Homeobox protein GBX-2	Gbx2	Required for pharyngeal arch and cardiovascular development (Byrd and Meyers, 2005)<br>Participates in neural crest induction (Li et al., 2009)	Associated to craniofacial microsomia (Zhang et al., 2016)
Protein jagged-1	Jag1	Induces neural crest stem cell self-renewal and osteoblast differentiation (Kamalakar et al., 2019; Nikopoulos et al., 2007)<br>Regulates face development (Zuniga et al., 2010)<br>Regulates specification of the coronal suture (Yen et al., 2010)<br>Regulates maxillary ossification (Hill et al., 2014)<br>Regulates bone patterning of middle ear (Teng et al., 2017)	Deleted in Alagille Syndrome (Humphreys et al., 2012; Micaglio et al., 2019; Pilia et al., 1999)
Laminin subunit alpha-5	Lama5	Regulates neural crest cell migration (Coles et al., 2006)<br>Required for tooth development (Fukumoto et al., 2006)	
Semaphorin-3F	Sema3f	Regulates cranial neural crest cell migration (York et al., 2018; Yu and Moens, 2005)<br>Regulates trunk neural crest migration (Gammill et al., 2006)	
Semaphorin-4A	Sema4a	Phylogenetic-based functional annotation	
Semaphorin-4G	Sema4g	Phylogenetic-based functional annotation	
Semaphorin-6B	Sema6b	Patterns cardiac neural crest migration (Toyofuku et al., 2008)	
Semaphorin-6C	Sema6c	Phylogenetic-based functional annotation	
Semaphorin-7A	Sema7a	Expressed in cranial and trunk neural crest cells (Bao and Jin, 2006)	Associated to craniofacial microsomia (Zhang et al., 2016)
Secreted frizzled-related protein 1	Sfrp1	Expressed in migrating neural crest cells (Duprez et al., 1999)<br>Regulates Periodontal Mineral Homeostasis (Gopinathan et al., 2019)	
Transcription factor SOX-11	Sox11	Expressed in neural crest and derivatives (Hargrave et al., 1997; Sock et al., 2004)<br>Ablation generates Clefting of the Secondary Palate (Huang et al., 2016)<br>Linked to cranial vault shape in humans (Roosenboom et al., 2018)	
Mitogen-activated protein kinase 3	Mapk3, Erk1	Expressed in neural crest and derivatives (Parada et al., 2015)	

Table S8 (Related to STAR Methods). Primer and Oligonucleotide sequences

Oligonucleotides		
qRT-PCR primers	Forward (5'-3')	Reverse (5'-3')
FoxP1 ex15-16	CACGTGGAAGAATGCAGTGCG	N/A
FoxP1 ex15-16b	CACGTGGAAGGGTGCCATTC	N/A
FoxP1 ex17	N/A	TGAGAGGTGTGCAGTAGGCG
Gapdh	CTCGTCCCGTAGACAAAA	TGAATTTGCCGTGAGTGG
Ntn1	CGCAACTGTACCAGTGACCTCT	TTGCGGCAGTAGATGAGGACGA
Dll1	ACCAAGTGCCAGTCACAGAG	TCCATCTTACACCTCAGTCGC
Kif1a	CACCACTATTGTCAACCCCAAA	CCCCAATGTCCCTGTAGACCT
Gfap	CAATGCTGGCTTCAAGGAGACACG	TCAGTTCAGCTGCCAGCGCCT
Unc5a	GTCTGGTGTGTGACTGTAGGCA	CCGAGCATGGAGGTTGCAGTTG
Primers for genomic DNA sequencing	Forward (5'-3')	Reverse (5'-3')
Srpk1 (KO)	TGCACTAACAGGCACTGTCAGG	CAGGCTCTGGTGAGACCTAGC
Srpk2 (KO)	GTAGAGTAACTGTCTCTGTAAACTTGTGTACTG	CTATAAAGCTGGACCAGGAGAGGC
Zfp42/Rex1 (KO)	TCACCATGGGCTCTCGTATTGG	AGAAGACTCGAGAAGGGAACTCG
Rlim 4xSA/y (KI)	AGAGCAAGAGCTGAAAGAGCCAGGGCACCTTTACAGCCAACAAGTGAAATTCC	GGTTCTACGATGCTCTGGGGCCCGGGCTCTTGCCCTCCTCTGAGCACG
Rlim ΔSR-motif/y (KI)	CCTTTACAGCCAACAAGTGAAATTCC	ACGCGTAGTCGGCACTTCTG
Rlim W576Y/y (KI), W576Y/y (KI), WT/y (KI)	ACAGCCTCAGCATCTTCTAGAGC	AGTGCATTGGAAAAGTACTGAGCC
gRNA sequences for CRISPR/Cas9	Sense gRNA (5'-3')	Antisense gRNA (5'-3')
Srpk1 (KO)	GAGCAGGAGGAGGAGATTCT	GCGGAGTGGGGTGCAGAGCCT
Srpk2 (KO)	GATTGATGACTTCAAGATCTC	GATGTCTTTGTTTGGGTCACT
Zfp42/Rex1 (KO)	GAGGAAGATGGCTTCCCTGA	GAATCTCACTTTCATCCCGGA
Rlim 4xSA/y (KI)	GAGTTCGTCCTGGAGAATAC	GTACTTGAAGATCAAGAACTA
Rlim ΔSR-motif/y (KI)	GAAGCCGGAGCCCAGAGCAT	GCTCCTCTGAGCTCTGGTGGT
Rlim W576Y/y (KI), W576Y/y (KI), WT/y (KI)	GCAGGGCAGTCTTATCTTCT	GTGGAATTCTCAGACAACCAG
